# Stereospecific Cu(I)-Catalyzed C–O Cross-Coupling Synthesis of Acyclic 1,2-Di- and Trisubstituted Vinylic Ethers from Alcohols and Vinylic Halides

**DOI:** 10.1021/acs.orglett.3c01849

**Published:** 2023-07-12

**Authors:** San L. Pham, Taehee Kim, Frank E. McDonald

**Affiliations:** Department of Chemistry, Emory University, 1515 Dickey Drive NE, Atlanta, Georgia 30322, United States

## Abstract

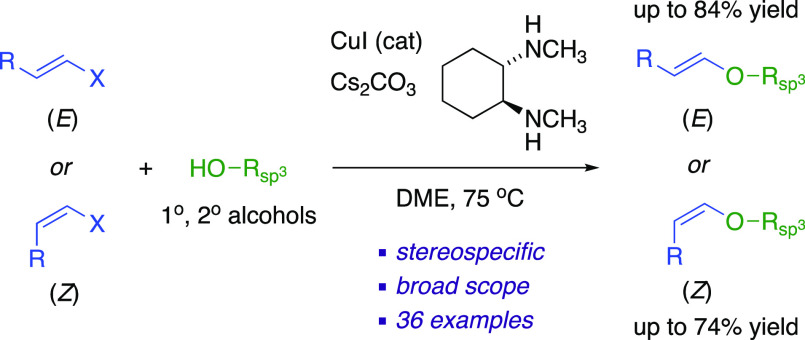

CuI and *trans*-*N*,*N′*-dimethylcyclohexyldiamine catalyze the single-step C–O bond cross-coupling between 1,2-di- and trisubstituted vinylic halides with functionalized alcohols, producing acyclic vinylic ethers. This stereospecific transformation selectively gives each of the (*E*)- and (*Z*)*-*vinylic ether products from the corresponding vinyl halide precursors. This method is compatible with carbohydrate-derived primary and secondary alcohols and several other functional groups. The conditions are mild enough to reliably generate vinylic allylic ethers without promoting Claisen rearrangements.

Vinylic ethers are electron-rich alkenes^1^ with applications including [3.3]-Claisen rearrangements,^2^ cyclization and cycloaddition processes,^3,4^ and bioorthogonal reactions.^5^ Plasmalogen phospholipid natural products feature acyclic (*Z*)-1,2-disubstituted vinylic ethers.^6^ A multistep synthetic route for acyclic disubstituted vinylic ethers via alkynyl ethers (**2**) provides each (*E*)- and (*Z*)-isomer (**3**, **4**) from primary and secondary alcohols, but with limited functionality in the vinylic sector (Figure 1a).^7^ Other methods for acyclic disubstituted vinylic ethers have limited scope^8^ or poor stereoselectivity,^9^ restricting potential applications. Intermolecular single-step C–O bond cross-couplings provide *aromatic ethers* from phenols and/or aromatic synthons,^10,11^ but corresponding syntheses of *substituted vinylic ethers* from less acidic *sp*^3^*-hybridized alcohols* and *substituted vinylic synthons* are not well developed. Cu(II)-catalyzed Chan–Evans–Lam etherifications with vinylic boron synthons require one reactant in excess.^12^ Cu(I)-phenanthroline (**L1**)-catalyzed Ullmann cross-couplings provide (*E*)-disubstituted vinylic ethers, i.e., **7**, although (*Z*)-disubstituted vinylic ethers cannot be prepared (Figure 1b).^13^ This report describes stereospecific Cu(I)-catalyzed C–O cross-couplings with functionalized primary and secondary alcohols, selectively producing (*E*)- and (*Z*)-1,2-disubstituted vinylic ethers (Figure 1c).

**Figure 1:**
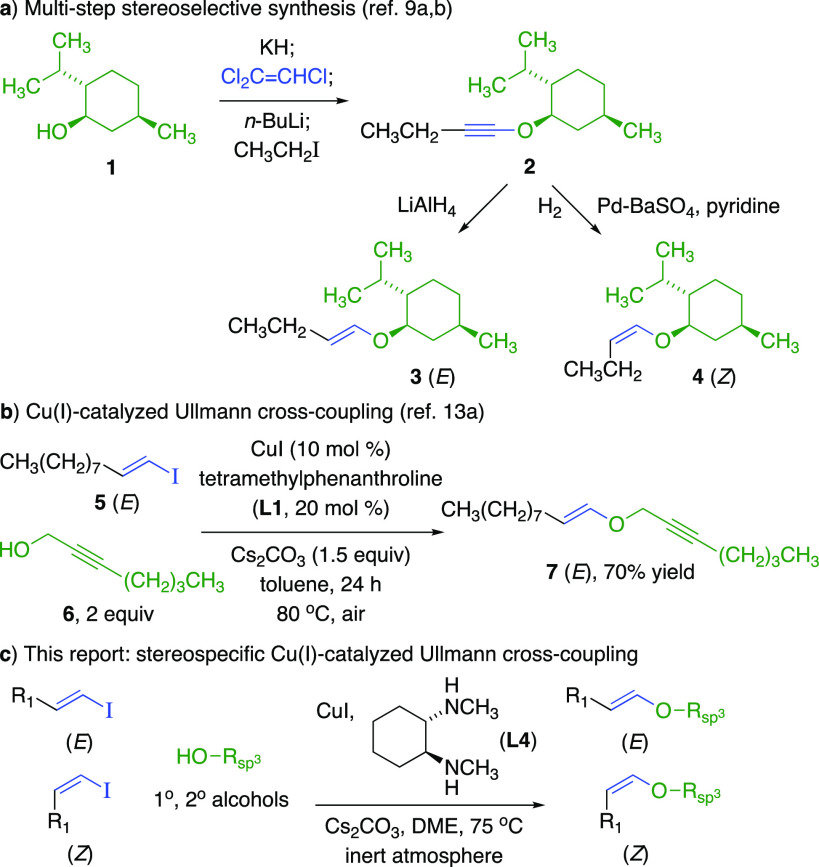
Stereoselective syntheses of 1,2-disubstituted vinylic ethers from alcohols.

We initially applied CuI-phenanthroline (**L1**)-catalyzed literature conditions^13^ with d-galactose-derived primary alcohol **8** (Table 1). Cross-coupling with (*E*)-1-iodo-1-decene (**5**) required 120 °C temperature *and strictly excluding air* (see Supporting Information), giving (*E*)*-*vinylic ether **9** in modest yield (entry 1). Conjugated (*E*)-enyne **10** was a significant side product, consuming two equivalents of vinylic iodide,^14^ as a consequential limitation of the CuI-**L1-**catalyzed method. Hypothesizing that an anionic ligand may promote oxidative addition in the mechanism, *N*,*N*-dimethylglycine (**L2**)^15^ gave an excellent yield of (*E*)*-*vinylic ether **9** at lower temperature (entry 2). The neutral ligand 1,2-dimethylethylenediamine (DMEDA, **L3**) also produced vinylic ether **9** (entry 3). Enyne formation was completely suppressed with *trans*-*N*,*N*′-dimethylcyclohexyldiamine (**L4**, entry 4).^16^ Optimized conditions used 1,2-dimethoxyethane (DME, 0.7 M), 0.2 equiv of CuI, 0.4 equiv of **L4** (CuI:**L4** ratio = 1:2), and 3 equiv of Cs_2_CO_3_, under an argon atmosphere, providing (*E*)*-*vinylic ether **9** in excellent yield *from equimolar vinylic iodide***5***and primary alcohol***8** (entries 5 and 6). The corresponding (*E*)*-*vinylic bromide gave vinylic ether **9** in a slightly lower yield (entry 7). A control experiment with CuI and without ligand gave much lower yield (entry 8).

**Table 1:**
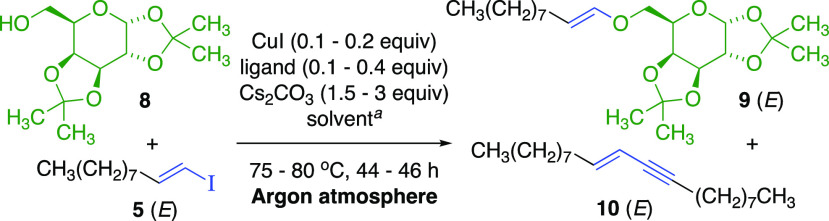

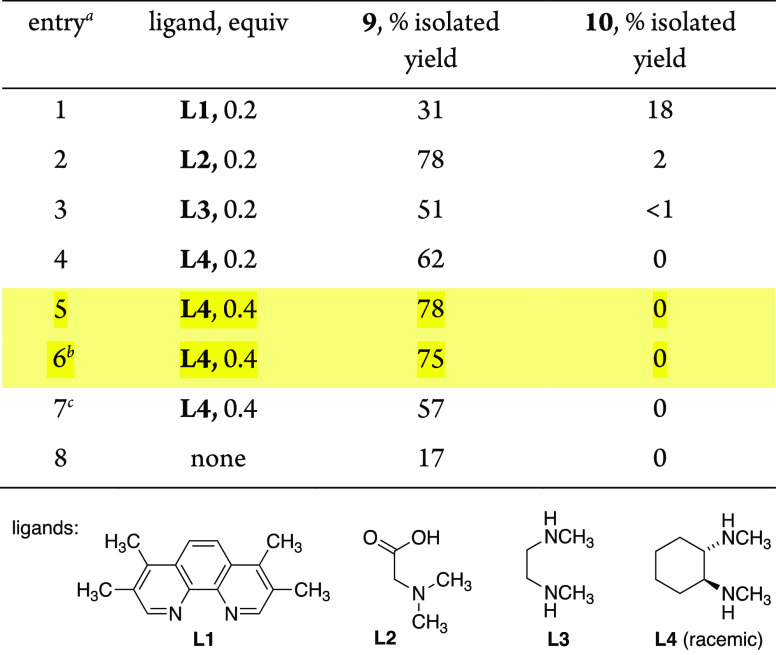
Optimizing C–O Cross-Coupling of (*E*)*-*Vinylic Iodide **5** with Alcohol **8** to Give (*E*)-Vinylic Ether **9**

a *o*-Xylene solvent at 120 °C in entry 1; tetraglyme solvent at 75–80 °C in entries 2–3; and DME solvent in entries 4–7.

b Produced 1.05 g (2.6 mmol) of (*E*)*-*vinylic ether **9**.

c With (*E*)-1-bromo-1-decene (1 equiv).

With (*Z*)-1-iodo-1-undecene (**11**), (*Z*)*-*vinylic ether **12** was not formed using ligand **L1** (Table 2, entry 1), producing only a trace of enyne. With dimethylglycine (**L2**), (*Z*)-enyne **13** predominated over vinylic ether **12**, from base-promoted *anti*-elimination of (*Z*)-vinylic iodide **11** (entry 2).^17^ However, neutral ligands **L3** and **L4** favored the (*Z*)-vinylic ether **12** over enyne **13** (entries 3 and 4). We used 2 equiv of alcohol **8**, 0.5 equiv of CuI, and 1 equiv of **L4** to outcompete the elimination side reaction (entries 5 and 6). Isomerically pure (*Z*)*-*1-iodo-1-decene gave exclusively (*Z*)*-*vinylic ether (entry 7), confirming the stereospecificity. A control experiment with CuI and without ligand only produced the terminal alkyne (entry 8).

**Table 2:**
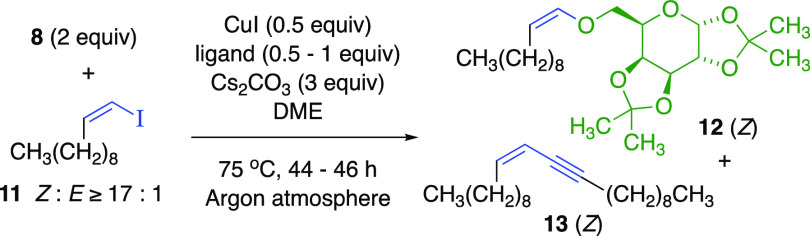

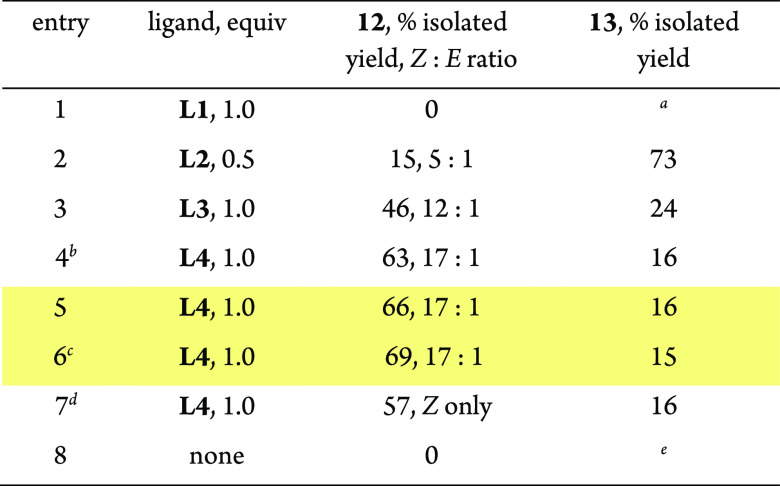
Optimizing C–O Cross-Coupling of (*Z*)*-*Vinylic Iodide **11** with Alcohol **8** to Give (*Z*)-Vinylic Ether **12**

a Trace of enyne, *E*:*Z* = 5:1.

b With 1.2 equiv of alcohol **8**.

c Produced 855 mg (2.1 mmol) of (*Z*)-vinylic ether **12**.

d With 100% (*Z*)-1-iodo-1-decene.

e Only produced 1-undecyne.

Stereospecific CuI-**L4-**catalyzed conditions were tested for other primary alcohols with the *E*/*Z* pair of vinylic iodides **5** and **11** (Figure 2a). For comparison with the literature,^13a^ CuI-**L4**-catalyzed cross-coupling of (*E*)-vinylic iodide **5** with 2-heptyn-1-ol (**6**) gave (*E*)-vinylic ether **7** in excellent yield. Moreover, CuI-**L4** produced (*Z*)-isomer **14** from (*Z*)-vinylic iodide **11**. Cross-couplings with cinnamyl alcohol at 70 °C generated vinylic allylic ethers **15** and **16***with minimal thermal Claisen rearrangement*. (*E*)-Vinylic ether **17** was prepared using only one equivalent of weakly nucleophilic trifluoroethanol.^18^ Vinylic ethers related to *E*/*Z* pairs **19**–**20** and **21**–**22** previously required multistep synthesis from alcohol precursors.^9b,19^ (*E*)-Vinylic ether syntheses were completely stereoselective. (*Z*)-Vinylic ether isomers were strongly favored from (*Z*)-**11**, although yields were generally lower due to enyne formation. Nonetheless, this (*Z*)-vinylic ether synthesis is a substantial advance, with only one previous example known for (*Z*)-vinylic ether from C–O cross-coupling.^12b^

**Figure 2:**
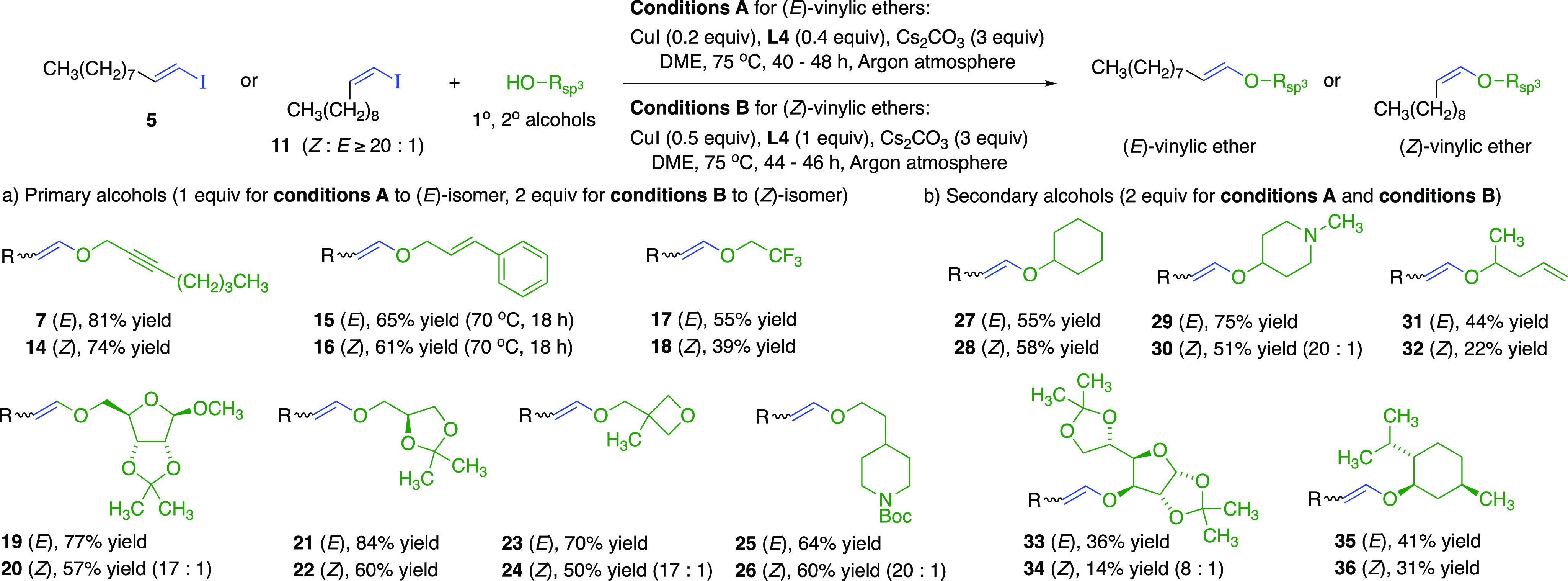
Scope of (*E*)*-* and (*Z*)*-*vinylic ethers synthesized from stereoselective cross-couplings with (a) primary alcohols and with (b) secondary alcohols, with isolated yields. (*Z*)/(*E*) ratios determined by ^1^H NMR analysis prior to chromatographic purification.

The scope of the CuI-**L4**-catalyzed process included several pairs of (*E*)*-* and (*Z*)*-*vinylic ethers **27**–**36** from secondary alcohols (Figure 2b), using two equivalents of secondary alcohols to maximize vinylic ether yields. Despite lower yields with sterically hindered secondary alcohols, this single-step process compared well with multistep routes to (*E*)- and (*Z*)-vinylic ethers related to **33**–**36**.^7b,9b^ This method is compatible with alcohols containing *N*-Boc-protected secondary amines and tertiary amines, giving *E*/*Z* pairs **25**–**26** and **29**–**30**.

We also demonstrated CuI-**L4** cross-couplings with *E*/*Z* pairs of other vinylic iodides, giving **37**–**38** and **39**–**40** (Figure 3a). (*Z*)-Cyclohexylvinyl iodide leading to (*Z*)-vinylic ether **38** was unexpectedly more reactive than the (*E*)-isomer. In contrast, glyceraldehyde-derived (*E*)*-*vinylic iodide gave a better yield of (*E*)-**39** than for (*Z*)-**40** from (*Z*)-vinylic iodide due to competing enyne formation. Trisubstituted (*E*)-1-iodo-2-methyldec-1-ene produced (*E*)*-*vinylic ethers **41**–**43** (Figure 3b). 1-Bromocyclohexene and 1-bromo-2-methyl-1-propene gave the trisubstituted vinylic ethers **44**–**46** (Figure 3c). Geraniol underwent cross-couplings to vinylic allylic ethers **43**, **45**, and **46** without triggering domino Claisen rearrangements.^13a^

**Figure 3:**
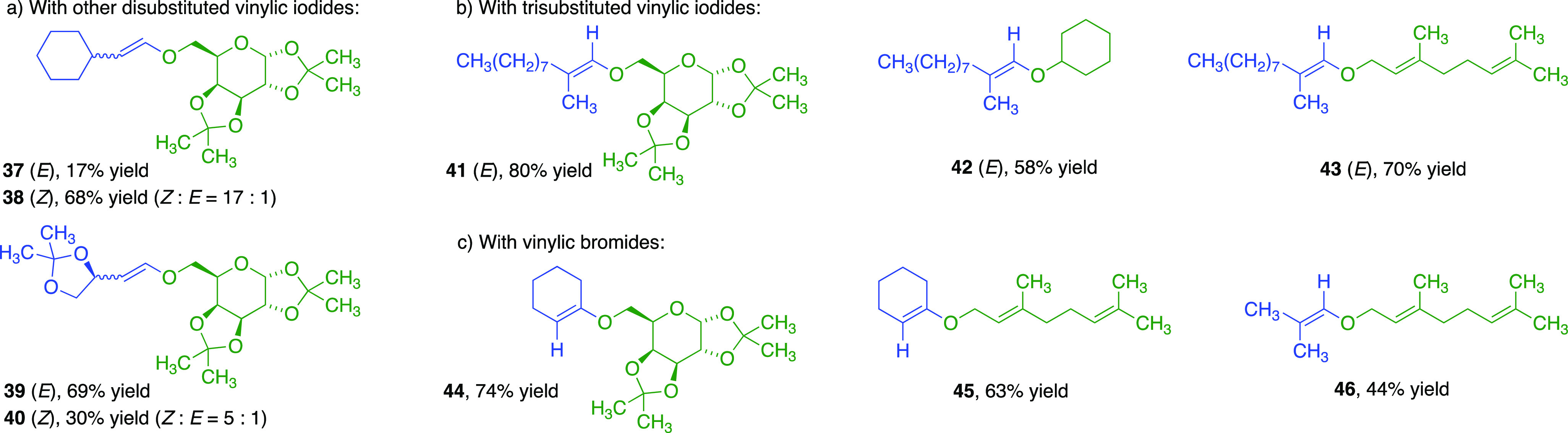
Scope of cross-coupling products with other vinylic halides.

In conclusion, CuI with *trans-N*,*N*′*-*dimethylcyclohexyldiamine (**L4**) promotes stereospecific C–O cross-couplings of vinylic halides with functionalized alcohols, giving vinylic ethers with high stereoselectivity for each (*E*)- and (*Z*)-isomer. The scope of this method is much broader than previous C–O cross-coupling syntheses of vinylic ethers.^12,13^ Notably, ligand **L4** suppresses competing side reactions unique to 1,2-disubstituted vinylic halides. Future research will include mechanistic studies^20^ and synthetic applications of this method.

## Data Availability

The data underlying this study are available in the published article and its online Supporting Information.

## Supporting Information Available

Detailed experimental procedures, compound characterization, and additional optimization experiments (PDF)
Copies of ^1^H and ^13^C NMR spectra for new compounds (PDF)
FAIR data, including the primary NMR FID files, for compounds **7**, **9**, and **12**–**38** (ZIP)
