# Supplementary material for: Stereospecific Cu(I)-Catalyzed C–O Cross-Coupling Synthesis of Acyclic 1,2-Di- and Trisubstituted Vinylic Ethers from Alcohols and Vinylic Halides
Source: Org Lett. 2023 Jul 12;25(28):5297–301. doi: 10.1021/acs.orglett.3c01849 (PMC10367064; doi:10.1021/acs.orglett.3c01849)
Supplement: Supplementary file 1 — ol3c01849_si_001.pdf [file ol3c01849_si_001.pdf]

Supporting Information

Stereospecific Cu(I)-catalyzed C-O cross-coupling synthesis of acyclic 1,2-di- and trisubstituted vinylic ethers from alcohols and vinylic halides

San L. Pham, Taehee Kim, and Frank E. McDonald*

Department of Chemistry, Emory University, 1515 Dickey Drive NE, Atlanta GA 30322 USA

*Corresponding author: fmcdona@emory.edu

Part 1: Detailed experimental procedures, compound characterization, additional optimization experiments

General Experimental 2
Correcting conditions from a published literature example 3
Synthesis of (*E*)-vinylic halides 5
Synthesis of (*Z*)-vinylic iodides 8
Synthesis of trisubstituted vinylic iodide 12
Optimized procedures for stereospecific cross-coupling to synthesize vinylic ethers 13
Additional optimization data for (*E*)-vinylic ether synthesis 18
Additional optimization data for (*Z*)-vinylic ether synthesis 21
Scope of (*E*)- and (*Z*)-selective cross-coupling products from primary alcohols 24
Scope of (*E*)- and (*Z*)-selective cross-coupling products from secondary alcohols 34
Scope of (*E*)- and (*Z*)-selective cross-coupling products with other disubstituted vinylic iodides .41
Scope of (*E*)- and (*Z*)-selective cross-coupling products with trisubstituted vinylic iodides 45
Scope of (*E*)- and (*Z*)-selective cross-coupling products with vinylic bromides 47
References 50

General Experimental

^{1}H, ^{13}C, and ^{19}F NMR spectra were recorded with Varian AVIII 400, INOVA 500, Bruker NEO 400, AVANCE 600 equipped with a cryogen probe, and ASCEND 800 spectrometers. Some structural assignments were made with additional information from gCOSY, gHSQC, and gHMBC experiments. NMR spectra were measured from solutions in deuterated chloroform ($CDCl_3$), using the residual chloroform resonances (7.26 ppm for ^{1}H NMR and 77.16 ppm for ^{13}C NMR) as internal standards, and were reported in parts per million (ppm). As vinylic ethers are susceptible to acid-catalyzed hydrolysis, deuterated chloroform was stored over anhydrous K_2CO_3 to neutralize traces of DCl, and kept dry with anhydrous Na_2SO_4. Abbreviations for NMR signal coupling are as follows: s, singlet; d, doublet; t, triplet; q, quartet; dd, doublet of doublet; ddd, doublet of doublet of doublet; dt, doublet of triplet; m, multiplet; br, broad.

Mass spectra (high resolution ESI and APCI) were recorded on a Scientific Exactive Plus Mass spectrometer, using the orbitrap mass analyzer, which is an electrostatic ion trap. IR spectra were collected on a Thermo Scientific Nicolet iS10 FT-IR spectrometer as neat films on a plate with diamond screw-down tip. Optical rotations were measured using a Perkin-Elmer 341 polarimeter (concentration in g/100mL). Thin layer chromatography (TLC) was performed on a precoated glass backed plates purchased from Silicycle (silica gel 60F254; 0.25 mm thickness), or on precoated aluminum-backed plates purchased from Whatman (silica gel 60F254). Flash column chromatography was carried out with silica gel 60 (230-400 mesh ASTM) from Silicycle, or with neutral alumina (activated, Brockman activity I, 60 Å mean pore size) from Sigma Aldrich and Supelco.

All reactions were conducted with anhydrous solvents in oven-dried glass vials or flasks. Reactions conducted under inert argon atmosphere were conducted by sealing the reaction vessel with a septum, and replacing the atmosphere with argon by three cycles of evacuating the atmosphere with a 10-mL syringe, then backfilling with argon after each evacuation. Reactants were used as received from commercial suppliers without prior purification, as were solvents used for extractions and chromatographic separations. All chemicals were purchased from Sigma Aldrich, Oakwood Chemical, TCI Chemicals, Ambeed, Synthonix, and Combi-blocks. Cs_2CO_3 (99.9% trace metal basis or 99% Reagent Plus) was purchased in 5-25 g quantities from Sigma Aldrich. CuI was purchased from Ambeed or Sigma Aldrich. Zn-Cu couple was purchased from Oakwood Chemicals. Anhydrous THF and DMF were obtained from the Pure Process Purification solvent system. Methanol, isopropanol, acetone, and tetraglyme were used as received from commercial suppliers. Anhydrous DME (with 100 ppm of BHT inhibitor, extra dry (≥99.0%, ≤0.005% H_2O), containing 4Å molecular sieves) was purchased from Acros Organic. Reactions conducted above room temperature were heated: 1) in an external silicone oil bath within an evaporating dish placed on top of a dual stirrer / hot plate (Ceramag Midi), magnetically stirred, with a thermometer measuring the oil bath temperature, or 2) in a 9-vial capacity aluminum heating block (Pie-Blocki CG-1991-P-13*), monitoring temperature with a temperature probe in a 4 mL vial of silicone oil, in the heating block.

Vinylic ethers are highly susceptible to hydrolysis, due to the mildly acidic nature of silica gel used for flash column chromatography. Most vinylic ethers were purified using silica gel pre-treated with at least 2% triethylamine. To prevent hydrolysis, compounds **12**, **S15**, **20**, **22**, and **34** were purified using neutral alumina, but this method was not suitable for purifying other vinylic ethers due to inefficient separation, or loss of product through decomposition.

Correcting conditions from a published literature example[1]

In 2003, Nordmann and Buchwald reported C-O cross-couplings of several primary alcohols with the 1,2-disubstituted (*E*)-vinylic iodide **5**, using catalytic CuI and 3,4,7,8-tetramethyl-1,10-phenanthroline (**L1**) with stoichiometric Cs_2CO_3 in toluene or *o*-xylene solvent, at 80 °C, with reaction times in the range 12 - 36 h.[1] For these examples (in ref. 1, Table 1, entries 4-5, and also in Table 3, entries 1 - 3), *the publication states that air atmosphere was used*. The "General procedure A" in the supporting information describing the experimental protocol for these examples did not indicate that inert atmosphere or air-free technique was required.

In contrast, the examples that combined CuI-**L1**-catalyzed cross-couplings of allylic alcohols + vinylic iodides with Claisen rearrangement of the allylic vinylic ether intermediates were conducted *at 120 °C using argon atmosphere*. The "General procedure B" in the supporting information for these examples also states that argon atmosphere was used. However, this difference implies that argon atmosphere was required only at the higher temperature of 120 °C.

After initially encountering difficulties extending the Nordmann - Buchwald method to other alcohols, we repeated an example in the 2003 paper to validate our technique, preparing (*E*)-vinylic ether **S1** from (*E*)-vinylic iodide **5** with benzyl alcohol:

From ref. 1, Table 3, entry 3:

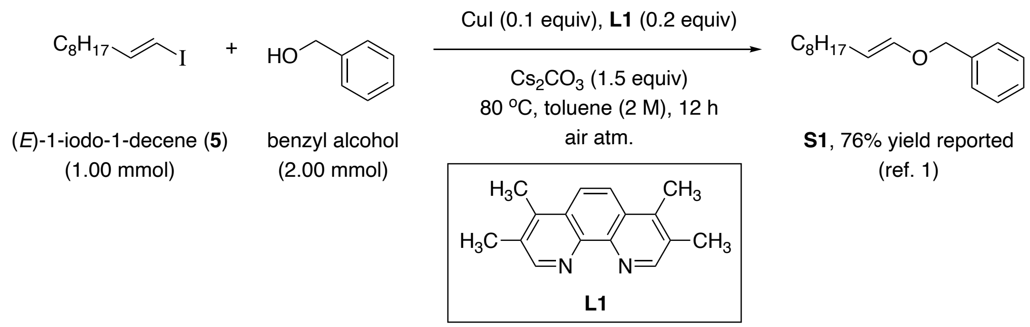

Our deviations from the published experimental procedure (ref. 1) are described in **bold**. We **did not use dodecane as an internal standard**, but relied on the isolated mass of purified product **S1** and calculated the isolated yield relative to the limiting reactant, (*E*)-1-iodo-1-decene (**5**).

1) An early validation experiment was conducted on **30% of the scale** reported in ref. 1, with **0.2 equiv CuI** and **0.4 equiv L1**, to ensure that enough catalyst and ligand was present. The reactants were weighed in air, and the reaction was performed in a sealed vial under air. Specifically:

A **5 mL conical vial** was charged with benzyl alcohol (65 mg, **0.60 mmol**, 2.0 equiv), CuI (11.4 mg, 0.06 mmol, **0.2 equiv** relative to vinylic iodide **5**), **L1** (28.4 mg, 0.12 mmol, **0.4 equiv** relative to vinylic iodide **5**), (*E*)-vinylic iodide **5** (80 mg, **0.30 mmol**, 1.0 equiv), Cs_2CO_3 (146.9 mg, 0.45 mmol, 1.5 equiv relative to vinylic iodide **5**) and toluene (0.2 mL, **1.5 M** based on vinylic iodide **5**). The vial was sealed with a screw cap. The reaction mixture was vigorously stirred at 80 ºC for **17 h**. The resulting suspension was cooled to room temperature and filtered through a 1 x 2 cm pad of silica gel eluting with diethyl ether (100 mL).The filtrate was concentrated by rotary evaporation, and the residue was purified by flash chromatography on silica gel (**eluent = 100% hexanes**) to afford pure product **S1** (25.6 mg, **35% yield**).

2) A subsequent validation experiment was conducted on the same scale, using 1 mmol of (*E*)-vinylic iodide **5** as the limiting reactant, **which contained 8% 1-decene**. Specifically:

A **5 mL conical vial** was charged with benzyl alcohol (216 mg, 2.0 mmol, 2.0 equiv), CuI (19.0 mg, 0.1 mmol, 0.1 equiv), **L1** (47.3 mg, 0.2 equiv), (*E*)-vinylic iodide **5** (266 mg, 1.0 mmol, 1.0 equiv), Cs_2CO_3 (489 mg, 1.5 mmol, 1.5 equiv) and toluene (0.5 mL, 2 M). **The vial was topped with a water-cooled condenser, topped with a septum, connected by a needle to an argon line.** (The reaction vessel was not purged.) The reaction mixture was vigorously stirred at 80 ºC for **17 h**. The resulting suspension was cooled to room temperature and filtered through a 1 x 2 cm pad of silica gel eluting with diethyl ether (100 mL).The filtrate was concentrated by rotary evaporation, and the residue was purified by flash chromatography on silica gel (**eluent = 100% hexanes**) to afford pure product **S1** in (118 mg, **53% yield** when accounting for the 1-decene impurity in the sample of vinylic iodide **5**).

3) A final validation experiment was conducted on **80% of the scale** reported in ref. 1. The sample of (*E*)-vinylic iodide **5** used in this experiment **contained 10% 1-decene**. Specifically:

A **4-mL flat bottom vial** was charged with benzyl alcohol (171 mg, **1.6 mmol**, 2.0 equiv), CuI (15.0 mg, 0.08 mmol, 0.1 equiv), **L1** (37.3 mg, 0.16 mmol, 0.2 equiv), (*E*)-vinylic iodide **5** (210 mg, **0.8 mmol**, 1.0 equiv), Cs_2CO_3 (386 mg, 1.5 equiv) and toluene (0.4 mL, 2 M). **The vial was sealed with a septum, evacuated by three cycles removing the atmosphere with 10-mL syringe, then backfilling with argon after each evacuation.** The reaction mixture was vigorously stirred at 80 ºC for **13 h**. The resulting suspension was cooled to room temperature and filtered through a 1 x 2 cm pad of silica gel eluting with diethyl ether (100 mL).The filtrate was concentrated on rotary evaporator, and the residue was purified by flash chromatography on silica gel (**eluent = 100% hexanes**) to afford pure product **S1** (129 mg, 73% yield when accounting for the 1-decene impurity in the sample of vinylic iodide **5**).

From these validation experiments, we concluded that *air-free technique was essential* to the success of the CuI-**L1**-catalyzed C-O cross-coupling reaction.

Synthesis of (*E*)-vinylic halides

(*E*)-vinylic halide substrates were synthesized from commercially available alkynes via hydrozirconation - halogenation. For vinylic iodide **S2**, the precursor alkyne **S6** was synthesized from commercially available D-glyceraldehyde acetonide **S4**.

General protocol for (*E*)-vinylic iodides:

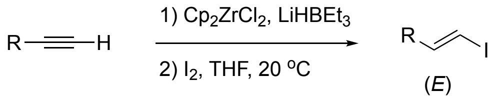

The following procedure has been modified from the published synthesis of (*E*)-vinyl iodide via hydrozirconation - iodination:[2]

An oven-dried reaction vessel (250-mL round bottom flask for the 20 mmol scale of decyne or 5-mL conical vial for 1-2 mmol of other alkynes) was charged with zirconocene dichloride (Cp_2ZrCl_2, 1.7 equiv) and corresponding alkyne in tetrahydrofuran (THF, 0.35 M). To this solution at room temperature was added a 1.0 M THF solution of lithium triethylborohydride ($LiHBEt_3$, 1.3 equiv). The cloudy solution turned clear orange or yellow. After 10 min of stirring, approximately 1 M solution of iodine (1.2 equiv) in THF was added dropwise until the brown color persisted. After additional stirring at rt for 10 min, the reaction mixture was quenched with a 1N HCl solution and then extracted with diethyl ether (× 3). The organic layer was washed with an aqueous solution of saturated $Na_2S_2O_3$ and brine, dried over $MgSO_4$, filtered the solution through a filter paper and concentrated on rotary evaporator. The details of column chromatography and yields are listed below. The characterization data for various vinyl iodides **5**,[3] **S2**,[4] and **S6**[5] matched the published data.

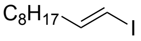

5

Synthesis of (*E*)-1-iodo-1-decene (5): Prepared 3.915 g (74% yield); eluent = pure hexanes

Characterization data for (*E*)-1-iodo-1-decene (**5**):[3]

^{1}H NMR (400 MHz, $CDCl_3$) δ 6.51 (dt, *J* = 14.3, 7.2 Hz, 1H), 5.97 (dt, *J* = 14.4, 1.4 Hz, 1H), 2.04 (qd, *J* = 7.2, 1.5 Hz, 2H), 1.43 – 1.21 (m, 12H), 0.94 – 0.81 (t, 3H).

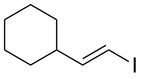

S2

Synthesis of (*E*)-(2-iodovinyl)cyclohexane (S2): Prepared 248.2 mg (76% yield), eluent = pure pentane; solvent was evaporated using rotary evaporator with bath temperature 5-10 °C.

Characterization data for (*E*)-(2-iodovinyl)cyclohexane (**S2**):[4]

^{1}H NMR (400 MHz, $CDCl_3$) δ 6.48 (dd, *J* = 14.5, 7.1 Hz, 1H), 5.95 (dd, *J* = 14.5, 1.3 Hz, 1H), 2.00 (dddd, *J* = 11.2, 5.5, 4.6, 2.8 Hz, 1H), 1.77 – 1.68 (m, 4H), 1.64 (ddq, *J* = 12.6, 3.6, 1.8 Hz, 1H), 1.37 – 1.03 (m, 5H).

Synthesis of (*S*,*E*)-4-(2-iodovinyl)-2,2-dimethyl-1,3-dioxolane (S6): Prepared in two steps via (*S*)-4-ethynyl-2,2-dimethyl-1,3-dioxolane (**S5**).

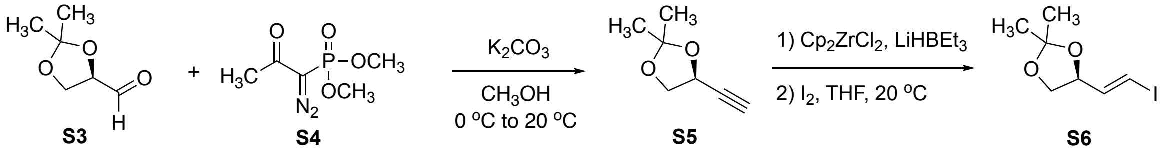

A solution of D-glyceraldehyde acetonide **S3** (50 w/w % in CH_2Cl_2, 0.956 g, 3.67 mmol) and the Bestmann–Ohira reagent **S4** (1.41 g, 7.35 mmol; synthesized according to the published literature)[6] in methanol (18 mL) was cooled to 0 °C. Anhydrous K_2CO_3 (1.02 g, 7.35 mmol) was added in four portions over 30 min. The mixture was stirred for 15 h, while slowly warming to room temperature. Saturated aqueous ammonium chloride (50 mL) was added, and the aqueous solution was extracted with pentane (50 mL × 3). The organic layer was separated, dried over $MgSO_4$, and then filtered with filter paper. The solvent was carefully evaporated on rotary evaporator (bath 5-10 °C). Due to its volatility, the crude alkyne **S5** was filtered through a short pad of Celite®/silica gel, then the short plug was washed with pentane/diethyl ether 10:1 (50 mL). Terminal alkyne **S5** (0.410 g, 88% yield) was isolated as a colorless oil. The ^{1}H NMR spectrum matched the published data.[7]

Characterization data for (*S*)-4-ethynyl-2,2-dimethyl-1,3-dioxolane (**S5**):

^{1}H NMR (400 MHz, $CDCl_3$) δ 4.71 (td, *J* = 6.3, 2.1 Hz, 1H), 4.18 (dd, *J* = 8.1, 6.3 Hz, 1H), 3.95 (dd, *J* = 8.1, 6.2 Hz, 1H), 2.50 (d, *J* = 2.1 Hz, 1H), 1.50 (d, *J* = 0.8 Hz, 3H), 1.39 (d, *J* = 0.7 Hz, 3H).

Alkyne **S5** (50 mg, 0.39 mmol) was converted into the (*E*)-vinylic iodide **S6** following the general hydrozirconation - iodination procedure, giving 69.4 mg (69% yield), eluent = 10:1 pentane / diethyl ether; solvents were removed using rotary evaporator with bath temperature 5-10 °C.

* Subsequent C-O cross-coupling with 1,2:3,4-di-*O*-isopropylidene-α-D-galactopyranose (**8**) revealed that vinylic iodide **S6** was produced as an 89 : 11 mixture of enantiomers. Stereochemical erosion is attributed to this step, preparing the alkyne **S5** from the epimerizable aldehyde **S3**.

Characterization data for (*S*,*E*)-4-(2-iodovinyl)-2,2-dimethyl-1,3-dioxolane (**S6**):[5]

^{1}H NMR (400 MHz, $CDCl_3$) δ 6.55 (dd, *J* = 14.5, 6.1 Hz, 1H), 6.50 (d, *J* = 14.5 Hz, 1H), 4.48 (dt, *J* = 7.1, 6.1 Hz, 1H), 4.11 (dd, *J* = 8.3, 6.3 Hz, 1H), 3.65 (dd, *J* = 8.4, 7.1 Hz, 1H), 1.43 (d, *J* = 0.8 Hz, 3H), 1.38 (q, *J* = 0.7 Hz, 3H).

$[\alpha]^{22}_D$ +3.8 (c = 0.1, $CHCl_3$)

Synthesis of (*E*)-1-bromo-1-decene (S7):

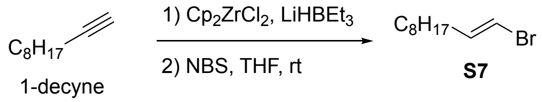

In an oven-dried 25mL round bottom flask was charged with Cp_2ZrCl_2 (987 mg, 1.7 equiv.) and 1-decyne (0.36 mL, 1.0 equiv) in THF (5.5 mL, 0.35 M). To this solution at room temperature was added a 1.0 M THF solution of $LiHBEt_3$ (2.6 mL, 1.3 equiv). After 10 min of stirring, *N*-bromosuccinimide (NBS, 471 mg, 1.2 equiv) was added. After additional stirring for 30 min, the reaction mixture was quenched with a 1N HCl solution (50 mL) and then extracted with diethyl ether (50 mL× 3). The organic layer was washed with aqueous saturated $NaHCO_3$ (50 mL) and brine (50 mL), dried over $MgSO_4$, and then filtered on filter paper. The solvent was concentrated on rotary evaporator. The crude was dissolved in hexanes, and white solids crashed out. The solution was filtered through a short pad of Celite®/silica gel and washed with hexanes to yield (*E*)-1-bromo-1-decene as a colorless oil (0.391 g, 89% yield). The ^{1}H NMR spectrum data matched the published data.[8]

Characterization data for (*E*)-1-bromo-1-decene **(S7):**

^{1}H NMR (400 MHz, $CDCl_3$) δ 6.17 (dt, *J* = 13.4, 7.2 Hz, 1H), 6.00 (dt, *J* = 13.5, 1.4 Hz, 1H), 2.03 (qd, *J* = 7.2, 1.4 Hz, 2H), 1.45 – 1.21 (m, 12H), 0.92 – 0.81 (m, 3H).

Synthesis of (*Z*)-vinylic iodides

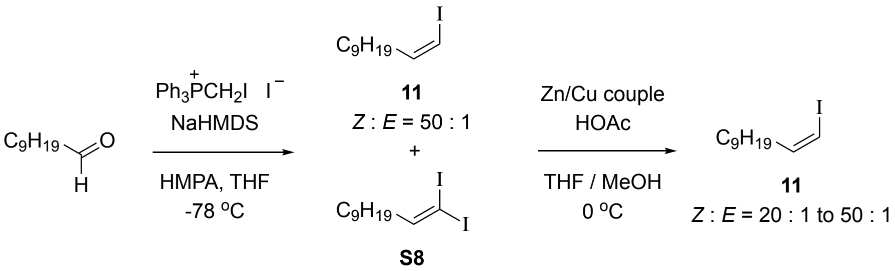

Synthesis of (*Z*)-1-iodo-1-undecene (11) via Wittig reaction:[9]

> Note: Due to availability of even-numbered aldehydes, decanal ($C_{10}H_{20}O$) was used instead of nonanal, with one-carbon homologation giving (*Z*)-1-iodo-1-undecene (**11**).

Step 1: (*Z*)-1-iodo-1-undecene (crude **11**, 50:1 *Z/E*, containing 15% 1,1-diiodo-1-undecene **S8**): In a 250 mL oven-dried round bottom flask charged with a stirbar and wrapped in aluminum foil, (iodomethyl)triphenylphosphonium iodide (15.0 g, 28.5 mmol, 1 equiv) was added and placed under inert atmosphere by purging with a constant stream of argon gas. Dry THF (71 mL, 0.4 M based on phosphonium salt, freshly distilled) was added to the reaction flask, followed by the dropwise addition of sodium hexamethyldisilazide (NaHMDS, 1 M in THF, 29 mL, 1 equiv) over 5 minutes in the dark at room temperature to give an orange liquid. After the addition, the mixture was stirred at room temperature for 20-30 minutes, resulting in a dark red liquid with some precipitate. The reaction flask was then cooled to -78 °C in a dry ice/acetone bath, and hexamethylphosphoramide (HMPA, 9 mL) was added. The mixture was stirred for approximately 5 minutes before adding decanal (4.2 mL, 22.8 mmol, 0.8 equiv) to the reaction mixture. For optimal Z/E ratio, the reaction was stirred vigorously at -78 °C in the dark under argon for 3 h to give a thick dark orange mixture. The reaction mixture warmed to about 10-15 °C, then saturated $NaHCO_3$ solution (~36 mL) was added to quench the reaction, and hexanes were added to dilute the reaction mixture, which gave a solution of orange liquid and a yellow-white precipitate. The mixture was filtered through a Celite® plug on a 100 mL fritted funnel, using hexanes and water to rinse. The organic layer was separated from the water layer in a 500 mL separatory funnel, and the aqueous layer was extracted twice with hexanes. The organic layer was filtered twice through a silica plug, using 100 mL fritted funnel. The silica plug was washed with additional hexanes, and the total organic layer was washed with saturated $Na_2S_2O_3$ solution, dried with anhydrous Na_2SO_4, filtered, and concentrated to give a yellow crude oil of vinylic iodide (4.2 g). The crude mixture was analyzed by ^1H NMR, to determine the Z/E ratio of the vinylic iodide **11** (50:1 Z/E) and the content of 1,1-diiodo-1-undecene (**S8**, approximately 15%) by integrating the following alkenyl proton resonances:

- 6.94 ppm (t, *J* = 7.0 Hz, 1H) for 1,1-diiodo-1-undecene (**S8**)
- 6.54 ppm (dt, *J* = 14.3, 7.2 Hz, 1H) for (*E*)-1-iodo-1-undecene
- 6.22-6.11 ppm (m, 2H) for (*Z*)-1-iodo-1-undecene (**11**).

The crude mixture was subsequently subjected to Zn-Cu-mediated stereoselective reduction without further purification.[10]

Step 2: (Z)-1-iodo-1-undecene (20-50:1 *Z/E*) (**11**): An argon-flushed 25 mL round bottom flask was charged with crude **11** (4.2 g, containing 2.1 mmol of 1,1-diiodo-1-undecene **S8**). The reaction flask was wrapped with aluminum foil, placed in an ice bath, and dissolved in dry THF (70 mL, 0.03 M based on 1,1-diiodo-1-undecene) and MeOH (40 mL, 0.05 M based on 1,1-diiodo-1-undecene). The mixture was stirred for 5 min, then concentrated acetic acid (12 mL, 100 equiv based on 1,1-diiodo-1-undecene) was added, followed by Zn-Cu couple (4.2 g, 32 mmol, 16 equiv based on 1,1-diiodo-1-undecene). The reaction mixture was stirred in an ice bath in the dark for 1.5 h.* The mixture was filtered over a Celite® plug (100 mL fritted funnel) to give a light orange-pink clear solution, using hexanes to rinse the flask and the Celite® plug. The filtrate was transferred into a separatory funnel, washed with saturated aqueous $NaHCO_3$, whereupon the organic layer became slightly cloudy white. The organic layer was washed with saturated $Na_2S_2O_3$ and brine to give a clear liquid, which was dried over Na_2SO_4. The organic layer was filtered and concentrated to give the title compound **11** as a pale clear oil (3.7 g, 58% overall yield over two steps, 20:1 *Z*/*E*; 0% 1,1-diiodo-1-undecene based on ^{1}H NMR analysis). The ^{1}H and ^{13}C spectra matched the data reported for similar iodoalkenes.[11]

*25 - 50:1 *Z/E* mixture was obtained in an experiment when reaction mixture was stirred for 1 h.

Characterization data for (*Z*)-1-iodo-1-undecene (**11**, 50:1 *Z/E*):

^{1}H NMR (400 MHz, $CDCl_3$) δ 6.22 – 6.11 (m, 2H), 2.20 – 2.08 (m, 2H), 1.41 (apparent quintet, *J* = 7.0 Hz, 2H), 1.27 (m, 12H), 0.92 – 0.84 (m, 3H).

^{13}C NMR (101 MHz, $CDCl_3$) δ 141.7, 82.3, 34.8, 32.0, 29.7, 29.6, 29.5, 29.3, 28.1, 22.8, 14.3.

HRMS (APCI): *m/z* calcd for $C_{11}H_{22}I$ $[M+H]^+$ 281.0761, found 281.0750.

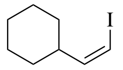

S9

(*Z*)-(2-iodovinyl)cyclohexane (**S9**, 17:1 *Z/E*): The compound was synthesized similarly to the synthesis of iodide **11** using (iodomethyl)triphenylphosphonium iodide (10 g, 19 mmol, 1 equiv) and cyclohexylcarbaldehyde (1.7 g,15 mmol, 0.8 equiv). After workup, a yellow crude oil (1.2 g, 30:1 *Z/E*, containing 25% 1,1-diiodoalkene) was obtained and analyzed by ^{1}H NMR using the integrations of the following alkenyl protons:

- 6.77 (d, *J* = 8.7 Hz, 1H) for 1,1-diiodoalkene
- 6.49 ppm (dd, *J* = 14.4, 7.1 Hz,1H) for *E*-isomer **S2**
- 6.06 ppm (dd, *J* = 7.3, 0.7 Hz, 1H) for *Z*-isomer **S9**

Without further purification, 930 mg of the iodoalkene mixture was subjected to Zn-Cu-mediated stereoselective reduction. The title compound **S9** was furnished as a clear yellow oil (630 mg, quantitative yield from the second step, 17:1 *Z/E*, no 1,1-diiodoalkene detected, >95% pure based on ^{1}H NMR analysis). The ^{1}H NMR spectrum matched the published values.[11]

Characterization data for (*Z*)-(2-iodovinyl)cyclohexane (**S9**, 17:1 *Z/E*):[11]

^{1}H NMR (400 MHz, $CDCl_3$) δ 6.09 (dd, *J* = 7.3, 0.7 Hz, 1H), 6.01 (dd, *J* = 8.5, 7.3 Hz, 1H), 2.40 – 2.30 (m, 1H), 1.79 – 1.63 (m, 5H), 1.42 – 1.29 (m, 2H), 1.27 – 1.10 (m, 3H).

HRMS (APCI): *m/z* calcd for $C_8H_{14}I$ $[M+H]^+$ 237.0135, found 237.0129.

Synthesis of (*S*,*Z*)-4-(2-iodovinyl)-2,2-dimethyl-1,3-dioxolane (S10):[12]

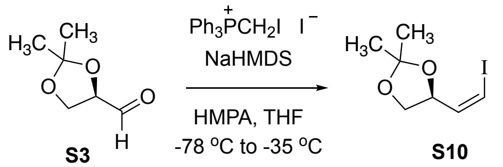

In a 250 mL oven-dried round bottom flask charged with a stirbar and wrapped in aluminum foil, (iodomethyl)triphenylphosphonium iodide (4.5 g, 8.5 mmol) was added (with minimal exposure to light) and placed under inert atmosphere via purging with a constant stream of argon gas. A portion of dry THF (10 mL, freshly distilled) was added to the reaction flask, followed by the dropwise addition of NaHMDS (1 M in THF, 8.5 mL) over 5 – 10 minutes in the dark at room temperature (wrapped in aluminum foil) to give an orange liquid. After the addition, the mixture was stirred at room temperature for 20-30 minutes, resulting in a dark red liquid with some precipitate. While waiting for ylide formation, D-glyceraldehyde acetonide (**S3**, 50 wt% in CH_2Cl_2, 1.77 g, 6.8 mmol) was weighed by pouring directly into a separate 25 mL round bottom flask. The aldehyde was placed under argon atmosphere and dissolved in dry THF (11 mL); this required constant swirling over 10 minutes to dissolve the aldehyde. The reaction flask for ylide generation was cooled to -78 °C with a dry ice/acetone bath, and HMPA (2.6 mL) was added. The aldehyde/THF solution was added to the reaction flask, and the reaction mixture was stirred in the dark, at -78 °C to -35 °C, over 3 hours. After completion of the reaction, saturated $NaHCO_3$ (12 mL) was added to quench and ethyl acetate (EtOAc) was used to dilute the reaction. The mixture was filtered through a Celite® plug (on a 100 mL fritted funnel) using EtOAc and water to rinse. Then, the organic layer was separated from the aqueous layer, and the organic components were extracted two more times from the aqueous layer before being washed with saturated $Na_2S_2O_3$ solution and brine, dried with anhydrous Na_2SO_4, filtered, and concentrated to give a yellow crude oil. The crude mixture was dissolved in 10:1 hexanes/EtOAc (100 mL), which caused precipitation to crash out, and was filtered through a silica plug using 200 mL of the same eluent to rinse the crude flask. The filtrate was concentrated to furnish the title compound **S10** as a yellow oil (1.17 g, 68% yield, 10: 1 *Z*/*E* containing <2% of 1,1-diiodoalkene). The mixture was analyzed by ^{1}H NMR for the Z/E ratio and the content of 1,1-diiodoalkene using the integrations of the following alkenyl protons:

- 7.15 (d, *J* = 7.3 Hz, 1H) for 1,1-diiodoalkene
- 6.59 – 6.46 (m, 2H) for *E*-isomer **S6**
- 6.46 – 6.35 ppm (m, 2H) for *Z*-isomer **S10**.

The characterization data matches previous literature.[12] Vinylic iodide **S10** was used in subsequent reactions without further purification.

* Subsequent C-O cross-coupling with 1,2:3,4-di-*O*-isopropylidene-α-D-galactopyranose (**8**) revealed that vinylic iodide **S10** was produced as an 91 : 9 mixture of enantiomers. Stereochemical erosion is attributed to this step, preparing vinylic iodide **S10** from the epimerizable aldehyde **S3**.

Characterization data for (*S*,*Z*)-4-(2-iodovinyl)-2,2-dimethyl-1,3-dioxolane (**S10**):[12]

^{1}H NMR (400 MHz, $CDCl_3$) δ 6.44 (dd, *J* = 7.8, 1.1 Hz, 1H), 6.38 (dd, *J* = 7.8, 7.0 Hz, 1H), 4.78 (tdd, *J* = 7.1, 6.3, 1.1 Hz, 1H), 4.24 (dd, *J* = 8.3, 6.4 Hz, 1H), 3.65 – 3.58 (m, 1H), 1.45 – 1.43 (m, 3H), 1.41 (t, *J* = 0.7 Hz, 3H).

^{13}C NMR (101 MHz, $CDCl_3$) δ 139.9, 110.0, 83.9, 78.7, 68.3, 26.7, 25.8.

$[\alpha]^{22}_{D}$ -15.4 (c = 0.34, $CHCl_3$).

Attempts to characterize compound **S10** by APCI-MS gave only fragmentation, with no evidence of a molecular ion corresponding to formula $C_7H_{11}IO_2$ or $[M+H]^+$ $C_7H_{12}IO_2^+$.

Synthesis of 100% (*Z*)-1-iodo-1-decene (S12) via *cis*-hydrogenation of 1-iodo-1-decyne (**S11**):

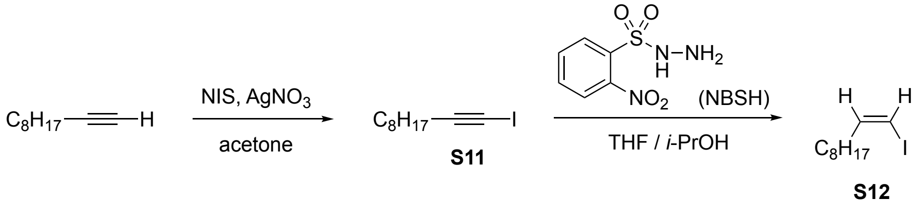

1-iodo-1-decyne (**S11**): To a solution of 1-decyne (2 g) in acetone (72 mL, purged with argon balloon) under argon atmosphere in the dark, *N*-iodosuccinimide (NIS, 3.9 g) was added followed by $AgNO_3$ (296 mg). The reaction was stirred overnight at room temperature under argon in the dark to give a pale clear liquid with yellow precipitate. The reaction was diluted with deionized H_2O and hexanes to give an orange mixture, which was then transferred into a separatory funnel. The aqueous layer was extracted with hexanes three times, and the combined organic layer was washed in sequence with deionized H_2O, saturated aqueous $Na_2S_2O_3$, and brine. The combined organic extracts were filtered through a silica plug, and rinsed with hexanes. The filtrate was concentrated at medium vacuum with the water bath at 20 °C to avoid product evaporation, to give a pink oil. ^{1}H NMR signals matched the literature report.[13] The crude product **S11** (3.2 g) was used without further purification.

Characterization data for 1-iodo-1-decyne (**S11**):

^{1}H NMR (500 MHz, $CDCl_3$) δ 2.36 (t, *J* = 7.1 Hz, 2H), 1.56 – 1.46 (m, 2H), 1.45 – 1.33 (m, 2H), 1.27 (dq, *J* = 10.5, 5.0 Hz, 8H), 0.88 (t, *J* = 6.8 Hz, 3H).

Synthesis of *ortho*-nitrobenzenesulfonylhydrazide (NBSH): To a 50 mL round bottom flask charged with a stir bar, *ortho*-nitrobenzenesulfonyl chloride (4 g) was added, purged with argon atmosphere, and dissolved with dry THF (18 mL). The reaction flask was cooled to -30 °C using a carefully monitored cold bath of dry ice and acetone. Hydrazine hydrate (N_2H_4-H_2O, 2.2 mL) was carefully added to the mixture, and the reaction was stirred at -30 °C over 30 minutes. After stirring was complete, 40 mL of ethyl acetate was added to dilute the reaction, and the organic layer was washed with 10% aqueous NaCl solution (w/v) (five times with 30 mL portions). The organic layer was dried with anhydrous Na_2SO_4 and filtered slowly into ~210 mL of hexanes in a separate flask over 5 minutes, which caused a precipitate to crash out of solution. The flask was stored in a freezer overnight. The solid precipitate was filtered and washed with cold hexanes (~100 mL total). The solid was dried overnight at high vacuum, to give NBSH as a pale yellow flaky solid (3.2 g, 82% yield).[14]

(*Z*)-1-iodo-1-decene (**S12**): 1-Iodo-1-decyne (**S11**, 869 mg, 3.3 mmol) was added to a 250 mL round bottom flask charged with a stir bar, and then purged with argon atmosphere. Compound **S10** was dissolved with *i*-PrOH (41.5 mL) and dry THF (41.5 mL). The reaction mixture was cooled to 0 °C in an ice bath and wrapped with aluminum foil. Freshly prepared NBSH (1.5 g, 6.9 mmol, 2.1 equiv) was added under argon, followed by triethylamine (1.6 mL, 12 mmol, 3.5 equiv). The reaction mixture slowly warmed to room temperature overnight. After 19 hours, the reaction mixture was dark clear yellow, which was concentrated by rotary evaporation to give an orange residue with pale yellow oil. The crude compound was dissolved in a minimum amount of CH_2Cl_2 and loaded onto 4 g of Celite®, and purified by silica gel chromatography, eluting with 100% hexanes. The desired product was collected (note: **S12** was UV-active and the first band to elute), and concentrated to give a clear colorless oil (450 mg, 51% yield, containing 5% iododecane). The ^{1}H NMR spectrum of the product **S12** matched previous literature.[13]

Characterization data for (*Z*)-1-iodo-1-decene (**S12**):[13]

^{1}H NMR (400 MHz, $CDCl_3$) δ 6.22 – 6.11 (m, 2H), 2.13 (dtd, J = 7.5, 5.4, 1.7 Hz, 2H), 1.48 – 1.37 (m, 2H), 1.36 – 1.20 (m, 10H, overlapping with 1-iododecane), 0.92 – 0.81 (m, 3H).

Synthesis of trisubstituted vinylic iodide

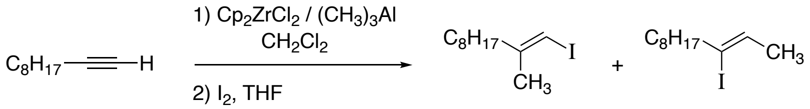

S13 95 : 5 **S14**

(*E*)-1-iodo-2-methyldec-1-ene (**S13**): The reaction protocol was modified based on reported procedures.[15-17] To an argon-charged 100 mL oven-dried round bottom flask, Cp_2ZrCl_2 (292 mg, 1 mmol) was added to dry CH_2Cl_2 (20 mL), followed by slow addition of $Al(CH_3)_3$ (2M in heptane, 7.5 mL) at room temperature (CAUTION: Smoke was observed). The reaction mixture was cooled to 0 °C over 10 min, and deionized water (0.13 mL) was added dropwise. The reaction mixture was then warmed to room temperature and stirred for 20 min, followed by the addition of 1-decyne (0.9 mL, 5 mmol). After stirring for 30 min, an argon-charged solution of iodine (1.42 g, 6 mmol) in dry THF (7.5 mL) was added to the reaction mixture, which was then stirred for an additional 30 min. The reaction was quenched by careful pouring into saturated aqueous K_2CO_3 (50 mL) (CAUTION: vigorous bubbling occurs) and was filtered through a Celite® pad using CH_2Cl_2 to rinse the reaction vessel. The organic solution was washed with saturated aqueous $Na_2S_2O_3$ and brine, dried with anhydrous Na_2SO_4, filtered and concentrated to give a clear light-yellow crude oil (730 mg). The crude mixture was purified by column chromatography using silica gel in 100% pentane to furnish the title compound (495 mg, 95:5 regioisomer ratio **S13**:**S14**, also containing ~15% of 2-methyl-1-decene, determined by ^{1}H NMR analysis). The ^{1}H NMR spectrum of the pure product matches reported values from literature.[18]

Characterization data for (*E*)-1-iodo-2-methyldec-1-ene (**S13**):[18]

^{1}H NMR (400 MHz, $CDCl_3$) δ 5.85 (apparent sextet, *J* = 1.2 Hz, 1H), 2.23 – 2.14 (m, 2H), 1.82 (d, *J* = 1.1 Hz, 3H), 1.48 – 1.36 (m, 2H), 1.26 (m, 10H, overlapping with other alkene side products), 0.91 – 0.86 (m, 3H).

HRMS (APCI): *m/z* calcd for $C_{11}H_{22}I$ $[M+H]^+$ 281.0761, found 281.0752.

Optimized procedures for stereospecific cross-couplings to synthesize vinylic ethers

Optimized procedure for synthesizing 1,2-disubstituted (*E*)-vinylic ether 9 (Table 1, entry 5):

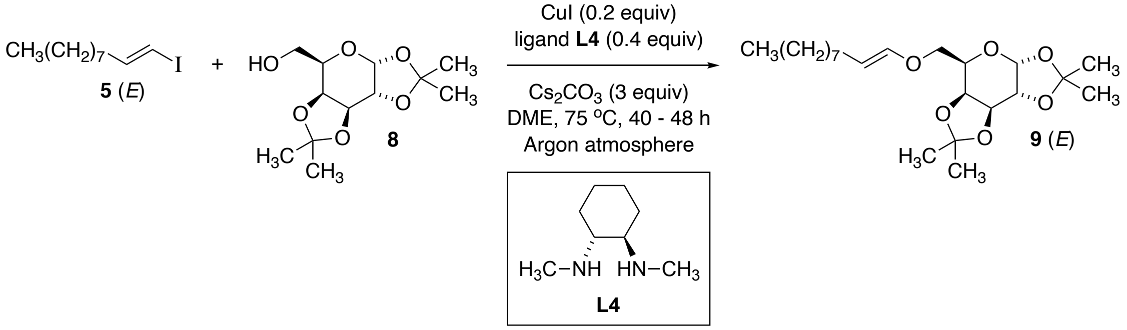

An oven-dried 4 mL vial with a stir bar was charged with 1,2:3,4-di-*O*-isopropylidene-α-D-galactopyranose (**8**, 127 mg, 0.49 mmol, 1 equiv), Cs_2CO_3 (477 mg, 3 equiv), ligand **L4** (30.8 μL, 0.4 equiv), and (*E*)-1-iodo-1-decene (**5**, 130 mg, 0.49 mmol, 1 equiv) under argon. The reaction vial was purged continuously with argon for 5 min before CuI (18.6 mg, 0.2 equiv) was added. Anhydrous 1,2-dimethoxyethane (DME, 0.7 mL, 0.7 M based on vinylic iodide) was added, and the reaction mixture was bubbled with argon for 5 minutes. The reaction vial was quickly closed with a solid cap, sealed with Teflon tape and electrical tape, and heated at an internal temperature at 75 °C. The reaction mixture was stirred for 48 hours, cooled to room temperature, and diluted with diethyl ether. The mixture was filtered through a Celite® pad and rinsed with diethyl ether (100 mL). The filtrate was concentrated by rotary evaporation to give a dark brown oil as the crude product. The crude product mixture was purified by flash column chromatography, using silica gel pre-treated with 2% Et_3N in hexanes (column: 1 inch diameter, up to 6-inch height), eluting with 92 : 8 hexanes/EtOAc, with fractions monitored by thin layer chromatography (TLC) visualized with *p*-anisaldehyde stain, to obtain the (*E*)-vinylic ether **9** as a clear pale-yellow oil (152 mg, 78% yield).

Characterization data for (*E*)-vinylic ether **9**:

^{1}H NMR (400 MHz, $CDCl_3$) δ 6.25 (dt, *J* = 12.6, 1.3 Hz, 1H), 5.54 (d, *J* = 5.0 Hz, 1H), 4.80 (dt, *J* = 12.6, 7.3 Hz, 1H), 4.61 (dd, *J* = 7.9, 2.4 Hz, 1H), 4.32 (dd, *J* = 5.0, 2.4 Hz, 1H), 4.26 (dd, *J* = 7.9, 1.9 Hz, 1H), 4.02 (ddd, *J* = 7.3, 5.5, 1.9 Hz, 1H), 3.81 (qd, *J* = 10.3, 6.3 Hz, 2H), 1.89 (qd, *J* = 7.2, 1.3 Hz, 2H), 1.53 (s, 3H), 1.45 (s, 3H), 1.40 – 1.17 (m, 18H), 0.87 (t, *J* = 6.8 Hz, 3H).

^{13}C NMR (101 MHz, $CDCl_3$) δ 145.9, 109.5, 108.8, 104.8, 96.5, 71.2, 70.8, 70.7, 67.5, 66.4, 32.0, 30.8, 29.6, 29.4, 29.2, 27.8, 26.2, 26.1, 25.1, 24.6, 22.8, 14.3.

HRMS (APCI): *m/z* calcd for $C_{22}H_{39}O_6^+$ $[M+H]^+$ 399.2741, found 399.2739.

$[\alpha]^{22}_D$ -50.4 (c = 0.1, $CHCl_3$)

Scale-up experiment for synthesizing (*E*)-vinylic ether 9 (Table 1, entry 6):

An oven-dried 20 mL vial with a stir bar was charged with 1,2:3,4-di-*O*-isopropylidene-α-D-galactopyranose (**8**, 911 mg, 3.5 mmol, 1 equiv), Cs_2CO_3 (3.42 g, 3 equiv), ligand **L4** (0.22 mL, 0.4 equiv), and (*E*)-1-iodo-1-decene (**5**, 932 mg, 3.5 mmol, 1 equiv) under argon. The reaction vial was purged continuously with argon for 5 min before CuI (133 mg, 0.2 equiv) was added. Anhydrous 1,2-dimethoxyethane (DME, 5 mL, 0.7 M based on vinylic iodide) was added, and the reaction mixture was bubbled with argon for 5 minutes. The reaction vial was quickly closed with a solid cap, sealed with Teflon tape and electrical tape, and heated at an internal temperature of 75 °C. The reaction mixture was stirred for 43 hours, cooled to room temperature, and diluted with diethyl ether (100 mL). The mixture was filtered through a Celite® pad and rinsed with diethyl ether (250 mL). The filtrate was concentrated by rotary evaporation to give a dark brown oil as the crude product. The crude product mixture was purified by flash column chromatography, using silica gel pre-treated with 2% Et_3N in hexanes (column: 1.5-inch diameter, 6-inch height), eluting with 92 : 8 hexanes/EtOAc, with fractions monitored by thin layer chromatography (TLC) visualized with *p*-anisaldehyde stain, to obtain the (*E*)-vinylic ether **9** as a clear pale-yellow oil (1.05 g, 75% yield).

Synthesis of (*E*)-vinylic ether 9 from 1-bromo-1-decene (S6, Table 1, entry 7):

(*E*)-1-bromo-1-decene **S6** was reacted with 1,2:3,4-di-*O*-isopropylidene-α-D-galactopyranose (**8**, 119 mg, 0.46 mmol, 1 equiv), Cs_2CO_3 (446 mg, 3 equiv), ligand **L4** (28.8 µL, 0.4 equiv), CuI (17.4 mg, 0.2 equiv), and DME (0.7 mL, 0.7 M based on vinylic iodide) in the same manner described above for *E*-1-iodo-1-decene (**5**). The crude product was purified by flash column chromatography as described above to obtain the (*E*)-vinylic ether **9** as a clear pale-yellow oil (96.4 mg, 57% yield).

(*E*)-Enyne **10** was isolated from early experiments using ligand **L1**.

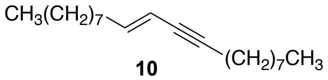

10

Characterization of (*E*)-enyne **10**:[19]

^{1}H NMR (600 MHz, $CDCl_3$) δ 6.04 (dtd, J = 15.7, 7.0, 1.4 Hz, 1H), 5.44 (dp, J = 15.8, 1.9 Hz, 1H), 2.27 (tt, J = 7.3, 1.8 Hz, 2H), 2.06 (qd, J = 7.1, 1.6 Hz, 2H), 1.35 – 1.16 (m, 24H), 0.88 (tt, J = 7.2, 1.6 Hz, 6H).

HRMS (APCI): *m/z* calcd for $C_{20}H_{37}^+$, 277.2890, found 277.2891.

Optimized procedure for the synthesis of 1,2-disubstituted (*Z*)-vinylic ether 12 (Table 2, entry 5):

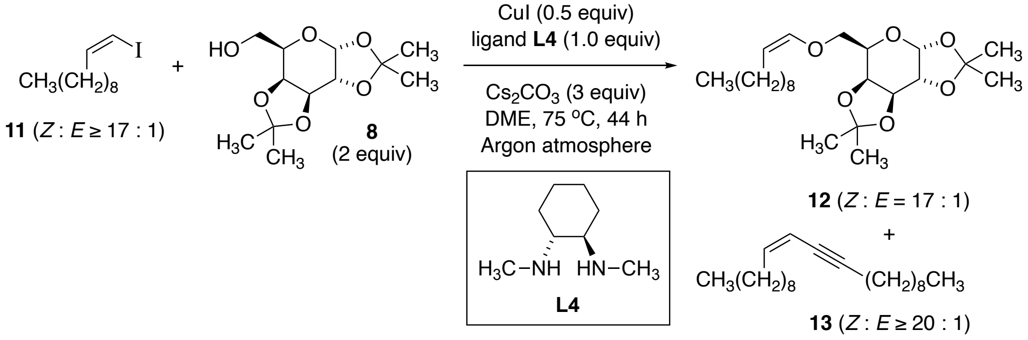

A similar protocol to (*E*)-vinylic ether synthesis was used, with the following stoichiometry: alcohol **8** (187 mg, 0.72 mmol, 2 equiv), (*Z*)-vinylic iodide **11** (20 : 1 *Z* / *E*, 100 mg, 0.36 mmol, 1 equiv), ligand **L4** (57 μL, 0.36 mmol, 1 equiv), CuI (34 mg, 0.18 mmol, 0.5 equiv), Cs_2CO_3 (352 mg, 1.08 mmol, 3 equiv), and anhydrous DME (0.5 mL). The reaction was stirred under argon atmosphere at 75 °C for 44 h. The reaction mixture was cooled to room temperature before dilution with EtOAc, and was filtered through a Celite® pad, using 80 – 100 mL of EtOAc to rinse the reaction vial and filter pad. An 18:1 Z/E ratio of the vinylic ether product was determined from ^{1}H NMR analysis of the crude product mixture. The dark brown crude oil was purified by flash column chromatography with neutral alumina (column: 1-inch diameter, filled up to 3-inch height), eluting with a gradient of 10:1 to 5:1 hexanes/EtOAc (TLC in 10:1 hexanes/EtOAc, visualized with *p*-anisaldehyde) to afford the title compound **12** as a clear pale-yellow oil (97 mg, 66% combined yield of inseparable diastereomers, 17:1 *Z*/*E*). (*Z*)-Enyne **13** was isolated (9.0 mg, 16% yield). The *Z/E* ratio of vinylic ether products were determined by ^{1}H NMR analysis, using alkenyl proton signals:

- 6.25 – 6.20 ppm (*J* = 12 – 14 Hz) for the *E*-isomer
- 6.00 - 5.95 ppm (*J* = 6 – 8 Hz) for the *Z*-isomer **12**.

K_2CO_3-treated deuterated $CDCl_3$ was used for all NMR spectra, both before and after the purification process.

Scale-up experiment for synthesizing (*Z*)-vinylic ether 12 (Table 2, entry 6): To an oven-dried 15 mL pressure tube charged with a stir bar, alcohol **8** (1.56 g, 6 mmol, 2 equiv) was weighed under air, followed by addition of vinylic iodide **11** (25:1 Z/E, 840 mg, 3 mmol, 1 equiv), and ligand **L4** (0.48 mL, 3 mmol, 1 equiv). The reaction vessel was purged continuously with argon. CuI (283 mg, 1.5 mmol, 0.5 equiv) and Cs_2CO_3 (2.9 g, 9 mmol, 3 equiv) were added sequentially under argon atmosphere. Anhydrous DME (4.3 mL) was added to dissolve the reactants, and the reaction mixture was bubbled with argon vigorously for 5 minutes. The tube was quickly closed with a solid cap, sealed with Teflon tape and electrical tape, and heated at an internal temperature of 75 °C. The reaction was stirred over 46 hours, cooled to room temperature, and diluted with EtOAc. The mixture was filtered through a Celite® pad, using several EtOAc washes to rinse the reaction vessel and the Celite pad. The filtrate was concentrated by rotary evaporation to give a thick dark-brown oil. This crude product was purified by flash column chromatography (column: 2-inch diameter, filled to 7-inch height) with 2% triethylamine-treated silica gel, eluted with a gradient of 97:3 to 95:5 hexanes/EtOAc to afford the *Z*-vinylic ether **12** (855 mg, 69% yield, 17:1 Z/E) and *Z*-enyne **13** (67 mg, 15% yield).

Characterization data for (*Z*)-vinylic ether **12**:

^{1}H NMR (800 MHz, CDCl$_3$) (major *Z*-isomer) δ 5.96 (dt, *J* = 6.2, 1.5 Hz, 1H), 5.52 (d, *J* = 5.0 Hz, 1H), 4.61 (dd, *J* = 7.9, 2.4 Hz, 1H), 4.37 (td, *J* = 7.3, 6.2 Hz, 1H), 4.31 (dd, *J* = 5.0, 2.4 Hz, 1H), 4.25 (dd, *J* = 8.0, 1.8 Hz, 1H), 3.98 (td, *J* = 6.3, 1.8 Hz, 1H), 3.92 (dd, *J* = 10.8, 6.2 Hz, 1H), 3.84 (dd, *J* = 10.8, 6.4 Hz, 1H), 2.06 (qd, *J* = 7.3, 1.5 Hz, 2H), 1.53 (s, 3H), 1.45 (s, 3H), 1.34 (s, 3H), 1.33 (s, 3H), 1.32 – 1.22 (m, 14H, overlapping with minor *E*-isomer), 0.88 (t, *J* = 7.1 Hz, 3H).

^{13}C NMR (201 MHz, CDCl$_3$) (major *Z*-isomer) δ 144.9, 109.5, 108.8, 108.1, 96.4, 71.1, 70.8, 70.7, 70.6, 66.8, 32.1, 29.9, 29.8, 29.7, 29.51, 29.47, 26.2, 26.1, 25.1, 24.5, 24.1, 22.8, 14.3.

HRMS (ESI): *m/z* calcd for $C_{23}H_{40}O_6Na^+$ $[M+Na]^+$ 435.2723, found 435.2719.

IR (neat): 2922, 2853, 1663 (weak), 1211, 1070 cm^{-1}.

$[\alpha]^{21}_D$ -48.4 (c = 0.1, CHCl$_3$).

Characterization data for (*Z*)-enyne **13** (*Z* : *E* ≥ 20 : 1):

^{1}H NMR (400 MHz, CDCl$_3$) δ 5.83 (dt, *J* = 10.7, 7.3 Hz, 1H), 5.45 (dt, *J* = 10.8, 1.9 Hz, 1H), 2.36 (td, *J* = 7.1, 2.3 Hz, 2H), 2.33 – 2.26 (m, 2H), 1.60 – 1.52 (m, 2H), 1.48 – 1.37 (m, 4H), 1.31 (qd, *J* = 8.1, 5.0 Hz, 22H), 0.93 – 0.87 (m, 6H).

^{13}C NMR (101 MHz, CDCl$_3$) δ 142.8, 109.4, 94.6, 77.5, 32.08, 32.05, 30.2, 29.75, 29.70, 29.6, 29.52, 29.47, 29.4, 29.3, 29.1, 29.0 (2 overlapping carbons), 22.8 (2 overlapping carbons), 19.7, 14.3 (2 overlapping carbons).

HRMS (APCI): *m/z* calcd for $C_{22}H_{41}^+$ $[M+H]^+$ 305.3203, found 305.3196.

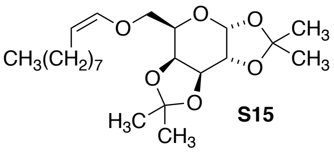

S15

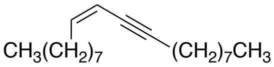

S16

(*Z*)-vinylic ether **S15** (100% *Z*, from **Table 2, entry 7**): The reaction was performed according to the general procedure using 1,2:3,4-di-*O*-isopropylidene-α-D-galactopyranose (**8**, 187 mg, 0.72 mmol, 2 equiv) and (*Z*)-1-iodo-1-decene (**S12**, 100% *Z*, <5% 1-iodododecane; 100 mg, 0.36 mmol, 1 equiv) as substrates. No *E*-isomer was observed in the crude mixture based on ^{1}H NMR analysis. The dark brown crude oil was subjected directly to flash column chromatography with neutral alumina (column: 1-inch diameter, filled up to 3-inch height), eluted with a gradient of 10:1 to 5:1 hexanes/EtOAc (TLC in 10:1 hexanes/EtOAc, visualized with *p*-anisaldehyde) to afford the title compound as a clear pale-yellow oil (82 mg, 57% yield, 100% *Z*, ~95% pure by ^{1}H NMR analysis). A small quantity of (*Z*)-enyne **S16** was isolated (8.0 mg, 16% yield). A second flash column chromatography was performed using silica gel treated with 2% Et$_3$N (column: 0.5-inch diameter, filled up to 7-inch height) and eluted with 97:3 hexanes/EtOAc to afford a fraction (35 mg) of pure **S15**, on which characterization was performed.

Characterization data for compound **S15** (100% *Z*):

^{1}H NMR (600 MHz, $CDCl_3$) (pure *Z*-isomer) δ 5.95 (dt, *J* = 6.2, 1.5 Hz, 1H), 5.52 (d, *J* = 4.9 Hz, 1H), 4.60 (dd, *J* = 7.9, 2.4 Hz, 1H), 4.37 (td, *J* = 7.3, 6.2 Hz, 1H), 4.30 (dd, *J* = 5.0, 2.4 Hz, 1H), 4.25 (dd, *J* = 7.9, 1.9 Hz, 1H), 3.97 (td, *J* = 6.2, 1.8 Hz, 1H), 3.91 (dd, *J* = 10.8, 6.2 Hz, 1H), 3.83 (dd, *J* = 10.8, 6.4 Hz, 1H), 2.06 (qd, *J* = 7.2, 1.5 Hz, 2H), 1.53 (s, 3H), 1.44 (s, 3H), 1.34 – 1.33 (s, 3H), 1.33 – 1.32 (s, 3H), 1.32 – 1.23 (m, 12H), 0.87 (t, *J* = 7.0 Hz, 3H).

^{13}C NMR (151 MHz, $CDCl_3$) (pure *Z*-isomer) δ 144.9, 109.5, 108.8, 108.1, 96.4, 71.0, 70.8, 70.7, 70.6, 66.8, 32.1, 29.9, 29.6, 29.47, 29.46, 26.2, 26.1, 25.1, 24.5, 24.1, 22.8, 14.3.

HRMS (APCI): *m/z* calcd for $C_{22}H_{39}O_6^+$ $[M+H]^+$ 399.2741, found 399.2745.

$[\alpha]^{22}_D$ -81.6 (c = 0.1, $CHCl_3$).

Additional optimization data for (*E*)-vinylic ether synthesis

Ligands evaluated for Cu(I)-catalyzed C-O cross-couplings:

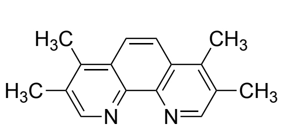

L1

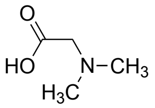

L2

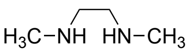

L3

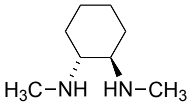

L4

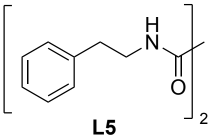

L5

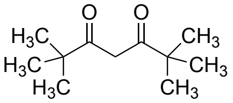

L6

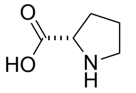

L7

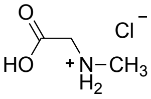

L8

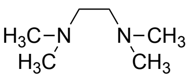

L9

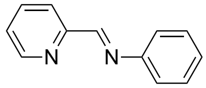

L10

L1: 3,4,7,8-Tetramethyl-1,10-phenanthroline (Me_4Phen)[1]

L2: *N*,*N*-Dimethylglycine (DMG)[20]

L3: *N*,*N'*-Dimethylethylenediamine (DMEDA)[21,22]

L4: (±)-*trans*-*N*,*N'*-Dimethylcyclohexane-1,2-diamine (CyDMEDA)[23]

L5: *N*,*N'*-Bis(2-phenylethyl)ethanediamide (DPEO)[24]

L6: 2,2,6,6-Tetramethyl-3,5-heptanedione[25,26]

L7: L-Proline[27-30]

L8: Sarcosine hydrochloride[31]

L9: *N*,*N*,*N'*,*N'*-Tetramethylethylenediamine (TMEDA)[31]

L10: *trans*-*N*-(2-Pyridylmethylene)aniline[32]

Under basic reaction conditions (with Cs_2CO_3), **L1**, **L3**, **L4**, **L9** and **L10** are neutral ligands, and **L2**, **L5**, **L6**, **L7** and **L8** are anionic ligands.

Table S1. Initial optimization data for synthesizing (*E*)-vinylic ether **9**

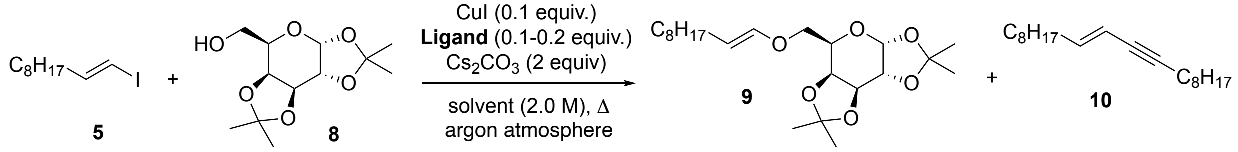

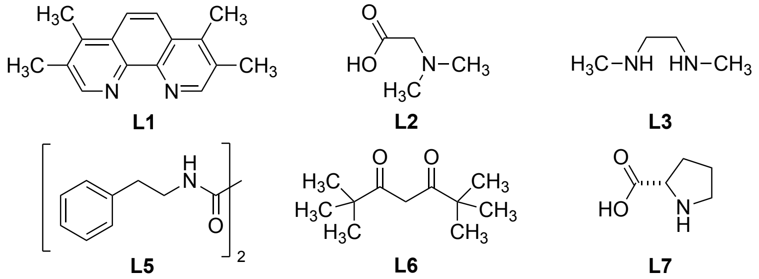

Note	Solvent	Alcohol **8** (equiv)	Ligand (equiv)	temp (°C)	time (h)	% yield vinylic ether **9**	% yield enyne[b] **10**	% of **5** recovered
Initial solvent screening	*o*-xylene	2.0	**L1** (0.2)	125-130	39	31	18	--
	DMF	2.0	**L1** (0.2)	125-130	40	30	18	--
	Tetraglyme	2.0	**L1** (0.2)	125-130	21	35	20	--
Initial ligand screening	Tetraglyme	2.0	**L2** (0.2)	125-130	19	49	10	--
	Tetraglyme	2.0	**L3** (0.2)	125-130	44	35	20	--
	Tetraglyme	2.0	**L5** (0.1)	125-130	44	32	22	--
Optimized temperature & equivalents	Tetraglyme	2.0	**L2** (0.2)	80	17	46	4.9	--
	Tetraglyme	1.0	**L2** (0.2)	80	42	50	< 5	17
	Tetraglyme	1.0	**L2** (0.1)	80	45	62	< 5	29
	Tetraglyme	1.0	**L2** (0.2)	80	45	78[a]	<5	--
Additional solvent screening	*o*-xylene	1.0	**L2** (0.1)	80	45	59	< 5	23
	DMF	1.0	**L2** (0.1)	80	45	56	14	8
Other anionic ligands	Tetraglyme	1.0	**L6** (0.1)	80	40	26	< 5	49
	Tetraglyme	1.0	**L7** (0.1)	80	41	39	< 5	36

[a] With CuI (0.2 equiv) and Cs_2CO_3 (3 equiv). [b] As the formation of each equivalent of enyne consumed two equivalents of vinylic iodide, the isolated yield was determined by dividing the mmol of enyne side-product by the mmol of vinylic iodide reactant, then multiplying it by 2.

$$\%\ \text{yield enyne} = \frac{\text{mmol of enyne}}{\text{mmol of vinylic iodide}} \times 2$$

The initial solvent and ligand screening (**Table S1**) revealed that the anionic ligand **L2** in tetraglyme solvent facilitated the C-O cross-coupling reaction, using a 1 : 1 ratio of CuI : **L2**. Reducing the temperature from 125-130 °C to 80 °C only slightly decreased the yield of (*E*)-vinylic ether **9**, and more substantially decreased the yield of enyne side-product **10**. With anionic ligand **L2** in tetraglyme solvent and increasing catalyst loading to 0.2 equiv CuI and 0.2 equiv **L2** with 3 equiv of Cs_2CO_3, we achieved full conversion of vinylic iodide **5** with equimolar amount of alcohol **8**, resulting in excellent yield of (*E*)-vinylic ether **9**. Anionic ligands **L5**, **L6**, and **L7** gave inferior results, as did *o*-xylene and DMF solvent with **L2**.

Table S2. Additional optimization data for synthesizing (*E*)-vinylic ether **9** with ligand **L4**

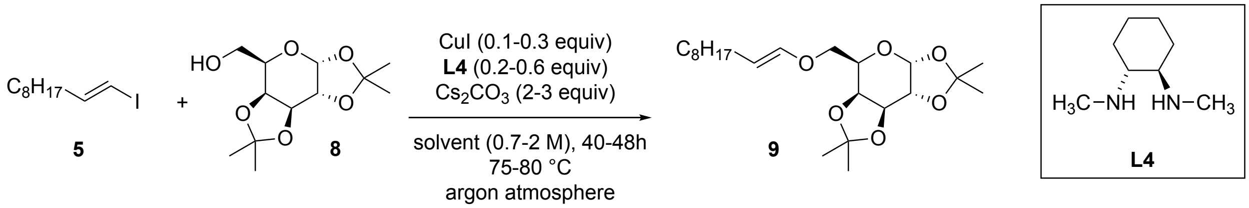

Note	Solvent	Alcohol **8** (equiv)	CuI (equiv)	Ligand **L4** (equiv)	Cs_2CO_3 (equiv)	% yield vinylic ether **9**	% of **5** recovered
Solvent screening	DME	1.0	0.1	0.2	2	62	13
	Diglyme	1.0	0.1	0.2	2	50	9
	Tetraglyme (2.0 M)	1.0	0.1	0.2	2	54	23
Optimized equivalents of each reactant	DME	2.0	0.1	0.2	2	66	10
	DME	2.0	0.3	0.6	2	72	8
	DME	1.0	0.2	0.4	3	78	0

Upon additional screening of polyether solvents, 1,2-dimethoxyethane (DME) gave excellent results for C-O cross-couplings with the neutral ligand **L4**. With neutral ligand **L4** in DME solvent and increasing catalyst loading to 0.2 equiv CuI and 0.4 equiv **L4** with 3 equiv of Cs_2CO_3, we achieved full conversion of vinylic iodide **5** with equimolar amount of alcohol **8**, resulting in excellent yield of (*E*)-vinylic ether **9**.

Additional optimization data for (*Z*)-vinylic ether synthesis

Table S3. Additional ligand screening for synthesizing (*Z*)-vinylic ether **12**

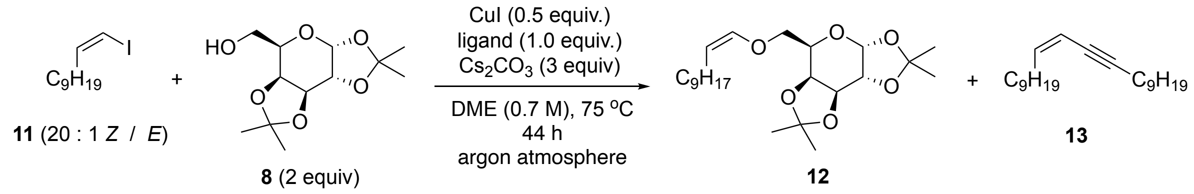

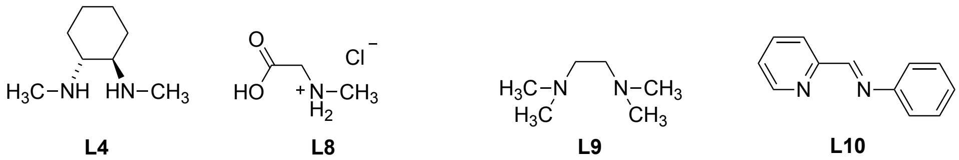

Note	Ligand	% yield vinylic ether **12** (*Z* : *E* ratio)	% yield enyne **13**	% of **11** recovered
Table 2, entry 5	**L4**	66% (17 : 1)	16%	0%
anionic with 2° amine	**L8**	0%	75%	20%
neutral 3° diamine	**L9**	0%[a]	0%	0%
neutral tuneable ligand	**L10**	0%	trace	0%

[a] Vinylic iodide **11** was completely consumed. ^{1}H NMR analysis of the crude reaction mixture showed no alkene resonances.

The anionic ligand **L8** was also incompatible with the (*Z*)-selective cross-coupling reaction. The additional basic carboxylate moiety of both amino acid ligands **L2** and **L8** accelerates the enyne-forming side reaction, outcompeting C-O bond formation. The increased steric bulk of the neutral diamine ligand **L9** adversely affected the outcome of the copper-catalyzed reaction. Iminopyridine ligand **L10**, were previously developed for C-O and C-N cross-couplings with aryl halides, providing different electronic environments for the oxidative addition step (electron-rich pyridine nitrogen) and reductive elimination step (electron-poor imine nitrogen). However, the only product observed was a trace of enyne **13**, arising from elimination of (*Z*)-vinylic iodide **11**, which is a pathway that was not available for the substrates described in that literature.[32]

Table S4. Additional optimization data for synthesizing (*Z*)-vinylic ether **12** with ligand **L3**

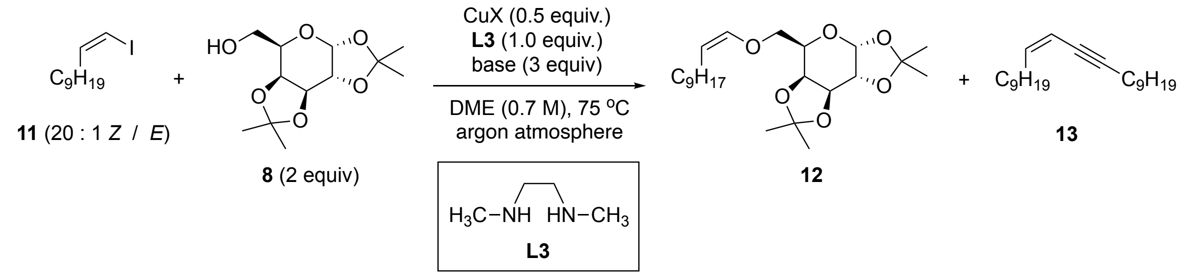

Note	CuX source	Base	Time (h)	% yield vinylic ether **12** (*Z* : *E* ratio)	% yield enyne **13**	% of **11** recovered
Base screening	CuI	K_2CO_3	66	39% (14 : 1)	32%	trace
	CuI	$CsHCO_3$	66	24% (8 : 1)	18%	13%
	CuI	Cs_2CO_3	44	46% (12 : 1)	24%	0%
Cu(I) sources	CuBr	Cs_2CO_3	44	45% (8 : 1)	36%	0%
	$Cu(MeCN)_4(BF_4)$	Cs_2CO_3	44	37% (10 : 1)	27%	0%

Reaction optimization was initially performed with **L3** after its initial success with the (*Z*)-selective C-O cross-coupling reaction. To suppress formation of enyne **13**, we screened several weaker bases such as K_2CO_3 and $CsHCO_3$. However, the lower basicity also retarded the rate of alcohol deprotonation, resulting in inferior yields of vinylic ether **12** and incomplete conversion of vinylic iodide **11**. Cs_2CO_3 was found to be the most effective base.

We also tested other Cu(I) sources with **L3** as alternatives to CuI. CuBr provided comparable yield of vinylic ether **12**, but the *Z* : *E* ratio was significantly reduced when compared with CuI. The halide-free catalyst $Cu(MeCN)_4(BF_4)$[33] gave lower yield of vinylic ether **12** despite the complete consumption of iodide **11**. ^1H NMR analysis of the crude mixture revealed that a portion of the vinylic iodide **11** was converted to the terminal alkyne product (1-undecyne) via base-promoted elimination (ca. 10%). Thus, we concluded that CuI and Cs_2CO_3 are necessary to catalyze and promote the desired Ullmann-type cross-coupling reaction.

Table S5. Optimizing conditions for synthesizing (*Z*)-vinylic ether **12** with ligand **L4**

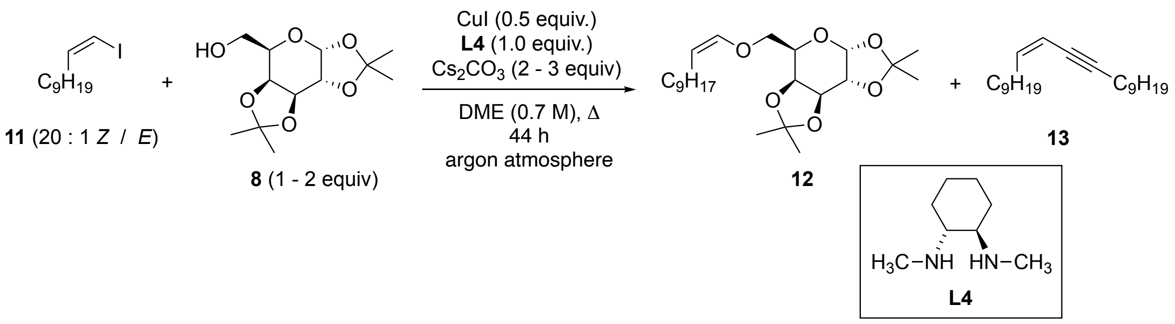

Note	Alcohol **8** (equiv)	Cs_2CO_3 (equiv)	temp (°C)	% yield vinylic ether **12** (*Z* : *E* ratio)	% yield enyne **13**	% of **11** recovered
Table 2, entry 4	1.2 equiv	3 equiv	75 °C	63% (17 : 1)	16%	0%
	1.2 equiv	2 equiv	75 °C	53% (8 : 1)	19%	0%
Table 2, entry 5	2 equiv	3 equiv	75 °C	66% (17 : 1)	16%	0%
	2 equiv	3 equiv	85 °C	57% (20 : 1)	22%	0%

Using only 1.2 equiv of alcohol **8** relative to the (*Z*)-vinylic iodide **11** provided comparable yield and retention of configuration to that of the optimized condition ***only*** when 3 equiv of Cs_2CO_3 were utilized, suggesting that the alcohol deprotonation step is critically important for the catalytic cycle. Utilizing 2 equiv of alcohol **8** for our optimized procedure offered more generality as we subsequently extended substrate scope. The success of the C-O cross-coupling reaction with (*Z*)-vinylic iodide **11** was highly sensitive to temperature: a slight increase from 75 to 85 °C reduced the yield of (*Z*)-vinylic ether **12** and increased the yield of (*Z*)-enyne side product **13**.

Scope of (*E*)- and (*Z*)-selective cross-coupling products from primary alcohols (Figure 2a)

Representative procedure for the synthesis of 1,2-disubstituted (*E*)-vinylic ethers (Conditions A, Figure 2): An oven-dried 4 mL vial charged with a stir bar was added alcohol (1 equiv), Cs_2CO_3 (3 equiv), **L4** (0.4 equiv), and *E*-vinylic iodide **5** (1 equiv) under argon. The reaction vial was purged continuously with argon for 5 min before CuI (0.2 equiv) was added. Anhydrous DME (0.5 mL, 0.7 M based on vinylic iodide) was added, and the reaction mixture was bubbled with argon for 5 minutes. The reaction vial was quickly closed with a solid cap, sealed with Teflon tape and electrical tape, and placed on the heat with internal temperature at 75 °C. The reaction was stirred over 40 - 48 hours, cooled to room temperature, and diluted with diethyl ether. The mixture was filtered through a Celite® pad and rinsed with diethyl ether (100 mL). The filtrate was concentrated by rotary evaporator, and the crude mixture was subjected to flash column chromatography with 2% Et_3N-treated silica gel using hexanes/EtOAc as eluent to purify vinylic ethers.

Representative procedure for the synthesis of 1,2-disubstituted (*Z*)-vinylic ethers (Conditions B, Figure 2): Similar protocol to *E*-vinylic ether synthesis was used with the following stoichiometry: alcohol (2 equiv), *Z*-vinylic iodide **11** (1 equiv), **L4** (1 equiv), CuI (0.5 equiv.), Cs_2CO_3 (3 equiv), and anhydrous DME (0.5 mL). The work-up was performed using EtOAc as diluting solvent. The crude mixture was subjected to flash column chromatography with either neutral alumina or 2% Et_3N-treated silica gel. The *Z*/*E* ratio of *Z*-vinylic ether product was determined by ^1H NMR analysis using alkenyl proton signals at 6.00 – 5.95 ppm (J = 6 – 8 Hz) for *Z*-isomer and 6.25 – 6.20 ppm (J = 12 – 14 Hz) for *E*-isomer, using K_2CO_3-treated deuterated chloroform before and after the purification process. Yields and isomer ratios of vinylic ether products are reported after chromatography, as combined yields for inseparable *Z*- and *E*-isomers.

(*E*)-vinylic ether 7, from 2-heptyn-1-ol and (*E*)-1-iodo-1-decene (**5**):

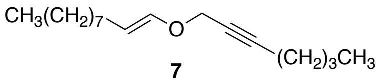

7

Using conditions A, with 2-heptyn-1-ol (46.4 mg, 0.41 mmol, 1 equiv) and **5** (110 mg, 0.41 mmol, 1 equiv) as substrates. The dark brown crude oil was subjected directly to flash column chromatography with silica gel pre-treated with 2% Et_3N in hexanes (column: 1-inch diameter, up to 6.7-inch height), eluting with 100% hexanes (visualized with *p*-anisaldehyde) to afford the title compound **7** as a clear pale-yellow oil (84.0 mg, 81% yield).

Characterization data for (*E*)-vinylic ether **7**:[1]

^{1}H NMR (400 MHz, $CDCl_3$) δ 6.21 (dt, J = 12.5, 1.3 Hz, 1H), 4.87 (dt, J = 12.6, 7.4 Hz, 1H), 4.30 (t, J = 2.1 Hz, 2H), 2.22 (tt, J = 7.1, 2.2 Hz, 2H), 1.91 (qd, J = 7.2, 1.3 Hz, 2H), 1.54 – 1.37 (m, 4H), 1.37 – 1.19 (m, 12H), 0.95 – 0.83 (m, 6H).

^{13}C NMR (101 MHz, $CDCl_3$) δ 144.7, 106.2, 87.9, 75.2, 57.5, 32.0, 30.70, 30.66, 29.6, 29.5, 29.2, 27.8, 22.8, 22.0, 18.6, 14.3, 13.7.

HRMS (APCI): *m/z* calcd for $C_{17}H_{31}O^+$ 251.2369, found 251.2370.

(*Z*)-vinylic ether 14, from 2-heptyn-1-ol and (*Z*)-1-iodo-1-undecene (**11**):

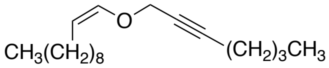

14

Using conditions B, with 2-heptyn-1-ol (81 mg, 0.72 mmol, 2 equiv) and vinylic iodide **11** (50:1 Z/E, 100 mg, 0.36 mmol, 1 equiv) as substrates. ^{1}H NMR analysis of the crude product mixture showed >20:1 Z/E ratio favoring vinylic ether **14**. The dark brown crude oil was subjected to flash column chromatography with silica gel treated with 2% Et_3N (column: 1-inch diameter, filled up to 7-inch height), eluted with 100% hexanes in 4-6 mL fractions (analyzed by TLC using 100% hexanes to elute, visualized with *p*-anisaldehyde stain), then concentrated by rotary evaporation with bath temperature at 30 °C, to afford the title compound **14** as a clear oil (70 mg, 74% combined yield of inseparable diastereomers, >20:1 Z/E). The (*Z*)-enyne **13** was also isolated (6 mg, 11% yield).

Characterization data for (*Z*)-vinylic ether **14**:

^{1}H NMR (400 MHz, CDCl$_3$) δ 6.03 (dt, *J* = 6.2, 1.5 Hz, 1H), 4.45 (td, *J* = 7.3, 6.2 Hz, 1H), 4.33 (t, *J* = 2.2 Hz, 2H), 2.22 (tt, *J* = 7.1, 2.2 Hz, 2H), 2.07 (qd, *J* = 7.3, 1.5 Hz, 2H), 1.53 – 1.44 (m, 2H), 1.44 – 1.36 (m, 2H), 1.35 – 1.23 (m, 14H), 0.89 (dt, *J* = 11.5, 7.2 Hz, 6H).

^{13}C NMR (101 MHz, CDCl$_3$) δ 143.3, 109.0, 87.7, 75.5, 59.6, 32.1, 30.7, 29.9, 29.8, 29.7, 29.5, 29.4, 24.1, 22.8, 22.1, 18.6, 14.3, 13.7.

HRMS (APCI): *m/z* calcd for $C_{18}H_{33}O^+$ $[M+H]^+$ 265.2526, found 265.2520.

IR: 3030 (weak), 2922, 2853, 1665, 1230 (weak), 1089 cm^{-1}.

(*E*)-vinylic ether 15, from cinnamyl alcohol and (*E*)-1-iodo-1-decene (**5**):

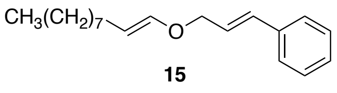

15

Using conditions A, with cinnamyl alcohol (65.5 mg, 0.49 mmol, 1 equiv) and vinylic iodide **5** (130 mg, 0.49 mmol, 1 equiv) as substrates. The reaction mixture was stirred at 70 °C for 18 h, to minimize thermal Claisen rearrangement of the allylic vinylic ether product **15** as it formed. However, the crude ^{1}H NMR spectrum showed about 9% of two aldehyde diastereomers, in a 5 : 4 ratio, with doublets at 9.58 ppm and 9.44 ppm, respectively. The dark brown crude oil was subjected directly to flash column chromatography with silica gel pre-treated with 2% Et_3N in hexanes (column: 1-inch diameter, up to 6-inch height), eluting with 100% hexanes (visualized with *p*-anisaldehyde) to afford the title compound **15** as a clear pale-yellow oil (85.9 mg, 65% yield).

Characterization data for (*E*)-vinylic ether **15**:

^{1}H NMR (400 MHz, $CDCl_3$) δ 7.44 – 7.22 (m, 5H), 6.64 (dt, *J* = 15.9, 1.6 Hz, 1H), 6.31 (dt, *J* = 15.9, 5.9 Hz, 1H), 6.27 (dt, *J* = 12.4, 1.1 Hz, 1H), 4.87 (dt, *J* = 12.5, 7.4 Hz, 1H), 4.35 (dd, *J* = 5.9, 1.5 Hz, 2H), 1.92 (qd, *J* = 7.2, 1.3 Hz, 2H), 1.40 – 1.23 (m, 12H), 0.88 (t, *J* = 6.7 Hz, 3H).

^{13}C NMR (101 MHz, $CDCl_3$) δ 145.6, 136. 7, 132.8, 128.7 (2C), 127.9, 126.7 (2C), 125.1, 105.5, 69.9, 32.0, 30.8, 29.6, 29.5, 29.2, 27.9, 22.8, 14.3.

HRMS (APCI): *m/z* calcd for $C_{19}H_{29}O^+$ $[M+H]^+$ 273.2213, found 273.2212.

(*Z*)-vinylic ether 16, from cinnamyl alcohol and (*Z*)-1-iodo-1-undecene (**11**):

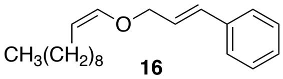

16

Using conditions B, with cinnamyl alcohol (97 mg, 0.72 mmol, 2 equiv.) and vinylic iodide **11** (50:1 Z/E, 100 mg, 0.36 mmol, 1 equiv) as substrates. *The reaction was conducted for only 18 h followed by the standard workup procedure, to avoid thermal Claisen rearrangement of the allylic vinylic ether product **16** as it formed. ^{1}H NMR analysis of the crude product mixture showed >20:1 Z/E ratio favoring vinylic ether **16**, with no trace of Claisen rearrangement product detected in the crude mixture (absence of aldehyde resonances at 9.56 and 9.44 ppm). The dark brown crude oil was subjected to flash column chromatography with silica gel treated with 2% Et_3N (column: 1-inch diameter, filled up to 7-inch height), eluted with 100% hexanes in 4-6 mL fractions (TLC in 100% hexanes, visualized with UV light and *p*-anisaldehyde stain) to afford the title compound **16** as a clear pale oil (63 mg, 61% combined yield of inseparable diastereomers, >20:1 Z/E). The (*Z*)-enyne **13** was also isolated (6 mg, 11% yield).

Characterization data for (*Z*)-vinylic ether **14** (>20 : 1 *Z* / *E*):

^{1}H NMR (400 MHz, $CDCl_3$) δ 7.43 – 7.35 (m, 2H), 7.36 – 7.27 (m, 2H), 7.27 – 7.20 (m, 1H), 6.62 (dt, *J* = 16.0, 1.6 Hz, 1H), 6.28 (dt, *J* = 15.9, 5.8 Hz, 1H), 6.00 (dt, *J* = 6.3, 1.5 Hz, 1H), 4.44 – 4.34 (m, 3H), 2.11 (qd, *J* = 7.2, 1.5 Hz, 2H), 1.38 – 1.22 (m, 14H), 0.88 (t, *J* = 6.7 Hz, 3H).

^{13}C NMR (101 MHz, $CDCl_3$) δ 144.3, 136.7, 132.7, 128.7, 128.0, 126.7, 125.6, 107.9, 72.4, 32.1, 30.0, 29.8, 29.7, 29.52, 29.47, 24.2, 22.8, 14.3.

HRMS (APCI): *m/z* calcd for $C_{20}H_{31}O^+$ $[M+H]^+$ 287.2369, found 287.2362.

IR: 3028 (weak), 2921, 2852, 1663, 1231 (weak), 1078 cm^{-1}.

R_f (2% Et_3N in hexanes) = 0.12.

(*E*)-vinylic ether 17, from trifluoroethanol and (*E*)-1-iodo-1-decene (**5**):

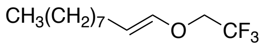

17

Using conditions A, with 2,2,2-trifluoroethanol (48.9 mg, 0.49 mmol, 1 equiv) and vinylic iodide **5** (130 mg, 0.49 mmol, 1 equiv) as substrates. The dark brown crude oil was subjected directly to flash column chromatography with silica gel pre-treated with 2% Et_3N in hexanes (column: 1-inch diameter, up to 6-inch height), eluting with 98:2 hexanes/EtOAc (visualized with *p*-anisaldehyde) to afford the title compound **17** as a clear pale-yellow oil (64.0 mg, 55% yield).

Characterization data for (*E*)-vinylic ether **17**:

^{1}H NMR (400 MHz, $CDCl_3$) δ 6.25 – 6.18 (m, 1H), 4.90 (dt, *J* = 12.5, 7.4 Hz, 1H), 3.99 (q, $^3J_{HF}$ = 8.3 Hz, 2H), 1.91 (qd, *J* = 7.3, 1.3 Hz, 2H), 1.39 – 1.19 (m, 12H), 0.92 – 0.84 (m, 3H).

^{13}C NMR (101 MHz, $CDCl_3$) δ 144.8, 123.6 (q, $^iJ_{CF}$ 278 Hz), 107.5, 66.6 (q, $^2J_{CF}$ = 35 Hz), 32.0, 30.4, 29.5, 29.4, 29.1, 27.4, 22.8, 14.3.

^{19}F NMR (376 MHz, $CDCl_3$) δ -74.22 (t, J = 8.3 Hz).

HRMS (APCI): *m/z* calcd for $C_{12}H_{21}F_3O^+$ $[M+H]^+$ 239.1617, found 239.1614.

(*Z*)-vinylic ether 18, from trifluoroethanol and (*Z*)-1-iodo-1-undecene (**11**):

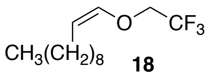

18

Using conditions B, with 2,2,2-trifluoroethanol (72 mg, 0.72 mmol, 2 equiv) and vinylic iodide **11** (50:1 Z/E, 100 mg, 0.36 mmol, 1 equiv) as substrates. ^{1}H NMR analysis of the crude product mixture showed >20:1 Z/E ratio favoring vinylic ether **18**. The dark brown crude oil was subjected to flash column chromatography with silica gel treated with 2% Et_3N (column: 1-inch diameter, filled up to 7-inch height), eluted with 100% hexanes in 4-5 mL fractions (TLC in 100% hexanes, visualized with *phosphomolybdic acid stain*) to afford the title compound **18** as a clear pale-yellow oil (32 mg, 39% yield, >20:1 Z/E). *The compound is volatile under high-pressure vacuum, so solvent removal was only conducted using the rotary evaporator. The (Z)-enyne **13** was also isolated (6 mg, 11% yield).

Characterization data for (*Z*)-vinylic ether **18** (>20:1 Z/E):

^{1}H NMR (400 MHz, $CDCl_3$) δ 5.93 (d, *J* = 6.1 Hz, 1H), 4.52 (td, *J* = 7.4, 6.1 Hz, 1H), 4.03 (q, $^3J_{HF}$ = 8.5 Hz, 2H), 2.09 (qd, *J* = 7.2, 1.5 Hz, 2H), 1.45 – 1.14 (m, 14H), 0.88 (t, *J* = 6.7 Hz, 3H).

^{13}C NMR (101 MHz, $CDCl_3$) δ 144.0, 123.6 (q, $^1J_{CF}$ = 280 Hz), 110.6, 68.9 (q, $^2J_{CF}$ = 35 Hz), 32.1, 29.7, 29.60, 29.58, 29.5, 29.3, 23.8, 22.8, 14.3.

^{19}F NMR (376 MHz, $CDCl_3$) δ -74.67 (t, $^3J_{HF}$ = 8.5 Hz).

HRMS (APCI): *m/z* calcd for $C_{13}H_{24}OF_3^+$ $[M+H]^+$ 253.1774, found 253.1764.

IR: 2924, 2855, 1669, 1280, 1160, 1133 cm^{-1}.

(*E*)-vinylic ether 19, from methyl 2,3-*O*-(1-methylethylidene)-D-ribofuranoside and (*E*)-1-iodo-1-decene (**5**):

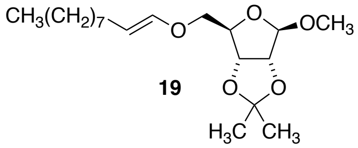

19

Using conditions A, with methyl 2,3-*O*-(1-methylethylidene)-β-D-ribofuranoside (99.7 mg, 0.49 mmol, 1 equiv; synthesized according to literature from D-ribose)[34] and vinylic iodide **5** (130 mg, 0.49 mmol, 1 equiv) as substrates. The dark brown crude oil was subjected directly to flash column chromatography with silica gel pre-treated with 2% Et_3N in hexanes (column: 1-inch diameter, up to 6-inch height), eluting with 99:1 hexanes/EtOAc (visualized with *p*-anisaldehyde) to afford the title compound **19** as a clear pale-yellow oil (129.5 mg, 77%).

Characterization data for (*E*)-vinylic ether **19**:

^{1}H NMR (400 MHz, $CDCl_3$) δ 6.23 (dt, *J* = 12.5, 1.2 Hz, 1H), 4.97 (s, 1H), 4.77 (dt, *J* = 12.6, 7.3 Hz, 1H), 4.67 (dd, *J* = 5.9, 1.0 Hz, 1H), 4.58 (d, *J* = 5.9 Hz, 1H), 4.37 (ddd, *J* = 7.7, 6.4, 1.0 Hz, 1H), 3.71 – 3.56 (m, 2H), 3.32 (s, 3H), 1.89 (qd, *J* = 7.1, 1.3 Hz, 2H), 1.48 (s, 3H), 1.31 (s, 3H), 1.30 – 1.20 (m, 12H), 0.88 (t, *J* = 7.0 Hz, 3H).

^{13}C NMR (101 MHz, $CDCl_3$) δ 145.6, 112.6, 109.4, 105.0, 85.2, 84.7, 82.1, 69.5, 55.0, 32.0, 30.8, 29.6, 29.5, 29.2, 27.8, 26.6, 25.1, 22.8, 14.3.

HRMS (APCI): *m/z* calcd for $C_{19}H_{35}O_5^+$ $[M+H]^+$ 343.2479, found 343.2473.

$[\alpha]^{23}_D$ -31.4 (c = 0.1, $CHCl_3$).

(*Z*)-vinylic ether 20, from methyl 2,3-*O*-(1-methylethylidene)-D-ribofuranoside and (*Z*)-1-iodo-1-undecene (**11**):

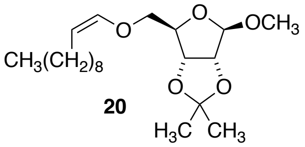

20

Using conditions B, with methyl 2,3-*O*-(1-methylethylidene)-β-D-ribofuranoside[34] (147 mg, 0.72 mmol, 2 equiv) and vinylic iodide **11** (50:1 Z/E, 100 mg, 0.36 mmol, 1 equiv) as substrates. ^{1}H NMR analysis of the crude product mixture showed 17:1 Z/E ratio favoring vinylic ether **20**. The dark brown crude oil was subjected directly to flash column chromatography with neutral alumina (column: 1-inch diameter, filled up to 3-inch height), eluted with a gradient of 10:1 to 5:1 hexanes/ethyl acetate (TLC in 10:1 hexanes/ethyl acetate, visualized with *p*-anisaldehyde) to afford the title compound **20** as a clear pale-yellow oil (73 mg, 57% combined yield of inseparable diastereomers, 17:1 Z/E, ~95% pure by ^{1}H NMR analysis). The (Z)-enyne **13** was also isolated (16 mg, 29% yield). A second column chromatography was performed using silica gel treated with 2% Et_3N (column: 1-inch diameter, filled up to 7-inch height) and eluted with 97:3 hexanes/EtOAc to afford a fraction of pure product **20** (54 mg, 20:1 Z/E), on which characterization was performed.

Characterization data for (*Z*)-vinylic ether **20** (20:1 Z/E):

^{1}H NMR (800 MHz, $CDCl_3$) δ 5.91 (dt, *J* = 6.2, 1.5 Hz, 1H), 4.96 (s, 1H), 4.70 (dd, *J* = 6.0, 1.0 Hz, 1H), 4.59 (d, *J* = 6.0 Hz, 1H), 4.39 (td, *J* = 7.3, 6.2 Hz, 1H), 4.35 (ddd, *J* = 8.3, 6.3, 1.0 Hz, 1H), 3.72 (dd, *J* = 10.5, 6.3 Hz, 1H), 3.70 (dd, *J* = 10.5, 8.4 Hz, 1H), 3.32 (s, 3H), 2.07 (qt, *J* = 7.2, 1.2 Hz, 2H), 1.49 (s, 3H), 1.34 – 1.24 (m, 17H, overlapping with *E*-isomer), 0.88 (t, *J* = 7.1 Hz, 3H).

^{13}C NMR (201 MHz, $CDCl_3$) δ 144.4, 112.6, 109.5, 108.6, 85.3, 84.9, 82.0, 72.5, 55.0, 32.1, 29.9, 29.8, 29.7, 29.5, 29.4, 26.6, 25.1, 24.1, 22.8, 14.3.

HRMS (APCI): *m/z* calcd for $C_{20}H_{37}O_5^+$ $[M+H]^+$ 357.2636, found 357.2628.

IR: 2923, 2853, 1664, 1210 (weak), 1096, 1061 cm^{-1}.

$[\alpha]^{23}_{D}$ -51.0 (c = 0.1, $CHCl_3$).

(*E*)-vinylic ether 21, from (*S*)-1,2-*O*-isopropylideneglycerol and (*E*)-1-iodo-1-decene (**5**):

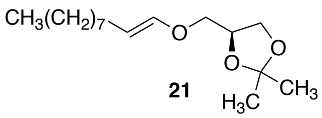

21

Using conditions A, with (*S*)-1,2-*O*-Isopropylideneglycerol (99.7 mg, 0.49 mmol, 1 equiv) and vinylic iodide **5** (130 mg, 0.49 mmol, 1 equiv) as substrates. The dark brown crude oil was subjected directly to flash column chromatography with silica gel pre-treated with 2% Et_3N in hexanes (column: 1-inch diameter, up to 6-inch height), eluting with 98:2 hexanes/EtOAc (visualized with *p*-anisaldehyde) to afford the title compound **21** as a clear pale-yellow oil (98.9 mg, 84%).

Characterization data for (*E*)-vinylic ether **21**:

^{1}H NMR (400 MHz, $CDCl_3$) δ 6.24 (dt, *J* = 12.7, 1.3 Hz, 1H), 4.77 (dt, *J* = 12.6, 7.4 Hz, 1H), 4.32 (apparent quintet, *J* = 6.0 Hz, 1H), 4.08 (dd, *J* = 8.4, 6.4 Hz, 1H), 3.77 (dd, *J* = 8.4, 6.2 Hz, 1H), 3.71 (dd, *J* = 10.0, 5.7 Hz, 1H), 3.63 (dd, *J* = 10.1, 5.6 Hz, 1H), 1.89 (qd, *J* = 7.3, 1.4 Hz, 2H), 1.43 (s, 3H), 1.37 (s, 3H), 1.32 – 1.22 (m, 12H), 0.87 (t, *J* = 6.6 Hz, 3H).

^{13}C NMR (101 MHz, $CDCl_3$) δ 145.9, 109.8, 104.9, 74.2, 69.7, 66.8, 32.0, 30.8, 29.6, 29.4, 29.2, 27.8, 26.9, 25.5, 22.8, 14.3.

HRMS (APCI): *m/z* calcd for $C_{16}H_{31}O_3^+$ $[M+H]^+$ 271.2268, found 271.2261.

$[\alpha]^{23}_{D}$ 5.80 (c = 0.1, $CHCl_3$).

(*Z*)-vinylic ether 22, from (*S*)-1,2-*O*-isopropylideneglycerol and (*Z*)-1-iodo-1-undecene (**11**):

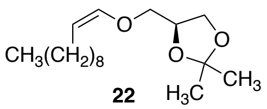

22

Using conditions B, with (*S*)-(2,2-dimethyl-1,3-dioxolan-4-yl)methanol (95 mg, 0.72 mmol, 2 equiv) and vinylic iodide **11** (50:1 Z/E) (100 mg, 0.36 mmol, 1 equiv) as substrates. ^{1}H NMR analysis of the crude product mixture showed >20:1 Z/E ratio favoring vinylic ether **22**. The dark brown crude oil was subjected to flash column chromatography with neutral alumina (column: 1-inch diameter, filled up to 3-inch height), eluted with a gradient of 10:1 to 5:1 hexanes/EtOAc (TLC in 10:1 hexanes/ethyl acetate, visualized with *p*-anisaldehyde) to afford the title compound **22** as a clear pale-yellow oil (61 mg, 60% combined yield of inseparable diastereomers, 20:1 Z/E, ~95% pure by ^{1}H NMR analysis). The (Z)-enyne **13** was also isolated (7 mg, 13% yield). A second column chromatography was performed using silica gel treated with 2% Et_3N (column: 1-inch diameter, filled up to 7-inch height) and eluted with 95:3:2 hexanes/EtOAc/Et_3N to afford a fraction of pure product **22** (42.5 mg, 20:1 Z/E), on which characterization was performed.

Characterization data for (*Z*)-vinylic ether **22** (20:1 Z/E):

^{1}H NMR (800 MHz, $CDCl_3$) δ 5.94 (dt, *J* = 6.2, 1.5 Hz, 1H), 4.36 (td, *J* = 7.3, 6.2 Hz, 1H), 4.27 (qd, *J* = 6.2, 4.8 Hz, 1H), 4.07 (dd, *J* = 8.4, 6.3 Hz, 1H), 3.83 (dd, *J* = 8.4, 6.2 Hz, 1H), 3.80 (dd, *J* = 10.7, 4.8 Hz, 1H), 3.72 (dd, *J* = 10.7, 6.0 Hz, 1H), 2.05 (qdd, *J* = 7.2, 2.4, 1.5 Hz, 2H), 1.42 (d, *J* = 0.8 Hz, 3H), 1.37 (d, *J* = 0.8 Hz, 3H), 1.34 – 1.22 (m, 14H, overlapping with minor diastereomer), 0.88 (t, *J* = 7.1 Hz, 3H).

^{13}C NMR (201 MHz, $CDCl_3$) δ 144.9, 109.7, 108.0, 74.6, 72.4, 66.7, 32.1, 29.9, 29.8, 29.7, 29.5, 29.4, 26.8, 25.6, 24.1, 22.8, 14.3.

HRMS (APCI): *m/z* calcd for $C_{17}H_{33}O_3^+$ $[M+H]^+$ 285.2424, found 285.2422.

IR: 2922, 2853, 1664, 1215, 1103, 1057 cm^{-1}.

$[\alpha]^{21}_D$ +25.0 (c = 0.1, $CHCl_3$).

(*E*)-vinylic ether 23, from 3-methyl-3-oxetanemethanol and (*E*)-1-iodo-1-decene (**5**):

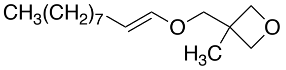

23

Using conditions A, with 3-methyl-3-oxetanemethanol (49.9 mg, 0.49 mmol, 1 equiv) and vinylic iodide **5** (130 mg, 0.49 mmol, 1 equiv) as substrates. The dark brown crude oil was subjected directly to flash column chromatography with silica gel pre-treated with 2% Et_3N in hexanes (column: 1-inch diameter, up to 6-inch height), eluting with 95:5 hexanes/EtOAc (visualized with *p*-anisaldehyde) to afford the title compound **23** as a clear pale-yellow oil (81.6 mg, 70%).

Characterization data for (*E*)-vinylic ether **23**:

^{1}H NMR (400 MHz, $CDCl_3$) δ 6.30 (dt, *J* = 12.8, 1.3 Hz, 1H), 4.79 (dt, *J* = 12.7, 7.3 Hz, 1H), 4.53 (d, *J* = 5.8 Hz, 2H), 4.38 (d, *J* = 5.8 Hz, 2H), 3.69 (s, 2H), 1.91 (qd, *J* = 7.2, 1.3 Hz, 2H), 1.33 (s, 3H), 1.32 – 1.22 (m, 12H), 0.88 (t, *J* = 6.5 Hz, 3H).

^{13}C NMR (101 MHz, $CDCl_3$) δ 146.3, 104.5, 80.0 (2C), 73.8, 39.7, 32.0, 30.9, 29.6, 29.5, 29.2, 27.9, 22.8, 21.4, 14.3.

HRMS (APCI): *m/z* calcd for $C_{15}H_{29}O_2^+$ $[M+H]^+$ 241.2162, found 241.2156.

(*Z*)-vinylic ether 24, from 3-methyl-3-oxetanemethanol and (*Z*)-1-iodo-1-undecene (**11**):

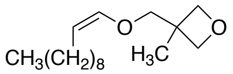

24

Using conditions B, with 3-methyl-3-oxetanemethanol (73 mg, 0.72 mmol, 2 equiv) and vinylic iodide **11** (20:1 Z/E, 100 mg, 0.36 mmol, 1 equiv) as substrates. ^{1}H NMR analysis of the crude product mixture showed 17:1 Z/E ratio favoring vinylic ether **24**, as well as <2% recovered vinylic iodide **11**. The dark brown crude oil was subjected to flash column chromatography with silica gel treated with 2% Et_3N (column: 1-inch diameter, filled up to 7-inch height), eluted with a mixture of 90:8:2 hexanes/EtOAc/Et_3N (TLC in 10:1 hexanes/ethyl acetate, visualized with *p*-anisaldehyde) to afford the title compound **24** as a clear pale-yellow oil (43 mg, 50% combined yield, 12:1 Z/E, >98% pure by ^{1}H NMR analysis, containing <2% of co-eluted **11**). The (Z)-enyne **13** was isolated (10 mg, 18% yield). A second column chromatography was performed using silica gel treated with 2% Et_3N (column: 0.5-inch diameter, filled up to 7-inch height) and eluted with 97:3 hexanes/EtOAc to afford a fraction (5 mg) of pure product (100% Z, <1 % co-eluted vinylic iodide **11**), on which characterization was performed.

Characterization data for (*Z*)-vinylic ether **24** (100% *Z*, <1% of co-eluted vinylic iodide **11**):

^{1}H NMR (400 MHz, $CDCl_3$) δ 5.98 (dt, *J* = 6.2, 1.5 Hz, 1H), 4.54 (d, *J* = 5.8 Hz, 2H), 4.37 (d, *J* = 5.8 Hz, 2H) overlapping with 4.37 (app q, *J* = 6.1 Hz, 1H), 3.78 (s, 2H), 2.06 (qd, *J* = 7.2, 1.5 Hz, 2H), 1.35 (s, 3H), 1.27 (d, *J* = 10.0 Hz, 14H), 0.88 (t, *J* = 6.8 Hz, 3H).

^{13}C NMR (101 MHz, $CDCl_3$) δ 145.1, 107.9, 80.0, 77.4, 40.3, 32.1, 29.84, 29.77, 29.66, 29.5, 29.4, 24.0, 22.8, 21.3, 14.3.

HRMS (APCI): *m/z* calcd for $C_{16}H_{31}O_2^+$ $[M+H]^+$ 255.2319, found 255.2312.

IR: 3032 (weak), 2922, 2853, 1663, 1102 cm^{-1}.

(*E*)-vinylic ether 25, from *N*-Boc-4-piperidineethanol and (*E*)-1-iodo-1-decene (**5**):

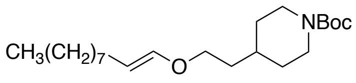

25

Using conditions A, with *N*-Boc-4-piperidineethanol (94.8 mg, 0.41 mmol, 1 equiv) and vinylic iodide **5** (110 mg, 0.41 mmol, 1 equiv) as substrates. The dark brown crude oil was subjected directly to flash column chromatography with silica gel pre-treated with 2% Et_3N in hexanes (column: 1-inch diameter, up to 6-inch height), eluting with 9:1 hexanes/EtOAc (visualized with *p*-anisaldehyde) to afford the title compound **25** as a clear pale-yellow oil (97.1 mg, 64%).

Characterization data for (*E*)-vinylic ether **25**:

^{1}H NMR (400 MHz, $CDCl_3$) δ 6.20 (dt, *J* = 12.6, 1.3 Hz, 1H), 4.75 (dt, *J* = 12.6, 7.3 Hz, 1H), 4.19 – 3.95 (br s, 2H), 3.67 (t, *J* = 6.1 Hz, 2H), 2.68 (br t, *J* = 12.8 Hz, 2H), 1.94 – 1.84 (m, 2H), 1.64 (d, *J* = 3.6 Hz, 2H), 1.60 – 1.54 (m, 2H), 1.45 (s, 9H), 1.34 – 1.20 (m, 12H), 1.11 (qd, *J* = 12.5, 4.4 Hz, 2H), 0.87 (t, *J* = 6.7 Hz, 3H).

^{13}C NMR (101 MHz, $CDCl_3$) δ 155.0, 146.0, 104.5, 79.4, 66.5, 44.1, 36.0, 32.9, 32.2, 32.0, 30.9, 29.6, 29.5, 29.2, 28.6, 27.9, 22.8, 14.3.

HRMS (ESI): *m/z* calcd for $C_{22}H_{42}NO_3^+$ $[M+H]^+$ 368.3159, found 368.3156.

(*Z*)-vinylic ether 26, from *N*-Boc-4-piperidineethanol and (*Z*)-1-iodo-1-undecene (**11**):

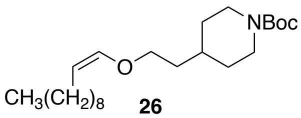

26

Using conditions B, with *N*-Boc-4-piperidineethanol (165 mg, 0.72 mmol, 2 equiv) and vinylic iodide **11** (20:1 Z/E, 100 mg, 0.36 mmol, 1 equiv) as substrates. ^{1}H NMR analysis of the crude product mixture showed 20:1 Z/E ratio favoring vinylic ether **26**, as well as <2% recovered vinylic iodide **11**. The dark brown crude oil was subjected to flash column chromatography with silica gel treated with 2% Et_3N (column: 1-inch diameter, filled up to 7-inch height), eluted with a mixture of 92:6:2 hexanes/EtOAc/Et_3N (TLC in 10:1 hexanes/ethyl acetate, visualized with *p*-anisaldehyde) to afford the title compound **26** as a clear yellow oil (82 mg, 60% combined yield of inseparable diastereomers, 17:1 Z/E, >98% pure by ^{1}H NMR analysis, containing ~1% of co-eluted vinylic iodide **11**). The (Z)-enyne **13** was also isolated (8 mg, 16% yield).

Characterization data for (*Z*)-vinylic ether **26** (17:1 Z/E, containing ~1% of co-eluted vinylic iodide **11**):

^{1}H NMR (600 MHz, $CDCl_3$) δ 5.90 (dt, *J* = 6.2, 1.4 Hz, 1H), 4.34 (td, *J* = 7.3, 6.2 Hz, 1H), 4.07 (broad s, 2H), 3.75 (t, *J* = 6.3 Hz, 2H), 2.69 (broad s, 2H), 2.05 (qd, *J* = 7.3, 1.5 Hz, 2H), 1.67 (d, *J* = 13.1 Hz, 2H), 1.59 – 1.53 (m, 3H), 1.45 (s, 9H), 1.35 – 1.23 (m, 14H), 1.12 (qd, *J* = 12.4, 4.2 Hz, 2H), 0.88 (t, *J* = 7.0 Hz, 3H).

^{13}C NMR (151 MHz, $CDCl_3$) δ 155.0, 144.7, 107.5, 79.4, 69.6, 43.7, 36.4, 33.0, 32.2, 32.1, 30.0, 29.8, 29.7, 29.5, 29.4, 28.6, 24.1, 22.8, 14.3.

HRMS (APCI): *m/z* calcd for $C_{23}H_{44}NO_3^+$ $[M+H]^+$ 382.3316, found 382.3315.

IR: 2922, 2852, 1694, 1663, 1104 cm^{-1}.

Scope of (*E*)- and (*Z*)-selective cross-coupling products from secondary alcohols (Figure 2b)

(*E*)-vinylic ether 27, from cyclohexanol and (*E*)-1-iodo-1-decene (**5**):

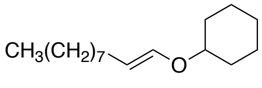

27

Using conditions A, with cyclohexanol (97.8 mg, 0.98 mmol, 2 equiv) and vinylic iodide **5** (130 mg, 0.49 mmol, 1 equiv) as substrates. The dark brown crude oil was subjected directly to flash column chromatography with silica gel pre-treated with 2% Et_3N in hexanes (column: 1-inch diameter, up to 6-inch height), eluting with gradient 98:2 to 97:3 hexanes/EtOAc (visualized with phosphomolybdic acid) to afford the title compound **27** as a clear pale-yellow oil (64.0 mg, 55%).

Characterization data for (*E*)-vinylic ether **27**:

^{1}H NMR (400 MHz, $CDCl_3$) δ 6.08 (dt, *J* = 12.3, 1.3 Hz, 1H), 4.88 (dt, *J* = 12.3, 7.4 Hz, 1H), 3.59 (tt, *J* = 9.2, 3.8 Hz, 1H), 1.88 (tdd, *J* = 8.7, 4.6, 2.4 Hz, 4H), 1.80 – 1.67 (m, 2H), 1.57 – 1.47 (m, 1H), 1.46 – 1.15 (m, 17H), 0.86 (t, *J* = 6.8 Hz, 3H).

^{13}C NMR (101 MHz, $CDCl_3$) δ 144.6, 106.5, 78.1, 32.3, 32.0, 30.8, 29.6, 29.5, 29.2, 27.8, 25.7, 24.0, 22.8, 14.3.

HRMS (APCI): *m/z* calcd for $C_{16}H_{31}O^+$ $[M+H]^+$ 239.2369, found 239.2365.

(*Z*)-vinylic ether 28, from cyclohexanol and (*Z*)-1-iodo-1-undecene (**11**):

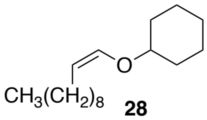

28

Using conditions B, with cyclohexanol (72 mg, 0.72 mmol, 2 equiv) and vinylic iodide **11** (50:1 Z/E, 100 mg, 0.36 mmol, 1 equiv) as substrates. ^{1}H NMR analysis of the crude product mixture showed >20:1 Z/E ratio favoring vinylic ether **28**. The yellow crude oil was subjected to flash column chromatography with silica gel treated with 2% Et_3N (column: 1-inch diameter, filled up to 7-inch height), eluted with pentane (TLC in pentane, visualized with *p*-anisaldehyde stain) in 4-5 mL fractions to afford the title compound **28** as a clear oil (53 mg, 58% combined yield, >20:1 Z/E). The (Z)-enyne **13** was isolated (16 mg, 29% yield). A fraction of vinylic ether **28** (100% Z) was obtained from the column chromatography and was used for full characterization.

Characterization data for (*Z*)-vinylic ether **28**:

^{1}H NMR (400 MHz, $CDCl_3$) δ 5.97 (dt, *J* = 6.3, 1.5 Hz, 1H), 4.33 (td, *J* = 7.2, 6.2 Hz, 1H), 3.56 (tt, *J* = 8.9, 3.7 Hz, 1H), 2.07 (qd, *J* = 7.1, 1.5 Hz, 2H), 1.89 – 1.79 (m, 2H), 1.74 (dp, *J* = 8.9, 3.1 Hz, 2H), 1.58 – 1.22 (m, 20H), 0.87 (t, *J* = 6.6 Hz, 3H).

^{13}C NMR (101 MHz, $CDCl_3$) δ 143.4, 107.3, 78.9, 32.3, 32.0, 29.9, 29.7, 29.5, 29.4, 29.3, 25.6, 24.0, 23.6, 22.7, 14.1.

HRMS (APCI): *m/z* calcd for $C_{17}H_{33}O^+$ $[M+H]^+$ 253.2526, found 253.2518.

IR (neat): 3029 (weak), 2925, 2854, 1663, 1233 (weak), 1097 cm^{-1}.

R_f (Z-isomer) (pentane) = 0.17.

R_f (E-isomer) (pentane) = 0.07.

(*E*)-vinylic ether 29, from *N*-methyl-4-piperidinol and (*E*)-1-iodo-1-decene (**5**):

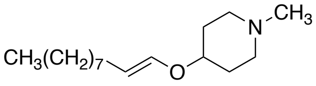

29

Using conditions A, with *N*-methyl-4-piperidinol (119 mg, 0.98 mmol, 2 equiv) and vinylic iodide **5** (130 mg, 0.49 mmol, 1 equiv) as substrates. The dark brown crude oil was subjected directly to flash column chromatography with silica gel pre-treated with 3% Et_3N in EtOAc (column: 1-inch diameter, up to 6-inch height), eluting with 100:3 EtOAc/Et_3N (visualized with phosphomolybdic acid) to afford the title compound **29** as a clear pale-yellow oil (92.3 mg, 75%).

Characterization data for (*E*)-vinylic ether **29**:

^{1}H NMR (400 MHz, $CDCl_3$) δ 6.07 (dt, *J* = 12.4, 1.3 Hz, 1H), 4.90 (dt, *J* = 12.3, 7.4 Hz, 1H), 3.67 (br tt, *J* = 8.2, 3.9 Hz, 1H), 2.63 (br t, *J* = 9.9 Hz, 2H), 2.25 (s, 3H), 2.17 (br t, *J* = 9.4 Hz, 2H), 1.88 (dtd, *J* = 10.3, 5.6, 2.0 Hz, 4H), 1.70 (dtd, *J* = 12.6, 8.4, 3.6 Hz, 2H), 1.39 – 1.17 (m, 12H), 0.87 (t, *J* = 6.8 Hz, 3H).

^{13}C NMR (101 MHz, $CDCl_3$) δ 144.2, 107.2, 74.7, 52.9, 46.3, 32.0, 31.3, 30.7, 29.6, 29.4, 29.2, 27.8, 22.8, 14.2.

HRMS (APCI): *m/z* calcd for $C_{16}H_{32}NO^+$ $[M+H]^+$ 254.2478, found 254.2476.

(*Z*)-vinylic ether 30, from *N*-methyl-4-piperidinol and (*Z*)-1-iodo-1-undecene (**11**):

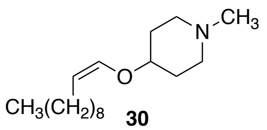

30

Using conditions B, with *N*-methyl-4-piperidinol (83 mg, 0.72 mmol, 2 equiv) and vinylic iodide **11** (20:1 Z/E, 100 mg, 0.36 mmol, 1 equiv) as substrates. ^{1}H NMR analysis of the crude product mixture showed 20:1 Z/E ratio favoring vinylic ether **30**, as well as <2% recovered vinylic iodide **11**. The dark brown crude oil was subjected to flash column chromatography with silica gel treated with 3% Et_3N (column: 1-inch diameter, filled up to 7-inch height), eluted with an eluent mixture of 3% Et_3N in 5:1 hexanes/EtOAc (TLC in 4% Et_3N in 5:1 hexanes/EtOAc, visualized with *p*-anisaldehyde stain) to afford the title compound **30** as a clear yellow oil (49 mg, 51% yield, 13:1 Z/E, >98% pure by ^{1}H NMR analysis, containing ~1% of co-eluted vinylic iodide **11**). The (Z)-enyne **13** was also isolated (11 mg, 20% yield).

Characterization data for (*Z*)-vinylic ether **30** (13:1 Z/E, containing ~1% of co-eluted vinylic iodide **11**):

^{1}H NMR (400 MHz, $CDCl_3$) δ 5.94 (dt, *J* = 6.2, 1.5 Hz, 1H), 4.38 (td, *J* = 7.3, 6.2 Hz, 1H), 3.65 (broad s, 1H), 2.71 – 2.50 (broad s, 2H), 2.31 – 2.13 (m, 5H), 2.07 (qd, *J* = 7.1, 1.4 Hz, 2H), 1.91 – 1.82 (m, 2H), 1.78 – 1.65 (m, 2H), 1.35 – 1.22 (m, 14H), 0.87 (t, *J* = 6.7 Hz, 3H).

^{13}C NMR (101 MHz, $CDCl_3$) δ 143.0, 108.2, 77.4, 52.7, 46.5, 32.1, 31.5, 29.9, 29.8, 29.7, 29.5, 29.4, 24.1, 22.8, 14.3.

HRMS (APCI): *m/z* calcd for $C_{17}H_{34}NO^+$ $[M+H]^+$ 268.2635, found 268.2633.

IR: 3030 (weak), 2922, 2852, 2779, 1662, 1092 cm^{-1}.

R_f (4% Et_3N in 5:1 hexanes/EtOAc) = 0.13 - 0.15.

(*E*)-vinylic ether 31, from 4-penten-2-ol and (*E*)-1-iodo-1-decene (**5**):

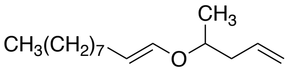

31

Using conditions A, with 4-penten-2-ol (84.1 mg, 0.98 mmol, 2 equiv) and vinylic iodide **5** (130 mg, 0.49 mmol, 1 equiv) as substrates. The dark brown crude oil was subjected directly to flash column chromatography with silica gel pre-treated with 2% Et_3N in hexanes (column: 1-inch diameter, up to 6-inch height), eluting with 100% hexanes (visualized with *p*-anisaldehyde) to afford the title compound **31** as a clear pale-yellow oil (48.6 mg, 44% yield).

Characterization data for (*E*)-vinylic ether **31**:

^{1}H NMR (400 MHz, $CDCl_3$) δ 6.06 (dt, *J* = 12.3, 1.3 Hz, 1H), 5.80 (ddt, *J* = 17.2, 10.2, 7.1 Hz, 1H), 5.12 – 5.07 (m, 1H), 5.06 (dtd, *J* = 3.2, 2.1, 1.3 Hz, 1H), 4.88 (dt, *J* = 12.4, 7.4 Hz, 1H), 3.81 (sextet, *J* = 6.2 Hz, 1H), 2.37 (dddt, *J* = 14.0, 7.0, 5.7, 1.4 Hz, 1H), 2.29 – 2.16 (m, 1H), 1.89 (qd, *J* = 7.3, 1.3 Hz, 2H), 1.36 – 1.21 (m, 12H), 1.19 (d, *J* = 6.2 Hz, 3H), 0.88 (t, *J* = 6.6 Hz, 3H).

^{13}C NMR (101 MHz, $CDCl_3$) δ 144.8, 134.5, 117.4, 106.9, 75.7, 40.8, 32.0, 30.7, 29.6, 29.5, 29.2, 27.8, 22.8, 19.7, 14.3.

HRMS (APCI): *m/z* calcd for $C_{15}H_{29}O^+$ $[M+H]^+$ 225.2212, found 225.2210.

(*Z*)-vinylic ether 32, from 4-penten-2-ol and (*Z*)-1-iodo-1-undecene (**11**):

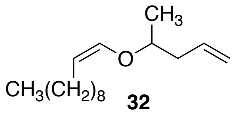

32

Using conditions B, with 4-penten-2-ol (62 mg, 0.72 mmol, 2 equiv) and vinylic iodide **11** (50:1 Z/E, 100 mg, 0.36 mmol, 1 equiv) as substrates. ^{1}H NMR analysis of the crude product mixture showed >20:1 Z/E ratio favoring vinylic ether **32**, and <5% recovered vinylic iodide **11**. The dark brown crude oil was subjected to flash column chromatography with silica gel treated with 2% Et_3N (column: 1-inch diameter, filled up to 7-inch height), carefully eluted with pentane in 4-5 mL fractions (TLC in 100% hexanes, visualized with *p*-anisaldehyde stain) to afford the title compound **32** as a clear oil (19 mg, 22% yield, >20:1 Z/E). The (Z)-enyne **13** was isolated (16 mg, 29% yield).

Characterization data for (*Z*)-vinylic ether **30** (>20:1 Z/E):

^{1}H NMR (400 MHz, $CDCl_3$) δ 5.95 (dt, *J* = 6.3, 1.5 Hz, 1H), 5.81 (ddt, *J* = 17.2, 10.2, 7.1 Hz, 1H), 5.09 (ddt, *J* = 12.1, 2.2, 0.9 Hz, 1H), 5.05 (ddt, *J* = 4.3, 2.1, 0.9 Hz, 1H), 4.34 (td, *J* = 7.3, 6.3 Hz, 1H), 3.75 (apparent sextet, *J* = 6.2 Hz, 1H), 2.36 (dddt, *J* = 14.1, 7.1, 5.8, 1.4 Hz, 1H), 2.23 (dddt, *J* = 13.9, 7.5, 6.4, 1.2 Hz, 1H), 2.11 – 2.01 (m, 2H), 1.28 (d, *J* = 14.6 Hz, 14H), 1.19 (d, *J* = 6.2 Hz, 3H), 0.88 (t, *J* = 6.8 Hz, 3H).

^{13}C NMR (101 MHz, $CDCl_3$) δ 143.6, 134.6, 117.3, 107.7, 77.1, 41.1, 32.1, 30.0, 29.8, 29.7, 29.5, 29.4, 24.1, 22.9, 20.0, 14.3.

HRMS (APCI): *m/z* calcd for $C_{16}H_{31}O^+$ $[M+H]^+$ 239.2369, found 239.2371.

IR (neat): 3100 (weak), 3030 (weak), 2922, 2852, 1662, 1093 cm^{-1}.

(*E*)-vinylic ether 33, from 1,2:5,6-di-*O*-isopropylidene-α-D-glucofuranose and (*E*)-1-iodo-1-decene (**5**):

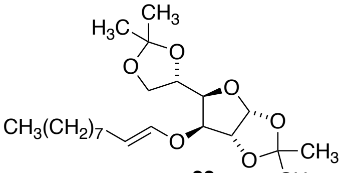

33

Using conditions A, with 1,2:5,6-di-*O*-isopropylidene-α-D-glucofuranose (254.3 mg, 0.98 mmol, 2 equiv) and vinylic iodide **5** (130 mg, 0.49 mmol, 1 equiv) as substrates. The dark brown crude oil was subjected directly to flash column chromatography with silica gel pre-treated with 2% Et_3N in hexanes (column: 1-inch diameter, up to 6-inch height), eluting with 92:8 hexanes/EtOAc (visualized with *p*-anisaldehyde) to afford the title compound **33** as a clear pale-yellow oil (70.0 mg, 36% yield).

Characterization data for (*E*)-vinylic ether **33**:

^{1}H NMR (400 MHz, $CDCl_3$) δ 6.14 (dt, *J* = 12.6, 1.3 Hz, 1H), 5.87 (d, *J* = 3.8 Hz, 1H), 4.94 (dt, *J* = 12.5, 7.4 Hz, 1H), 4.57 (d, *J* = 3.8 Hz, 1H), 4.31 (dt, *J* = 7.7, 5.8 Hz, 1H), 4.23 (d, *J* = 3.0 Hz, 1H), 4.16 (dd, *J* = 7.6, 3.0 Hz, 1H), 4.08 (dd, *J* = 8.6, 6.1 Hz, 1H), 4.01 (dd, *J* = 8.6, 5.4 Hz, 1H), 1.90 (qd, *J* = 7.1, 1.3 Hz, 2H), 1.51 (s, 3H), 1.43 (s, 3H), 1.34 (s, 3H), 1.31 (s, 3H), 1.30 – 1.16 (m, 12H), 0.90 (t, *J* = 6.5 Hz, 3H).

^{13}C NMR (101 MHz, $CDCl_3$) δ 144.2, 112.0, 109.3, 107.7, 105.3, 82.3, 81.2, 80.7, 72.3, 67.2, 32.0, 30.5, 29.6, 29.4, 29.3, 27.7, 27.0, 26.9, 26.4, 25.5, 22.8, 14.3.

HRMS (APCI): *m/z* calcd for $C_{22}H_{39}O_6^+$ $[M+H]^+$ 399.2741, found 399.2729.

$[\alpha]^{22}_D$ -12.0 (c = 0.1, $CHCl_3$).

(*Z*)-vinylic ether 34, from 1,2:5,6-di-*O*-isopropylidene-α-D-glucofuranose and (*Z*)-1-iodo-1-undecene (**11**):

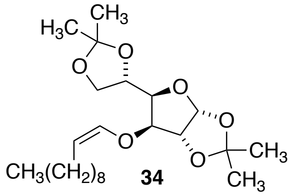

34

Using conditions B, with 1,2:5,6-di-*O*-isopropylidene-α-D-glucofuranose (187 mg, 0.72 mmol, 2 equiv) and vinylic iodide **11** (20:1 Z/E, 100 mg, 0.36 mmol, 1 equiv) as substrates. ^{1}H NMR analysis of the crude product mixture showed 8:1 Z/E ratio favoring vinylic ether **34**. The dark brown crude oil was subjected to flash column chromatography with neutral alumina (column: 1-inch diameter, filled up to 3-inch height), eluted with a gradient of 10:1 to 5:1 hexanes/EtOAc (TLC in 10:1 hexanes/EtOAc, visualized with *p*-anisaldehyde) to afford the title compound **34** as a clear pale-yellow oil (21 mg, 14% combined yield of inseparable diastereomers, 7:1 Z/E). A fraction of pure product **34** (17:1 Z/E) was obtained for full characterization. The (Z)-enyne **13** was isolated (7 mg, 13% yield).

Characterization data for (*Z*)-vinylic ether **34** (17:1 Z/E):

^{1}H NMR (600 MHz, $CDCl_3$) δ 6.00 (dq, *J* = 6.2, 1.5 Hz, 1H), 5.89 (dd, *J* = 3.8, 1.2 Hz, 1H), 4.54 (dd, *J* = 3.7, 1.2 Hz, 1H), 4.47 (tdd, *J* = 7.4, 5.9, 1.3 Hz, 1H), 4.28 – 4.33 (app. q, 1H), 4.21 – 4.14 (m, 2H), 4.09 (ddd, *J* = 7.7, 6.2, 1.3 Hz, 1H), 4.02 (ddd, *J* = 8.6, 5.6, 1.3 Hz, 1H), 2.02 (qt, *J* = 7.3, 1.4 Hz, 2H), 1.50 (s, 3H), 1.43 (s, 3H), 1.34 (s, 3H), 1.31 (s, 3H), 1.23 (m, 14H, overlapping with minor *E*-isomer), 0.87 (td, *J* = 7.0, 1.3 Hz, 3H).

^{13}C NMR (151 MHz, $CDCl_3$) δ 143.2, 112.2, 109.5, 109.2, 105.3, 83.3, 83.0, 80.9, 72.5, 67.2, 32.1, 29.8, 29.69, 29.66, 29.5, 29.4, 27.0, 26.9, 26.4, 25.4, 24.1, 22.8, 14.3.

HRMS (APCI): *m/z* calcd for $C_{23}H_{41}O_6^+$ $[M+H]^+$ 413.2898, found 413.2891.

IR: 2923, 2853, 1664, 1215 (medium-weak), 1074, 1021 cm^{-1}.

$[\alpha]^{22}_D$ -8.6 (c = 0.1, $CHCl_3$).

(*E*)-vinylic ether 35, from (-)-menthol and (*E*)-1-iodo-1-decene (**5**):

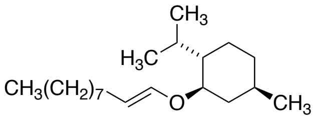

35

Using conditions A, with (-)-menthol (153 mg, 0.98 mmol, 2 equiv) and vinylic iodide **5** (130 mg, 0.49 mmol, 1 equiv) as substrates. The dark brown crude oil was subjected directly to flash column chromatography with silica gel pre-treated with 2% Et_3N in hexanes (column: 1-inch diameter, up to 6-inch height), eluting with 100% hexanes (visualized with *p*-anisaldehyde) to afford the title compound **35** as a clear pale-yellow oil (59.0 mg, 41% yield).

Characterization data for (*E*)-vinylic ether **35**:

^{1}H NMR (400 MHz, $CDCl_3$) δ 6.07 (dt, *J* = 12.3, 1.3 Hz, 1H), 4.86 (dt, *J* = 12.3, 7.4 Hz, 1H), 3.38 (td, *J* = 10.7, 4.3 Hz, 1H), 2.13 (pd, *J* = 7.0, 2.8 Hz, 1H), 2.08 – 1.99 (m, 1H), 1.88 (qd, *J* = 7.3, 1.4 Hz, 2H), 1.64 (ddq, *J* = 12.6, 6.3, 3.2 Hz, 2H), 1.42 – 1.29 (m, 1H), 1.31 – 1.20 (m, 15H), 1.08 – 0.80 (m, 4H), 0.90 (d, *J* = 2.2 Hz, 3H), 0.88 (d, *J* = 2.1 Hz, 3H), 0.77 (d, *J* = 6.9 Hz, 3H).

^{13}C NMR (101 MHz, $CDCl_3$) δ 145.4, 106.0, 80.2, 48.0, 41.2, 34.6, 32.0, 31.7, 30.8, 29.6, 29.5, 29.2, 27.8, 25.8, 23.5, 22.8, 22.3, 20.9, 16.4, 14.3.

HRMS (APCI): *m/z* calcd for $C_{20}H_{39}O^+$ $[M+H]^+$ 295.2995, found 295.2993.

$[\alpha]^{23}_D$ -25.2 (c = 0.1, $CHCl_3$)

(*Z*)-vinylic ether 36, from (-)-menthol and (*Z*)-1-iodo-1-undecene (**11**):

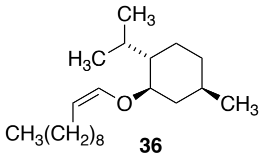

36

Using conditions B, with (-)-menthol (112 mg, 0.72 mmol, 2 equiv) and vinylic iodide **11** (50:1 Z/E, 100 mg, 0.36 mmol, 1 equiv) as substrates. ^{1}H NMR analysis of the crude product mixture showed >20:1 Z/E ratio favoring vinylic ether **36**, along with <5% of recovered vinylic iodide **11**. The dark green crude oil was subjected to flash column chromatography with silica gel treated with 2% Et_3N (column: 1-inch diameter, filled up to 10-inch height), eluted with pentane (TLC in pentane, visualized with *p*-anisaldehyde stain) in 4-5 mL fractions to afford the title compound **36** in an inseparable mixture with (*Z*)-enyne **13** as a clear pale-yellow oil (40 mg combined yield, 5.6:1 ratio of vinylic ether **36** : enyne **13** based on ^{1}H NMR analysis). Based on the combined yield, the title compound **36** was estimated to be 0.11 mmol (31% yield, >20:1 Z/E based on crude ^{1}H NMR analysis). Enyne **13** was obtained in 29% combined yield, of pure enyne and estimated enyne impurity with vinylic ether **36** (16 mg). Due to the difficulty in purification, characterization of this compound was performed on the mixture of 5.6:1 vinylic ether **36** : enyne **13**.

Characterization data for (*Z*)-vinylic ether **36** (5.6 : 1 mixture of vinylic ether to enyne):

^{1}H NMR (400 MHz, $CDCl_3$) δ 5.98 (dt, *J* = 6.2, 1.5 Hz, 1H), 4.27 (td, *J* = 7.2, 6.2 Hz, 1H), 3.32 (td, *J* = 10.7, 4.3 Hz, 1H), 2.14 (quintet of doublets, *J* = 7.0, 2.8 Hz, 1H), 2.09 – 2.02 (m, 2H), 2.02 – 1.96 (m, 1H), 1.64 (dddd, *J* = 12.7, 9.3, 4.9, 2.6 Hz, 2H), 1.42 – 1.34 (m, 2H), 1.33 – 1.23 (m, 14H, overlapping with enyne side product), 1.05 – 0.93 (m, 2H), 0.92 – 0.82 (m, 11H, overlapping with enyne side product), 0.77 (d, *J* = 6.9 Hz, 3H).

^{13}C NMR (101 MHz, $CDCl_3$) δ 144.2, 106.5, 81.5, 47.9, 41.7, 34.6, 32.1, 31.7, 30.1, 29.8, 29.7, 29.5, 29.4, 26.0, 24.1, 23.7, 22.9, 22.3, 20.9, 16.6, 14.3.

HRMS (APCI): *m/z* calcd for $C_{21}H_{41}O^+$ $[M+H]^+$ 309.3152, found 309.3148.

IR (neat): 3030 (weak), 2923, 2854, 1662, 1217 (weak), 1102 cm^{-1}.

$[\alpha]^{22}_D$ -15.4 (c = 0.1, $CHCl_3$).

Scope of (*E*)- and (*Z*)-selective cross-coupling products with other disubstituted vinylic iodides (Figure 3a)

(*E*)-vinylic ether 37, from 1,2:3,4-di-*O*-isopropylidene-α-D-galactopyranose (**8**) and (*E*)-(2-iodovinyl)cyclohexane (**S2**):

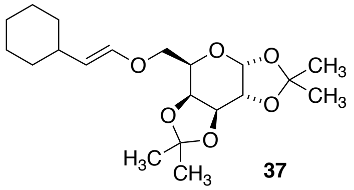

37

Using conditions A, with alcohol **8** (220.3 mg, 0.85 mmol, 2 equiv) and vinylic iodide **S2** (100 mg, 0.42 mmol, 1 equiv) as substrates. The dark brown crude oil was subjected directly to flash column chromatography with silica gel pre-treated with 2% Et_3N in hexanes (column: 1-inch diameter, up to 6-inch height), eluting with 92:8 hexanes/EtOAc (visualized with phosphomolybdic acid stain) to afford the title compound **37** as a clear pale-yellow oil (27.0 mg, 17% yield). 47% of vinylic iodide **S2** was recovered.

Characterization data for (*E*)-vinylic ether **37**:

^{1}H NMR (400 MHz, $CDCl_3$) δ 6.26 (dd, *J* = 12.8, 1.0 Hz, 1H), 5.54 (d, *J* = 5.0 Hz, 1H), 4.76 (dd, *J* = 12.7, 7.7 Hz, 1H), 4.62 (dd, *J* = 7.9, 2.4 Hz, 1H), 4.32 (dd, *J* = 5.0, 2.4 Hz, 1H), 4.26 (dd, *J* = 7.9, 1.9 Hz, 1H), 4.02 (ddd, *J* = 7.6, 5.6, 2.1 Hz, 1H), 3.80 (qt, *J* = 7.1, 5.4 Hz, 2H), 1.87 (tdd, *J* = 11.2, 8.9, 6.9 Hz, 1H), 1.74 – 1.60 (m, 5H), 1.53 (s, 3H), 1.46 (s, 3H), 1.35 (s, 3H), 1.33 (s, 3H), 1.29 – 0.97 (m, 5H).

^{13}C NMR (101 MHz, $CDCl_3$) δ 144.7, 111.2, 109.6, 108.8, 96.5, 71.2, 70.8, 70.7, 67.5, 66.4, 37.1, 34.4, 26.3, 26.24, 26.22, 26.1, 25.1, 24.6.

HRMS (APCI): *m/z* calcd for $C_{20}H_{33}O_6^+$ $[M+H]^+$ 369.2272, found 369.2268.

$[\alpha]^{22}_D$ -34.6 (c = 0.1, $CHCl_3$).

(*Z*)-vinylic ether 38, from 1,2:3,4-di-*O*-isopropylidene-α-D-galactopyranose (**8**) and (*Z*)-(2-iodovinyl)cyclohexane (**S9**):

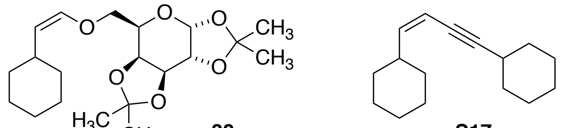

38 **S17**

Using conditions B, with alcohol **8** (187 mg, 0.72 mmol, 2 equiv) and vinylic iodide **S9** (17:1 Z/E) (85 mg, 0.36 mmol, 1 equiv) as substrates. ^{1}H NMR analysis of the crude product mixture showed 17:1 Z/E ratio favoring vinylic ether **38**. The dark brown crude oil was subjected to flash column chromatography with silica gel treated with 2% Et_3N (column: 1-inch diameter, filled up to 7-inch height), eluted with a gradient of 97:3 to 95:5 hexanes/EtOAc (TLC in 10:1 hexanes/EtOAc, visualized with *p*-anisaldehyde stain) to afford the title compound **38** as a clear pale-yellow oil (90 mg, 68% combined yield, 14:1 *Z*/*E*). (*Z*)-Enyne **S17** was also isolated (10 mg, 26% yield).

Characterization data for (*Z*)-vinylic ether **38** (14:1 *Z*/*E*):

^{1}H NMR (600 MHz, $CDCl_3$) δ 5.87 (d, *J* = 6.2 Hz, 1H), 5.52 (d, *J* = 5.0 Hz, 1H), 4.61 (dd, *J* = 8.1, 2.5 Hz, 1H), 4.31 (dt, *J* = 3.7, 1.7 Hz, 1H), 4.25 (dd, *J* = 9.1, 7.0 Hz, 2H), 3.98 (td, *J* = 6.4, 2.0 Hz, 1H), 3.93 – 3.87 (m, 1H), 3.86 – 3.81 (m, 1H), 2.44 (dtt, *J* = 12.4, 8.2, 3.5 Hz, 1H), 1.71 – 1.64 (m, 4H), 1.63 – 1.57 (m, 1H), 1.53 (s, 3H), 1.45 (s, 3H), 1.34 (d, *J* = 5.4 Hz, 6H), 1.28 (ddd, *J* = 15.5, 10.2, 3.4 Hz, 2H), 1.16 (ddt, *J* = 16.5, 13.2, 6.6 Hz, 1H), 1.08 – 0.98 (m, 2H).

^{13}C NMR (151 MHz, $CDCl_3$) δ 143.6, 114.1, 109.5, 108.8, 96.4, 71.1, 70.83, 70.75, 66.9, 33.54, 33.51, 26.4, 26.21, 26.16 (2 carbons overlapping), 26.1, 25.1, 24.5.

HRMS (APCI): *m/z* calcd for $C_{20}H_{32}O_6^+$ [M+H]$^+$ 369.2272, found 369.2264.

IR (neat): 3015 (weak), 2921, 2849, 1662, 1211, 1069 cm^{-1}.

$[\alpha]^{22}_D$ -64.3 (c = 0.103, $CHCl_3$).

Characterization data for enyne **S17** (17:1 *Z*/*E*):

^{1}H NMR (400 MHz, $CDCl_3$) δ 5.67 (dd, *J* = 10.7, 9.0 Hz, 1H), 5.35 (ddd, *J* = 10.7, 2.0, 0.9 Hz, 1H), 2.63 – 2.48 (m, 2H), 1.85 – 1.76 (m, 2H), 1.76 – 1.67 (m, 6H), 1.67 – 1.61 (m, 1H), 1.50 (td, *J* = 9.2, 4.0 Hz, 3H), 1.39 – 1.27 (m, 5H), 1.23 – 1.04 (m, 4H), 0.91 – 0.80 (m, 1H). *NMR signals from 1.9 – 1.0 ppm have overlapping resonances between the (*Z*)- and the (*E*)-isomer.

^{13}C NMR (101 MHz, $CDCl_3$) δ 148.3, 107.6, 98.4, 77.6 (overlapping with $CDCl_3$), 39.2, 32.9, 32.5 (2 overlapping carbons), 29.9, 26.2, 26.1, 26.0 (2 overlapping carbons), 24.9.

HRMS (APCI): *m/z* calcd for $C_{16}H_{25}^+$ [M+H]$^+$ 217.1951, found 217.1950.

(*E*)-vinylic ether 39, from 1,2:3,4-di-*O*-isopropylidene-α-D-galactopyranose (**8**) and (*S*,*E*)-4-(2-iodovinyl)-2,2-dimethyl-1,3-dioxolane (**S6**):

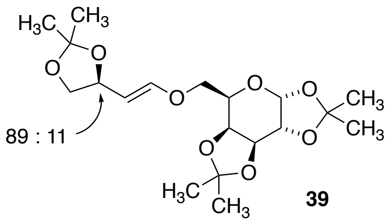

39

Using conditions A, with alcohol **8** (188 mg, 0.71 mmol, 2 equiv) and vinylic iodide **S6** (90 mg, 0.35 mmol, 1 equiv) as substrates. The dark brown crude oil was subjected directly to flash column chromatography with silica gel pre-treated with 2% Et_3N in hexanes (column: 1-inch diameter, up to 6-inch height), eluting with 9:1 hexanes/EtOAc (visualized with *p*-anisaldehyde) to afford the title compound **39** as a viscous residue (92.4 mg, 67% yield, 89:11 dr arising from vinylic iodide **S6**).

Characterization data for (*E*)-vinylic ether **39** (89:11 dr):

^{1}H NMR (400 MHz, $CDCl_3$) δ 6.59 (d, *J* = 12.6 Hz, 1H), 5.53 (d, *J* = 5.0 Hz, 1H), 4.78 (dd, *J* = 12.7, 9.0 Hz, 1H), 4.62 (dd, *J* = 7.9, 2.5 Hz, 1H), 4.43 (td, *J* = 8.4, 5.9 Hz, 1H), 4.32 (dd, *J* = 5.0, 2.5 Hz, 1H), 4.24 (dd, *J* = 7.9, 1.9 Hz, 1H), 4.07 – 3.98 (m, 2H), 3.91 – 3.85 (m, 2H), 3.53 (t, *J* = 8.1 Hz, 1H), 1.53 (s, 3H), 1.44 (s, 3H), 1.39 (s, 3H), 1.36 (s, 3H), 1.32 (s, 3H), 1.31 (s, 3H).

The diastereomers are distinguished in the ^{1}H NMR spectrum by two sets of doublets at 6.59 that partially overlap. The dr is determined from integrating the resonances at δ 6.62 (s, 1H) and 6.60 (s, 9H).

^{13}C NMR (101 MHz, $CDCl_3$) δ 150.9, 109.7, 108.93, 108.88, 101.4, 96.4, 75.1, 71.1, 70.7, 70.6, 70.1, 68.0, 66.2, 27.0, 26.18, 26.17, 26.1, 25.1, 24.6.

All observed ^{13}C resonances are for the major diastereomer.

$[\alpha]^{23}_D$ -63.8 (c = 0.1, $CHCl_3$)

HRMS (ESI): *m/z* calcd for $C_{19}H_{31}O_8^+$ $[M+H]^+$ 387.2013, molecular ion not observed.

However, we observed a peak at *m/z* 385.1857 corresponding to formula $C_{19}H_{29}O_8^+$, calcd 385.1861. This corresponds to loss of hydride in the mass spectrometer, with proposed structure **S18**.

We also observed an identical mass peak in the mass spectrum of the (*Z*)-isomer **40**, see below.

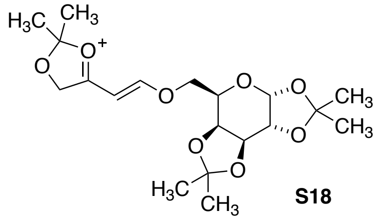
S18

(*Z*)-vinylic ether 40, from 1,2:3,4-di-*O*-isopropylidene-α-D-galactopyranose (**8**) and (*S*,*Z*)-4-(2-iodovinyl)-2,2-dimethyl-1,3-dioxolane (**S10**):

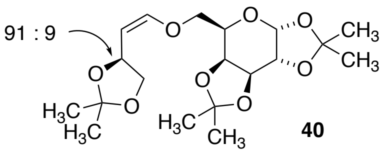

40

Using conditions B, with alcohol **8** (187 mg, 0.72 mmol, 2 equiv) and vinylic iodide **S10** (10:1 Z/E, 91 mg, 0.36 mmol, 1 equiv) as substrates. ^{1}H NMR analysis of the crude product mixture showed 5:1 Z/E ratio favoring vinylic ether **40**, and a side-product with characteristics consistent with an enyne, as a ~3:1 Z/E ratio. The dark brown crude oil was subjected to flash column chromatography with silica gel treated with 2% Et_3N (column: 1-inch diameter, filled up to 7-inch height), eluted with a mixture of 88:10:2 hexanes/EtOAc/Et_3N (TLC in 4:1 hexanes/EtOAc, visualized with *p*-anisaldehyde stain) to afford the title compound **40** as a clear pale yellow oil (42 mg, 30% combined yield, 4:1 Z/E, 10:1 dr for the major *Z*-isomer, ~95% pure based on ^{1}H NMR analysis). The putative enyne side-product had similar ^{1}H NMR resonances in the alkene region as enyne **S18** from pseudo-homocoupling of iodide **S9**, but was not successfully purified.

Characterization data for (*Z*)-vinylic ether **40** (4:1 *Z*/*E*; 91:9 dr):

^{1}H NMR (600 MHz, CDCl$_3$) δ 6.14 (dd, *J* = 6.3, 1.1 Hz, 1H), 5.51 (d, *J* = 5.0 Hz, 1H), 5.02 (tdd, *J* = 8.1, 6.0, 1.2 Hz, 1H), 4.61 (dt, *J* = 8.0, 1.8 Hz, 1H), 4.51 (dd, *J* = 8.1, 6.2 Hz, 1H), 4.31 (dd, *J* = 5.0, 2.4 Hz, 1H), 4.23 (dd, *J* = 7.9, 1.6 Hz, 1H), 4.10 (dd, *J* = 8.0, 6.0 Hz, 1H), 3.95 – 3.87 (m, 3H), 3.50 (t, *J* = 8.0 Hz, 1H), 1.52 (s, 3H), 1.44 – 1.43 (m, 3H), 1.40 (d, *J* = 0.7 Hz, 3H), 1.37 (d, *J* = 0.7 Hz, 3H), 1.34 – 1.33 (m, 3H), 1.32 (d, *J* = 0.9 Hz, 3H).

^{13}C NMR (151 MHz, CDCl$_3$) δ 148.7, 109.8, 109.1, 108.9, 105.4, 96.6, 71.3, 71.0, 70.93, 70.92, 70.6, 70.0, 66.6, 27.2, 26.4, 26.33, 26.29, 25.3, 24.8.

IR (neat): 2985, 2933, 1664, 1209, 1066 cm^{-1}.

$[\alpha]^{22}_D$ -67.8 (c = 0.1, $CHCl_3$).

HRMS (APCI): *m/z* calcd for $C_{19}H_{31}O_8^+$ $[M+H]^+$ 387.2013, molecular ion not observed, analogous to characterization of the (*E*)-isomer **39**. We observed a peak at *m/z* 385.1857 corresponding to formula $C_{19}H_{29}O_8^+$, calcd 385.1861. This corresponds to loss of hydride in the mass spectrometer, with proposed structure **S19**.

The APCI HRMS also showed *m/z* calcd for fragment $C_{16}H_{25}O_7^+$ $[M-(CH_3)_2CO+H]^+$ 329.1595, found 329.1589. The proposed fragment structure **S20** corresponds to loss of acetone upon protonation of an acetonide oxygen.

In an electrospray ionization (ESI) HRMS, we observed a Na^+ adduct corresponding to the same organic structure as **S19**, *m/z* calcd for $C_{19}H_{29}O_8Na^+$ $[M+Na]^+$ 409.1838, found 409.1844.

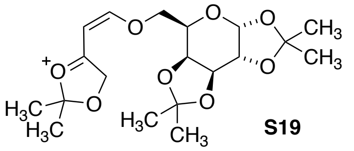

S19

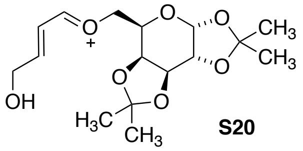

S20

Scope of (*E*)- and (*Z*)-selective cross-coupling products with trisubstituted vinylic iodides (Figure 3b)

(*E*)-vinylic ether 41, from 1,2:3,4-di-*O*-isopropylidene-α-D-galactopyranose (**8**) and (*E*)-1-iodo-2-methyldec-1-ene (**S13**):

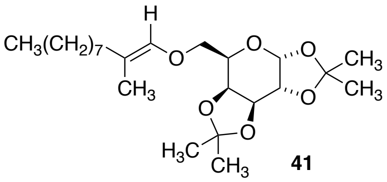

41

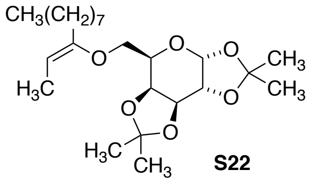

S22

Using conditions B, with alcohol **8** (187 mg, 0.72 mmol, 2 equiv) and (*E*)-1-iodo-2-methyldec-1-ene (**S13** : **S14**, 95 : 5 regioisomer ratio, containing 15% 2-methyl-1-decene, 110 mg, 0.36 mmol, 1 equiv) as substrates. The dark yellow crude oil was subjected to flash column chromatography with silica gel treated with 2% Et_3N (column: 1-inch diameter, filled up to 7-inch height), eluted with a gradient of 98:2 to 90:10 hexanes/EtOAc (TLC in 10:1 hexanes/EtOAc, visualized with *p*-anisaldehyde stain) to afford the title compound **41** as a clear yellow oil (120 mg, 80% yield, ~95% pure by ^{1}H NMR analysis). A second column chromatography was performed using silica gel treated with 2% Et_3N, eluted with 3% EtOAc in hexanes (column: 0.5', filled up to 7' height) to obtain a fraction (20 mg) of vinylic ether **41**, containing ~5% of regioisomer **S22**, for full characterization.

Characterization data for (*E*)-vinylic ether **41** (major regioisomer)

^{1}H NMR (400 MHz, $CDCl_3$) δ 5.82 (apparent sextet, *J* = 1.3 Hz, 1H), 5.53 (d, *J* = 4.9 Hz, 1H), 4.60 (dd, *J* = 7.9, 2.4 Hz, 1H), 4.31 (dd, *J* = 5.0, 2.4 Hz, 1H), 4.25 (dd, *J* = 7.9, 1.9 Hz, 1H), 3.97 (td, *J* = 6.2, 1.8 Hz, 1H), 3.86 (dd, *J* = 10.7, 5.9 Hz, 1H), 3.81 (dd, *J* = 10.7, 6.7 Hz, 1H), 1.83 (t, *J* = 6.9 Hz, 2H), 1.58 (d, *J* = 1.3 Hz, 3H), 1.52 (s, 3H), 1.44 (s, 3H), 1.34 (s, 3H), 1.32 (s, 3H), 1.32 – 1.18 (m, 11H), 0.86 (t, *J* = 6.7 Hz, 3H).

^{13}C NMR (151 MHz, $CDCl_3$) δ 140.4, 115.8, 109.5, 108.8, 96.5, 71.2, 70.83, 70.77, 70.3, 66.6, 34.0, 32.0, 29.6, 29.44, 29.39, 28.1, 26.13, 26.11, 25.1, 24.6, 22.7, 14.3, 13.1.

HRMS (APCI): *m/z* calcd for $C_{23}H_{41}O_6^+$ $[M+H]^+$ 413.2898, found 413.2888.

IR (neat): 2925, 2855, 1685, 1212, 1071 cm^{-1}.

$[\alpha]^{22}_D$ -101.8 (c = 0.1, $CHCl_3$).

(*E*)-vinylic ether 42, from cyclohexanol and (*E*)-1-iodo-2-methyldec-1-ene (**S13**):

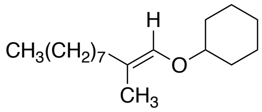

42

Using conditions B, with cyclohexanol (72 mg, 0.72 mmol, 2 equiv) and (*E*)-1-iodo-2-methyldec-1-ene (**S13** : **S14**, 95 : 5 regioisomer ratio, containing 15% 2-methyl-1-decene, 110 mg, 0.36 mmol, 1 equiv) as substrates. The dark green-yellow crude oil was subjected to flash column chromatography with silica gel treated with 2% Et_3N (column: 1-inch diameter, filled up to 7-inch height), eluted with pentane (TLC in 100% hexanes, visualized with *p*-anisaldehyde stain) to afford the title compound **42** as a clear oil (53 mg, 58% yield).

Characterization data for (*E*)-vinylic ether **42**:

^{1}H NMR (400 MHz, $CDCl_3$) δ 5.84 (apparent quintet, *J* = 1.3 Hz, 1H), 3.52 (tt, *J* = 8.8, 3.7 Hz, 1H), 1.89 – 1.80 (m, 4H), 1.74 (tt, *J* = 10.7, 4.7 Hz, 2H), 1.59 (d, *J* = 1.4 Hz, 3H), 1.53 – 1.45 (m, 1H), 1.45 – 1.34 (m, 3H), 1.34 – 1.20 (m, 14H), 0.88 (t, *J* = 6.5 Hz, 3H).

^{13}C NMR (101 MHz, $CDCl_3$) δ 139.0, 114.9, 78.6, 34.2, 32.4, 32.1, 29.6, 29.5, 29.3, 28.2, 25.8, 23.8, 22.8, 14.3, 13.1.

HRMS (APCI): *m/z* calcd for $C_{17}H_{33}O^+$ $[M+H]^+$ 253.2526, found 253.2518.

IR (neat): 2926, 2855, 1682, 1059 (weak) cm^{-1}.

(*E*)-vinylic ether 43, from geraniol and (*E*)-1-iodo-2-methyldec-1-ene (**S13**):

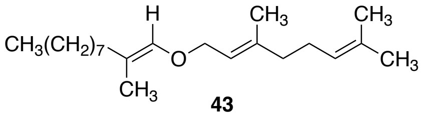

43

Using conditions B, with geraniol (111 mg, 0.72 mmol, 2 equiv) and (*E*)-1-iodo-2-methyldec-1-ene (**S13** : **S14**, 95 : 5 regioisomer ratio, containing 15% 2-methyl-1-decene, 110 mg, 0.36 mmol, 1 equiv) as substrates. *The reaction was for 16 hours before the work-up, to minimize thermal Claisen rearrangement of the allylic vinylic ether product **43**. ^{1}H NMR analysis of the crude mixture showed traces of Claisen rearrangement product (<2%, aldehyde singlets at 9.63 ppm and 10.03 ppm for two diastereomers). The dark green crude oil was subjected to flash column chromatography with silica gel treated with 2% Et_3N (column: 1-inch diameter, filled up to 5-inch height), eluted with 100% pentane (TLC in 100% hexanes, visualized with *p*-anisaldehyde stain) to afford the title compound **43** as a clear oil (77 mg, 70% yield).

Characterization data for (*E*)-vinylic ether **43**:

^{1}H NMR (400 MHz, $CDCl_3$) δ 5.83 (apparent quintet, *J* = 1.3 Hz, 1H), 5.36 (tq, *J* = 6.7, 1.3 Hz, 1H), 5.09 (tdt, *J* = 5.8, 2.9, 1.5 Hz, 1H), 4.21 (dd, *J* = 6.8, 1.1 Hz, 2H), 2.16 – 2.05 (m, 2H), 2.08 – 1.99 (m, 2H), 1.90 – 1.81 (m, 2H), 1.68 (dd, *J* = 3.1, 1.4 Hz, 6H), 1.60 (d, *J* = 1.4 Hz, 3H), 1.59 (d, *J* = 1.4 Hz, 3H), 1.40 – 1.26 (m, 3H), 1.26 (s, 10H), 0.88 (t, *J* = 6.9 Hz, 3H).

^{13}C NMR (101 MHz, $CDCl_3$) δ 140.3, 140.1, 131.8, 124.1, 120.8, 114.7, 68.3, 39.7, 34.2, 32.1, 29.7, 29.5, 29.3, 28.2, 26.5, 25.8, 22.8, 17.8, 16.7, 14.3, 13.1.

HRMS (APCI): *m/z* calcd for $C_{21}H_{39}O^+$ $[M+H]^+$ 307.2995, found 307.2987.

IR (neat): 2923, 2854, 1683, 1008 cm^{-1}.

Scope of (*E*)- and (*Z*)-selective cross-coupling products with vinylic bromides (Figure 3c)

Vinylic ether 44, from 1,2:3,4-di-*O*-isopropylidene-α-D-galactopyranose (**8**) and 1-bromocyclohexene:

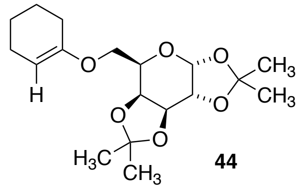

44

Using conditions B, with alcohol **8** (187 mg, 0.72 mmol, 2 equiv) and 1-bromo-1-cyclohexene (58 mg, 0.36 mmol, 1 equiv) as substrates. The dark brown crude oil was subjected to flash column chromatography with silica gel treated with 2% Et_3N (column: 1-inch diameter, filled up to 5-inch height), eluted with 95:5 hexanes/EtOAc (TLC in 10:1 hexanes/EtOAc, visualized with *p*-anisaldehyde stain) to afford the title compound **44** as a clear pale-yellow oil (90 mg, 74% yield).

Characterization data for vinylic ether **44**:

^{1}H NMR (400 MHz, $CDCl_3$) δ 5.54 (d, *J* = 5.0 Hz, 1H), 4.65 (t, *J* = 3.9 Hz, 1H), 4.61 (dd, *J* = 7.9, 2.4 Hz, 1H), 4.31 (dd, *J* = 5.0, 2.4 Hz, 1H), 4.29 (dd, *J* = 8.0, 1.9 Hz, 1H), 4.06 (td, *J* = 6.3, 1.9 Hz, 1H), 3.86 (dd, *J* = 10.0, 6.2 Hz, 1H), 3.80 (dd, *J* = 10.0, 6.4 Hz, 1H), 2.05 (dtdd, *J* = 9.9, 6.0, 3.7, 1.8 Hz, 4H), 1.68 – 1.61 (m, 2H), 1.57 – 1.47 (m, 5H), 1.45 (s, 3H), 1.34 (s, 3H), 1.33 (s, 3H).

^{13}C NMR (151 MHz, $CDCl_3$) δ 154.4, 109.5, 108.8, 96.5, 94.5, 71.3, 70.8, 70.8, 66.2, 64.9, 27.9, 26.2, 26.1, 25.1, 24.6, 23.6, 23.0, 22.8.

HRMS (APCI): *m/z* calcd for $C_{18}H_{28}O_6^+$ $[M+H]^+$ 341.1959, found 341.1949.

IR (neat): 2927, 2856, 1667, 1210, 1070 cm^{-1}.

$[\alpha]^{22}_D$ -94.0 (c = 0.1, $CHCl_3$).

Vinylic ether 45, from geraniol and 1-bromocyclohexene:

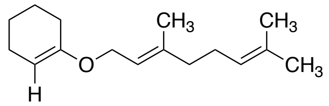

45

Using conditions B, with geraniol (111 mg, 0.72 mmol, 2 equiv) and 1-bromo-1-cyclohexene (58 mg, 0.36 mmol, 1 equiv) as substrates. *The reaction was run for 16 h before work-up, to avoid thermal Claisen rearrangement of the allylic vinylic ether product **45**. ^{1}H NMR analysis of the crude mixture showed no evidence of Claisen rearrangement product (no aldehyde resonances between 9 - 10 ppm). The dark green crude oil was subjected to flash column chromatography with silica gel treated with 2% Et_3N (column: 1-inch diameter, filled up to 5-inch height), eluted with hexanes (TLC in 100% hexanes, visualized with *p*-anisaldehyde stain) to afford the title compound **45** as a clear pale-yellow oil (53 mg, 63% yield).

Characterization data for vinylic ether **45**:

^{1}H NMR (400 MHz, $CDCl_3$) δ 5.40 (tq, *J* = 6.6, 1.3 Hz, 1H), 5.10 (doublet of doublet of quintets, *J* = 7.0, 5.7, 1.4 Hz, 1H), 4.63 (td, *J* = 3.3, 1.9 Hz, 1H), 4.19 (d, *J* = 6.6 Hz, 2H), 2.17 – 2.00 (m, 8H), 1.73 – 1.60 (m, 8H), 1.60 (d, *J* = 1.3 Hz, 3H), 1.55 (ddt, *J* = 8.5, 6.1, 2.7 Hz, 2H).

^{13}C NMR (101 MHz, $CDCl_3$) δ 154.7, 140.3, 131.8, 124.1, 120.2, 94.0, 63.4, 39.7, 28.1, 26.5, 25.9, 23.7, 23.1, 22.9, 17.8, 16.7.

HRMS (APCI): *m/z* calcd for $C_{16}H_{27}O^+$ $[M+H]^+$ 235.2056, found 235.2049.

IR (neat): 2923, 2857, 2842, 1664, 1016 cm^{-1}.

Vinylic ether 46, from geraniol and 1-bromo-2-methyl-1-propene:

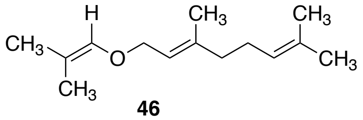

46

Using conditions B, with geraniol (308 mg, 2.0 mmol, 2 equiv) and 1-bromo-2-methyl-1-propene (135 mg, 1.0 mmol, 1 equiv) as substrates. The reaction was conducted for 24 h, to minimize thermal Claisen rearrangement of the allylic vinylic ether product **46**. ^{1}H NMR analysis of the crude mixture showed traces of Claisen rearrangement product (<2%, aldehyde singlets at 9.66 ppm and 10.00 ppm for two diastereomers, respectively). The dark green crude oil was subjected to flash column chromatography with silica gel treated with 2% Et_3N (column: 1-inch diameter, filled up to 5-inch height), eluted with 100:0 to 99:1 hexanes/diethyl ether (TLC in 100% hexanes, visualized with *p*-anisaldehyde stain) to afford the title compound **46** as a clear oil (92 mg, 44%).

Characterization data for vinylic ether **46**:

^{1}H NMR (400 MHz, $CDCl_3$) δ 5.84 (septet, *J* = 1.4 Hz, 1H), 5.38 (tq, *J* = 6.7, 1.3 Hz, 1H), 5.11 (doublet of doublet of quintets, *J* = 7.0, 5.8, 1.5 Hz, 1H), 4.23 (dq, *J* = 6.7, 0.8 Hz, 2H), 2.17 – 2.09 (m, 2H), 2.09 – 2.03 (m, 2H), 1.71 (d, *J* = 1.3 Hz, 3H), 1.70 – 1.69 (m, 3H), 1.64 (d, *J* = 1.4 Hz, 3H), 1.63 (d, *J* = 1.3 Hz, 3H), 1.57 (d, *J* = 1.3 Hz, 3H).

^{13}C NMR (101 MHz, $CDCl_3$) δ 140.4, 139.8, 131.8, 124.0, 120.7, 110.6, 68.2, 39.7, 26.5, 25.8, 19.8, 17.8, 16.6, 15.2.

HRMS (APCI): *m/z* calcd for $C_{14}H_{25}O^+$ $[M+H]^+$ 209.1900, found 209.1899.

IR (neat): 2917, 2856, 1691, 1045 (weak) cm^{-1}.

References

(1) Nordmann, G.; Buchwald, S. L. A Domino Copper-Catalyzed C−O Coupling−Claisen Rearrangement Process. *J. Am. Chem. Soc.* **2003**, *125* (17), 4978–4979. https://doi.org/10.1021/ja034809y

(2) Zhao, Y.; Snieckus, V. A Practical in Situ Generation of the Schwartz Reagent. Reduction of Tertiary Amides to Aldehydes and Hydrozirconation. *Org. Lett.* **2014**, *16* (2), 390–393. https://doi.org/10.1021/ol403183a

(3) Pradal, A.; Evano, G. A Vinylic Rosenmund–von Braun Reaction: Practical Synthesis of Acrylonitriles. *Chem. Commun.* **2014**, *50* (80), 11907–11910. https://doi.org/10.1039/C4CC05557H

(4) Skotnitzki, J.; Kremsmair, A.; Keefer, D.; Gong, Y.; de Vivie-Riedle, R.; Knochel, P. Stereoselective Csp3−Csp2 Cross-Couplings of Chiral Secondary Alkylzinc Reagents with Alkenyl and Aryl Halides. *Angew. Chem. Int. Ed.* **2020**, *59* (1), 320–324. https://doi.org/10.1002/anie.201910397

(5) Wu, Y.-D.; Lai, Y.; Dai, W.-M. Synthesis of Two Diastereomeric C1–C7 Acid Fragments of Amphidinolactone B Using B-Alkyl Suzuki–Miyaura Cross-Coupling as the Modular Assembly Step. *ChemistrySelect* **2016**, *1* (5), 1022–1027. https://doi.org/10.1002/slct.201600315

(6) Pietruszka, J.; Witt, A. Synthesis of the Bestmann-Ohira Reagent. *Synthesis* **2006**, *2006* (24), 4266–4268. https://doi.org/10.1055/s-2006-950307

(7) Mames, A.; Stecko, S.; Mikołajczyk, P.; Soluch, M.; Furman, B.; Chmielewski, M. Direct, Catalytic Synthesis of Carbapenams via Cycloaddition/Rearrangement Cascade Reaction: Unexpected Acetylenes' Structure Effect. *J. Org. Chem.* **2010**, *75* (22), 7580–7587. https://doi.org/10.1021/jo101355h

(8) Nitelet, A.; Evano, G. A General Copper-Catalyzed Vinylic Halogen Exchange Reaction. *Org. Lett.* **2016**, *18* (8), 1904–1907. https://doi.org/10.1021/acs.orglett.6b00678

(9) Stork, G.; Zhao, K. A Stereoselective Synthesis of (*Z*)-1-Iodo-1-Alkenes. *Tetrahedron Lett.* **1989**, *30* (17), 2173–2174. https://doi.org/10.1016/S0040-4039(00)99640-0

(10) Kadota, I.; Ueno, H.; Ohno, A.; Yamamoto, Y. A Simple and practical method for the stereoselective synthesis of (*Z*)-1-iodo-1-alkenes from 1,1-diiodo-1-alkenes. *Tetrahedron Lett.* **2003**, *44* (48), 8645-8647. https://doi.org/10.1016/j.tetlet.2003.09.166

(11) Beshai, M.; Dhudshia, B.; Mills, R.; Thadani, A. N. Terminal Alkynes from Aldehydes via Dehydrohalogenation of (*Z*)-1-Iodo-1-Alkenes with TBAF. *Tetrahedron Lett.* **2008**, *49* (48), 6794–6796. https://doi.org/10.1016/j.tetlet.2008.09.060

(12) Kang, S. H.; Lee, Y. M. A Formal Total Synthesis of (-)-Dysiherbaine. *Synlett* **2003** (7), 993-994. https://doi.org/10.1055/s-2003-39318

(13) Smithers, L.; van Dalsen, L.; Boland, C.; Reid, G.; Weichert, D.; Caffrey, M. Effects of 2-Monoacylglycerol on in Meso Crystallization and the Crystal Structures of Integral Membrane Proteins. *Cryst. Growth Des.* **2020**, *20* (8), 5444–5454. https://doi.org/10.1021/acs.cgd.0c00660

(14) Nicolaou, K. C.; Bellavance, G.; Buchman, M.; Pulukuri, K. K. Total Syntheses of Disorazoles A_1 and B_1 and Full Structural Elucidation of Disorazole B_1. *J. Am. Chem. Soc.* **2017**, *139* (44), 15636-15639. https://doi.org/10.1021/jacs.7b09843

(15) Negishi, E.-i.; Van Horn, D. E.; Yoshida, T. Carbometalation Reaction of Alkynes with Organoalane-Zirconocene Derivatives as a Route to Stereo- and Regiodefined Trisubstituted Alkenes. *J. Am. Chem. Soc.* **1985**, *107* (23), 6639-6647. https://doi.org/10.1021/ja00309a036

(16) Wipf, P.; Kendall, C.; Stephenson, C. R. J. Dimethylzinc-Mediated Additions of Alkenylzirconocenes to Aldimines. New Methodologies for Allylic Amine and *C*-Cyclopropylalkylamine Syntheses. *J. Am. Chem. Soc.* **2003**, *125* (3), 761-768. https://doi.org/10.1021/ja028092a

(17) Davoust, M.; Cantagrel, F.; Metzner, P.; Brière, J.-F. A stereodivergent synthesis of β-hydroxy-α–methylene lactones *via* vinyl epoxides. *Org. Biomol. Chem.* **2008**, *6*, 1981-1993. https://doi.org/10.1039/B802310G

(18) Temple, K. J.; Wright, E. N.; Fierke, C. A.; Gibbs, R. A. Exploration of GGTase-I substrate requirements. Part 2: Synthesis and biochemical analysis of novel saturated geranylgeranyl diphosphate analogs. *Bioorg. Med. Chem. Lett.* **2016**, *26* (15), 3503-3507. https://doi.org/10.1016/j.bmcl.2016.06.035

(19) Yang, C.; Nolan, S. P. Regio- and Stereoselective Dimerization of Terminal Alkynes to Enynes Catalyzed by a Palladium/Imidazolium System. *J. Org. Chem.* **2002**, *67* (2), 591–593. https://doi.org/10.1021/jo010837s

(20) Cai, Q.; Zou, B.; Ma, D. Mild Ullmann-Type Biaryl Ether Formation Reaction by Combination Of ortho-Substituent and Ligand Effects. *Angew. Chem. Int. Ed.* **2006**, *45* (8), 1276–1279. https://doi.org/10.1002/anie.200503538

(21) Surry, D. S.; Buchwald, S. L. Diamine Ligands in Copper-Catalyzed Reactions. *Chem. Sci.* **2010**, *1* (1), 13-31. https://doi.org/10.1039/c0sc00107d

(22) Jiang, L.; Job, G. E.; Klapars, A.; Buchwald, S. L. Copper-Catalyzed Coupling of Amides and Carbamates with Vinyl Halides. *Org. Lett.* **2003**, *5* (20), 3667–3669. https://doi.org/10.1021/ol035355c

(23) Cai, J.; Wang, Z.-K.; Usman, M.; Lu, Z.-W.; Hu, X.-D.; Liu, W.-B. Enantioselective Synthesis of β-Quaternary Carbon-Containing Chromanes and 3,4-Dihydropyrans via Cu-Catalyzed Intramolecular C–O Bond Formation. *Org. Lett.* **2019**, *21* (21), 8852–8856. https://doi.org/10.1021/acs.orglett.9b03549

(24) Chen, Z.; Jiang, Y.; Zhang, L.; Guo, Y.; Ma, D. Oxalic Diamides and *Tert* -Butoxide: Two Types of Ligands Enabling Practical Access to Alkyl Aryl Ethers via Cu-Catalyzed Coupling Reaction. *J. Am. Chem. Soc.* **2019**, *141* (8), 3541–3549. https://doi.org/10.1021/jacs.8b12142

(25) Tlili, A.; Monnier, F.; Taillefer, M. Selective One-Pot Access to Symmetrical or Unsymmetrical Diaryl Ethers by Copper-Catalyzed Double Arylation of a Simple Oxygen Source. *Chem. Eur. J.* **2010**, *16* (41), 12299–12302. https://doi.org/10.1002/chem.201001373

(26) Buck, E.; Song, Z. J.; Tschaen, D.; Dormer, P. G.; Volante, R. P.; Reider, P. J. Ullmann Diaryl Ether Synthesis: Rate Acceleration by 2,2,6,6-Tetramethylheptane-3,5-Dione. *Org. Lett.* **2002**, *4* (9), 1623–1626. https://doi.org/10.1021/ol025839t

(27) Ma, D.; Cai, Q. Copper/Amino Acid Catalyzed Cross-Couplings of Aryl and Vinyl Halides with Nucleophiles. *Acc. Chem. Res.* **2008**, *41* (11), 1450–1460. https://doi.org/10.1021/ar8000298

(28) Zhang, H.; Cai, Q.; Ma, D. Amino Acid Promoted CuI-Catalyzed C−N Bond Formation between Aryl Halides and Amines or *N*-Containing Heterocycles. *J. Org. Chem.* **2005**, *70* (13), 5164–5173. https://doi.org/10.1021/jo0504464

(29) Zhu, W.; Ma, D. Synthesis of Aryl Azides and Vinyl Azides via Proline-Promoted CuI-Catalyzed Coupling Reactions. *Chem. Commun.* **2004** (7), 888–889. https://doi.org/10.1039/B400878B

(30) Zhang, H.; Cao, W.; Ma, D. L-Proline-Promoted CuI-Catalyzed C-S Bond Formation between Aryl Iodides and Thiols. *Synth. Commun.* **2007**, *37* (1), 25–35. https://doi.org/10.1080/00397910600977533

(31) Lo, Q. A.; Sale, D.; Braddock, D. C.; Davies, R. P. Mechanistic and Performance Studies on the Ligand-Promoted Ullmann Amination Reaction. *ACS Catal.* **2018**, *8* (1), 101-109. https://doi.org/10.1021/acscatal.7b03664

(32) Ouali, A.; Spindler, J.-F.; Jutand, A.; Taillefer, M. "Nitrogen Ligands in Copper-Catalyzed Arylation of Phenols: Structure/Activity Relationships and Applications." *Adv. Synth. Catal.* **2007**, *349* (11-12), 1906-1916. https://doi.org/10.1002/adsc.200600628

(33) Kim, B.; Chinn, A. J.; Fandrick, D. R.; Senanayake, C. H.; Singer, R. A.; Miller, S. J. "Distal Stereocontrol Using Guanidinylated Peptides as Multifunctional Ligands: Desymmetrization of Diarylmethanes via Ullman Cross-Coupling." *J. Am. Chem. Soc.* **2016**, *138* (25), 7939-7945. https://doi.org/10.1021/jacs.6b03444

(34) Moreau, C.; Kirchberger, T.; Swarbrick, J. M.; Bartlett, S. J.; Fliegert, R.; Yorgan, T.; Bauche, A.; Harneit, A.; Guse, A. H.; Potter, B. V. L. Structure–Activity Relationship of Adenosine 5′-Diphosphoribose at the Transient Receptor Potential Melastatin 2 (TRPM2) Channel: Rational Design of Antagonists. *J. Med. Chem.* **2013**, *56* (24), 10079–10102. https://doi.org/10.1021/jm401497a
