# Supplementary material for: Stereospecific Cu(I)-Catalyzed C–O Cross-Coupling Synthesis of Acyclic 1,2-Di- and Trisubstituted Vinylic Ethers from Alcohols and Vinylic Halides
Source: Org Lett. 2023 Jul 12;25(28):5297–301. doi: 10.1021/acs.orglett.3c01849 (PMC10367064; doi:10.1021/acs.orglett.3c01849)

Supporting Information

Stereospecific Cu(I)-catalyzed C-O cross-coupling synthesis of acyclic 1,2-di- and trisubstituted vinylic ethers from alcohols and vinylic halides

San L. Pham, Taehee Kim, and Frank E. McDonald*

Department of Chemistry, Emory University, 1515 Dickey Drive NE, Atlanta GA 30322 USA

*Corresponding author: fmcdona@emory.edu

Part 2: ^{1}H and ^{13}C NMR spectra for new compounds

^{1}H NMR spectrum of **7** (400 MHz, $CDCl_3$)

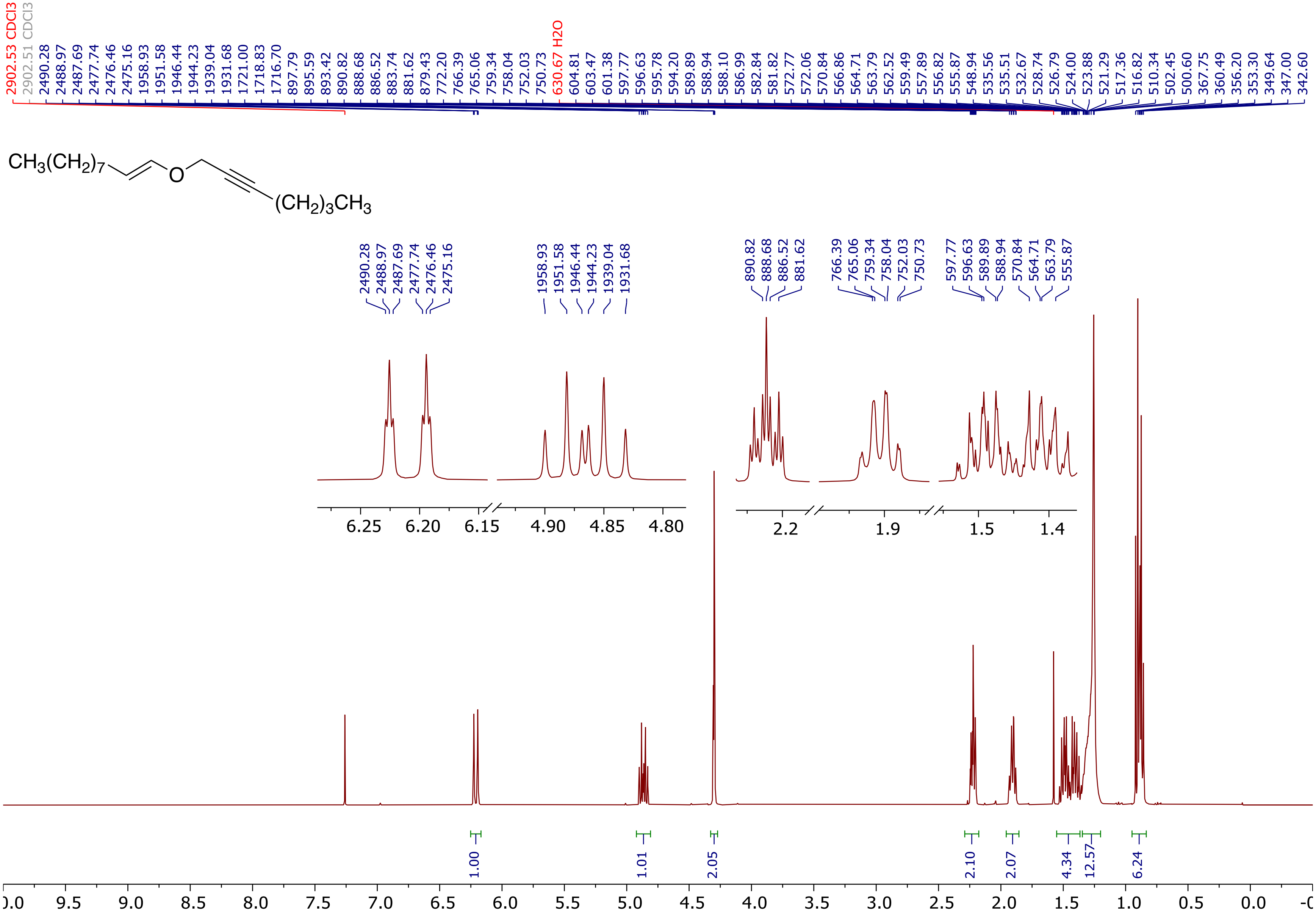

^{13}C NMR spectrum of **7** (101 MHz, $CDCl_3$)

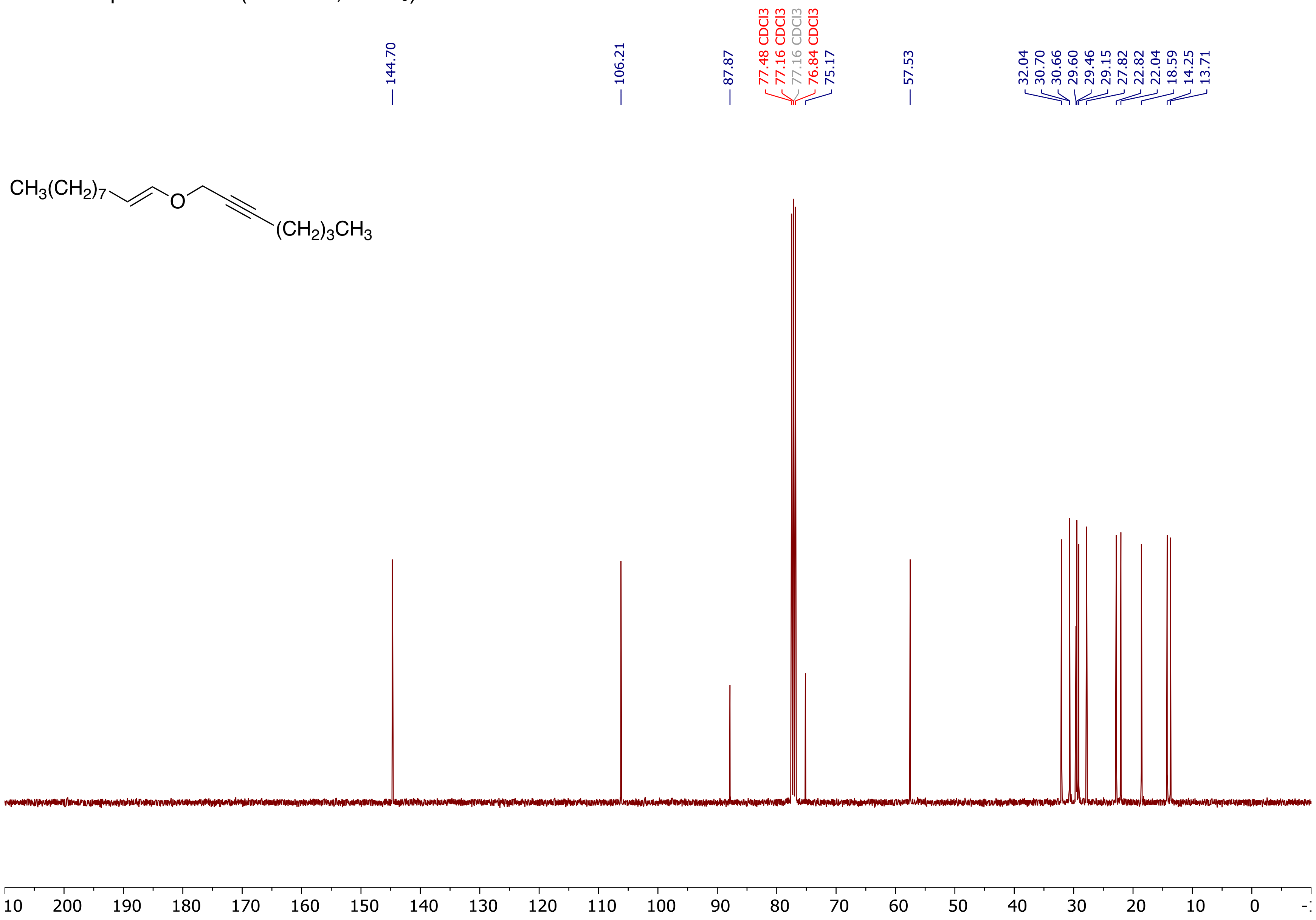

^{1}H NMR spectrum of **9** (400 MHz, $CDCl_3$)

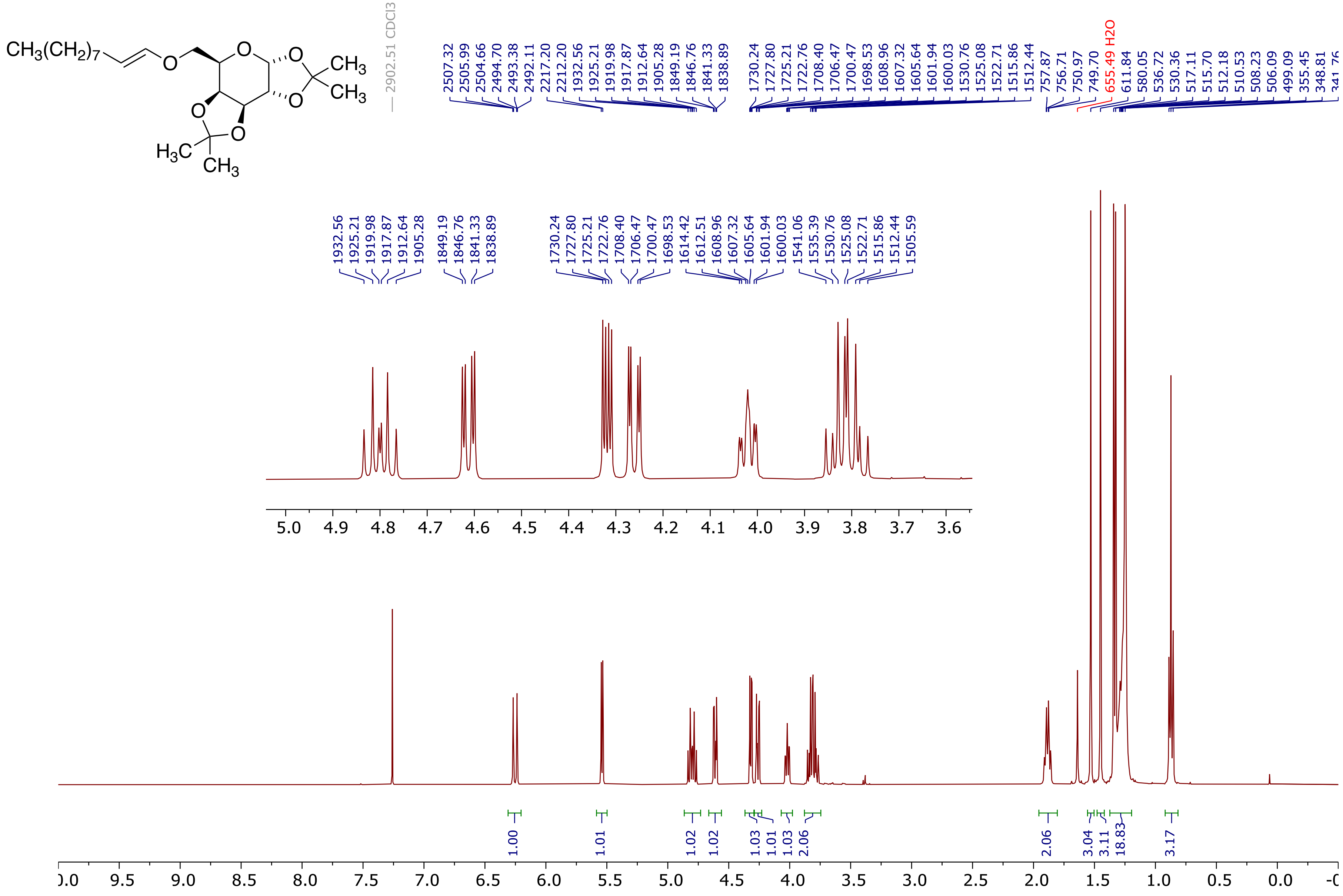

^{13}C NMR spectrum of **9** (101 MHz, $CDCl_3$)

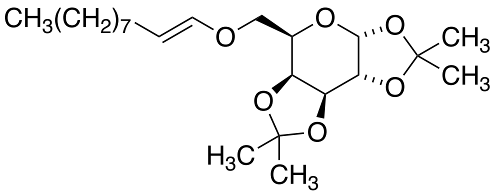

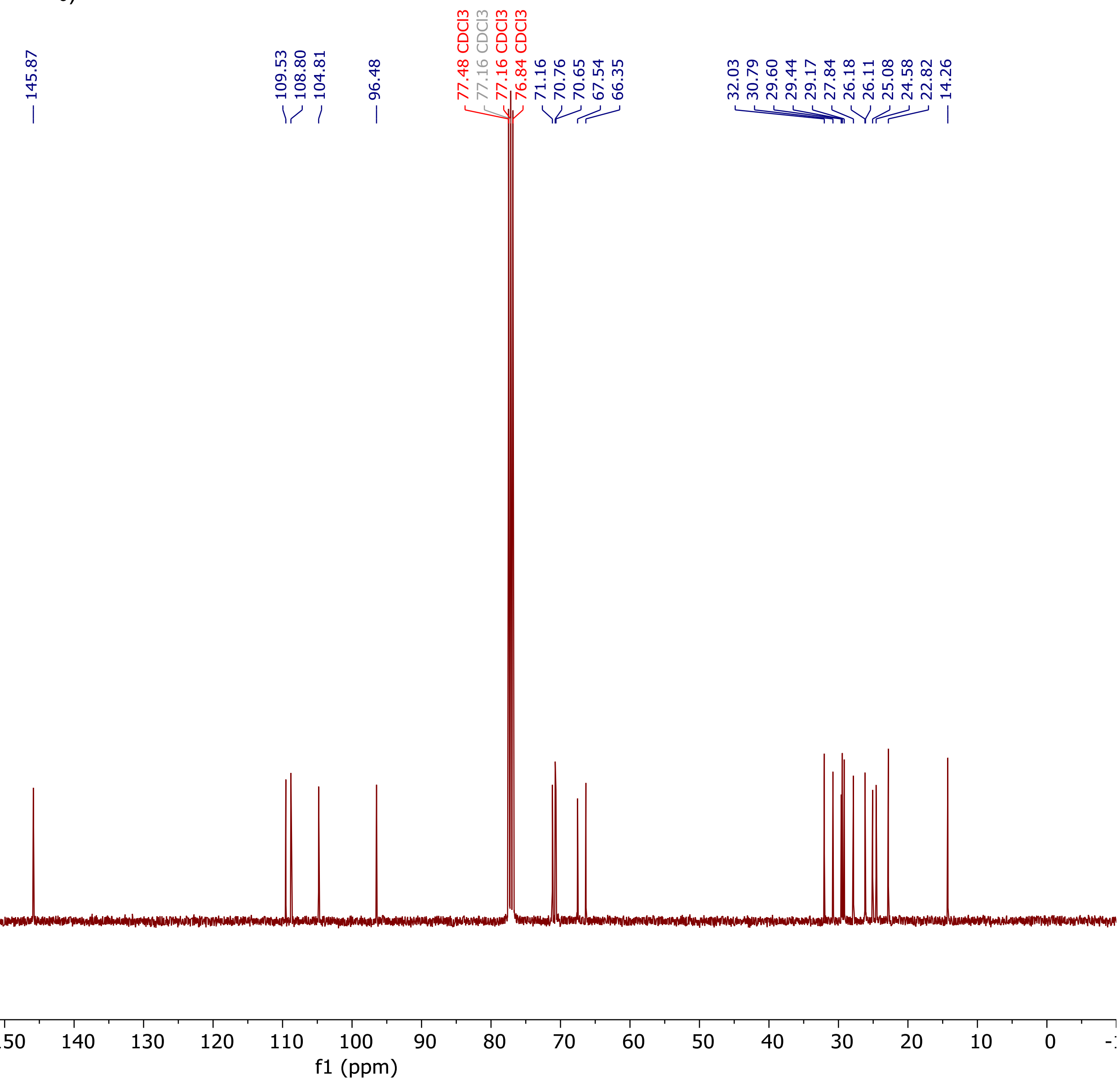

COSY spectrum of **9** (600 MHz, $CDCl_3$)

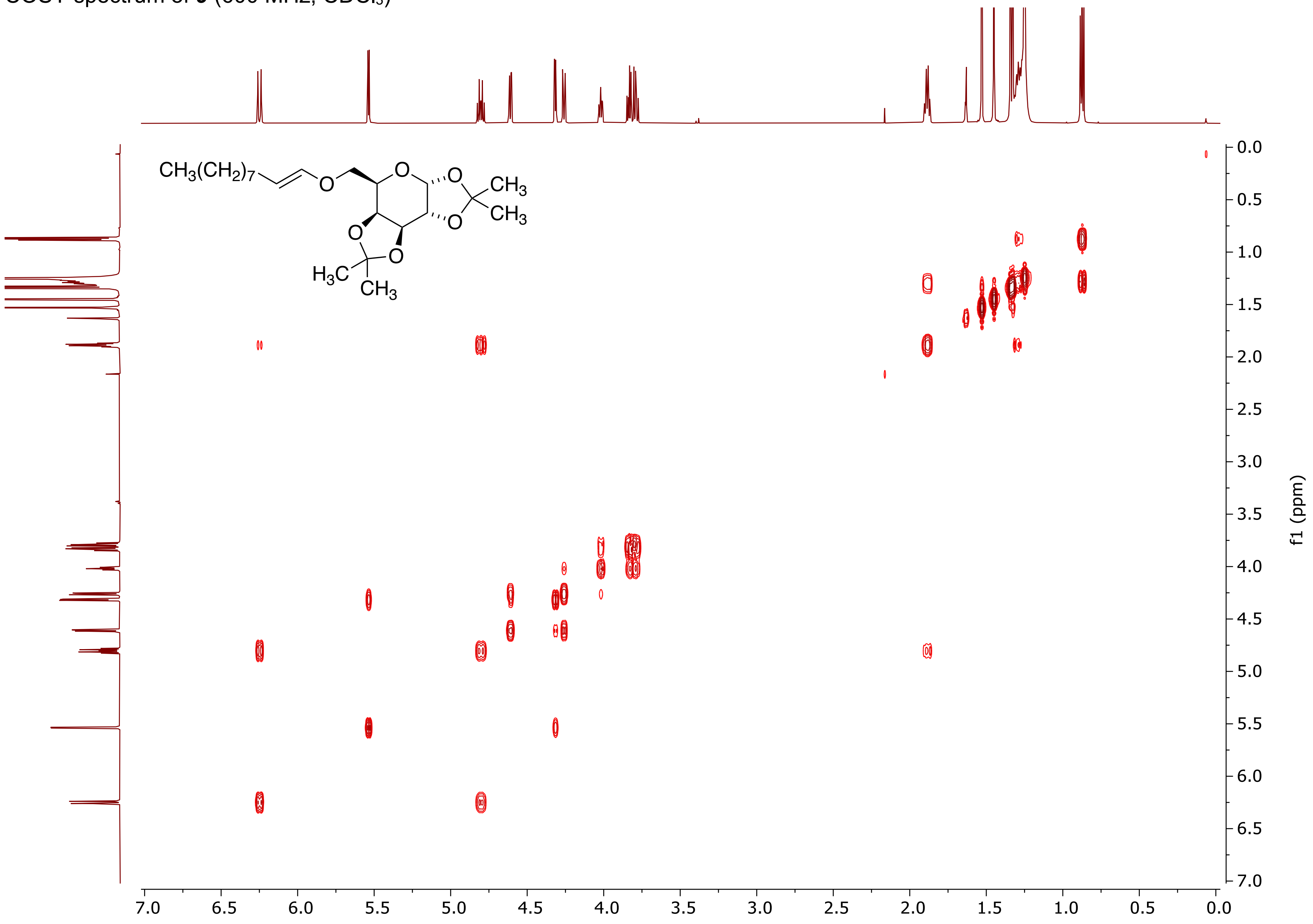

COSY spectrum of **9** (600 MHz, $CDCl_3$) expansion

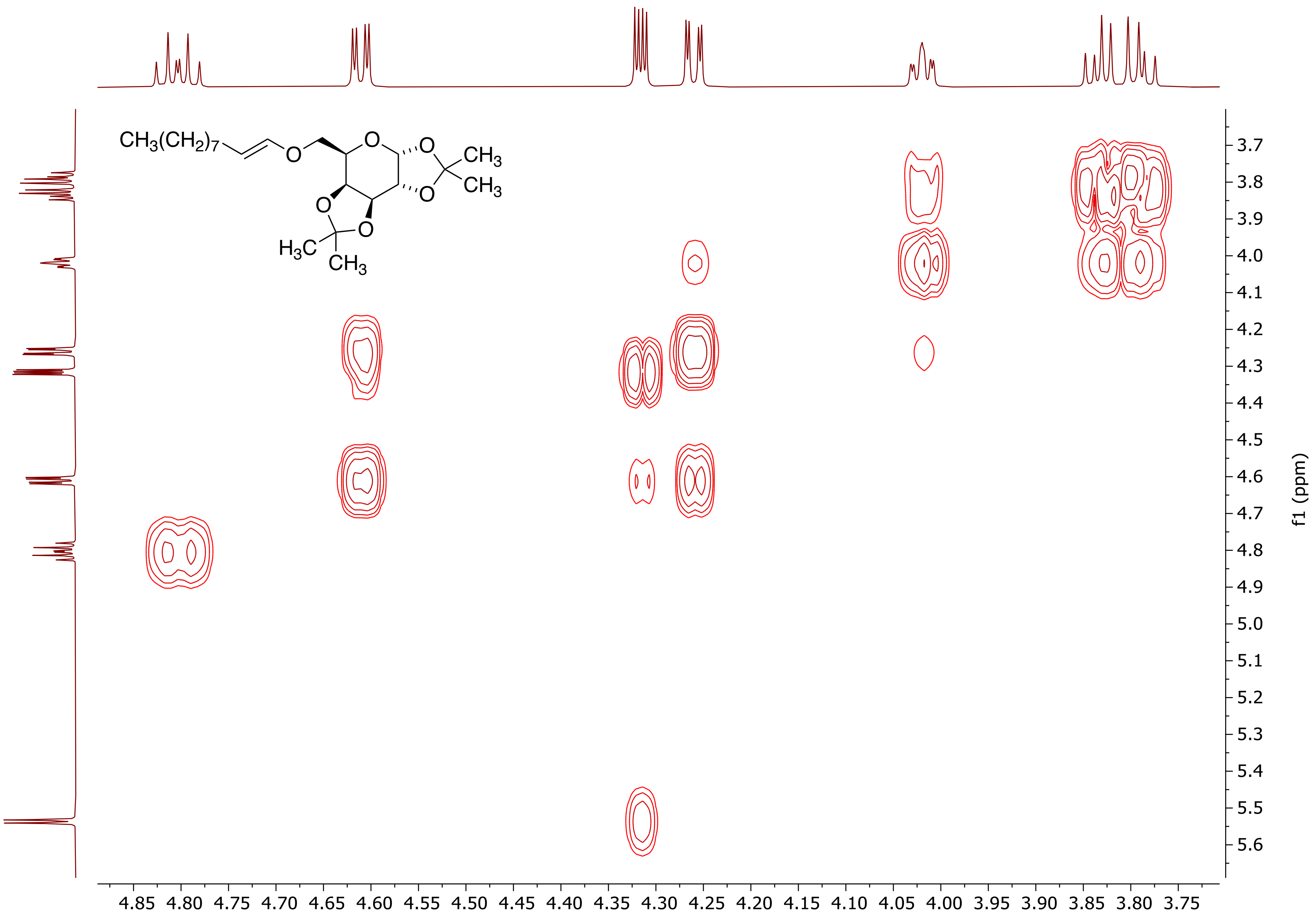

HSQC spectrum of **9** (600 MHz, $CDCl_3$)

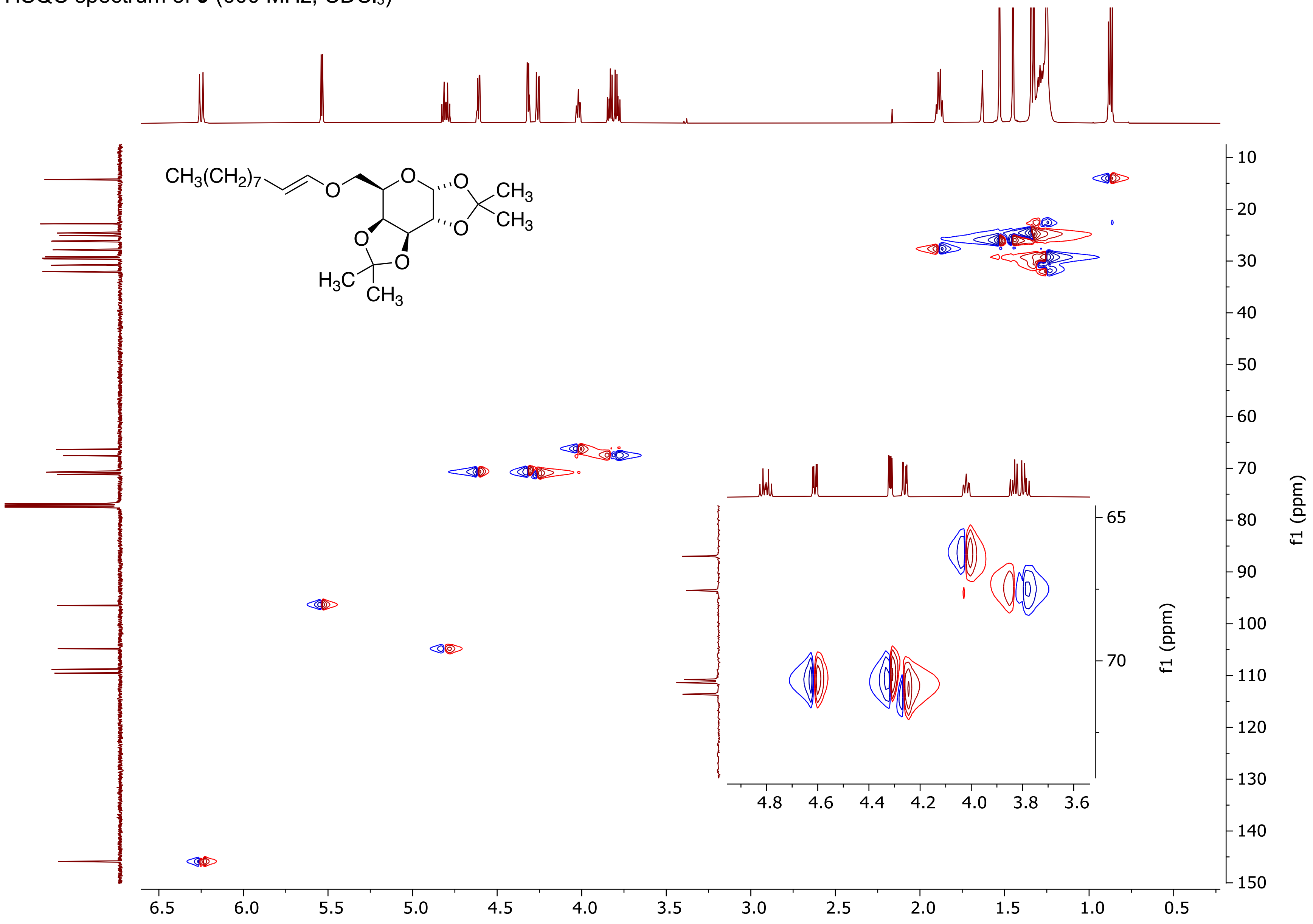

^{1}H NMR spectrum of **12** (17:1 Z/E, 800 MHz, $CDCl_3$)

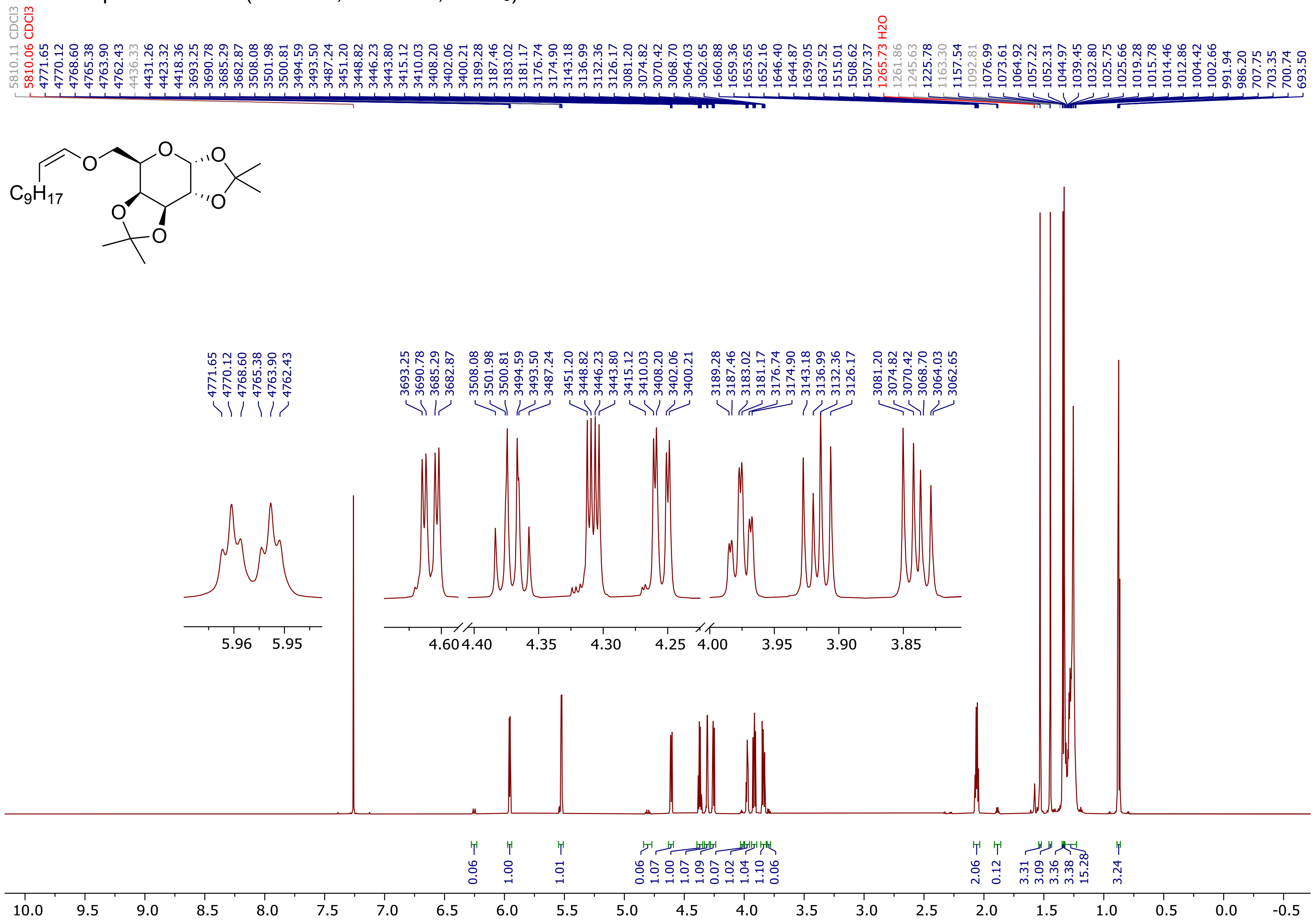

^{13}C NMR spectrum of **12** (17:1 Z/E, 201 MHz, CDCl$_3$)

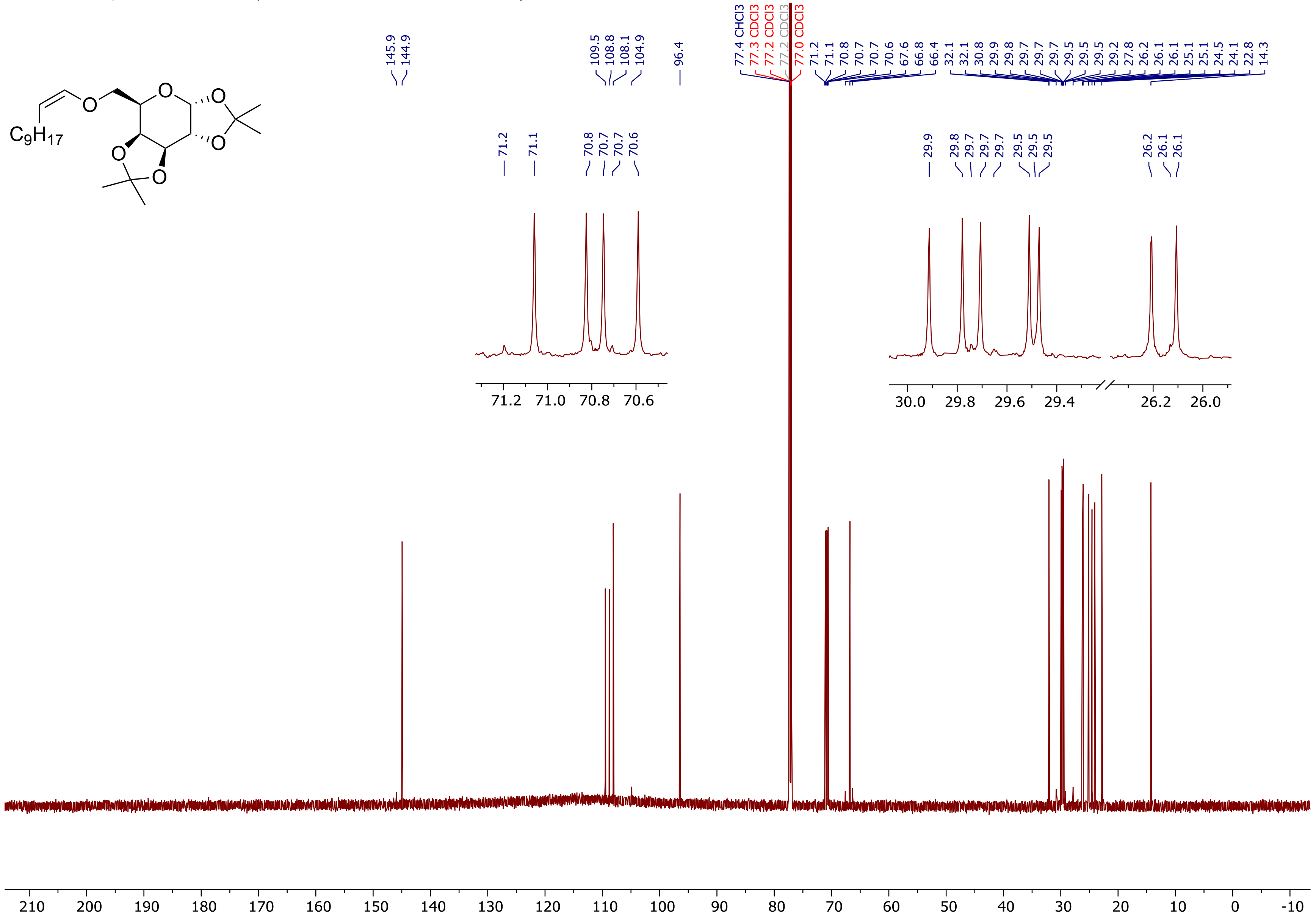

COSY spectrum of **12** (17:1 Z/E, 800 MHz, $CDCl_3$)

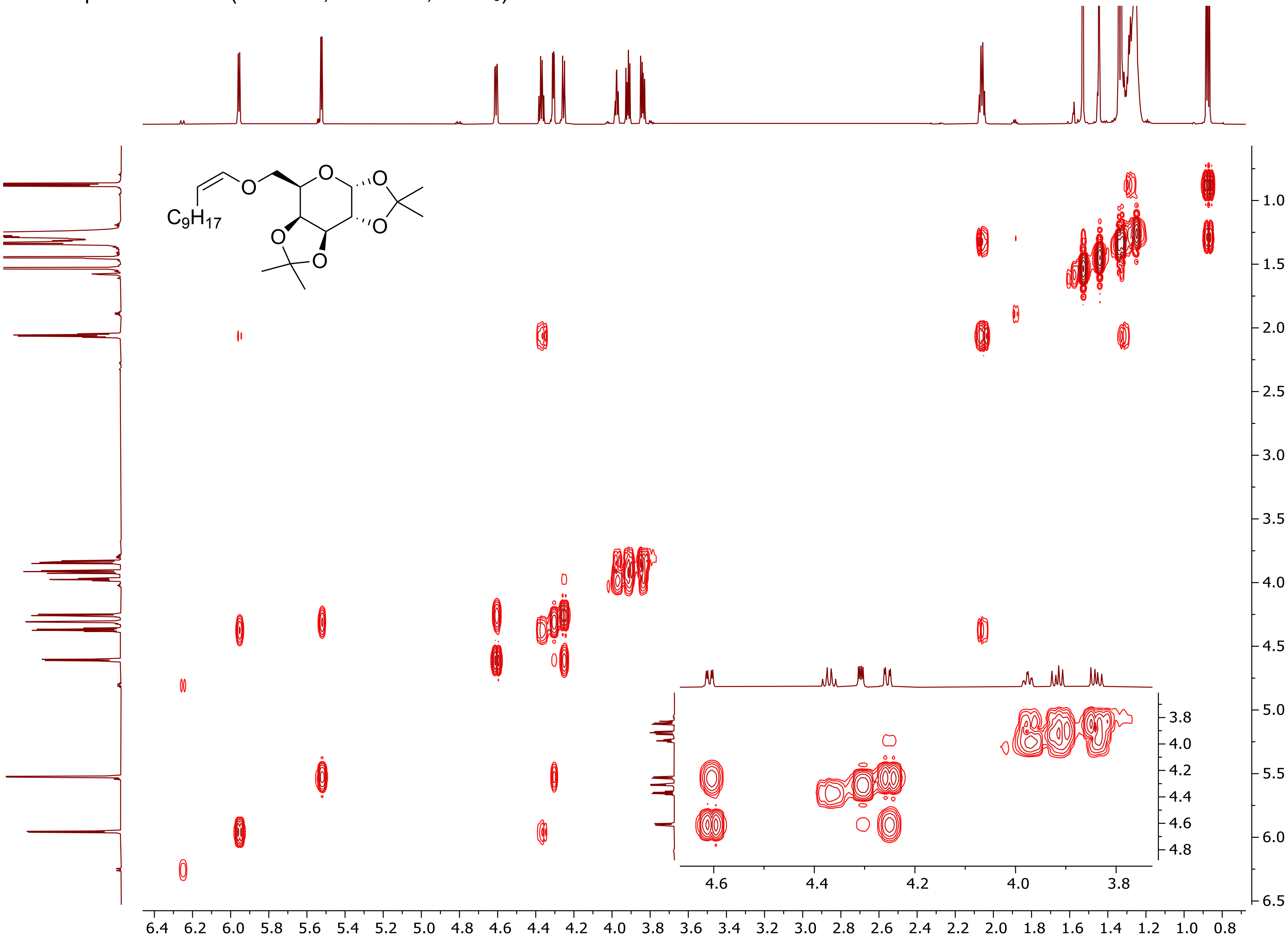

HSQC spectrum of **12** (17:1 Z/E, 800 MHz, $CDCl_3$)

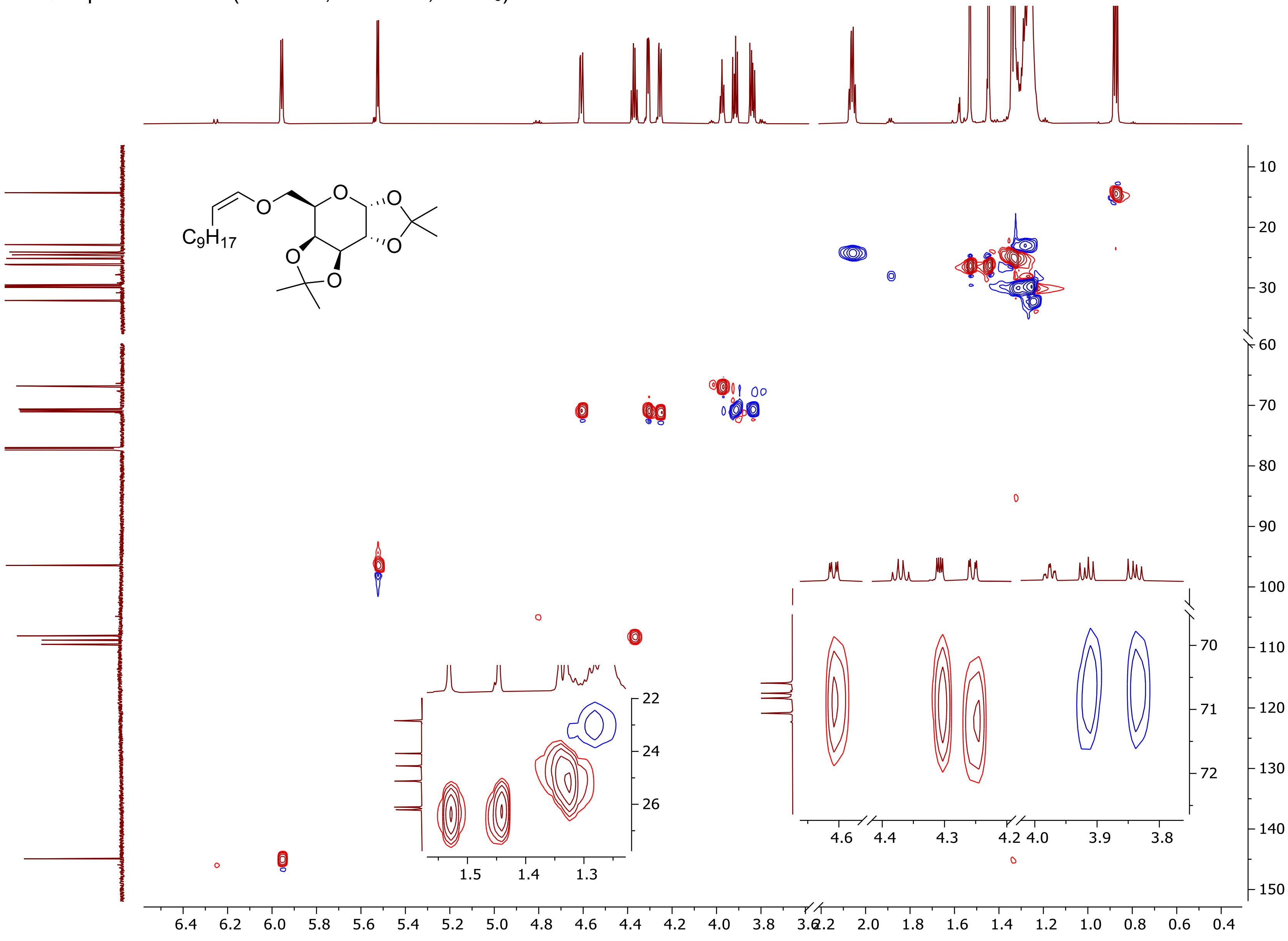

^{1}H NMR spectrum of enyne **13** (400 MHz, $CDCl_3$)

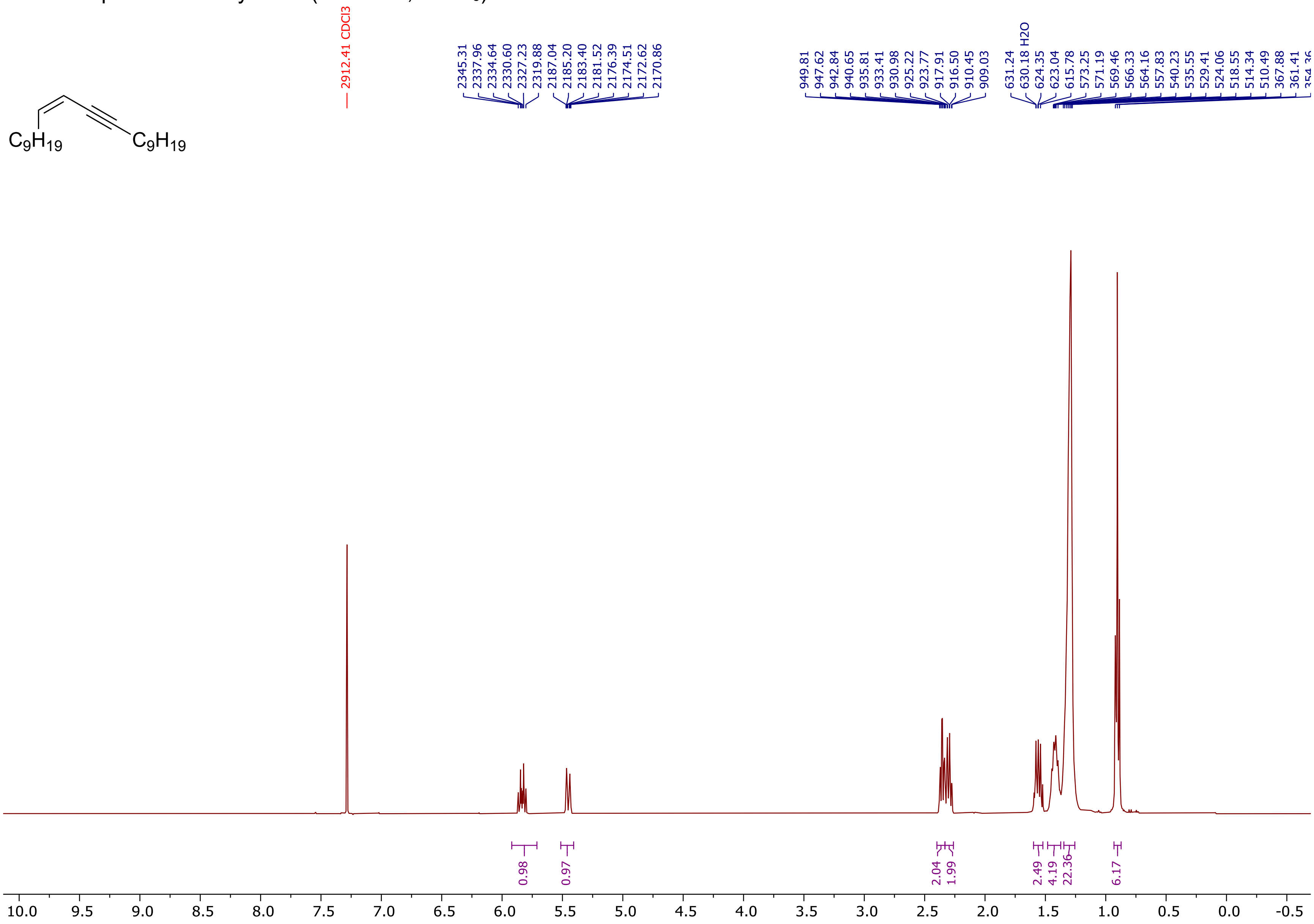

^{13}C NMR spectrum of enyne **13** (101 MHz, $CDCl_3$)

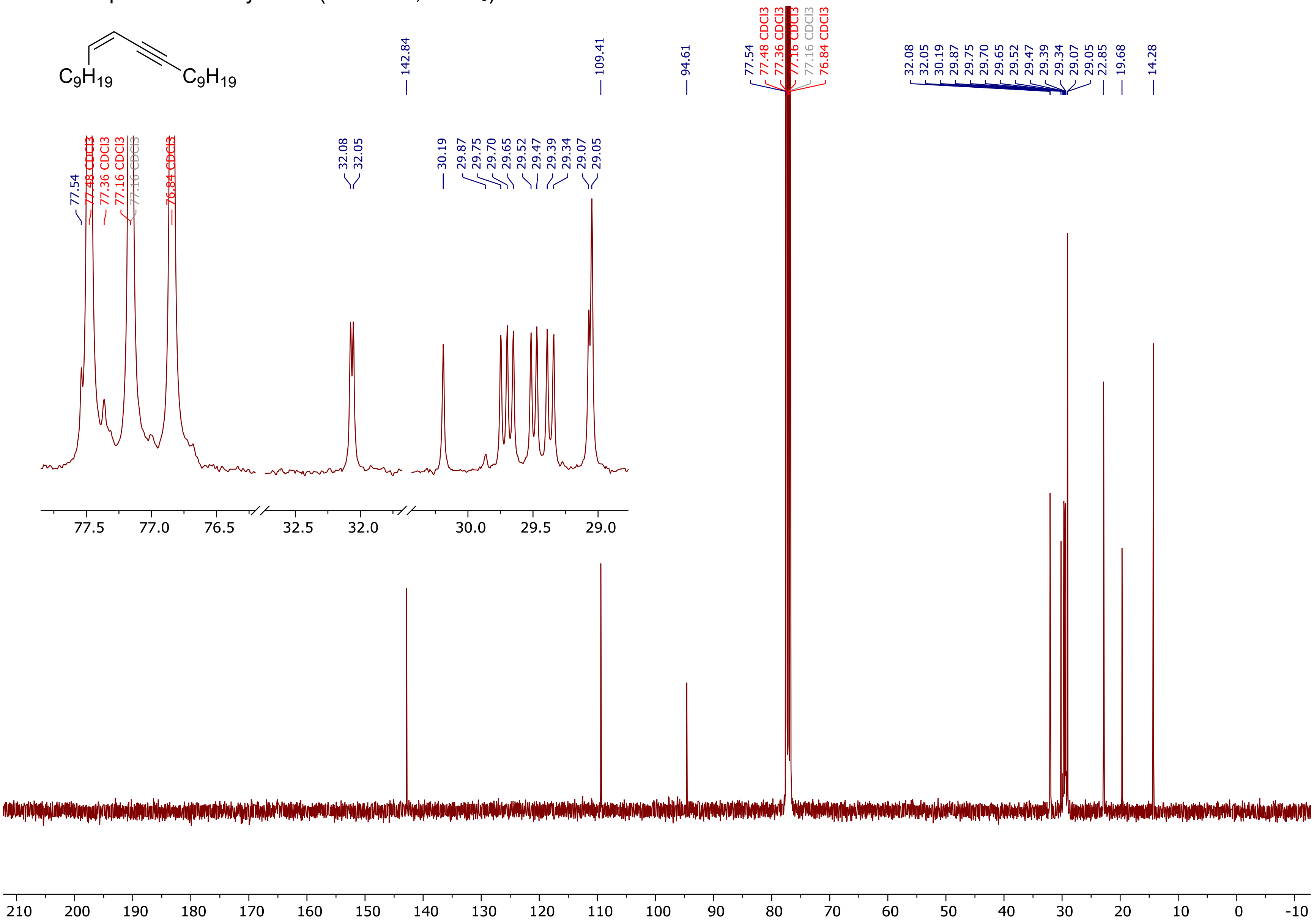

^{1}H NMR spectrum of **14** (>20:1 Z/E, 400 MHz, $CDCl_3$)

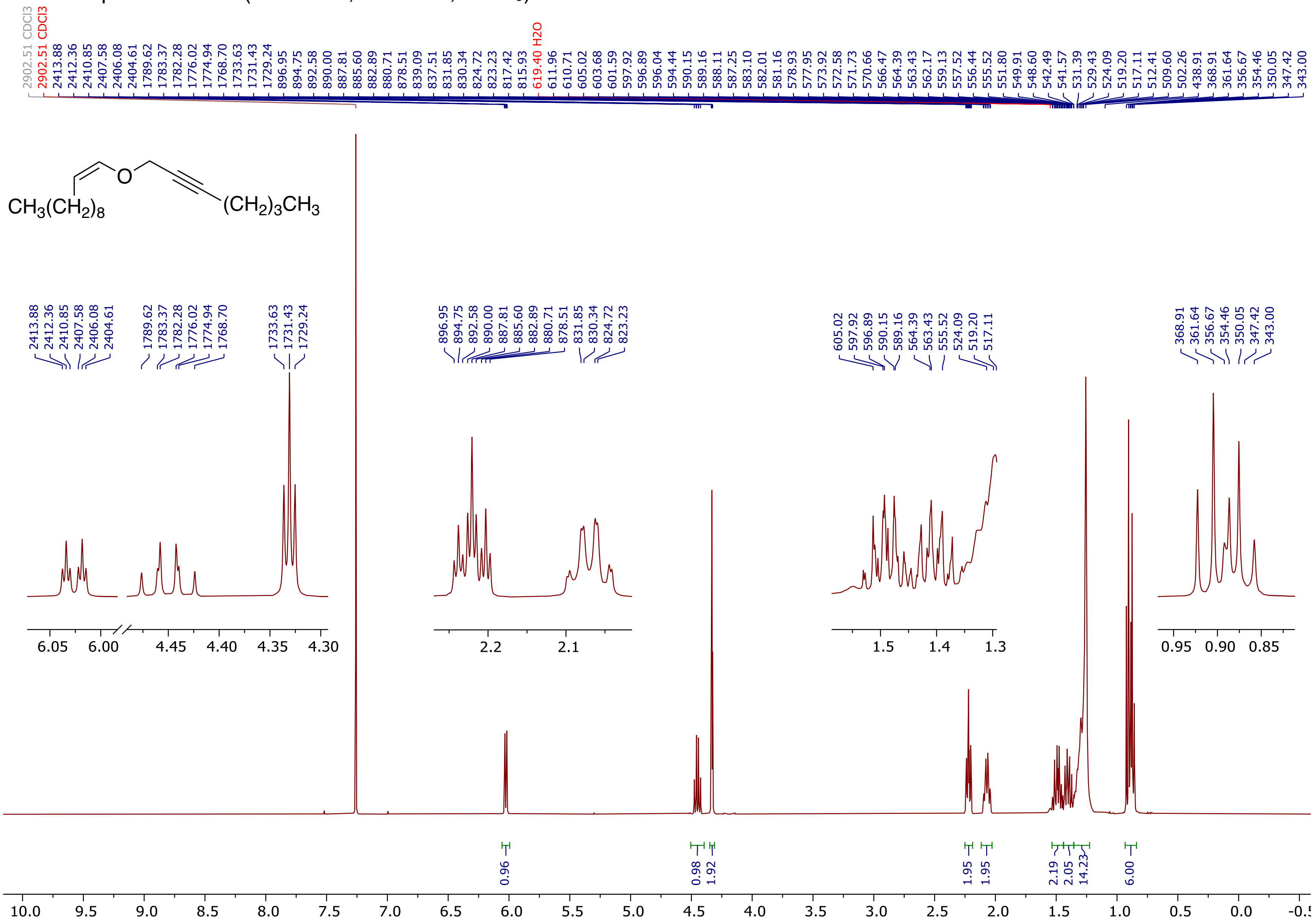

^{13}C NMR spectrum of **14** (>20:1 Z/E, 101 MHz, $CDCl_3$)

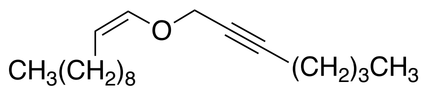

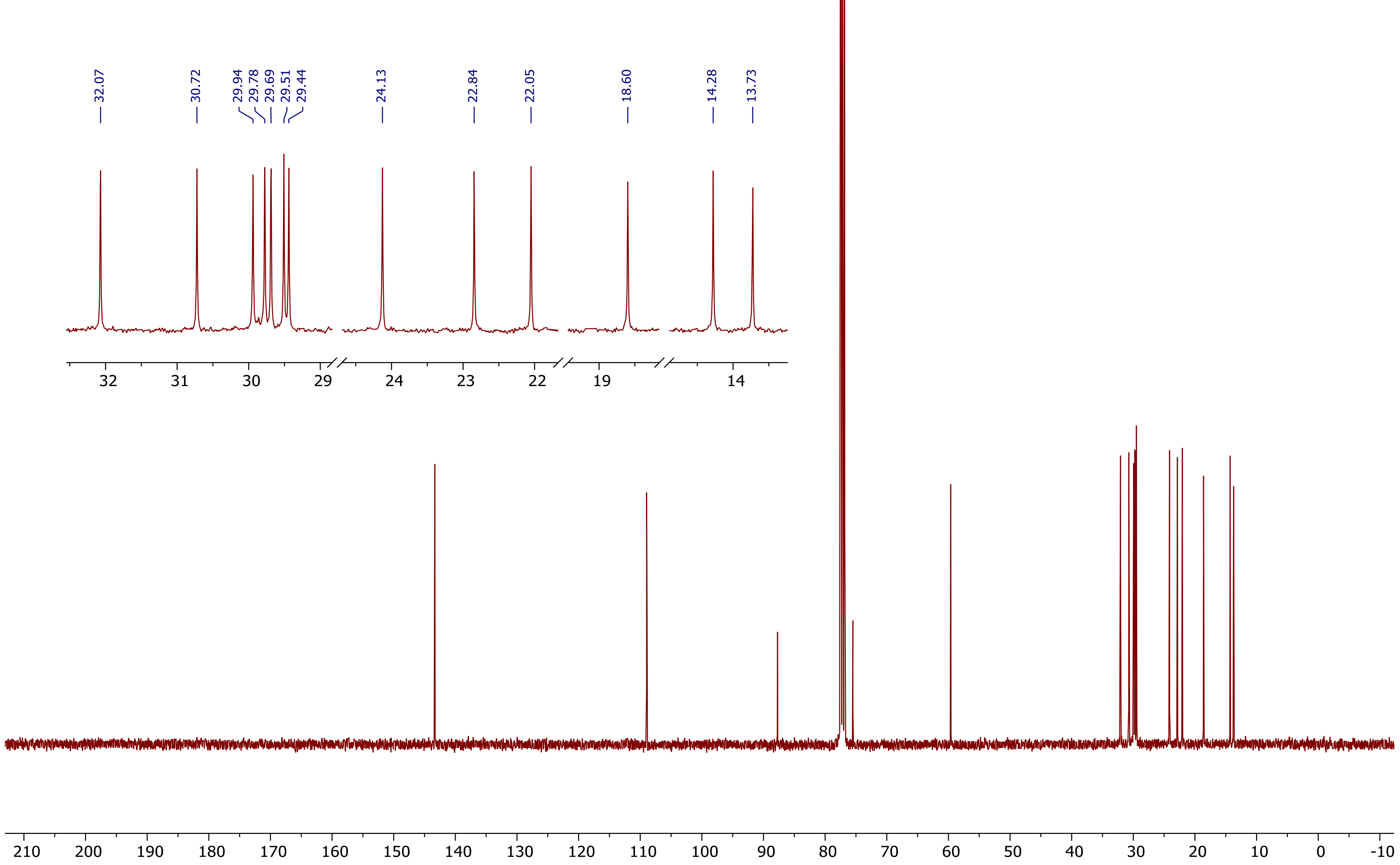

^{1}H NMR spectrum of **15** (400 MHz, $CDCl_3$)

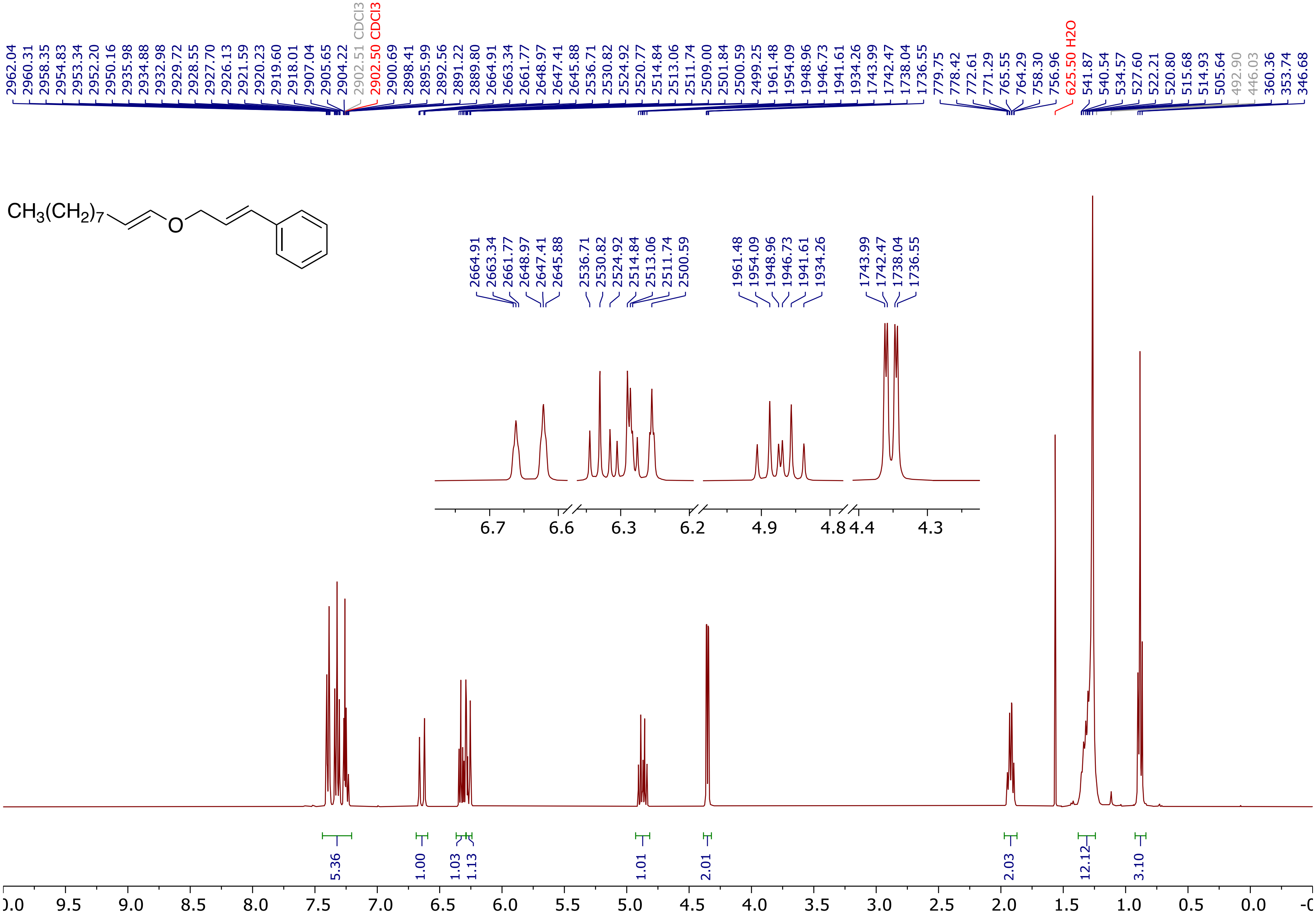

^{13}C NMR spectrum of **15** (101 MHz, $CDCl_3$)

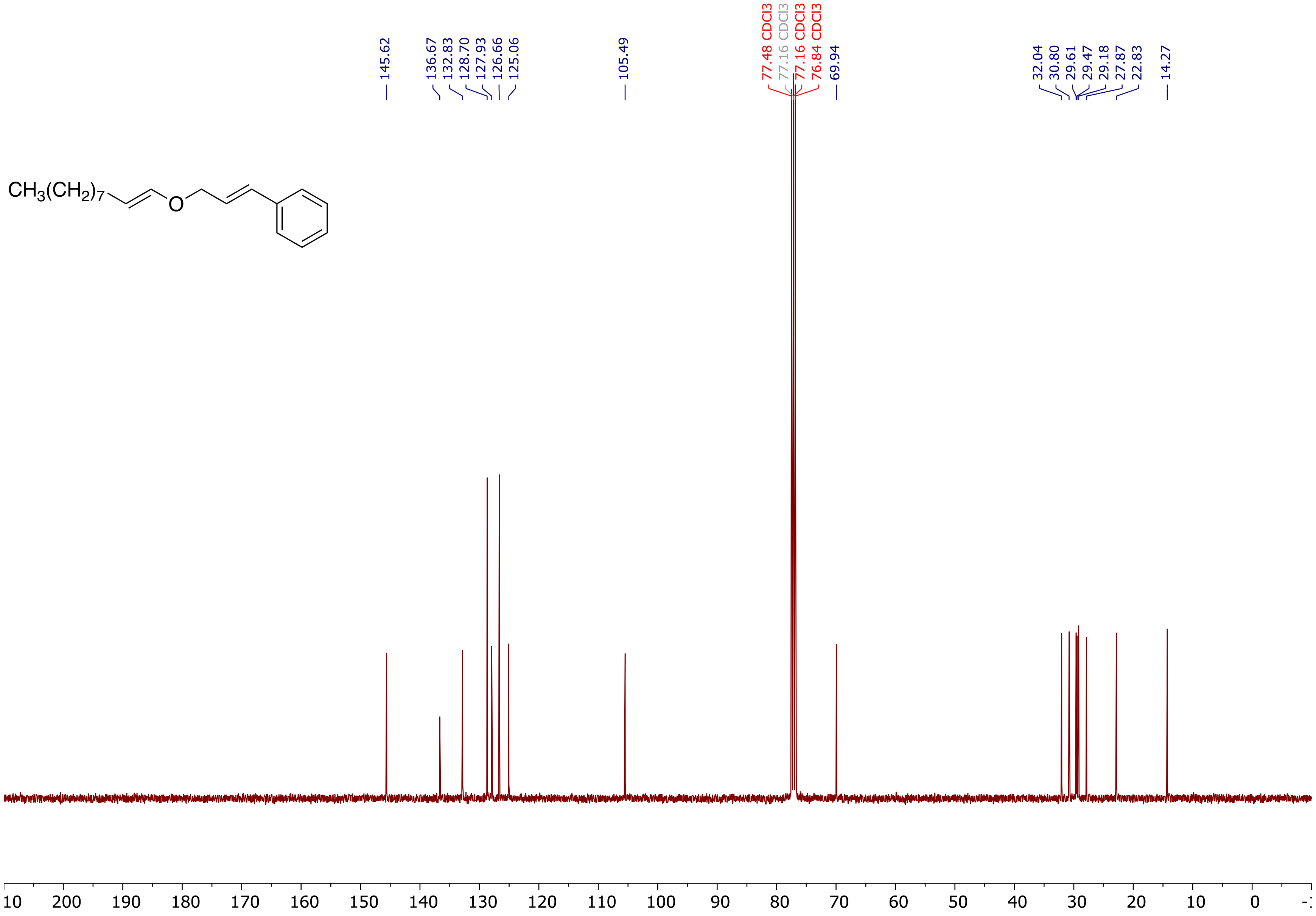

^{1}H NMR spectrum of **16** (>20:1 Z/E, 400 MHz, $CDCl_3$)

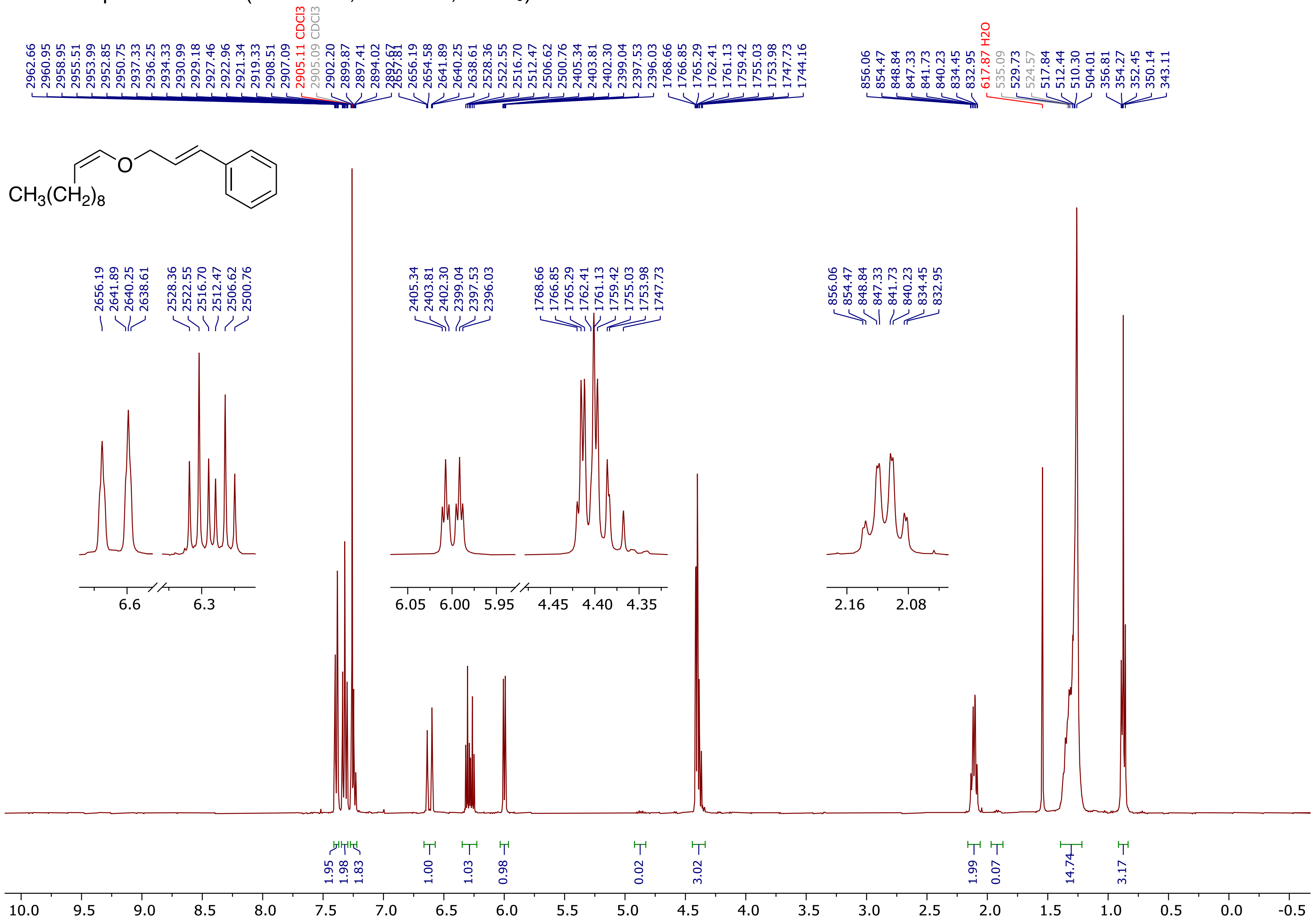

^{13}C NMR spectrum of **16** (>20:1 Z/E, 101 MHz, $CDCl_3$)

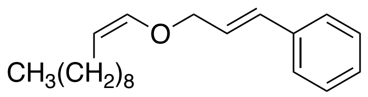

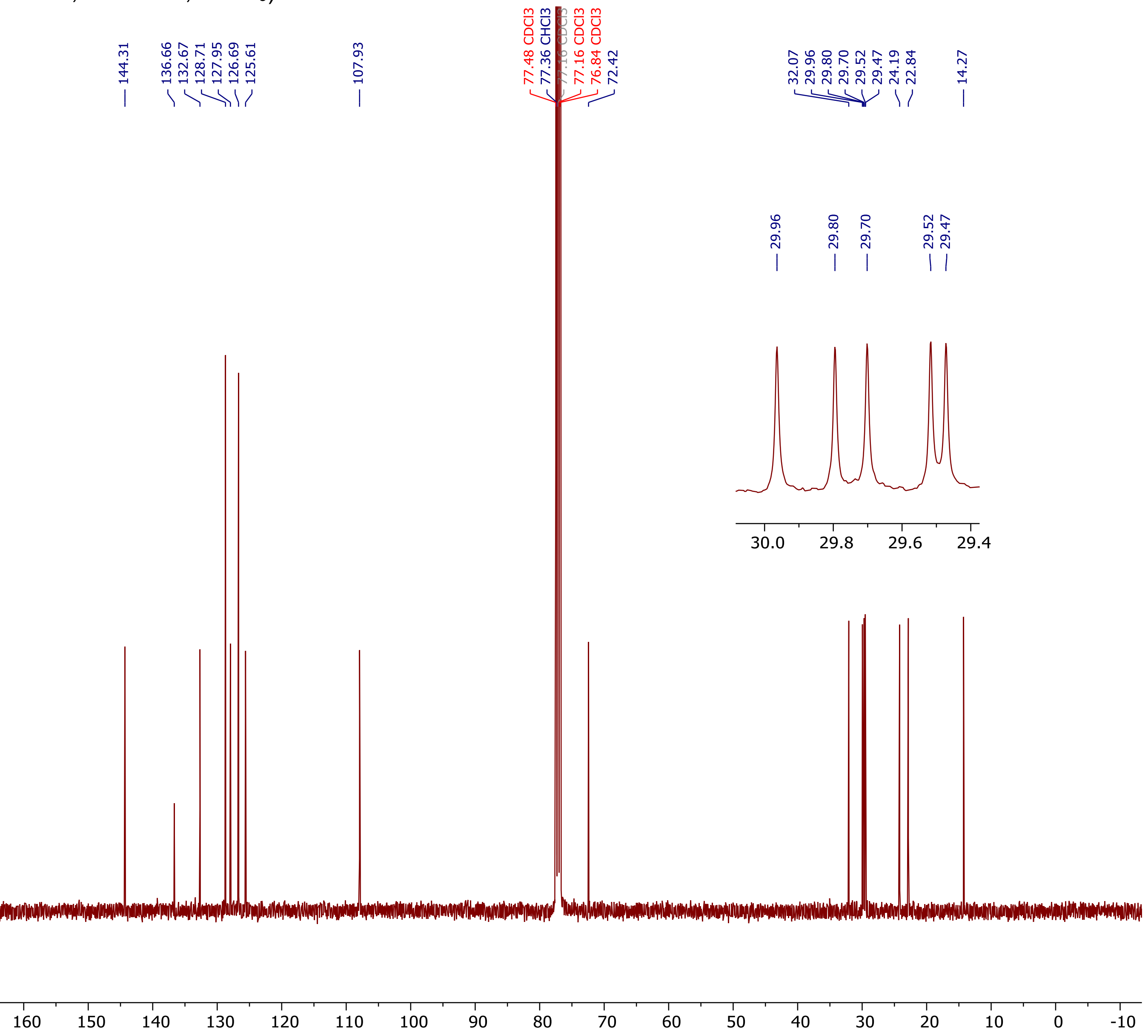

^{1}H NMR spectrum of **17** (400 MHz, $CDCl_3$)

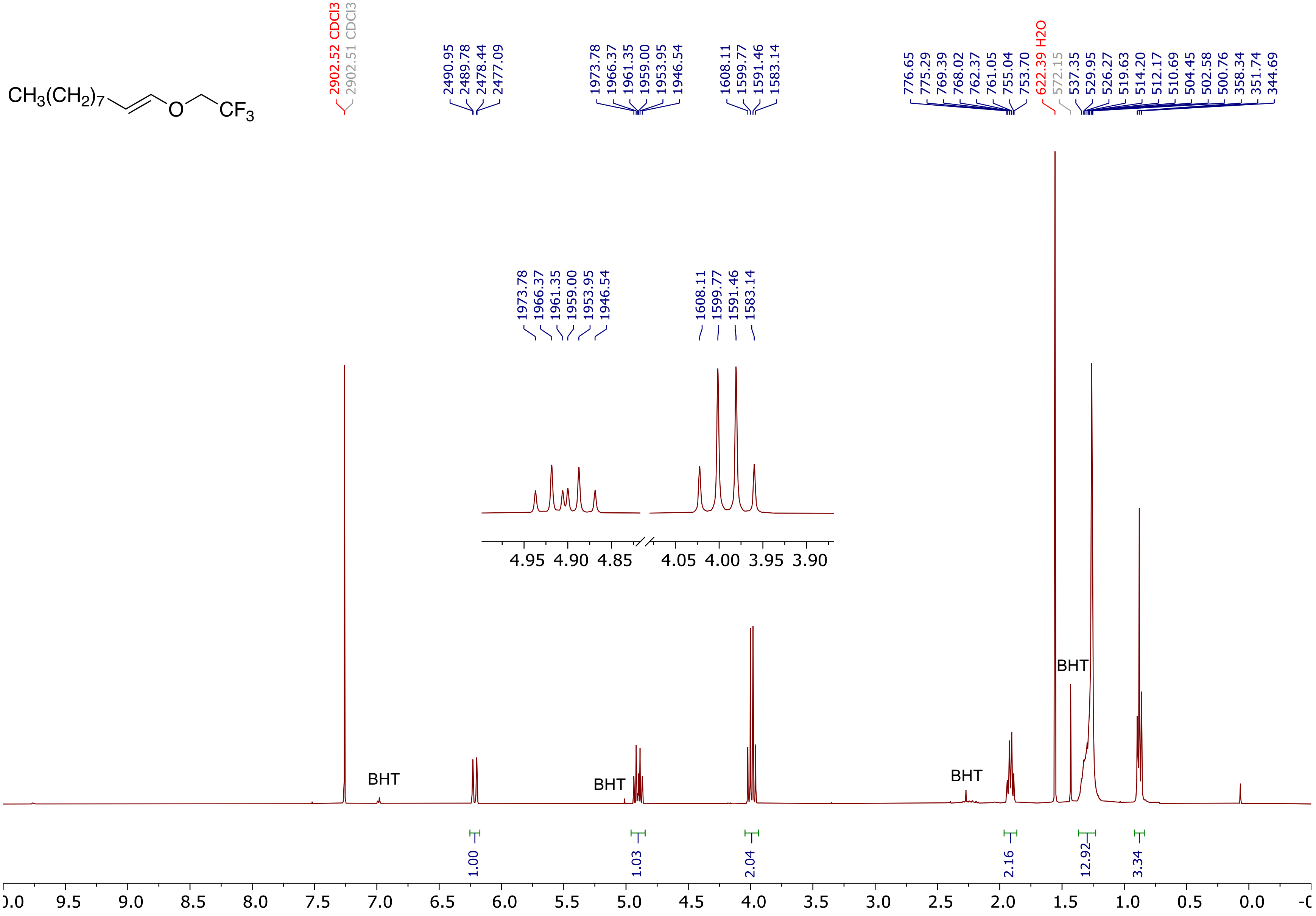

^{13}C NMR spectrum of **17** (101 MHz, $CDCl_3$)

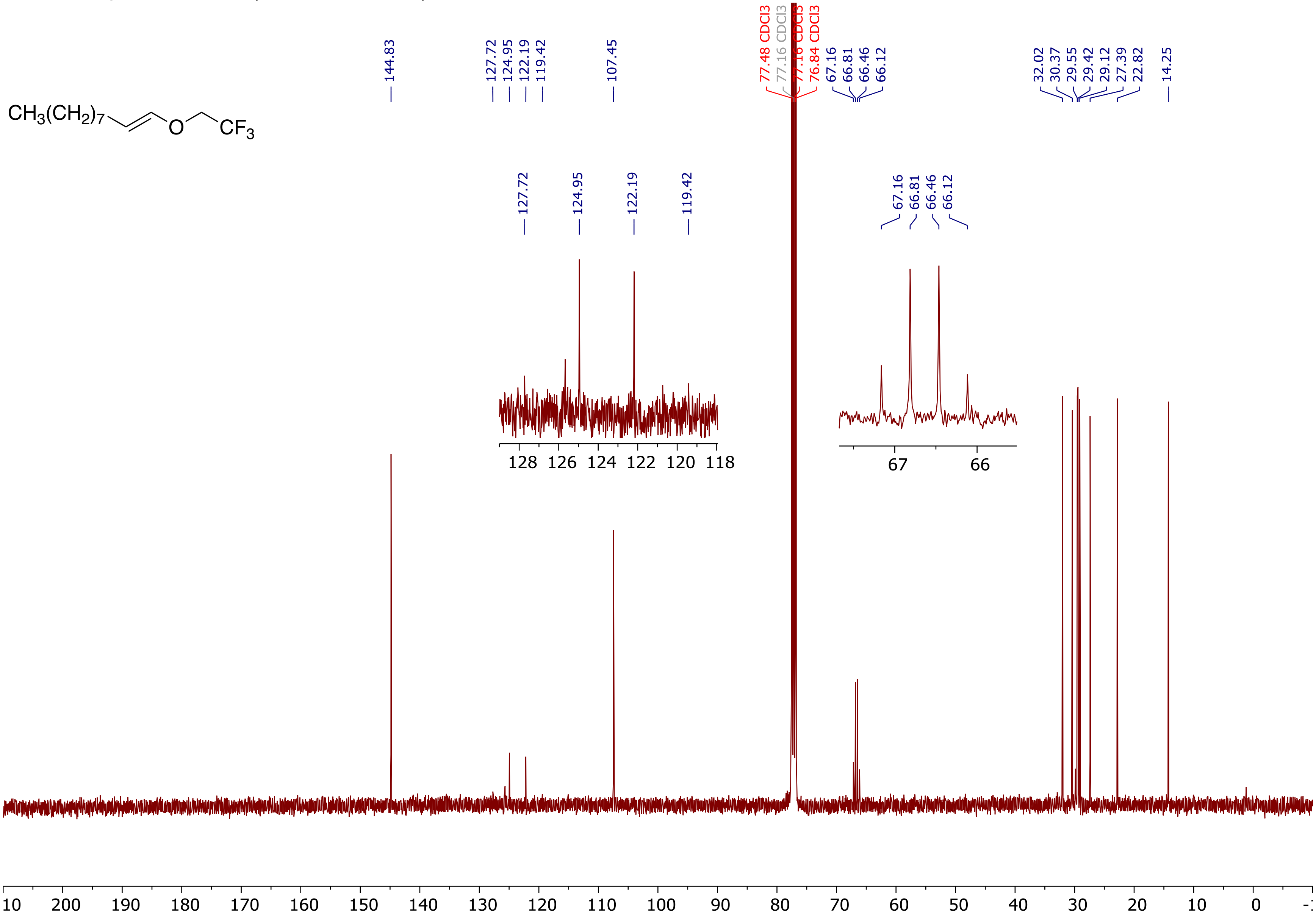

^{19}F NMR spectrum of **17** (376 MHz, $CDCl_3$)

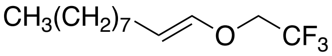

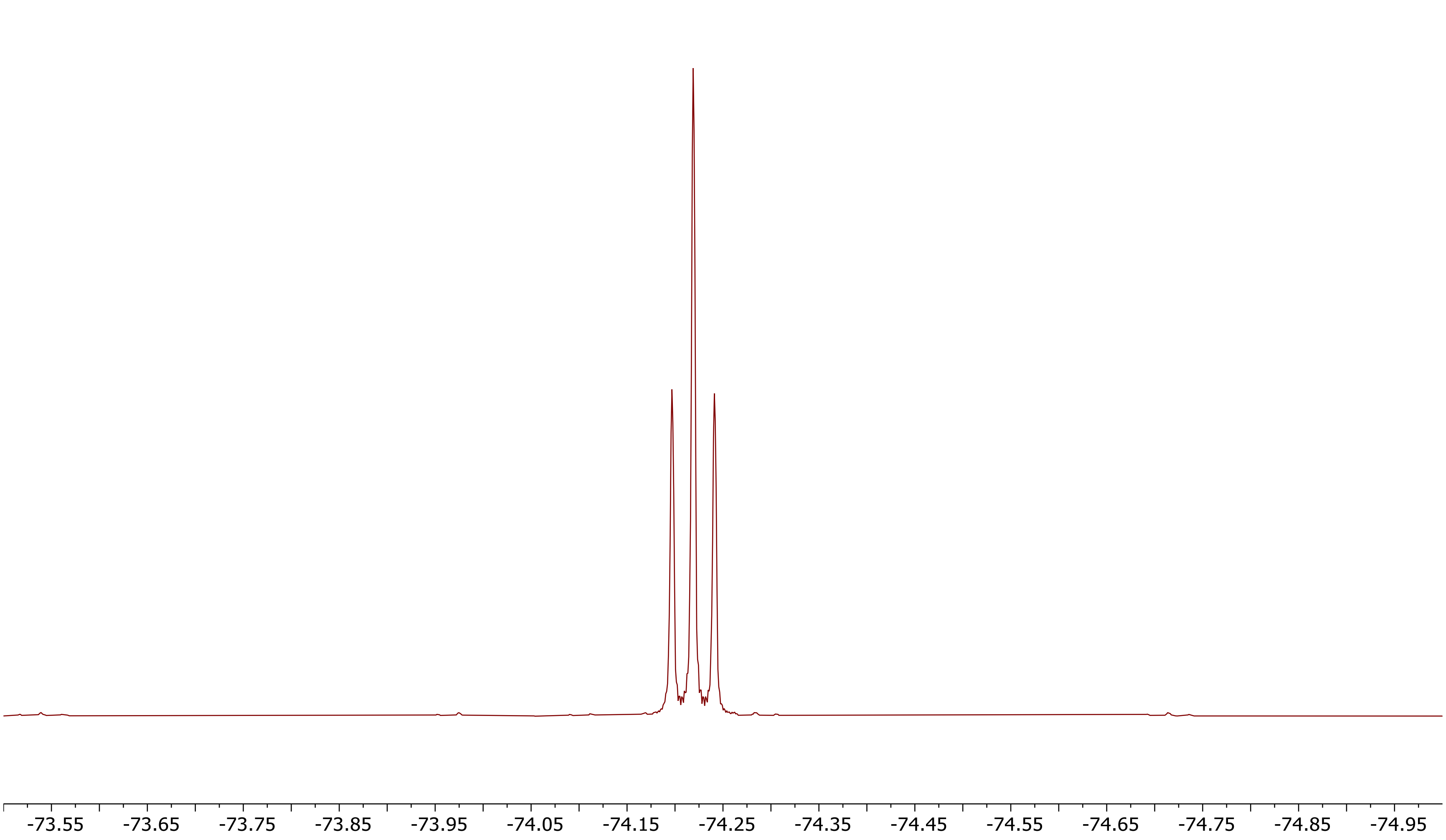

^{1}H NMR spectrum of **18** (>20:1 Z/E, 400 MHz, $CDCl_3$)

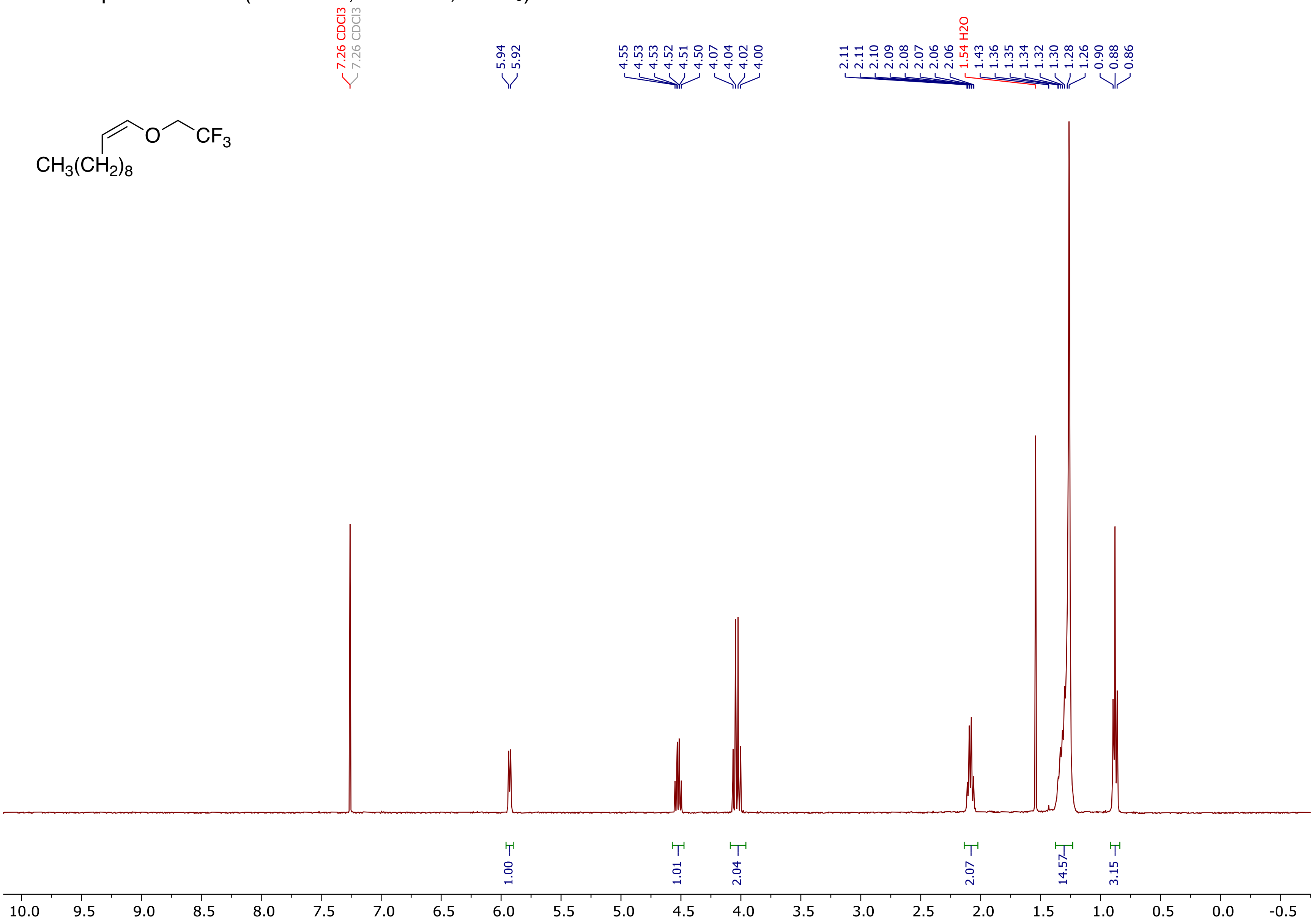

^{13}C NMR spectrum of **18** (>20:1 Z/E, 101 MHz, $CDCl_3$)

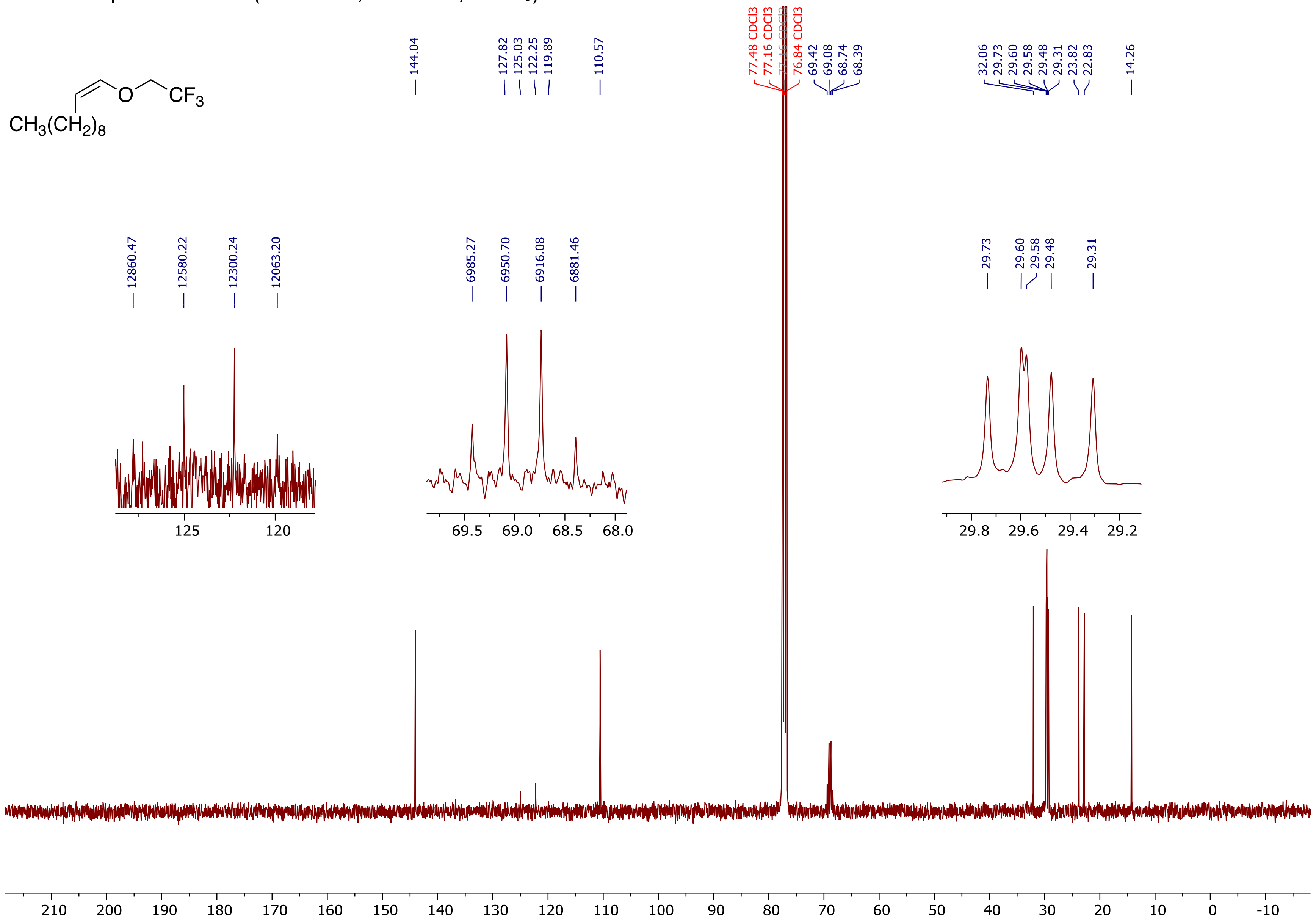

^{19}F NMR spectrum of **18** (>20:1 Z/E, 376 MHz, $CDCl_3$)

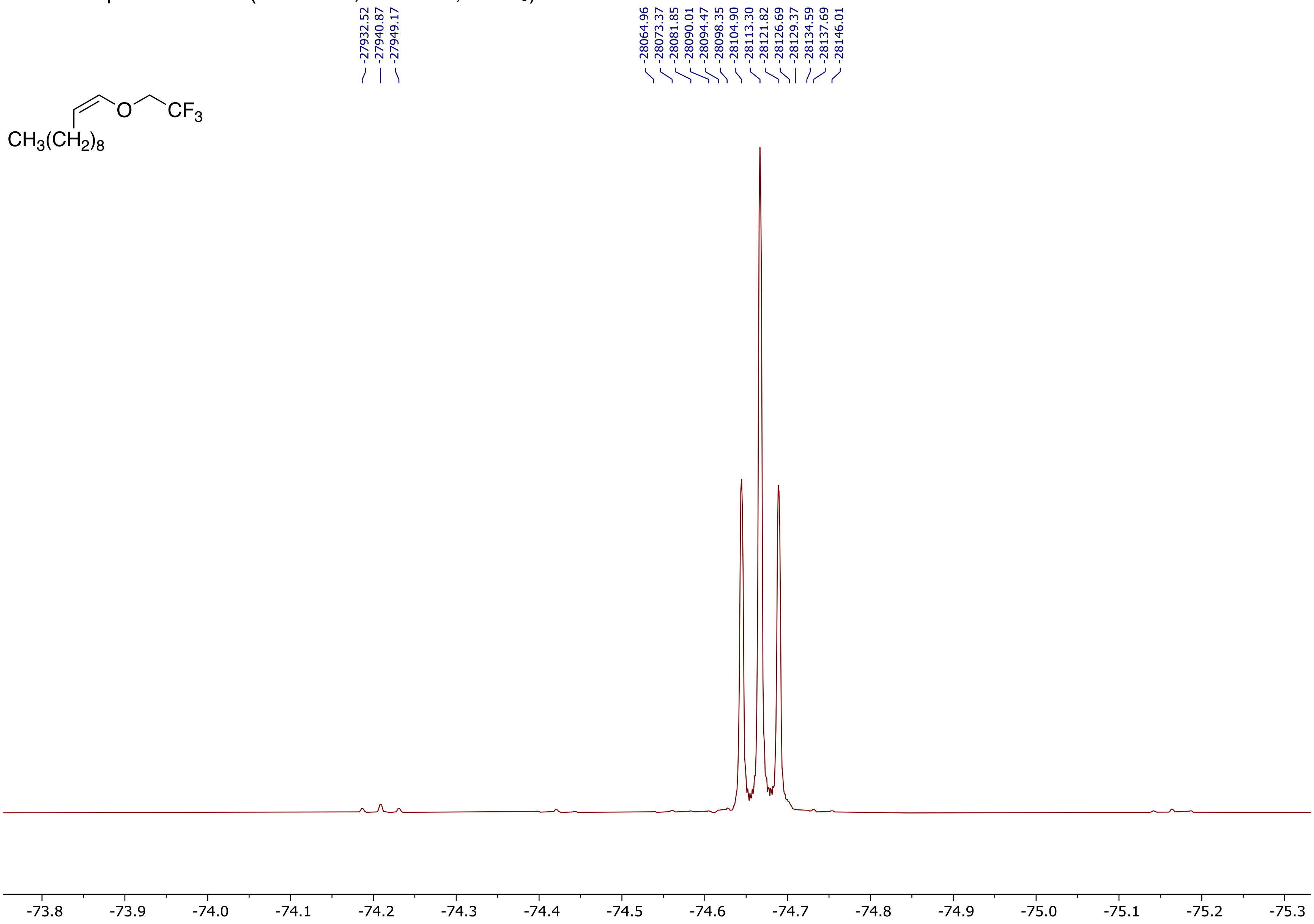

^{1}H NMR spectrum of **19** (400 MHz, $CDCl_3$)

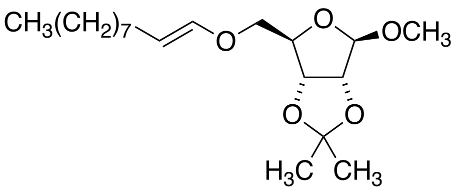

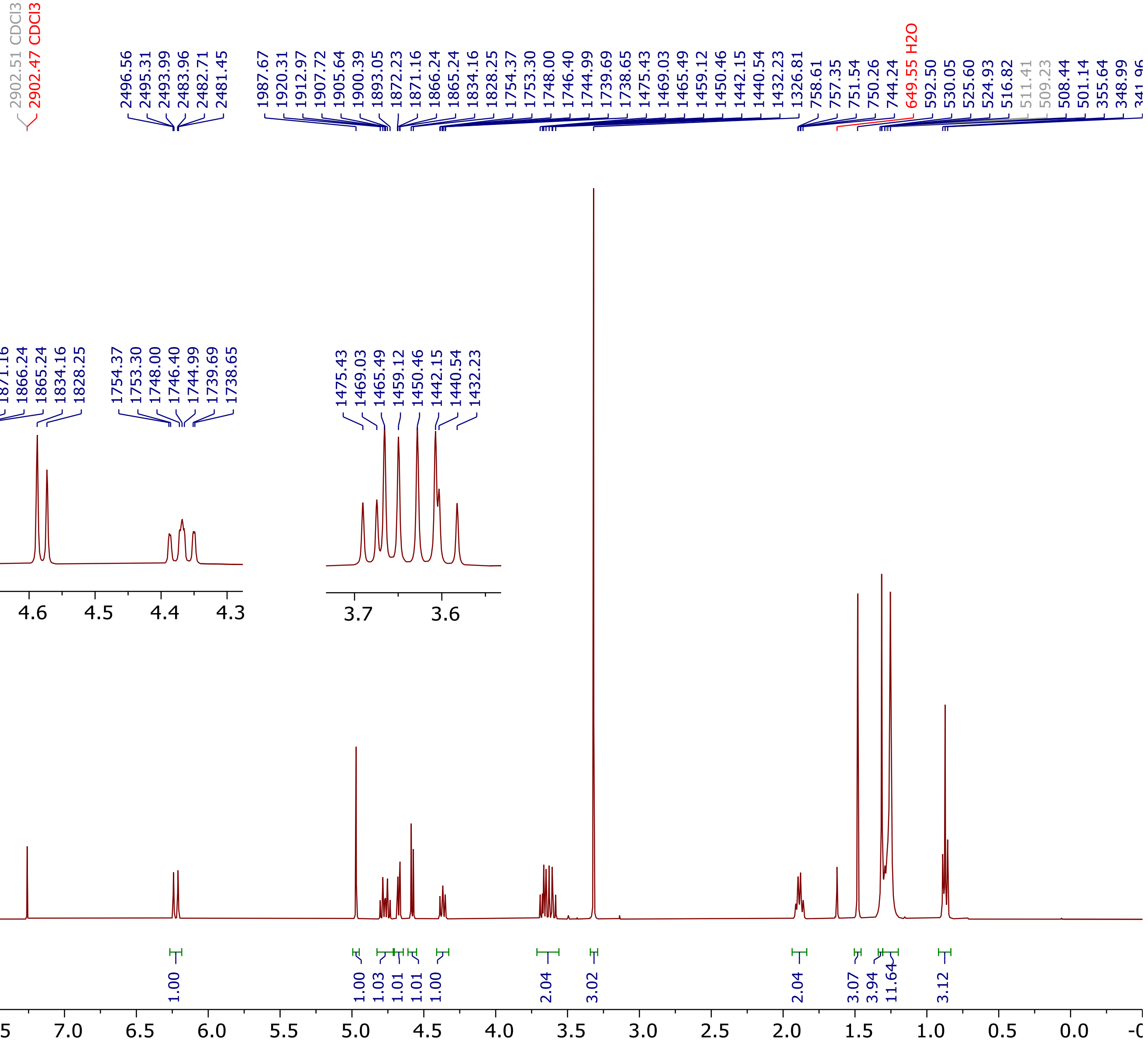

^{13}C NMR spectrum of **19** (101 MHz, $CDCl_3$)

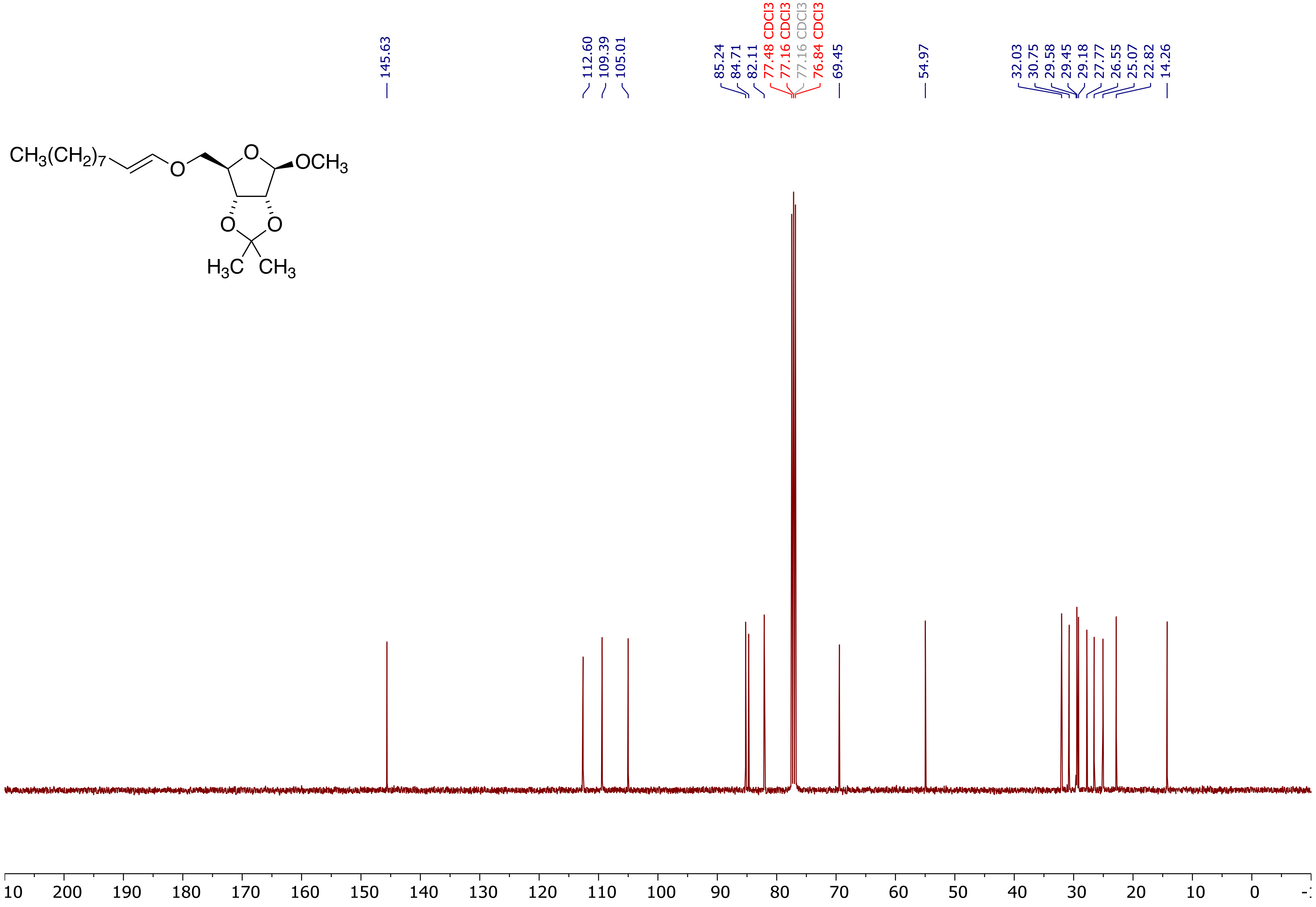

COSY spectrum of **19** (600 MHz, $CDCl_3$)

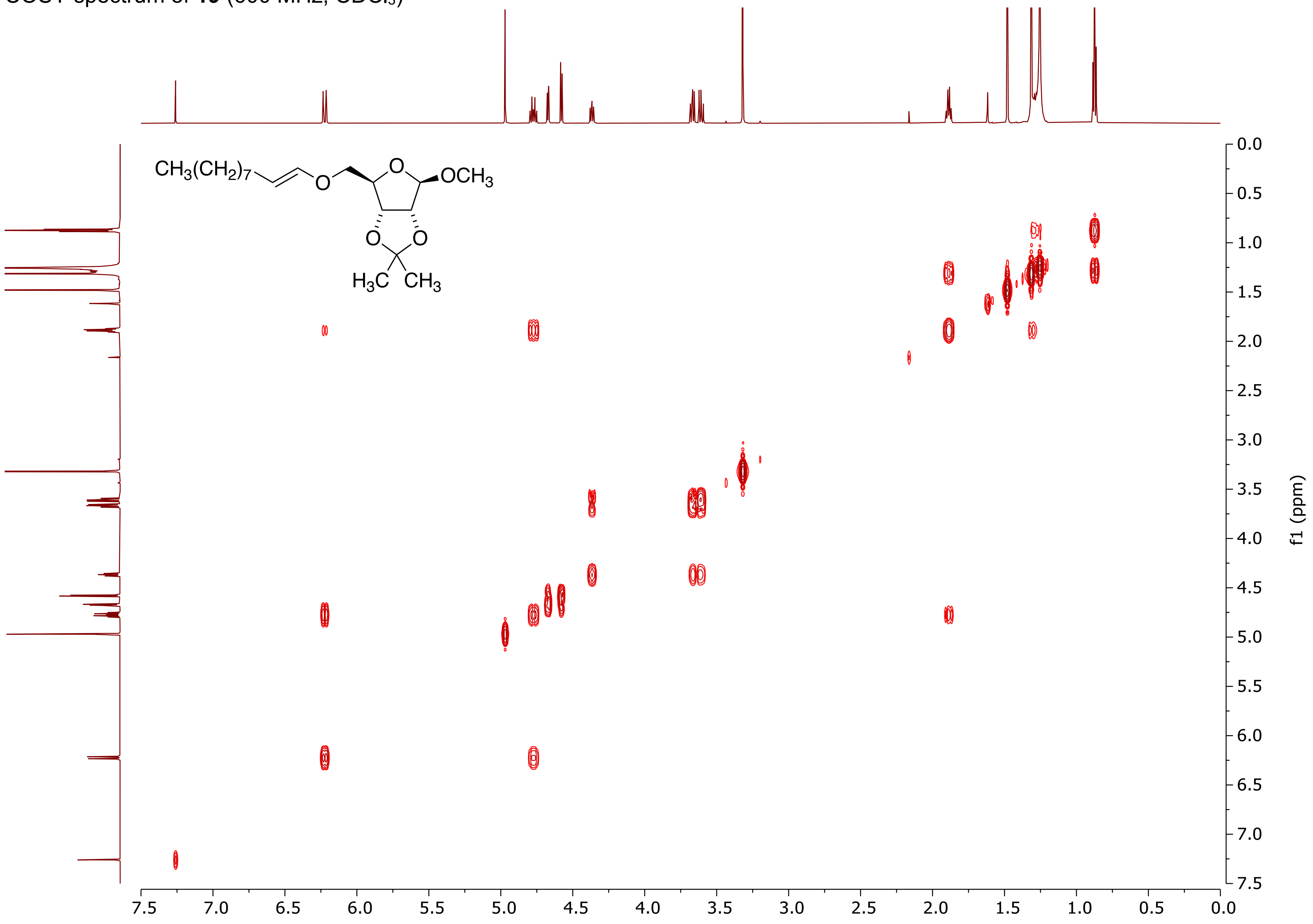

COSY spectrum of **19** (600 MHz, $CDCl_3$) expansion

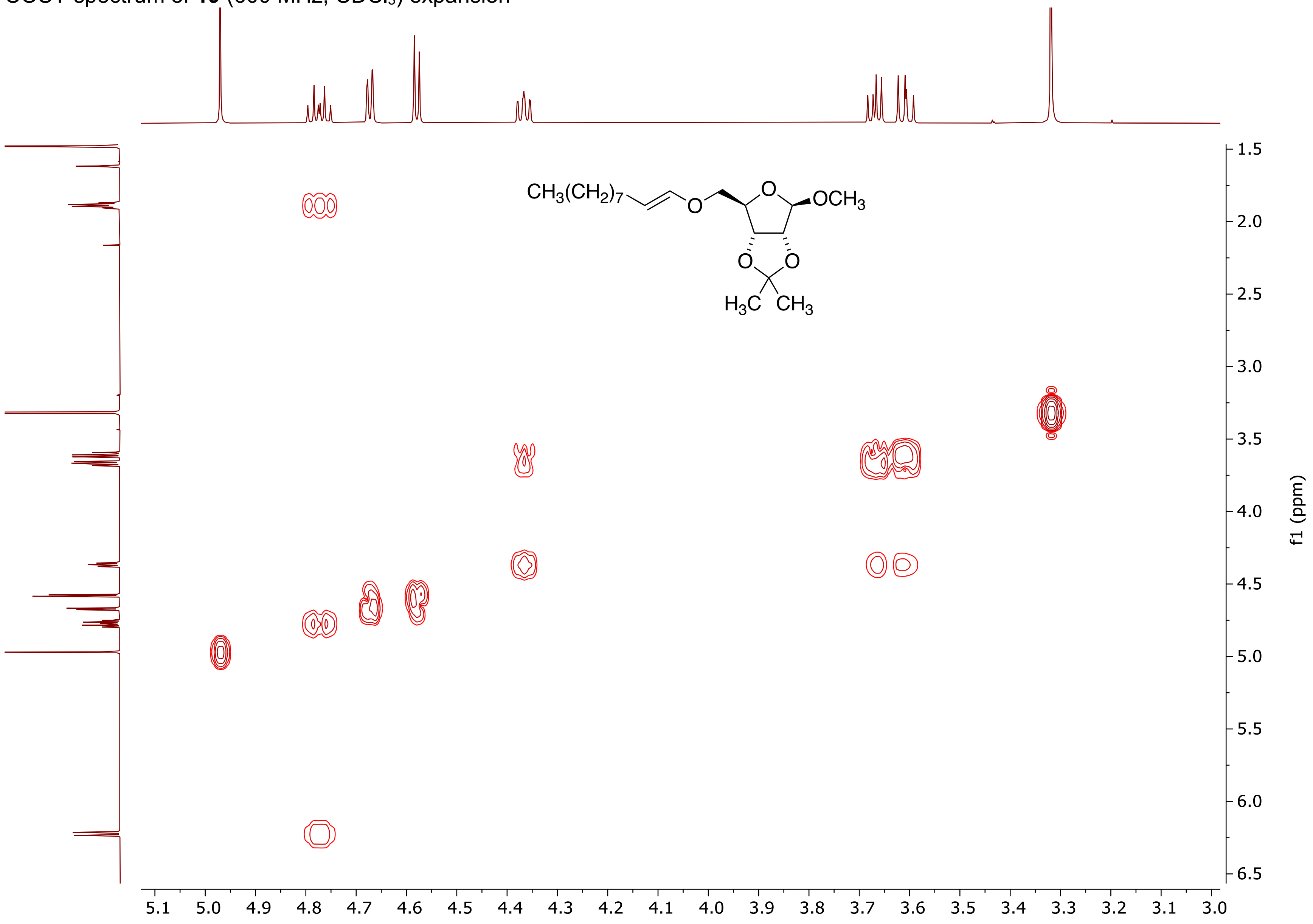

HSQC spectrum of **19** (600 MHz, $CDCl_3$)

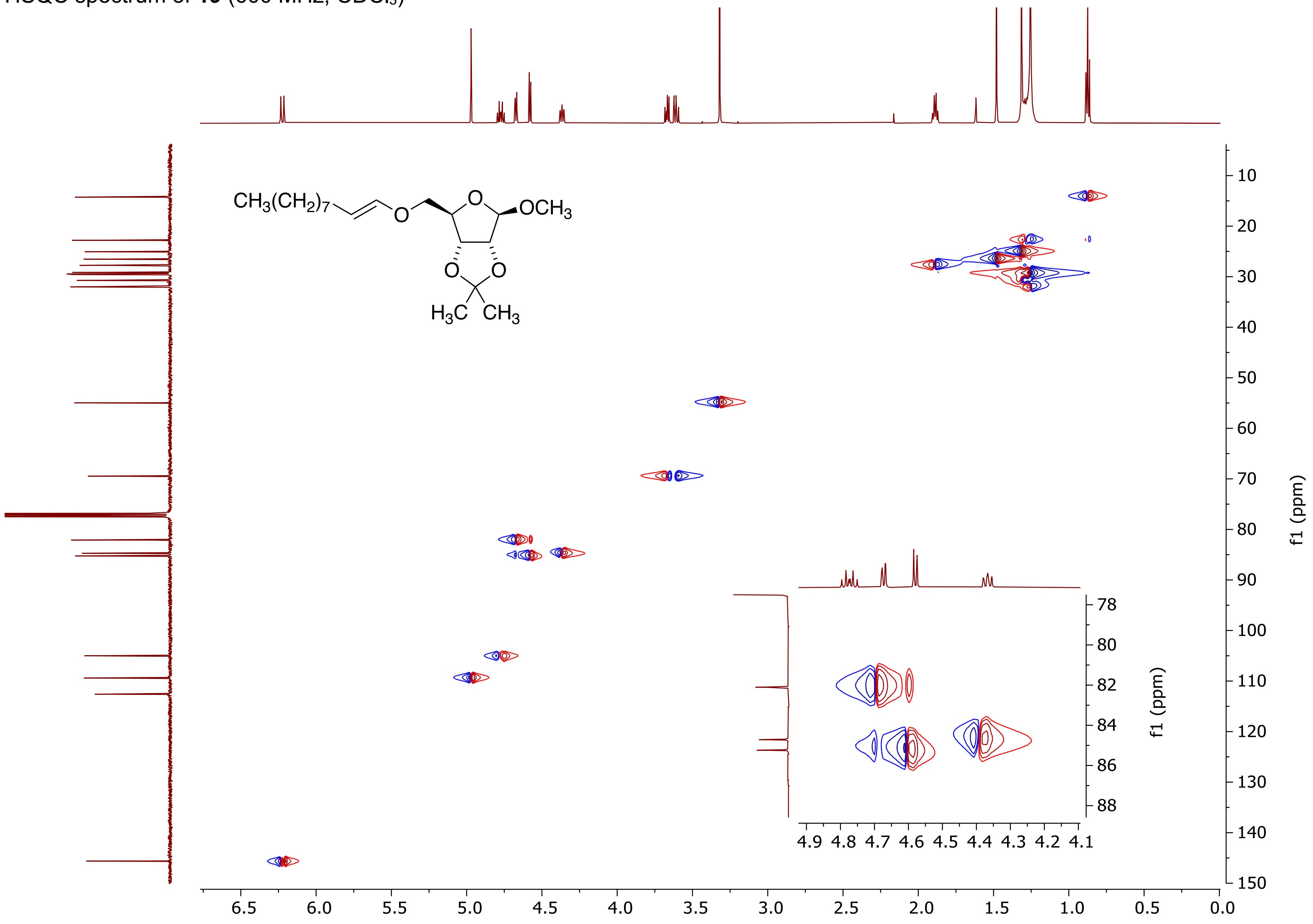

^{1}H NMR spectrum of **20** (20:1 Z/E, 800 MHz, $CDCl_3$)

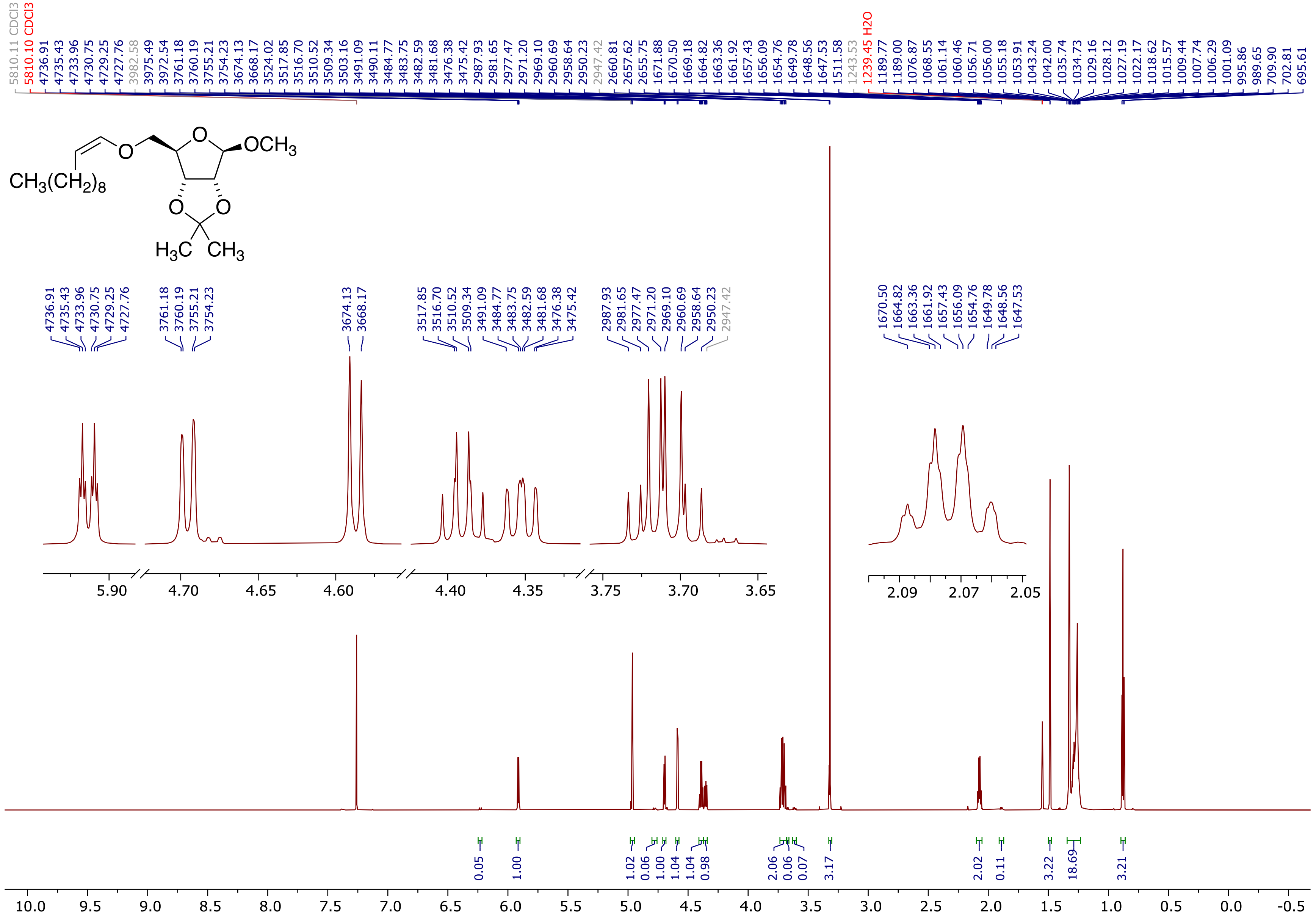

^{13}C NMR spectrum of **20** (20:1 Z/E, 201 MHz, $CDCl_3$)

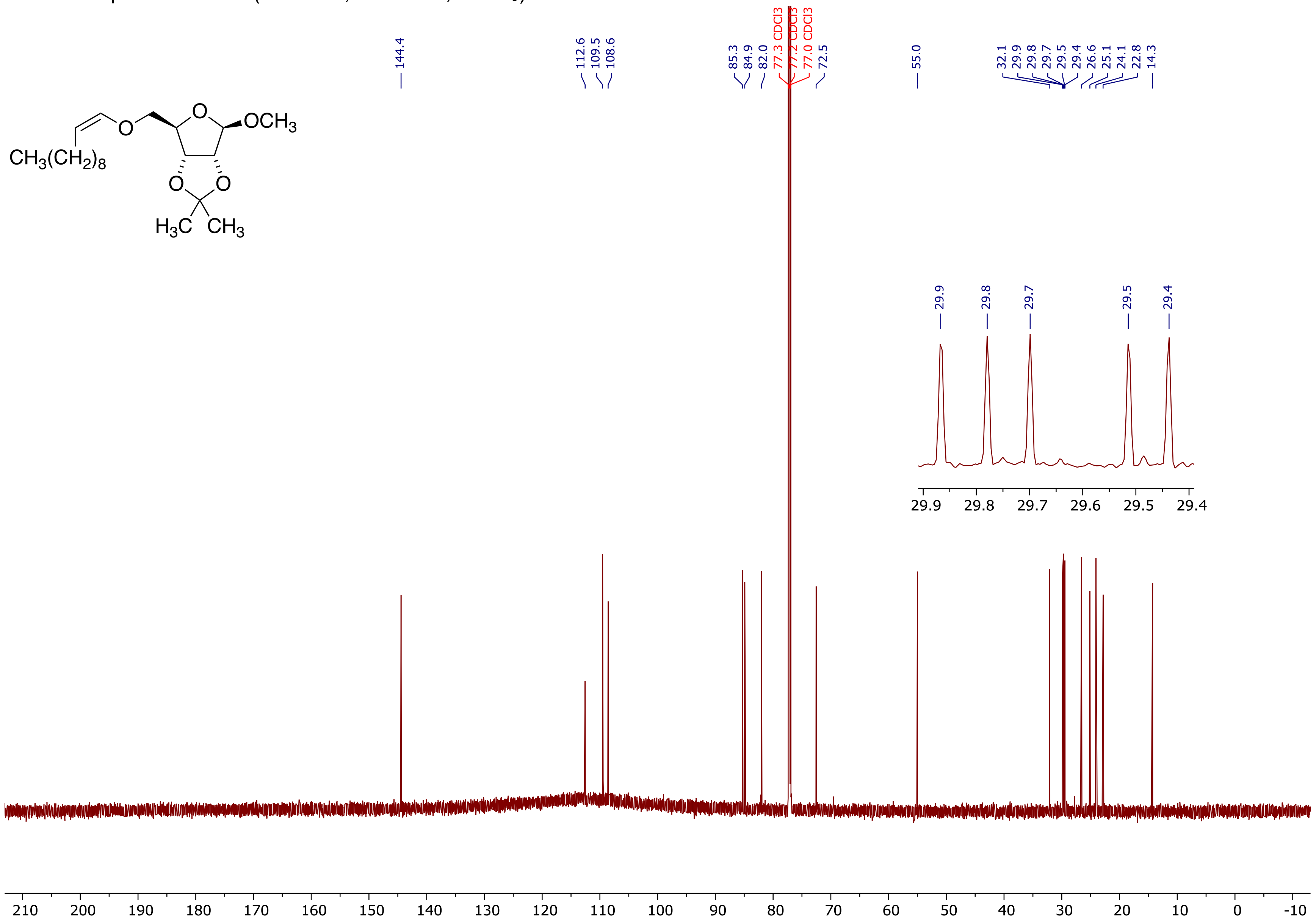

COSY spectrum of **20** (20:1 Z/E, 800 MHz, $CDCl_3$)

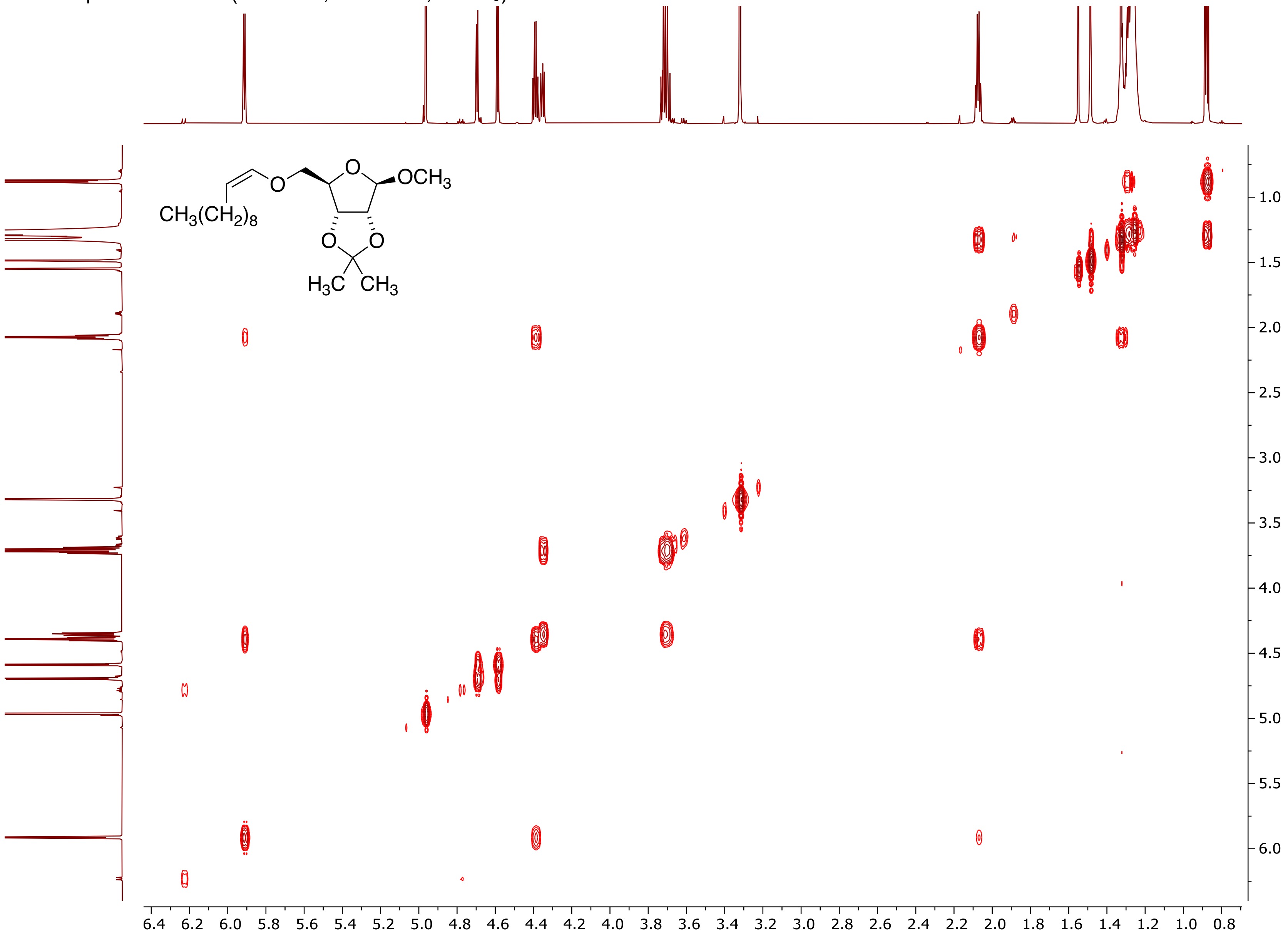

HSQC spectrum of **20** (20:1 Z/E, 800 MHz, $CDCl_3$)

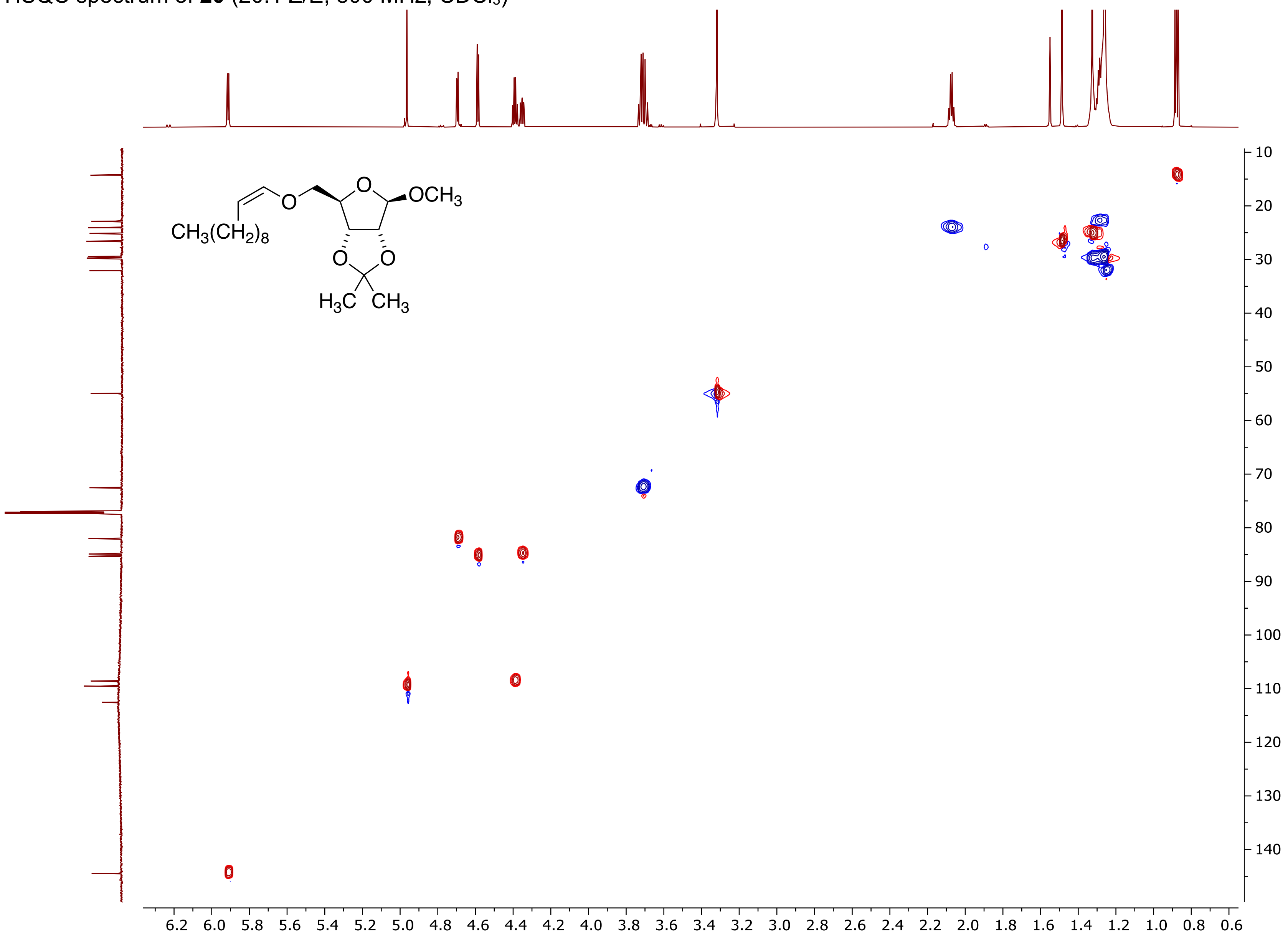

^{1}H NMR spectrum of **21** (400 MHz, $CDCl_3$)

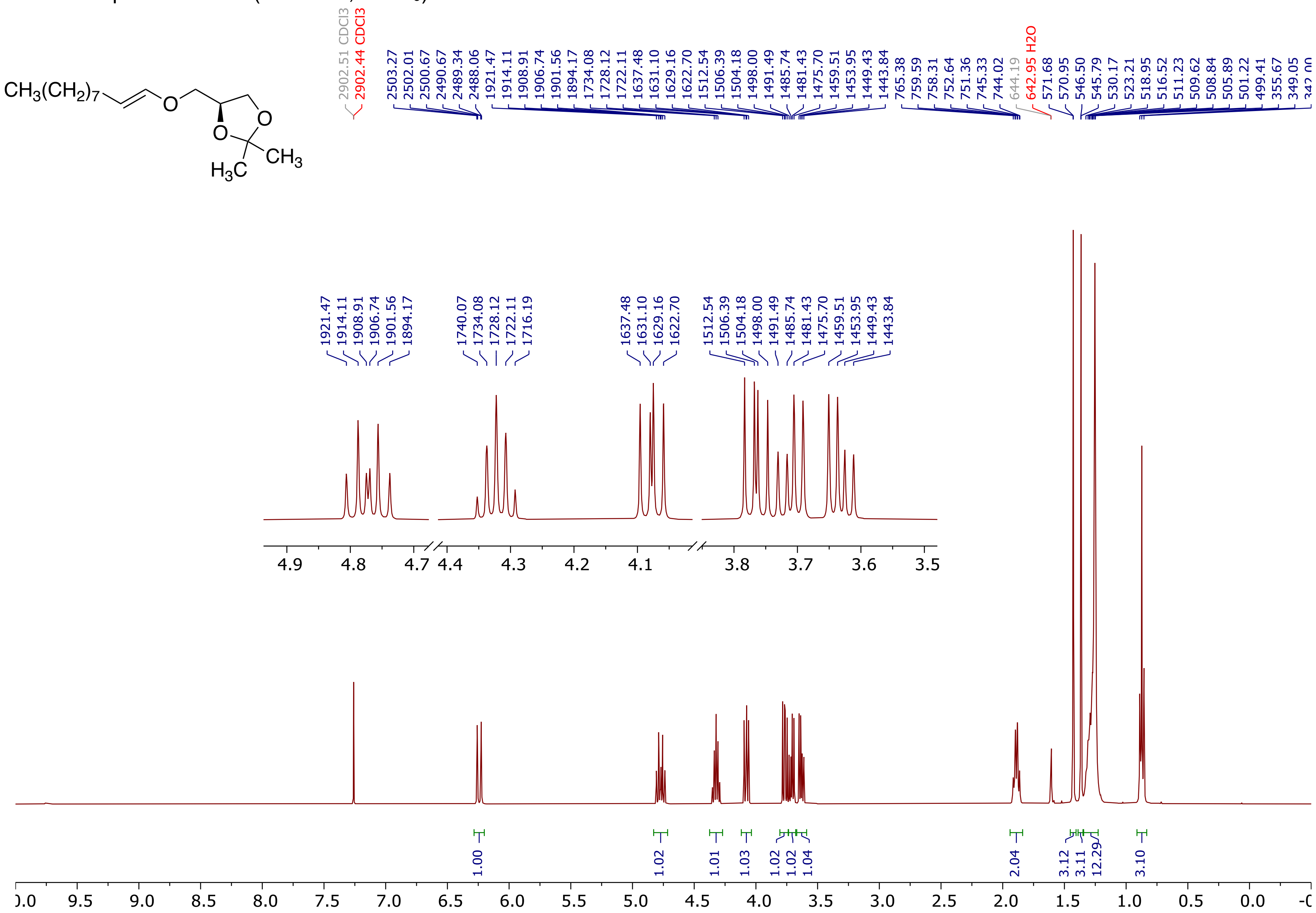

^{13}C NMR spectrum of **21** (101 MHz, $CDCl_3$)

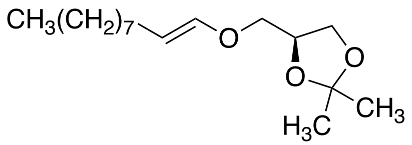

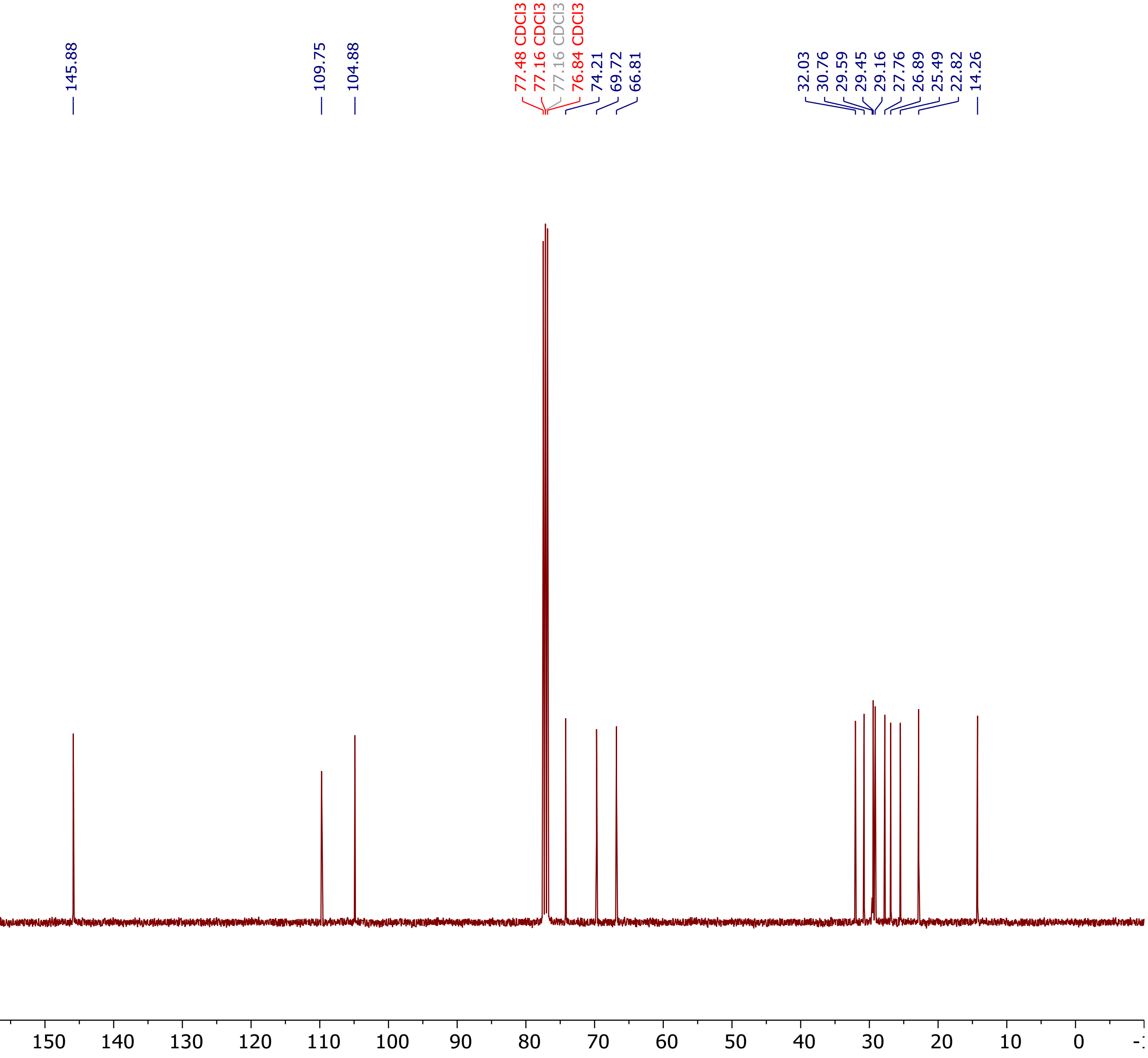

^{1}H NMR spectrum of **22** (20:1 Z/E, 800 MHz, $CDCl_3$)

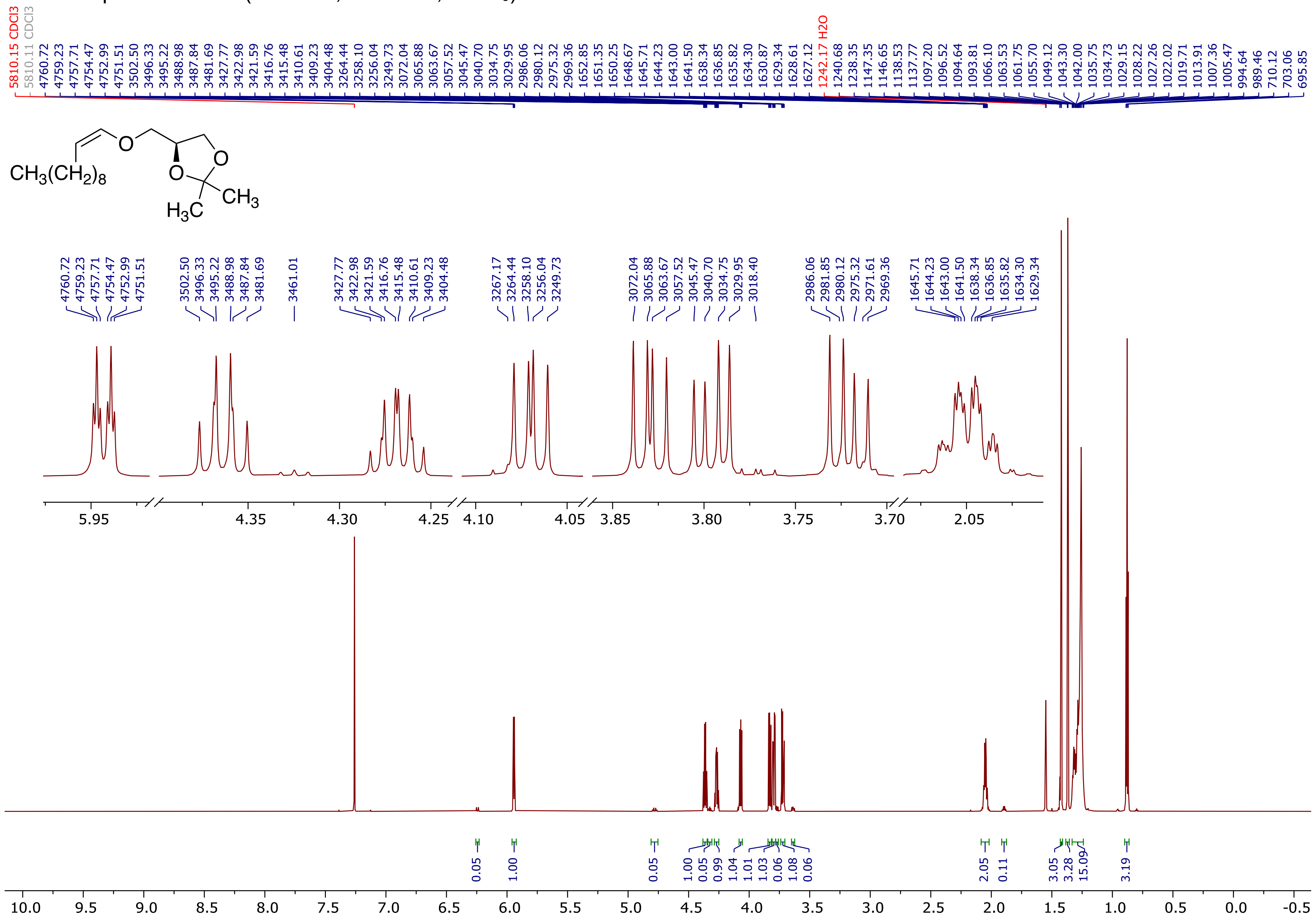

^{13}C NMR spectrum of **22** (20:1 Z/E, 201 MHz, $CDCl_3$)

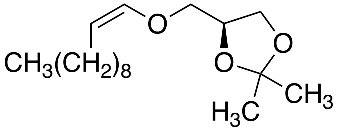

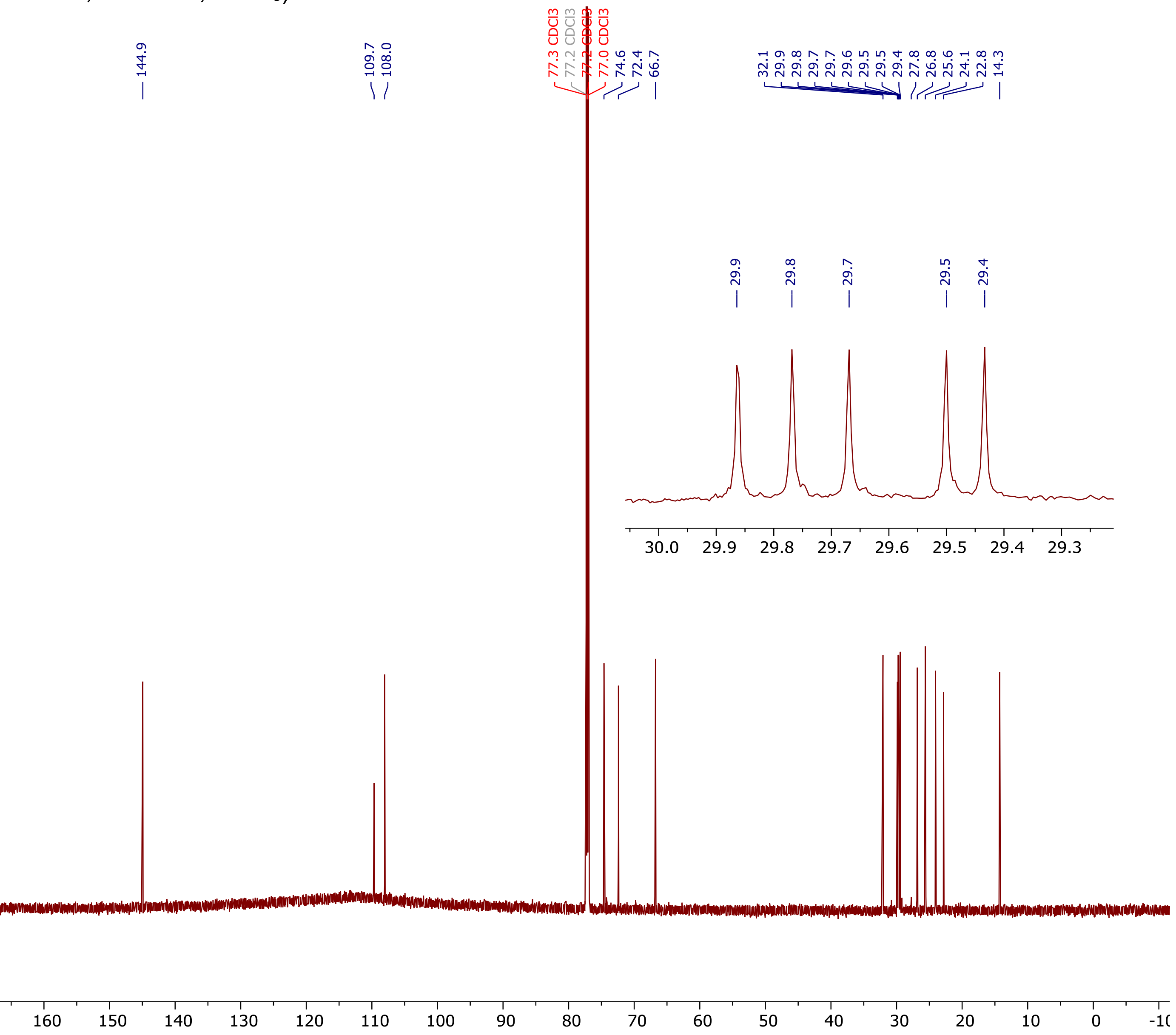

COSY spectrum of **22** (20:1 Z/E, 800 MHz, $CDCl_3$)

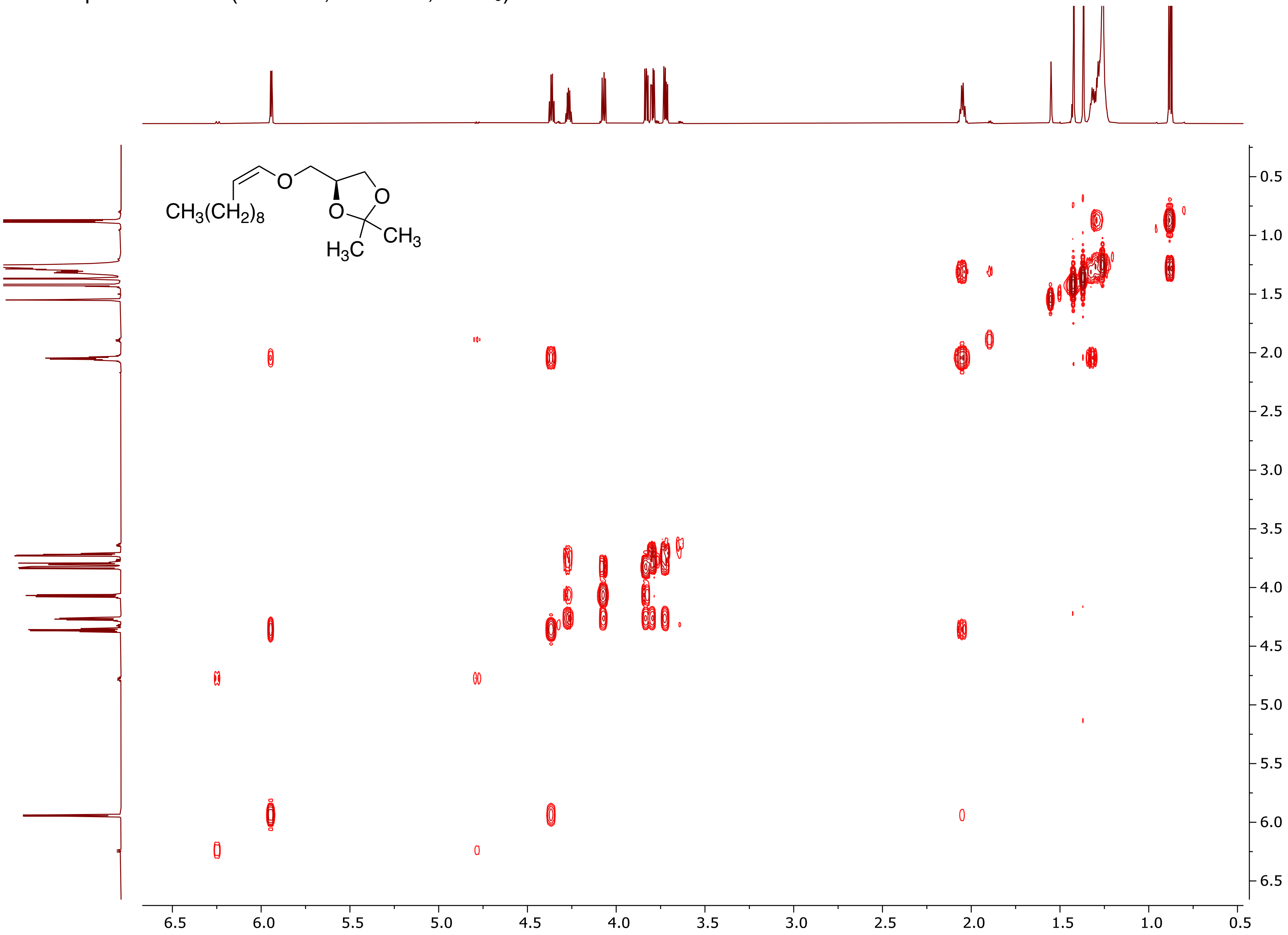

COSY spectrum of **22** (20:1 Z/E, 800 MHz, $CDCl_3$) expansion

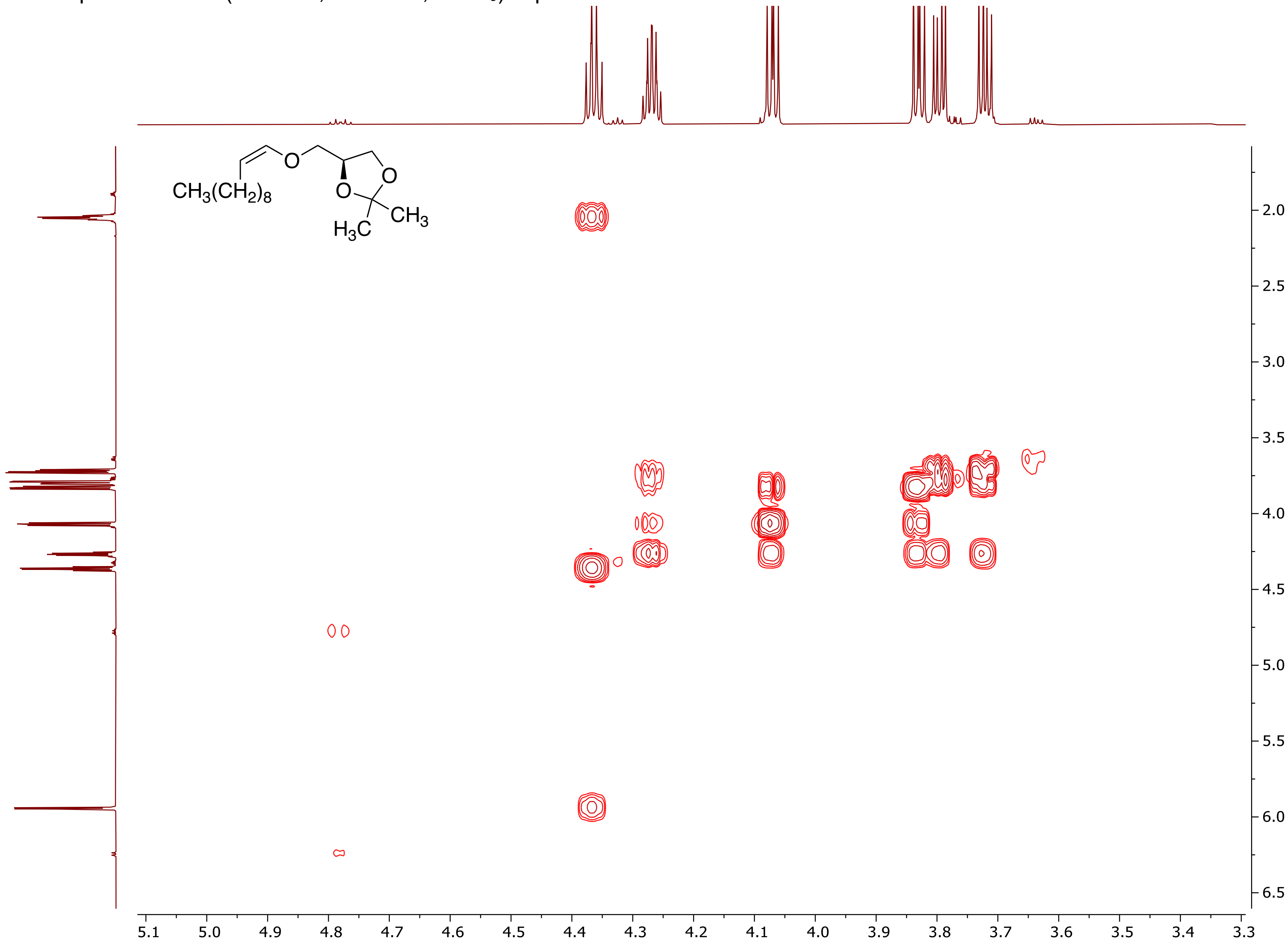

HSQC spectrum of **22** (20:1 Z/E, 800 MHz, $CDCl_3$)

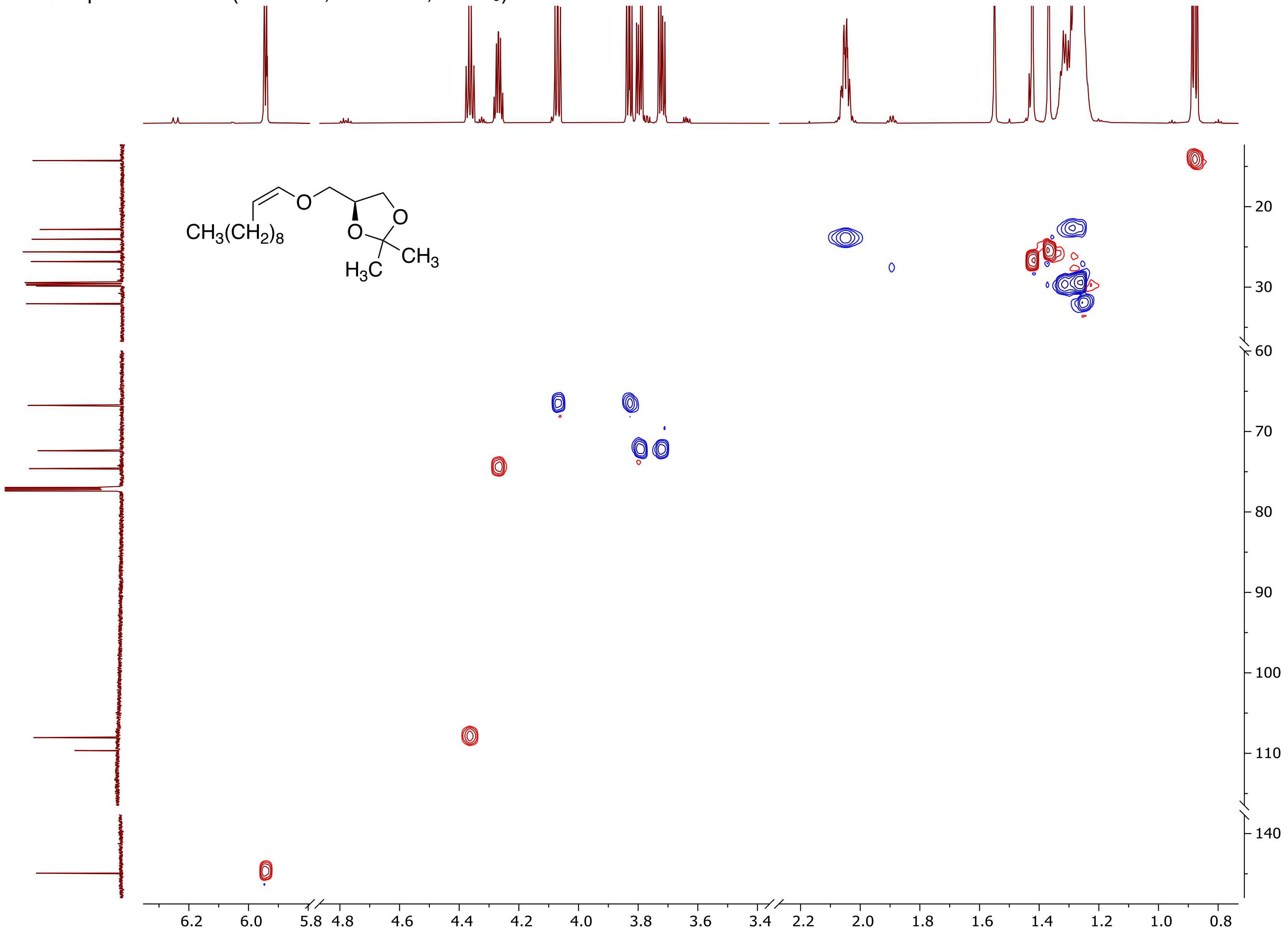

HMBC spectrum of **22** (20:1 Z/E, 800 MHz, $CDCl_3$)

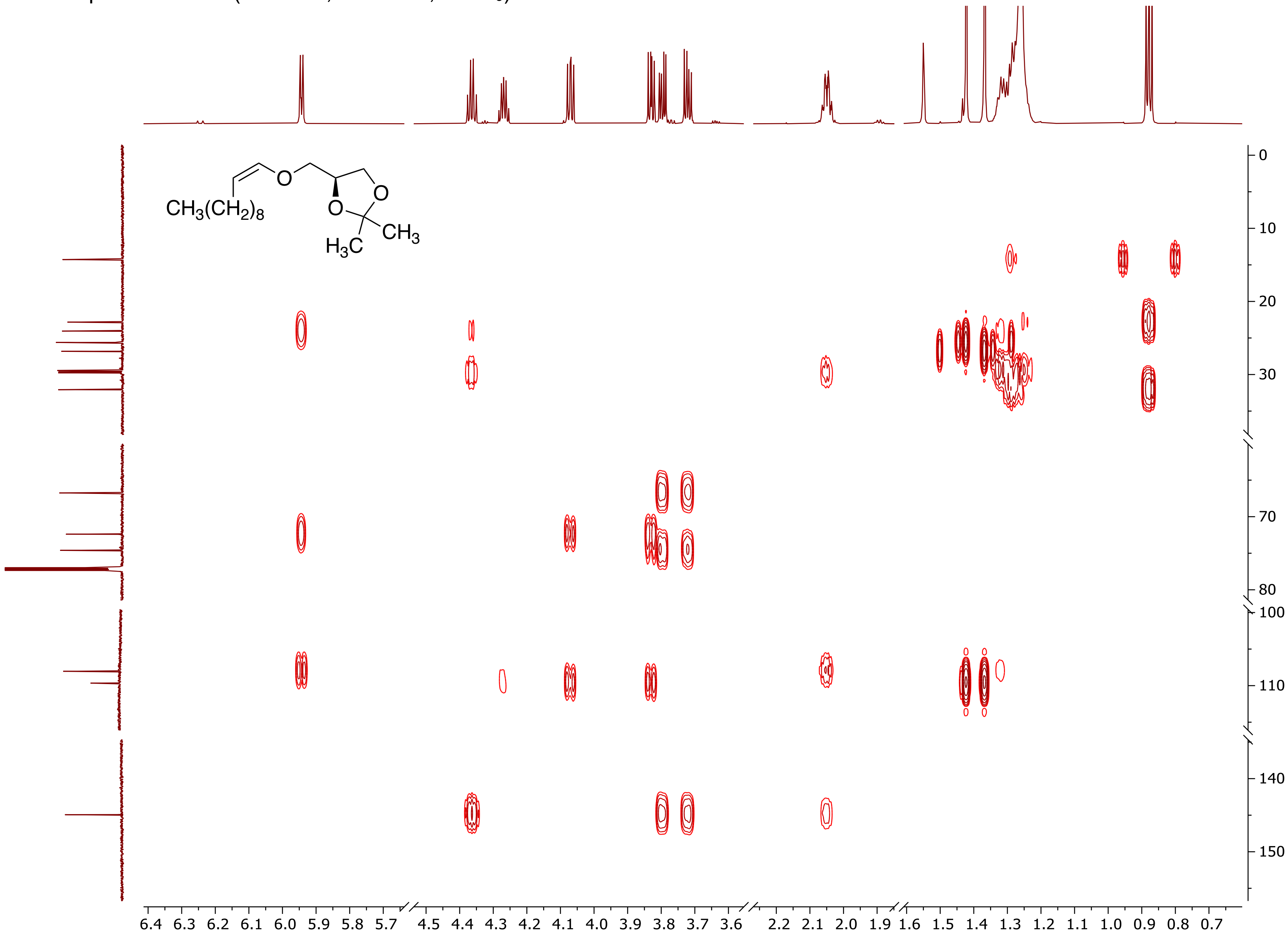

^{1}H NMR spectrum of **23** (400 MHz, $CDCl_3$)

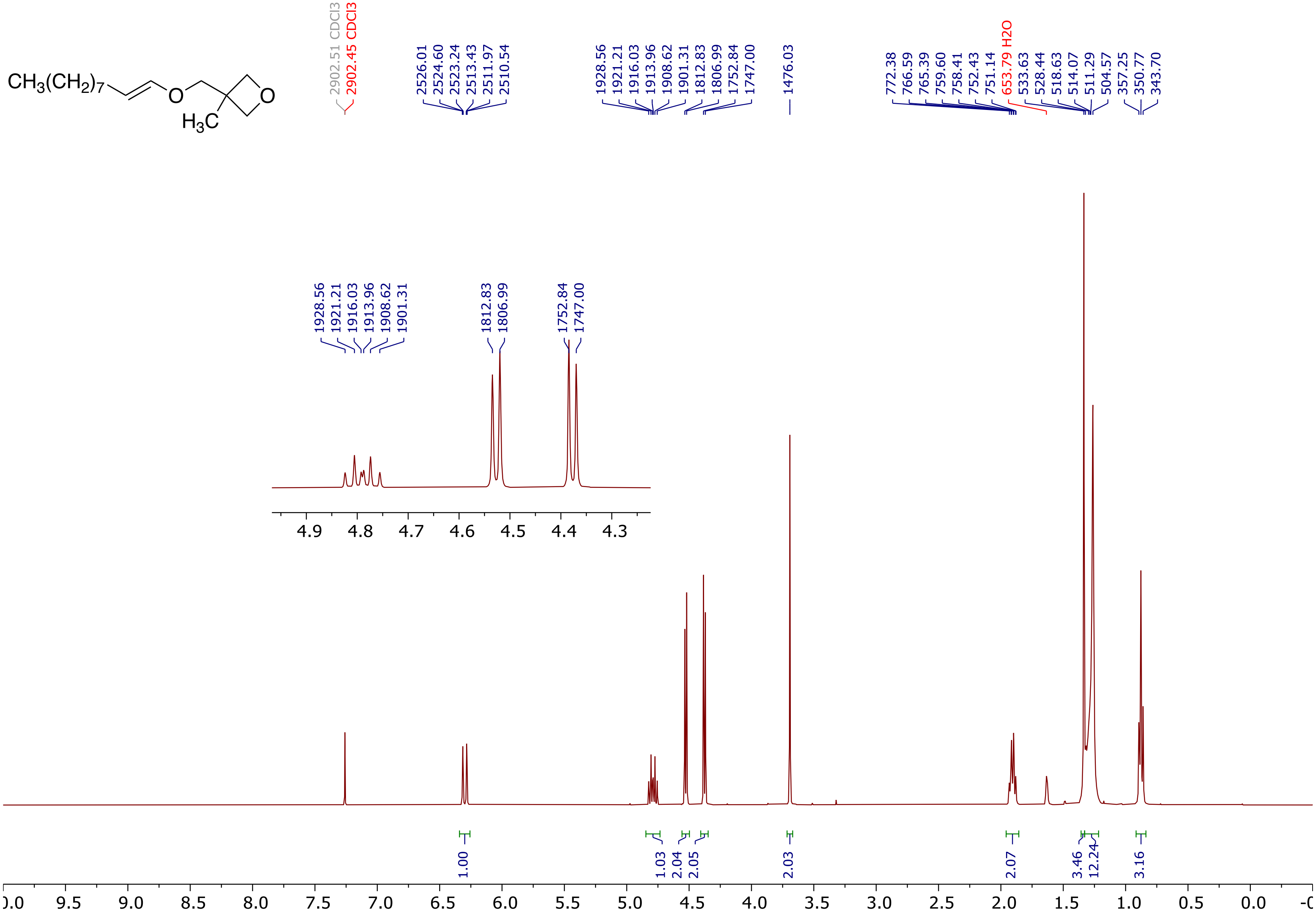

^{13}C NMR spectrum of **23** (101 MHz, $CDCl_3$)

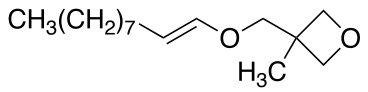

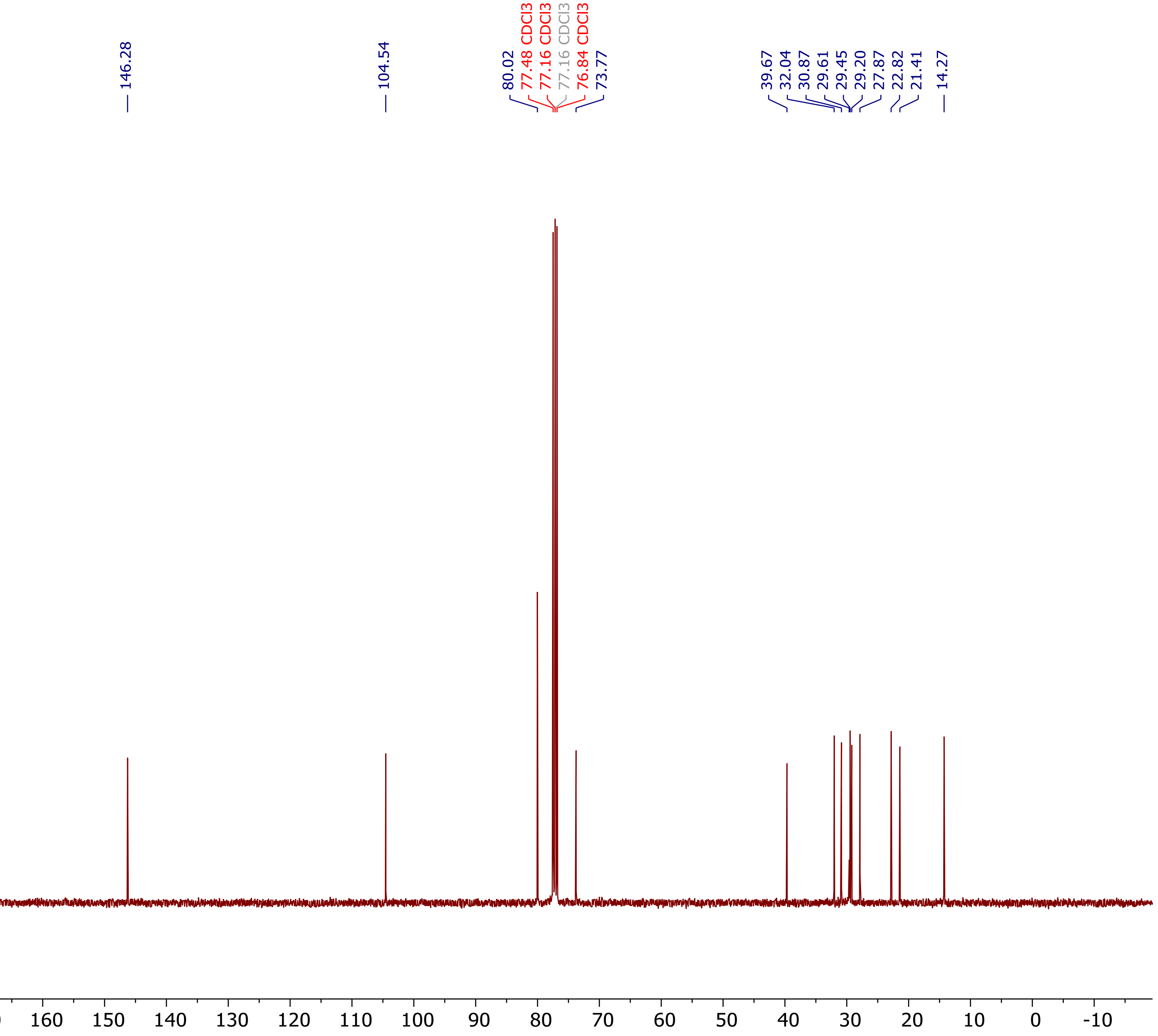

^{1}H NMR spectrum of **24** (100% Z, 400 MHz, $CDCl_3$)

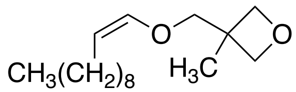

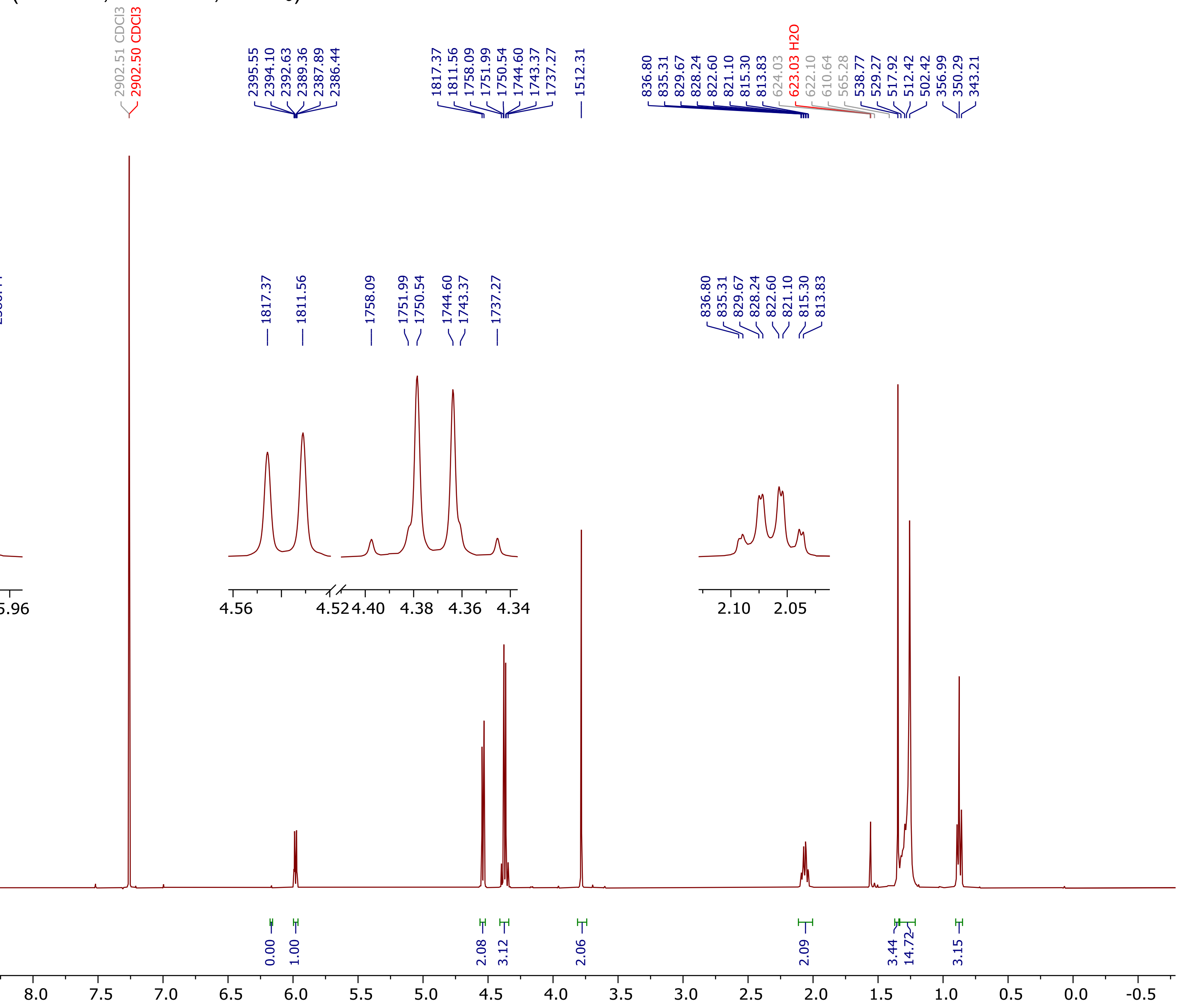

^{13}C NMR spectrum of **24** (100% Z, 101 MHz, $CDCl_3$)

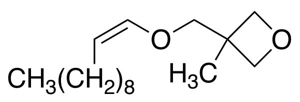

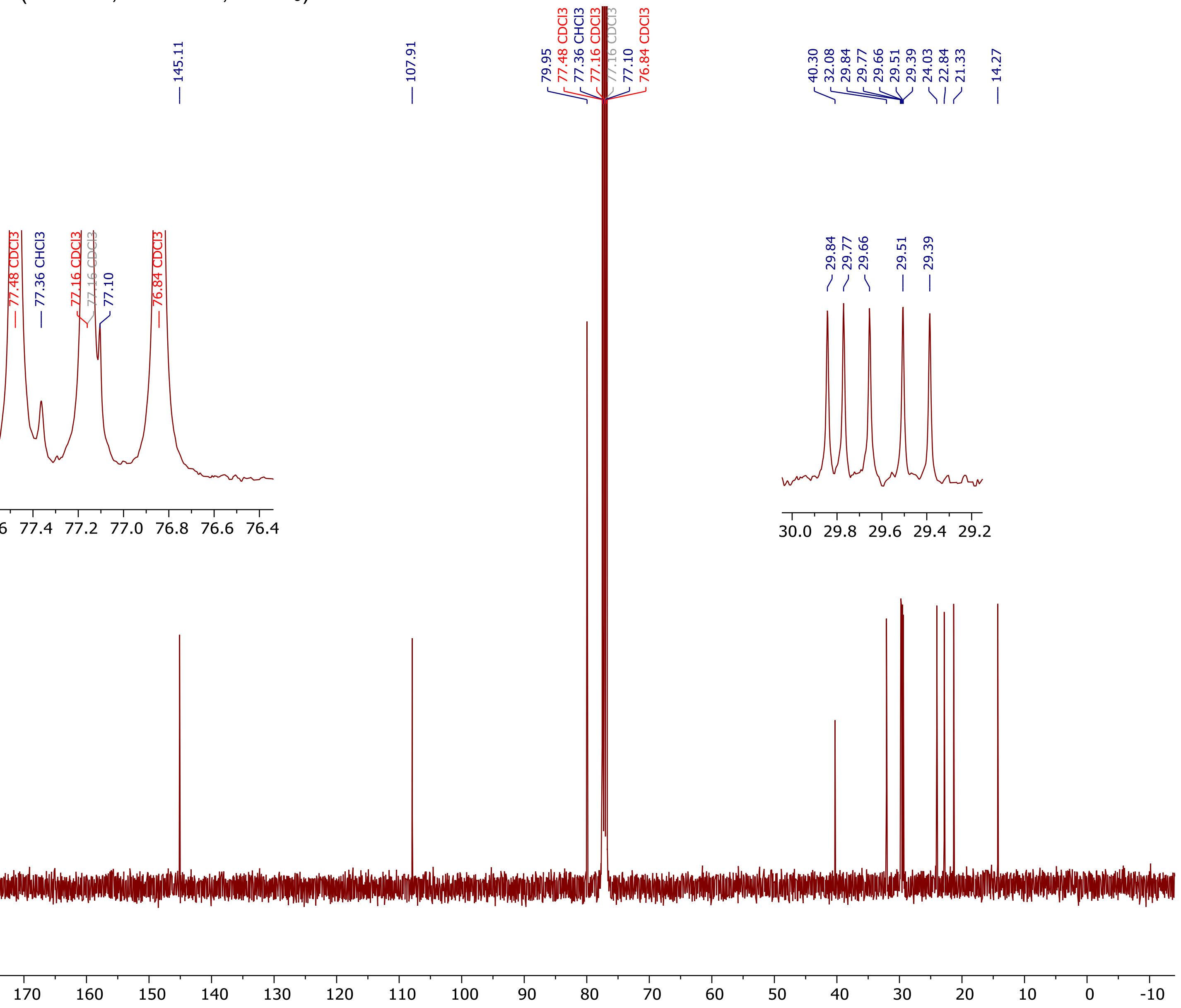

^{1}H NMR spectrum of **25** (400 MHz, $CDCl_3$)

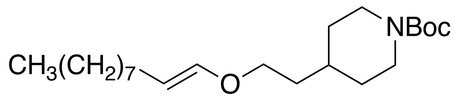

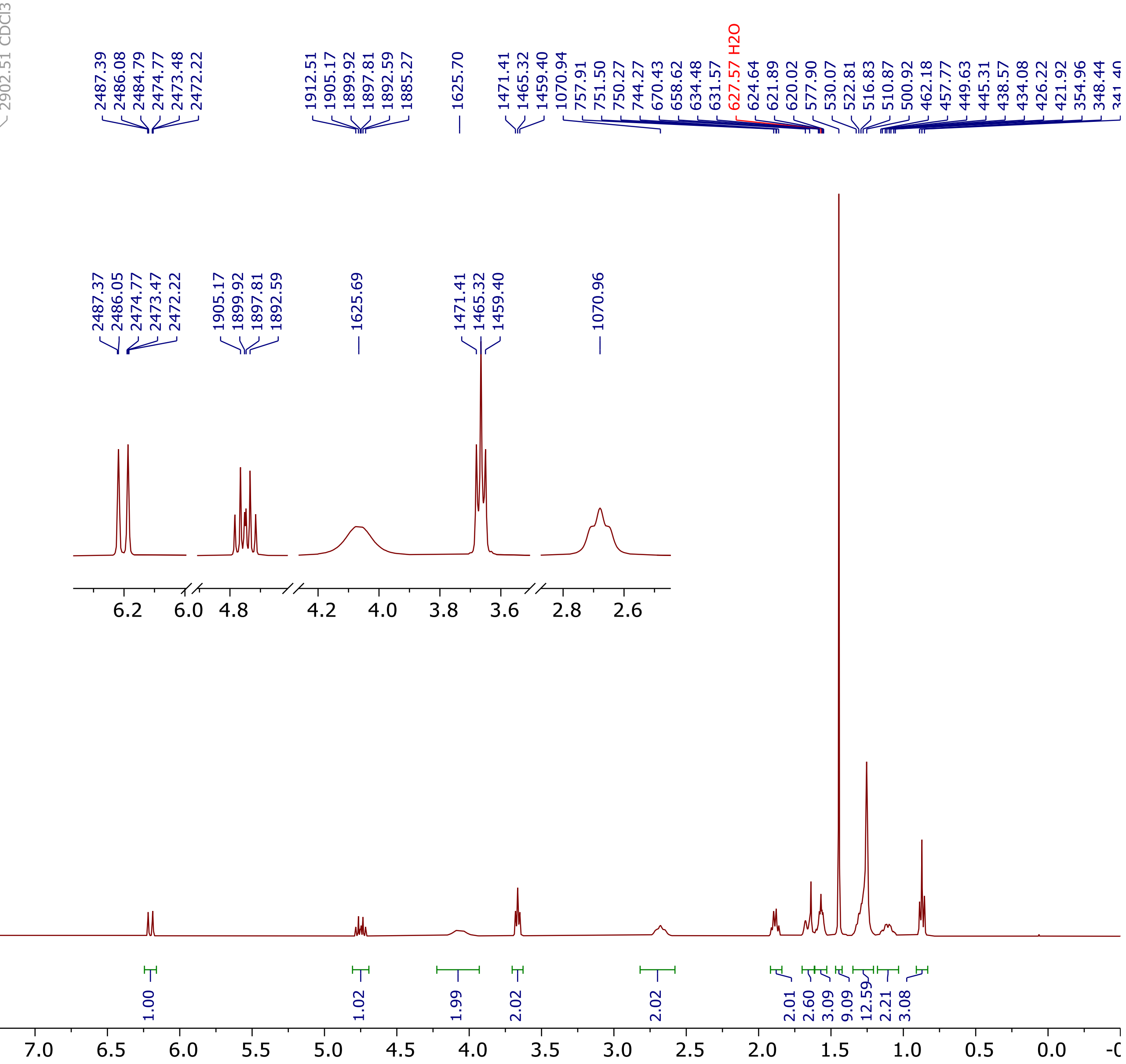

^{13}C NMR spectrum of **25** (101 MHz, $CDCl_3$)

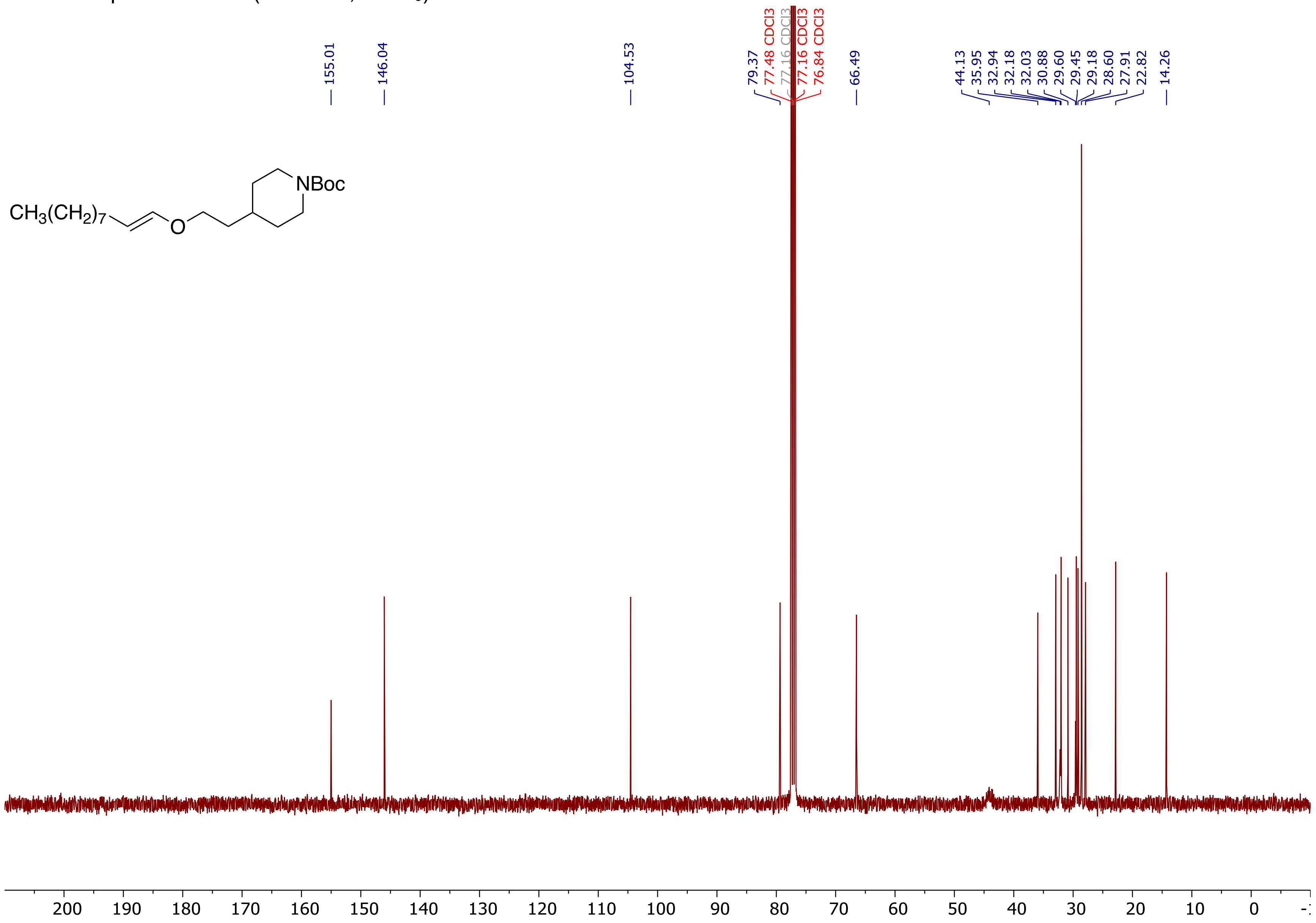

^{1}H NMR spectrum of **26** (20:1 Z/E, 600 MHz, $CDCl_3$)

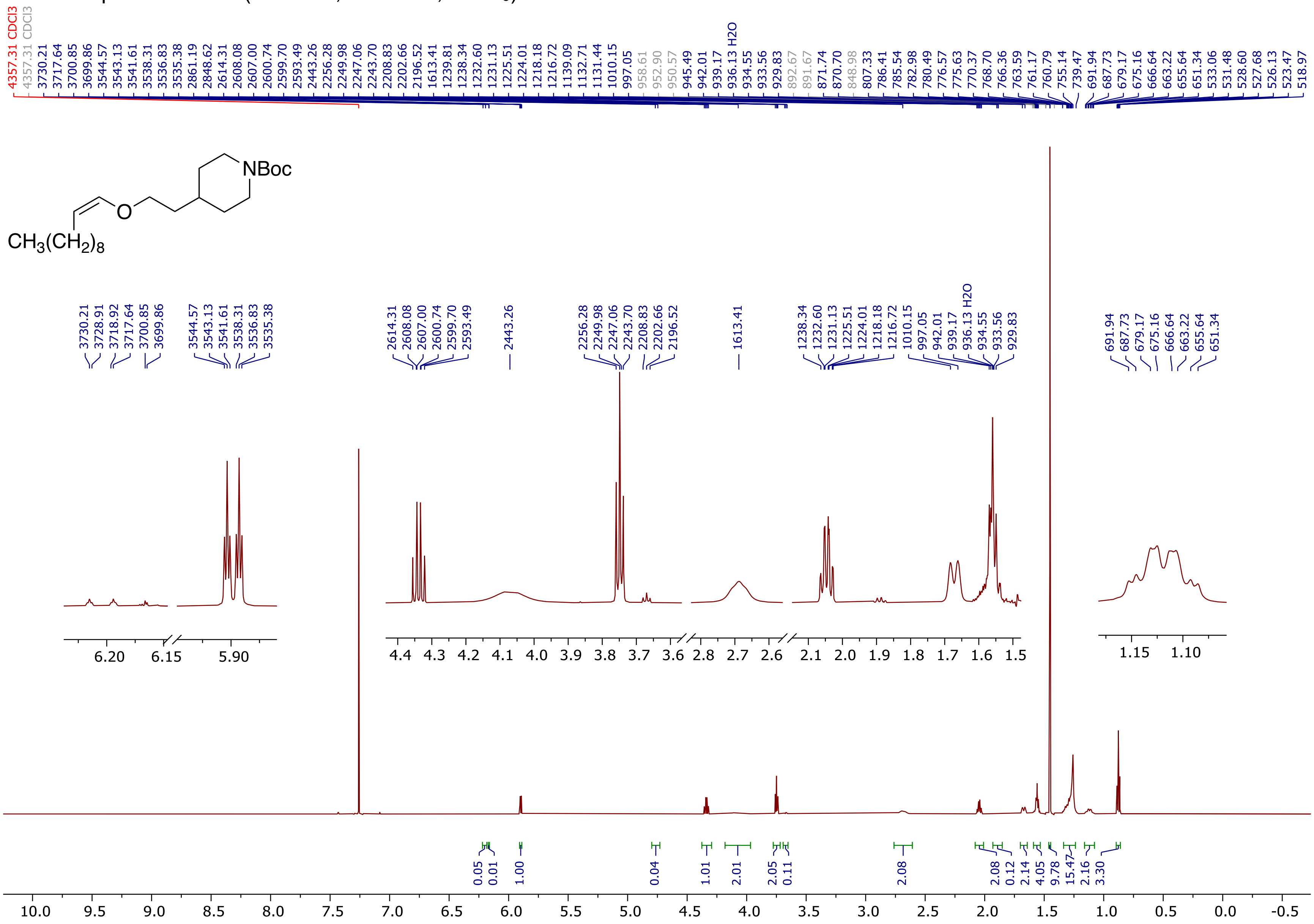

^{13}C NMR spectrum of **26** (20:1 Z/E, 151 MHz, $CDCl_3$)

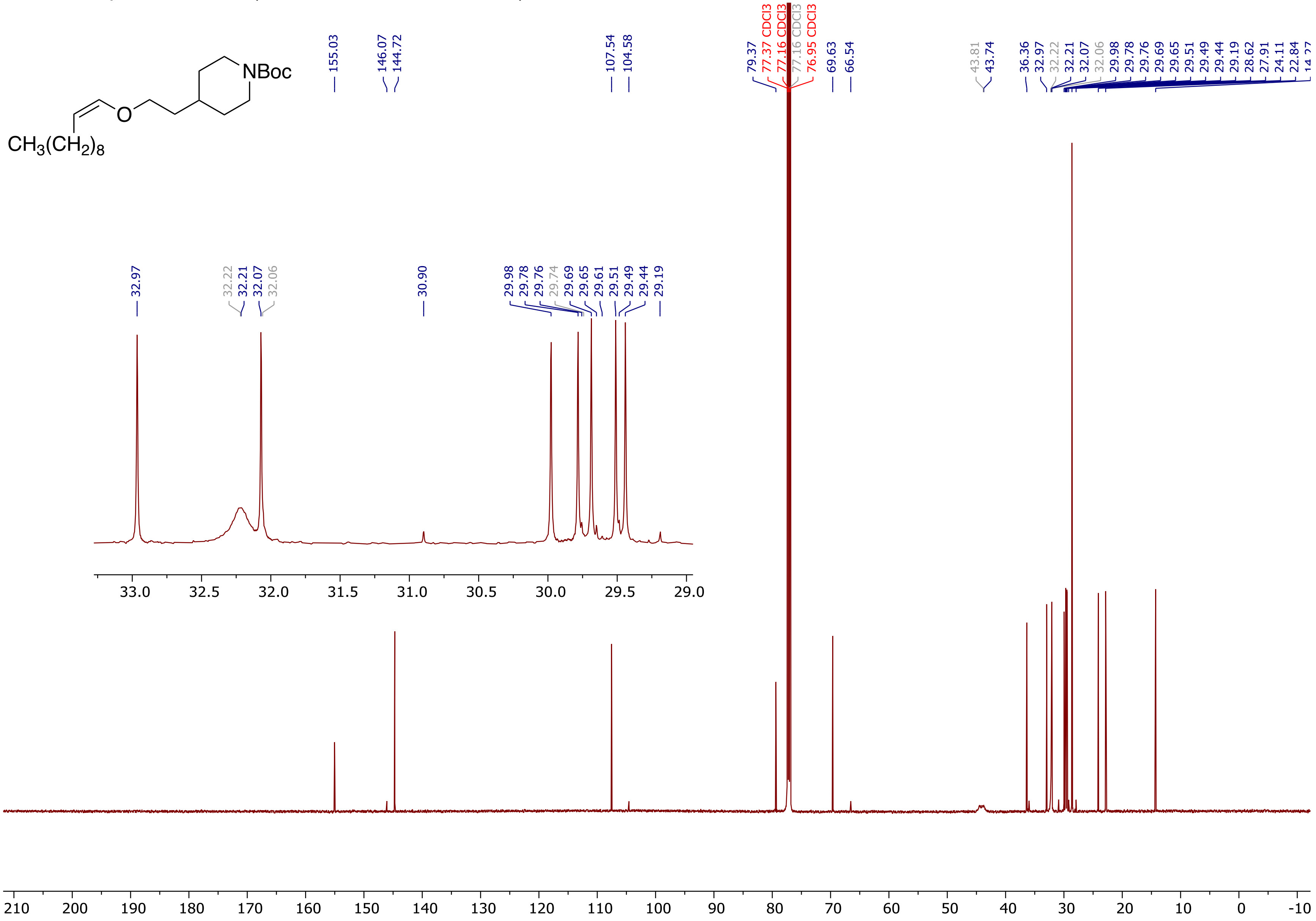

COSY spectrum of **26** (20:1 Z/E, 600 MHz, $CDCl_3$)

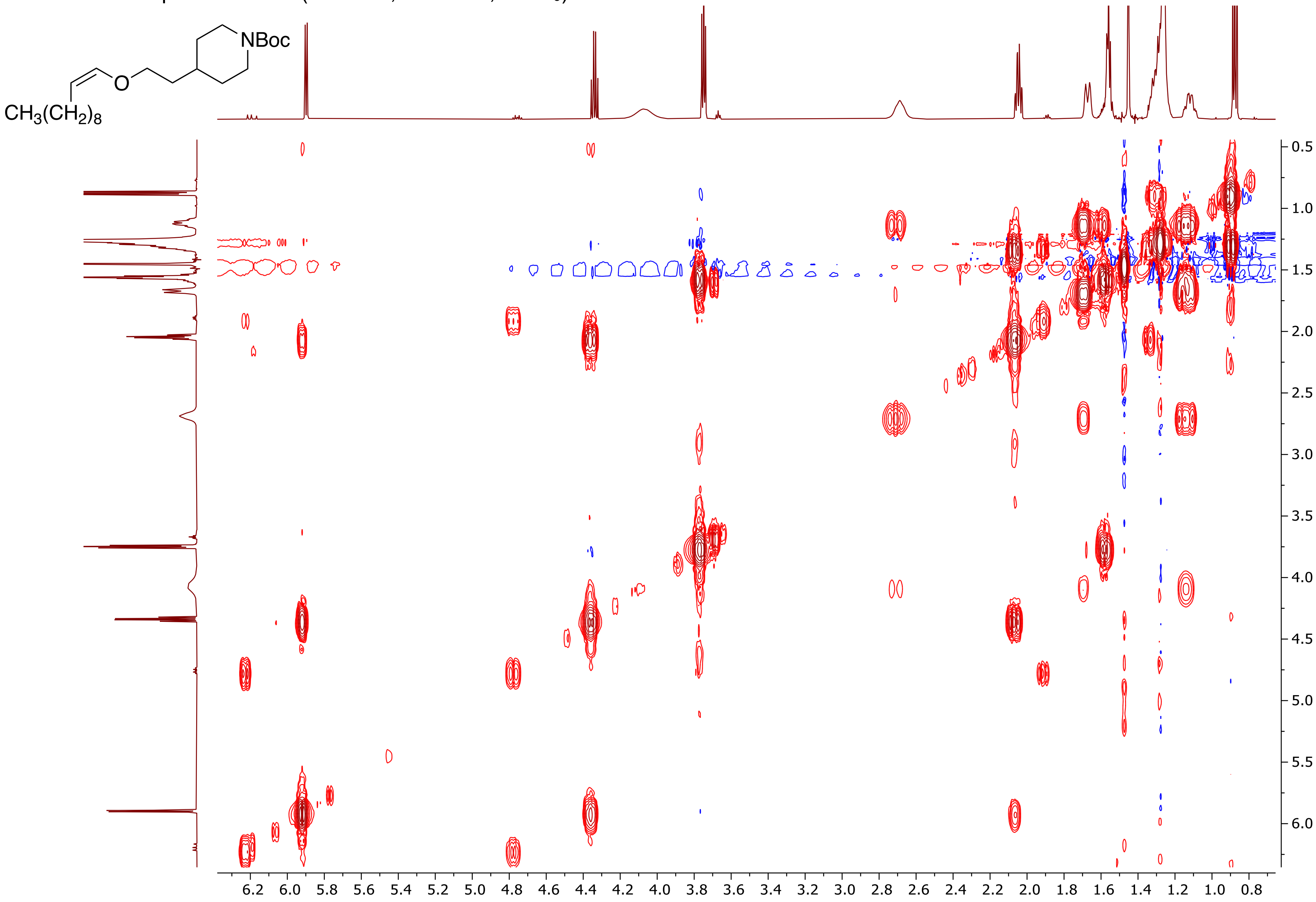

HSQC spectrum of **26** (20:1 Z/E, 600 MHz, $CDCl_3$)

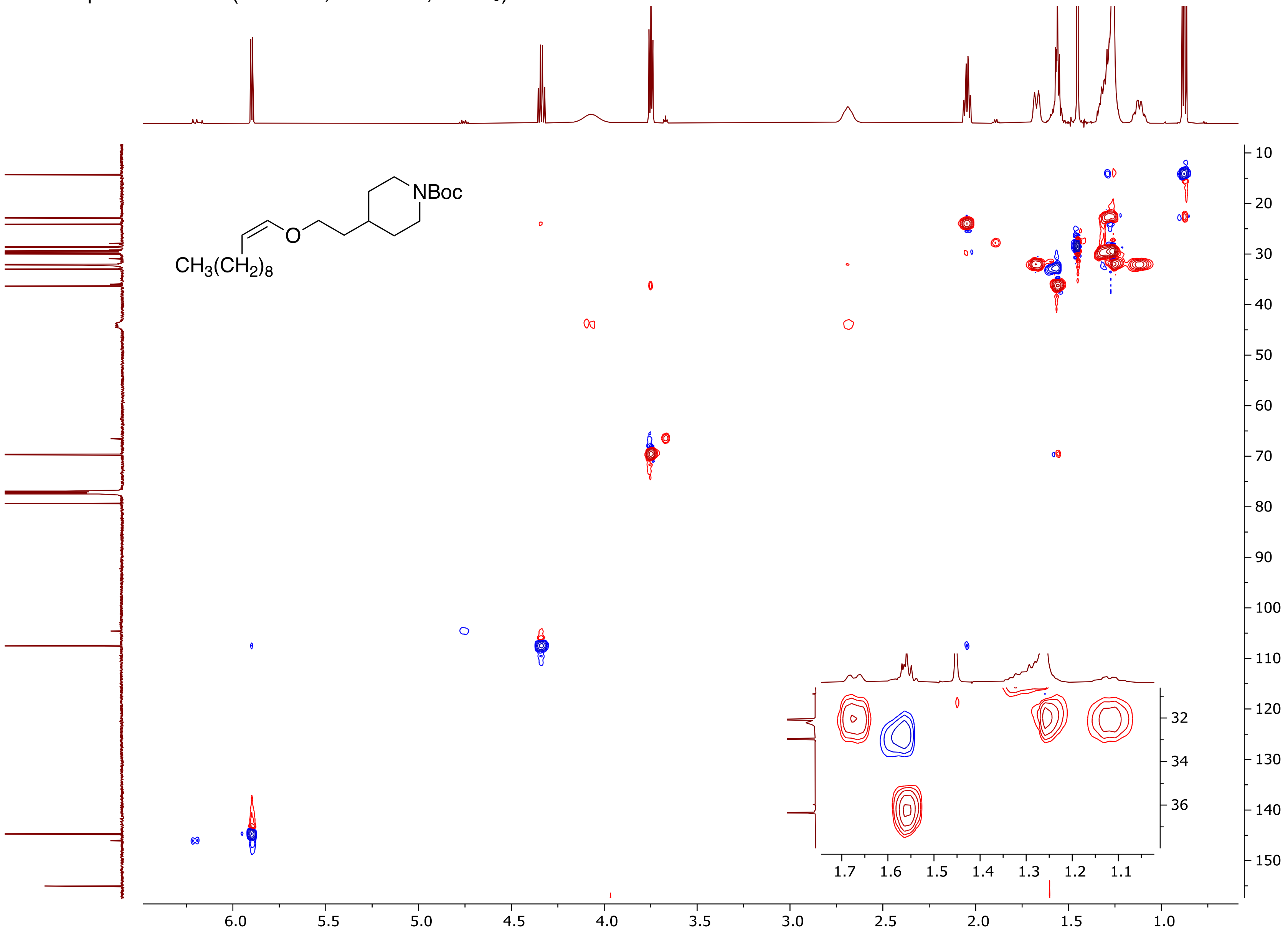

^{1}H NMR spectrum of **27** (400 MHz, $CDCl_3$)

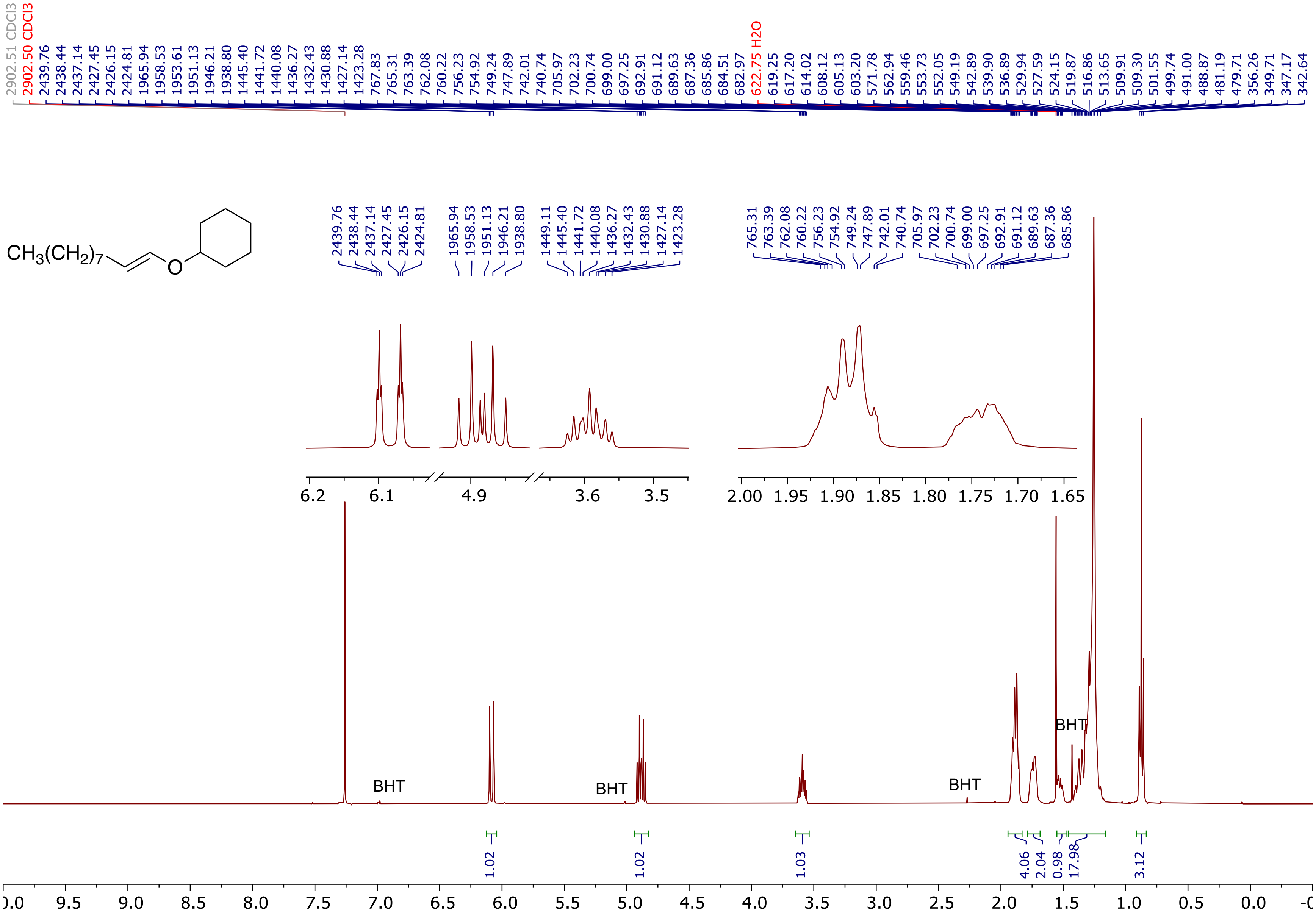

^{13}C NMR spectrum of **27** (101 MHz, $CDCl_3$)

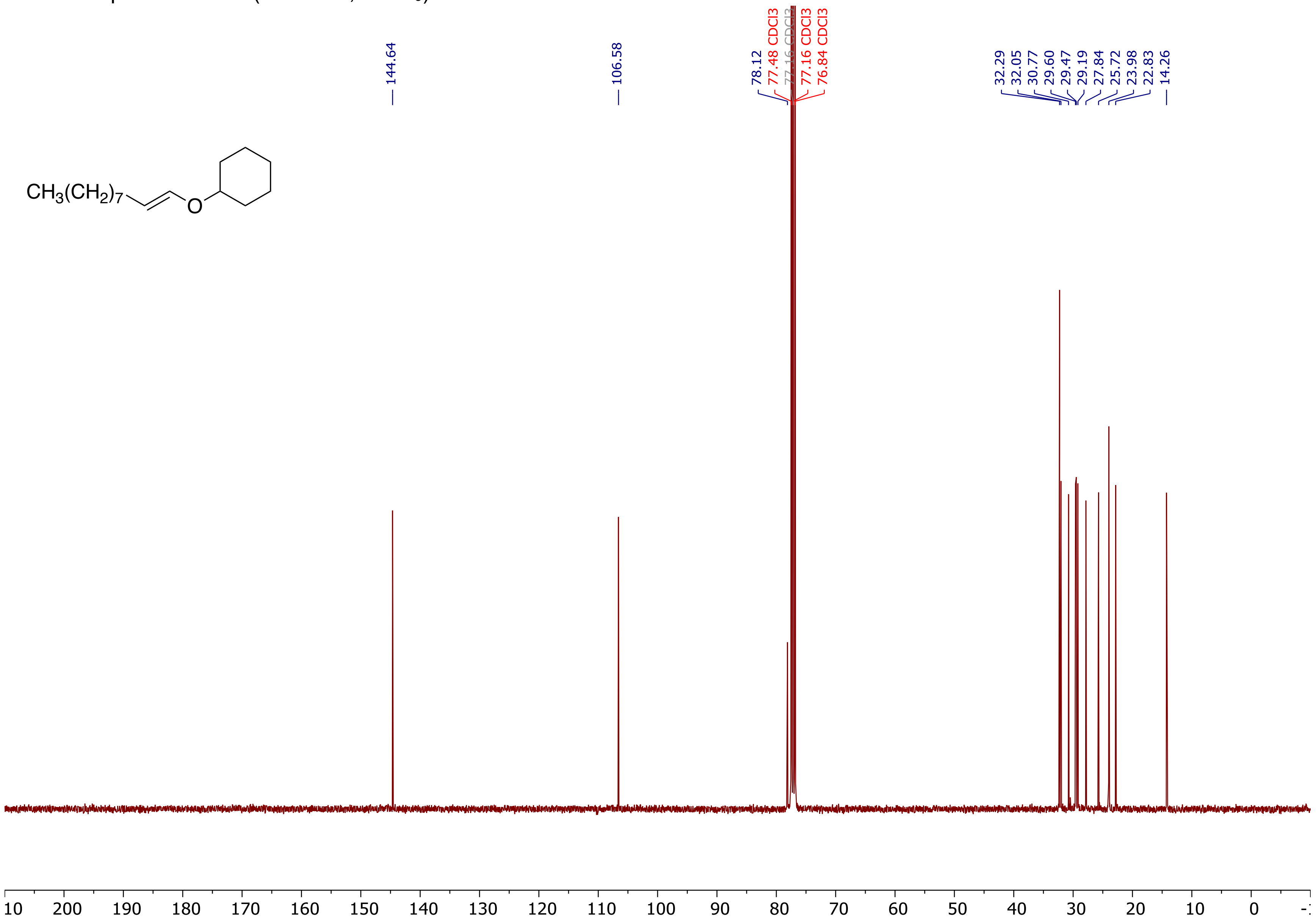

^1H NMR spectrum of **28** (100% Z, 400 MHz, $CDCl_3$)

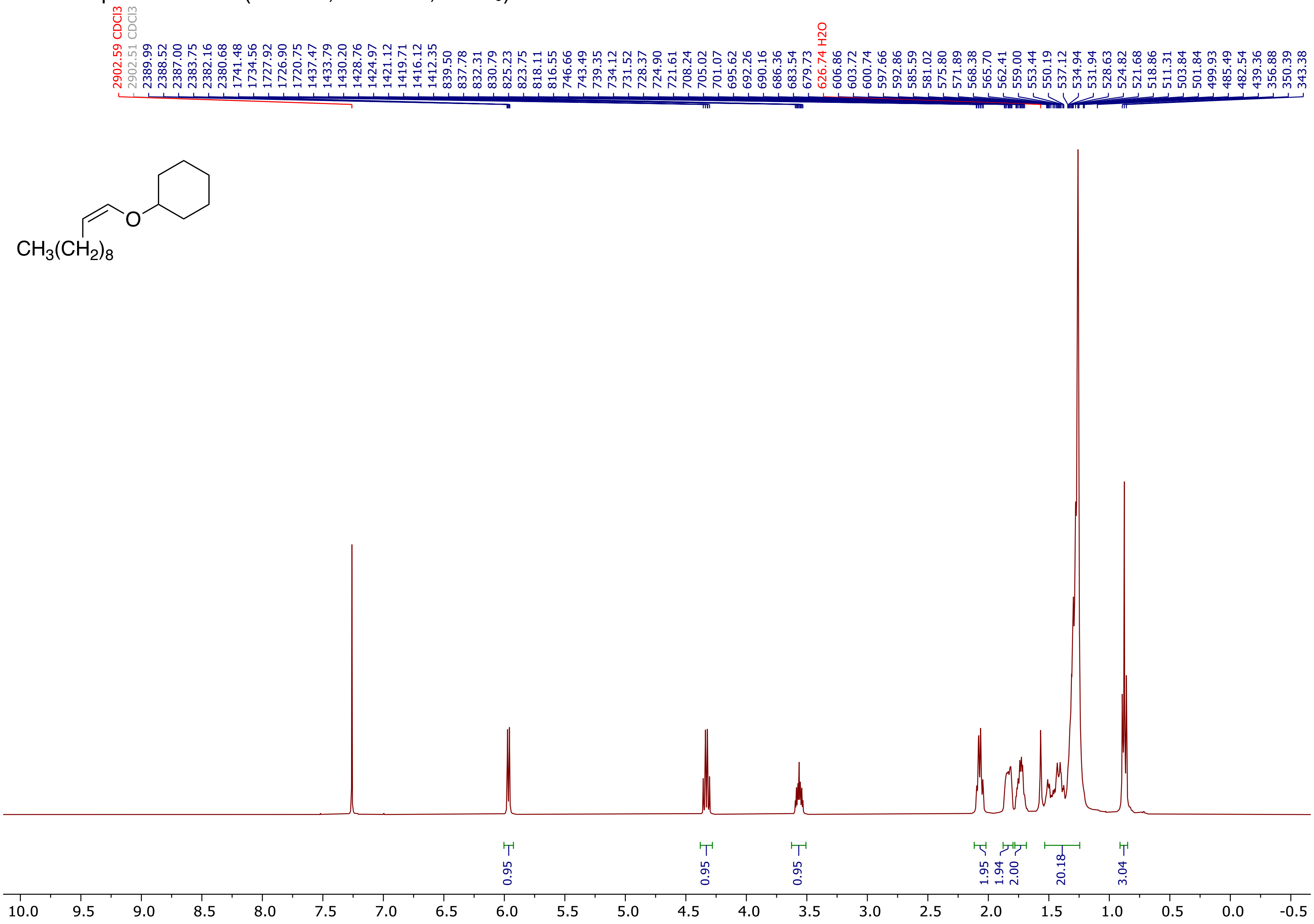

^{13}C NMR spectrum of **28** (100% Z, 101 MHz, $CDCl_3$)

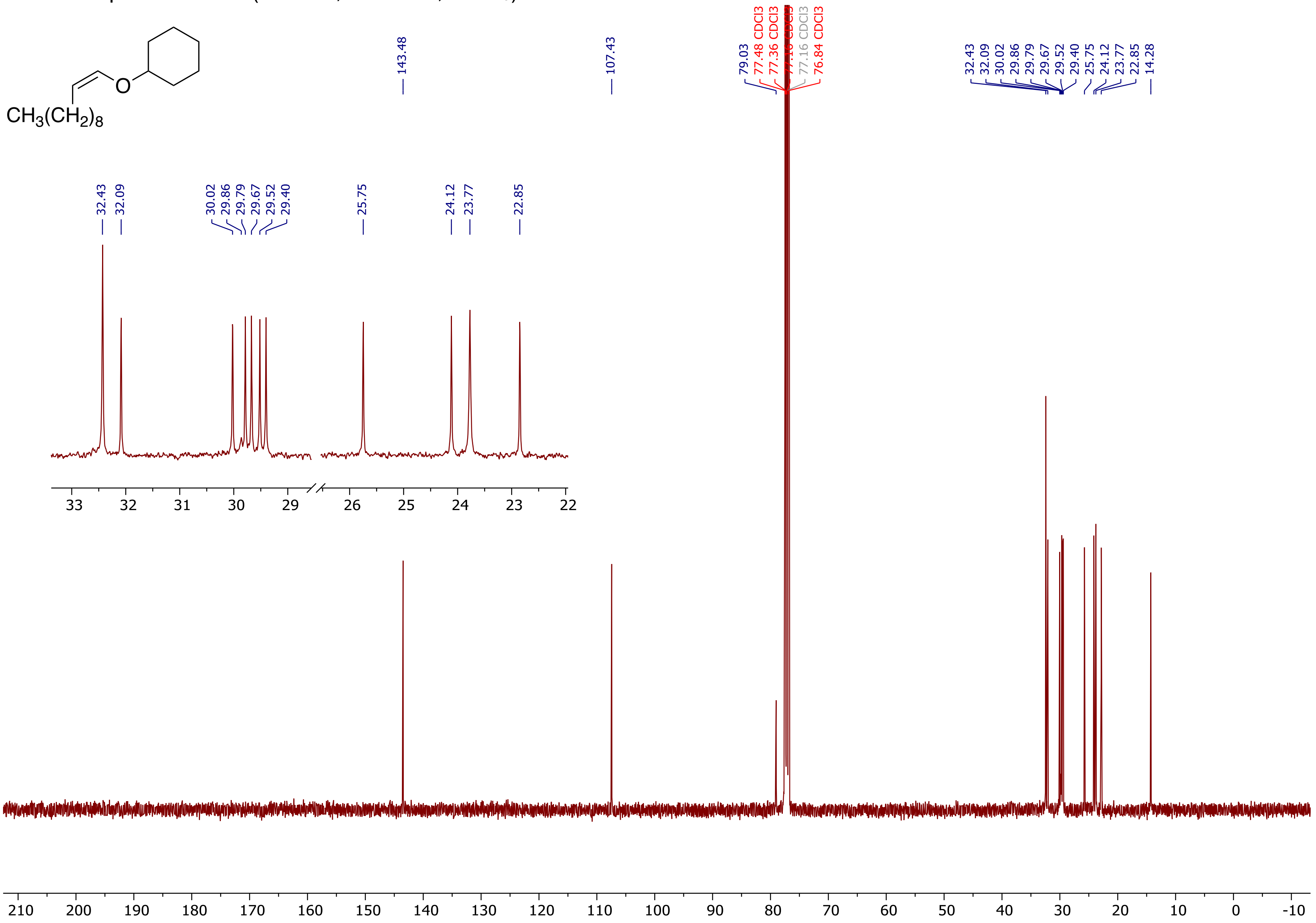

^{1}H NMR spectrum of **29** (400 MHz, $CDCl_3$)

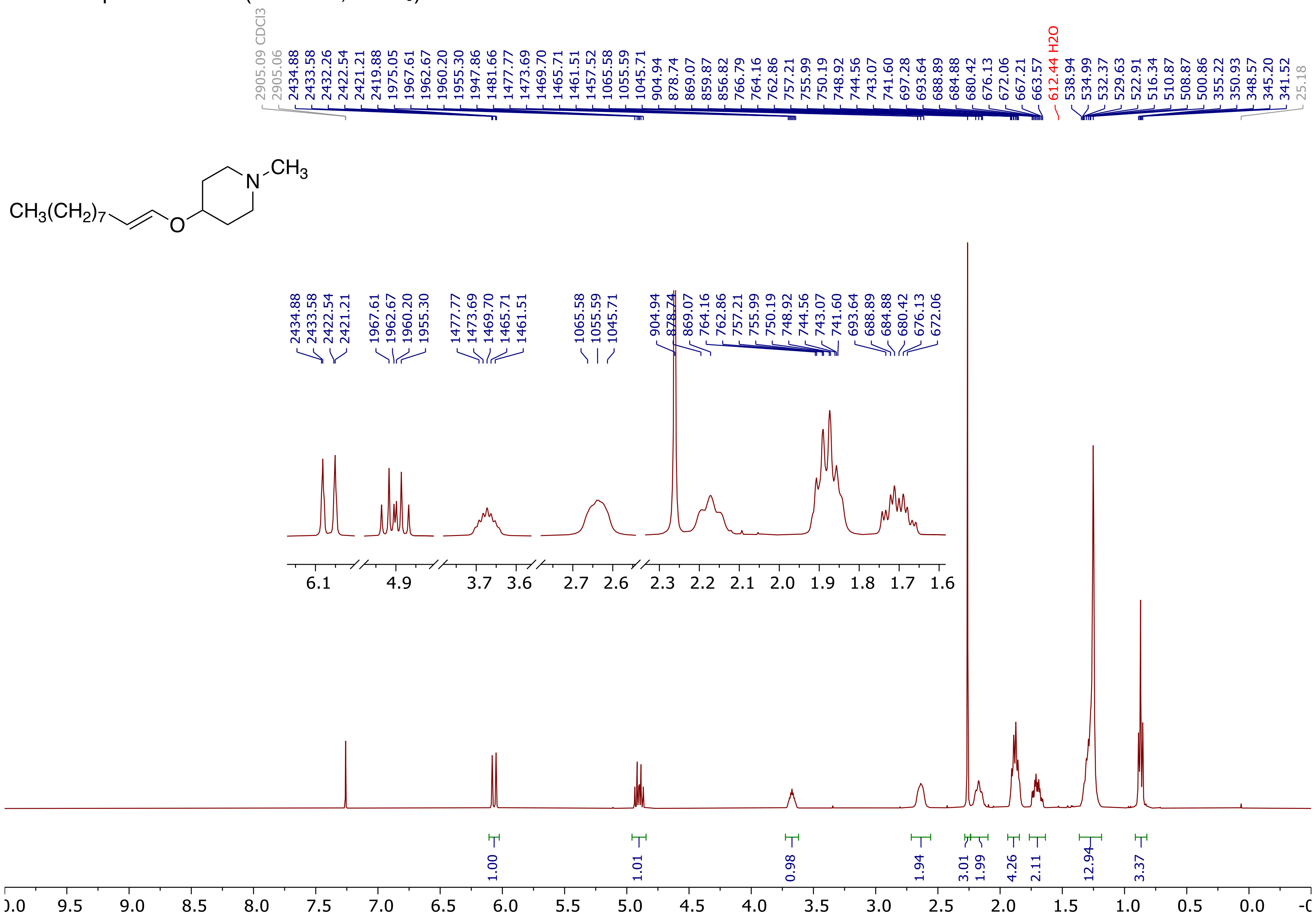

^{13}C NMR spectrum of **29** (101 MHz, $CDCl_3$)

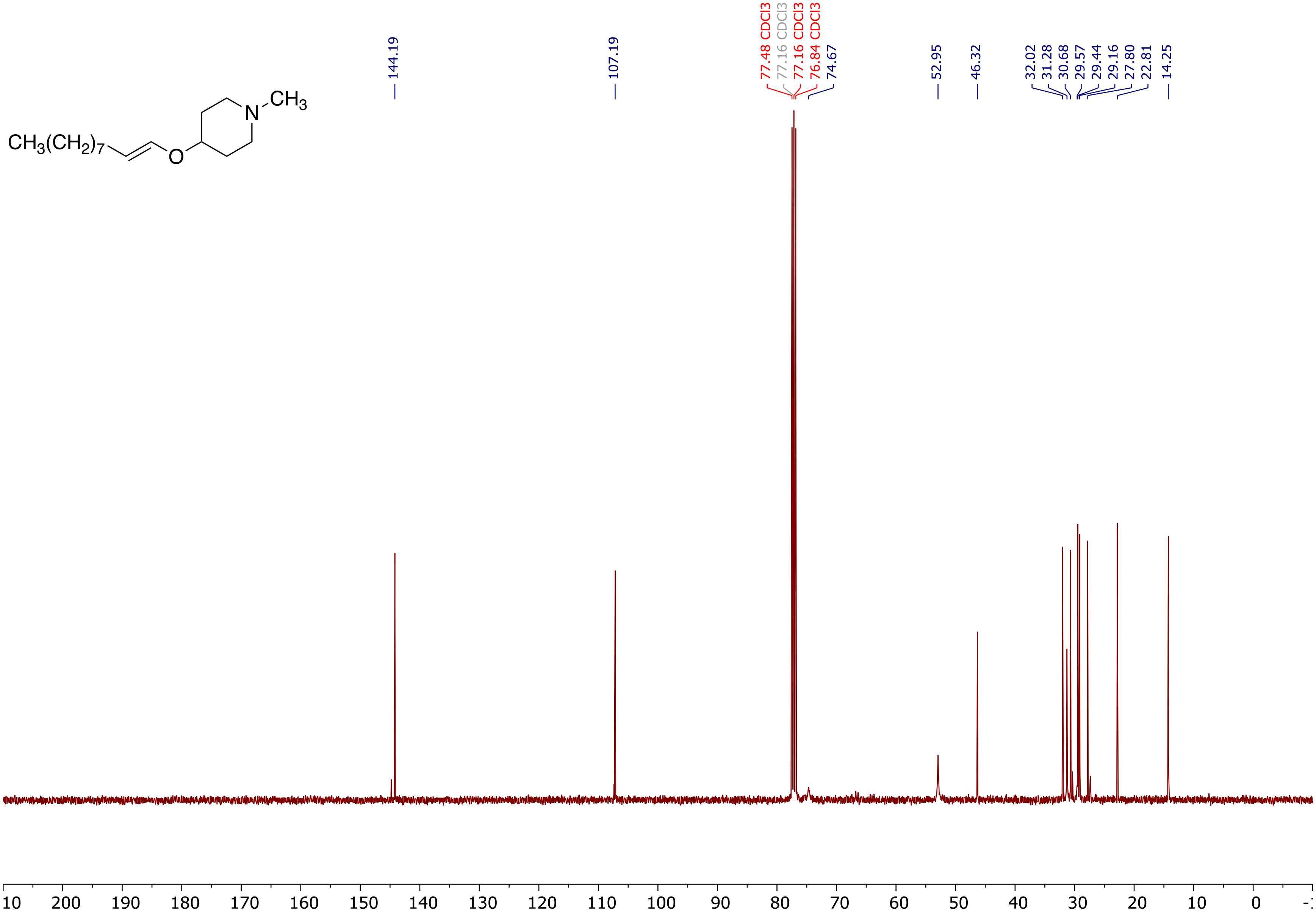

^{1}H NMR spectrum of **30** (13:1 Z/E, 400 MHz, $CDCl_3$)

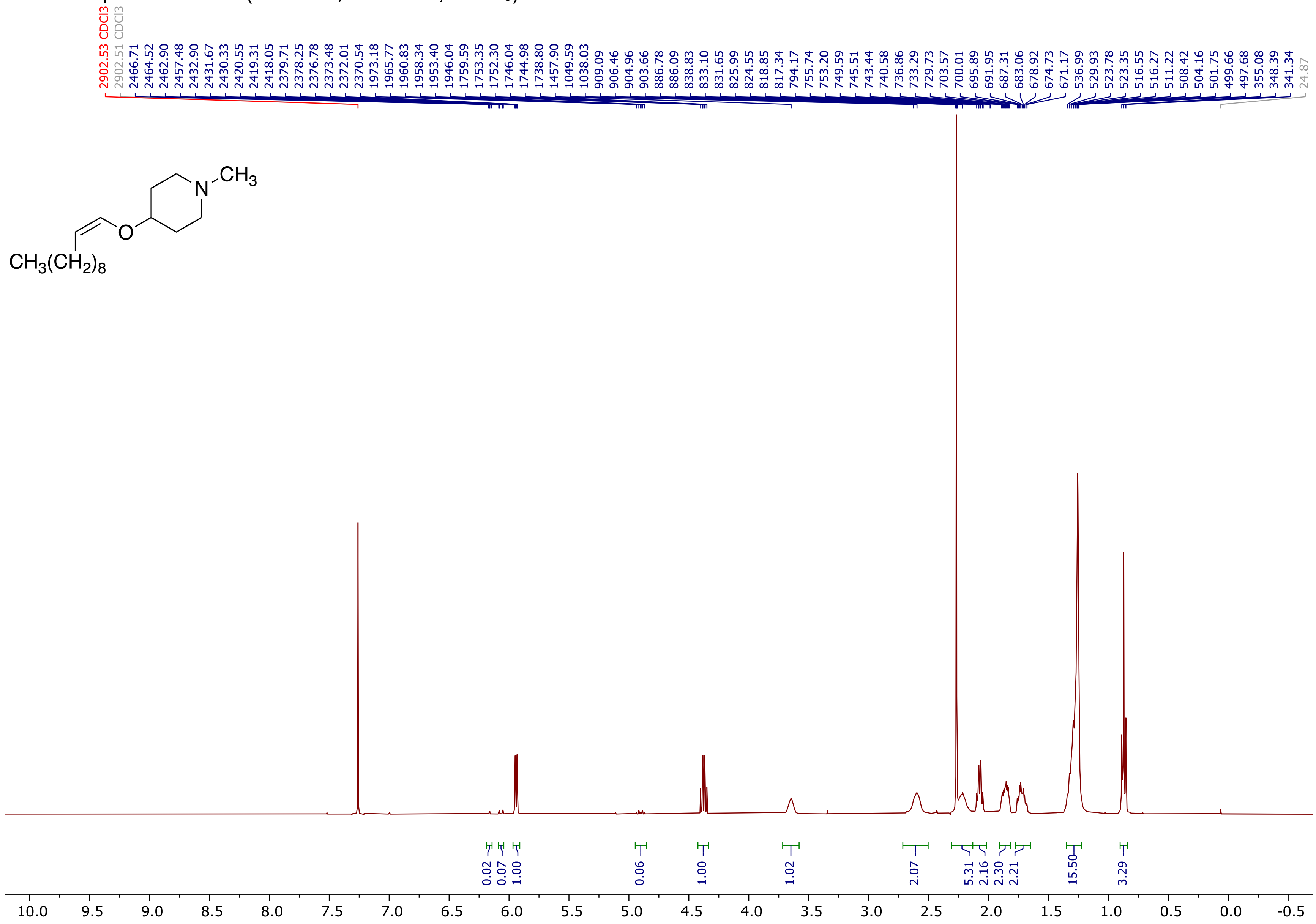

^{13}C NMR spectrum of **30** (13:1 Z/E, 101 MHz, $CDCl_3$)

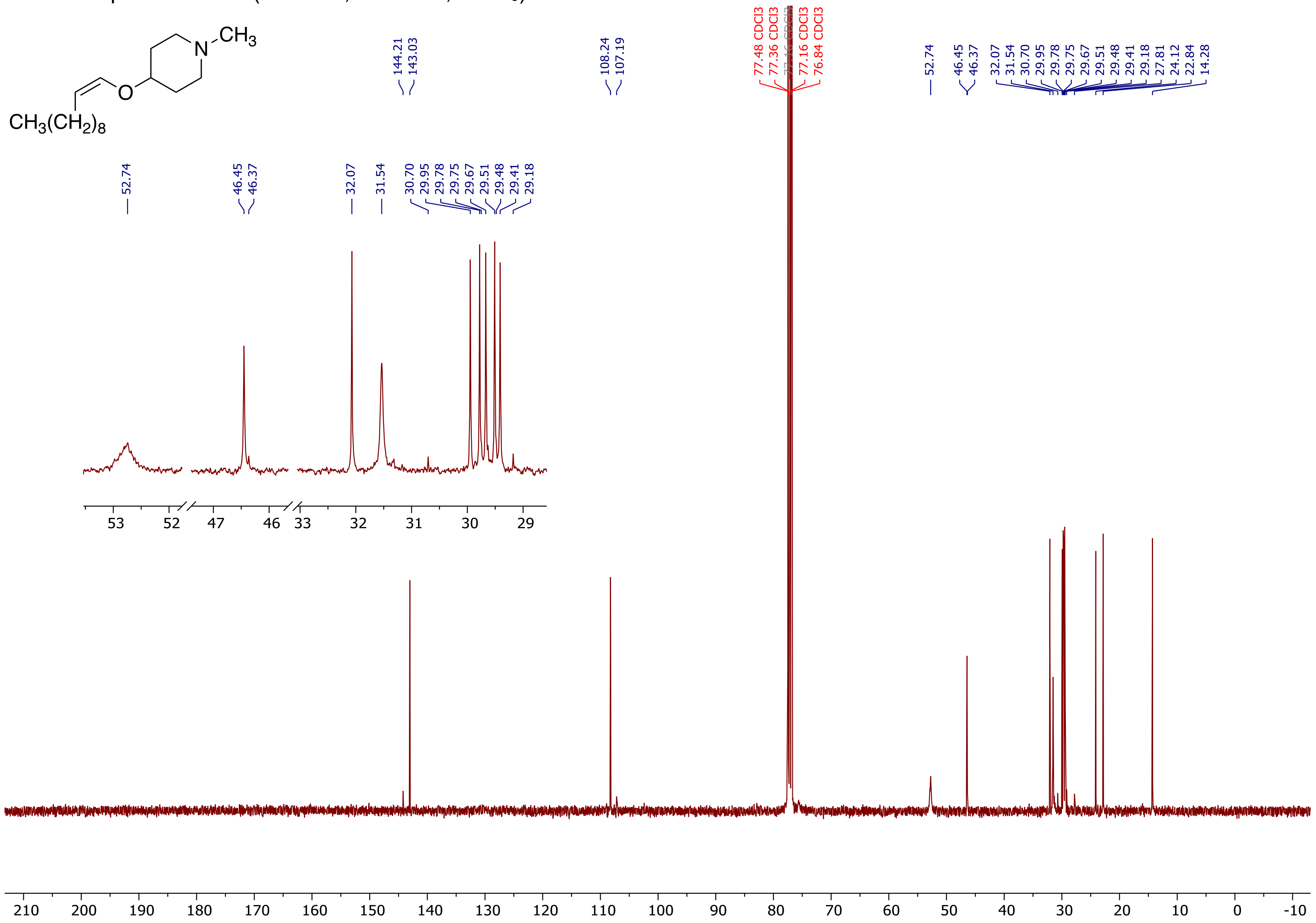

^1H NMR spectrum of **31** (400 MHz, $CDCl_3$)

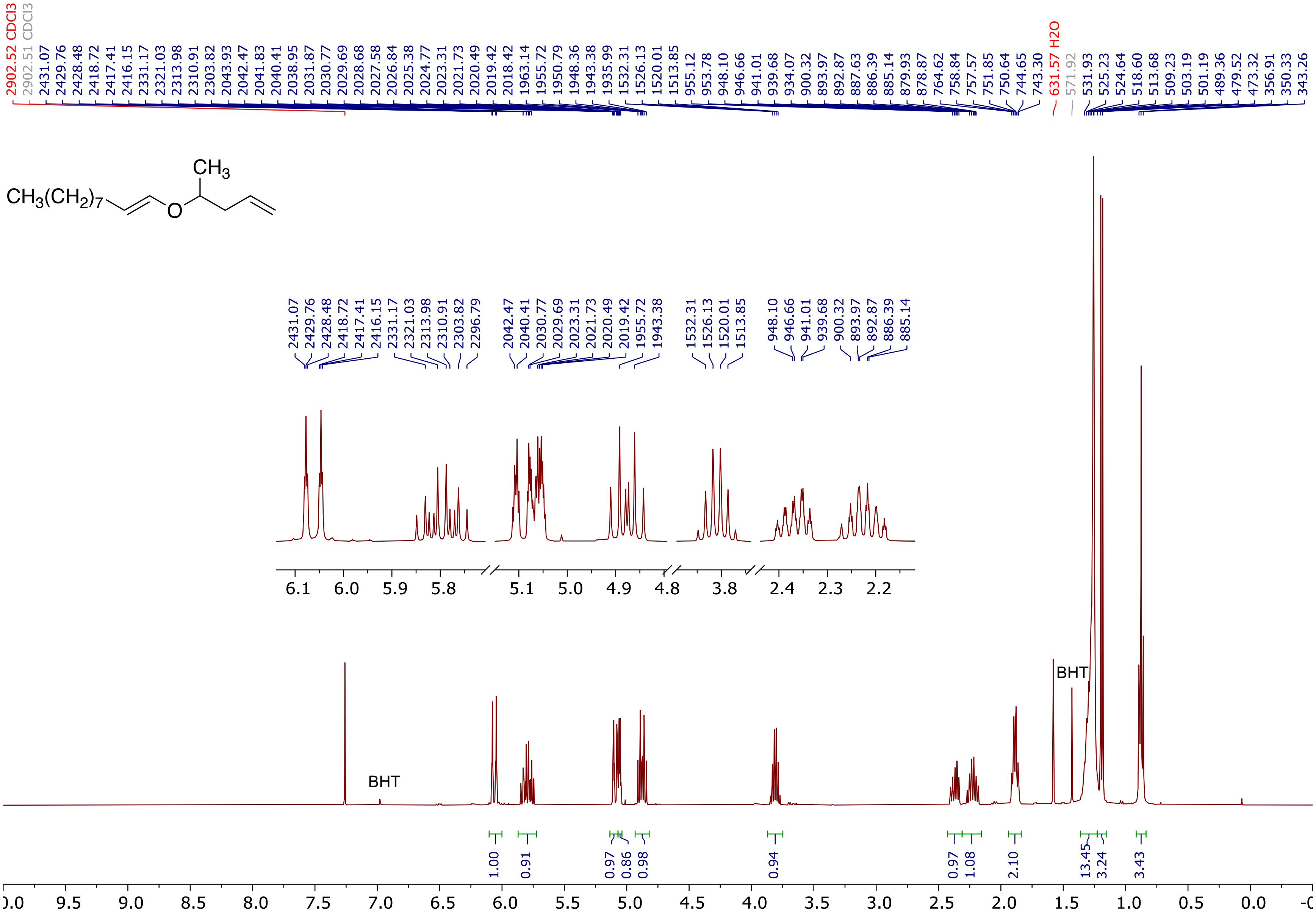

^{13}C NMR spectrum of **31** (101 MHz, $CDCl_3$)

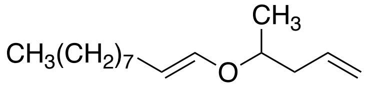

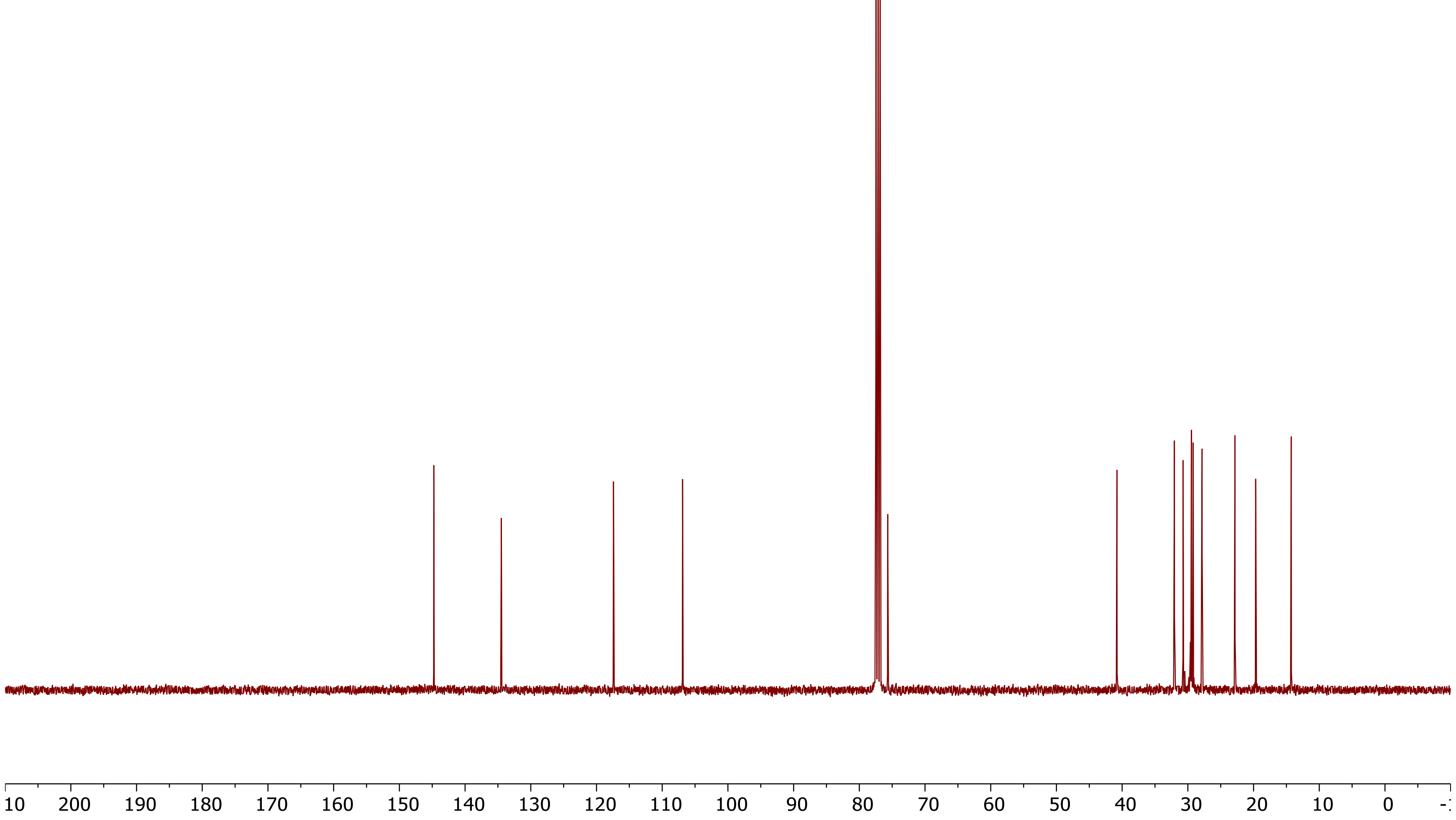

^{1}H NMR spectrum of **32** (>20:1 Z/E, 400 MHz, $CDCl_3$)

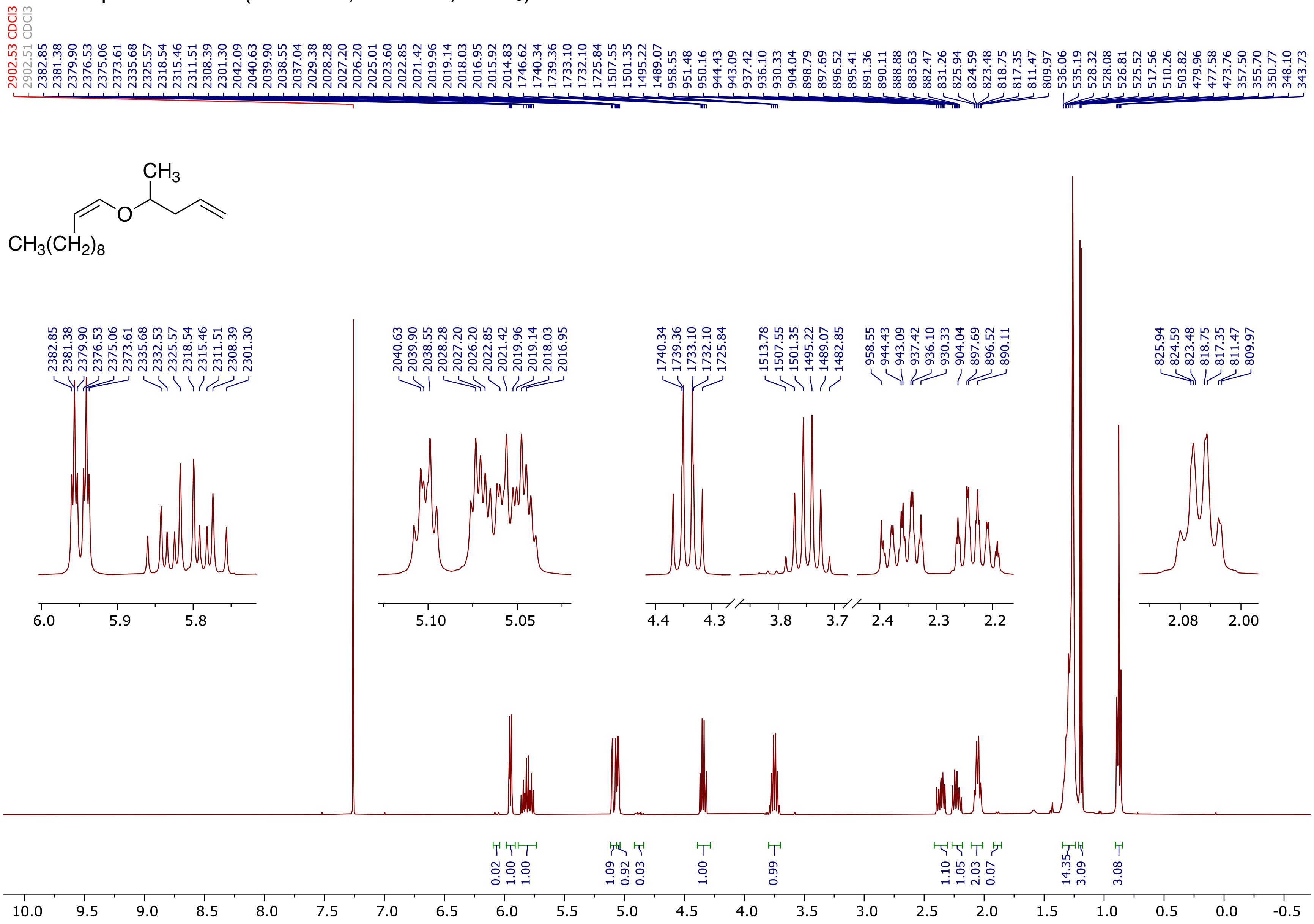

^{13}C NMR spectrum of **32** (>20:1 Z/E, 101 MHz, $CDCl_3$)

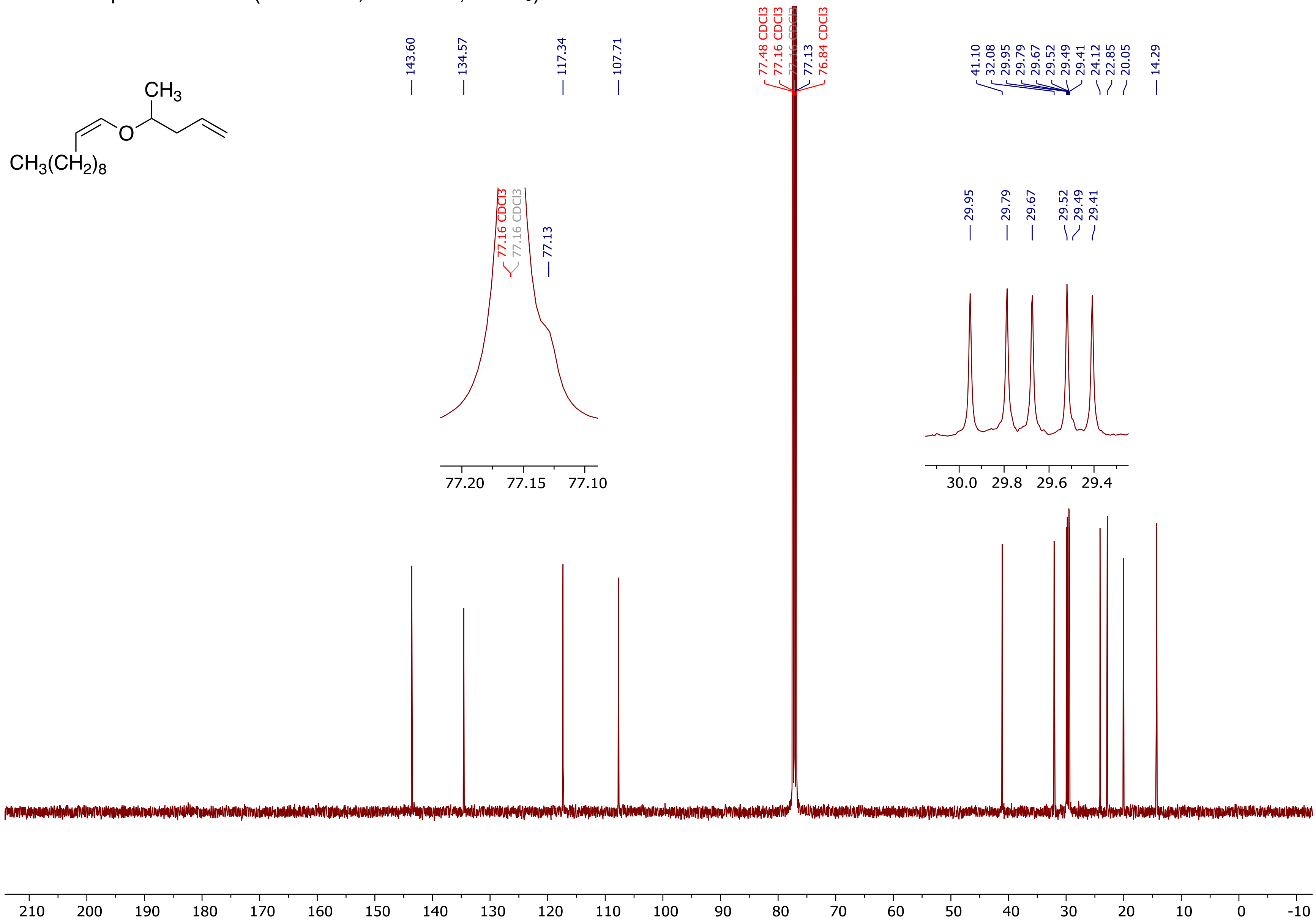

^{1}H NMR spectrum of **33** (400 MHz, $CDCl_3$)

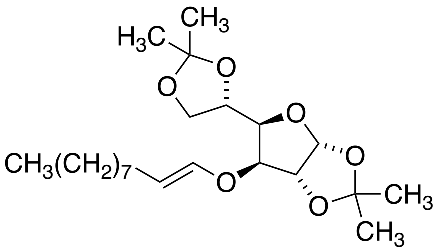

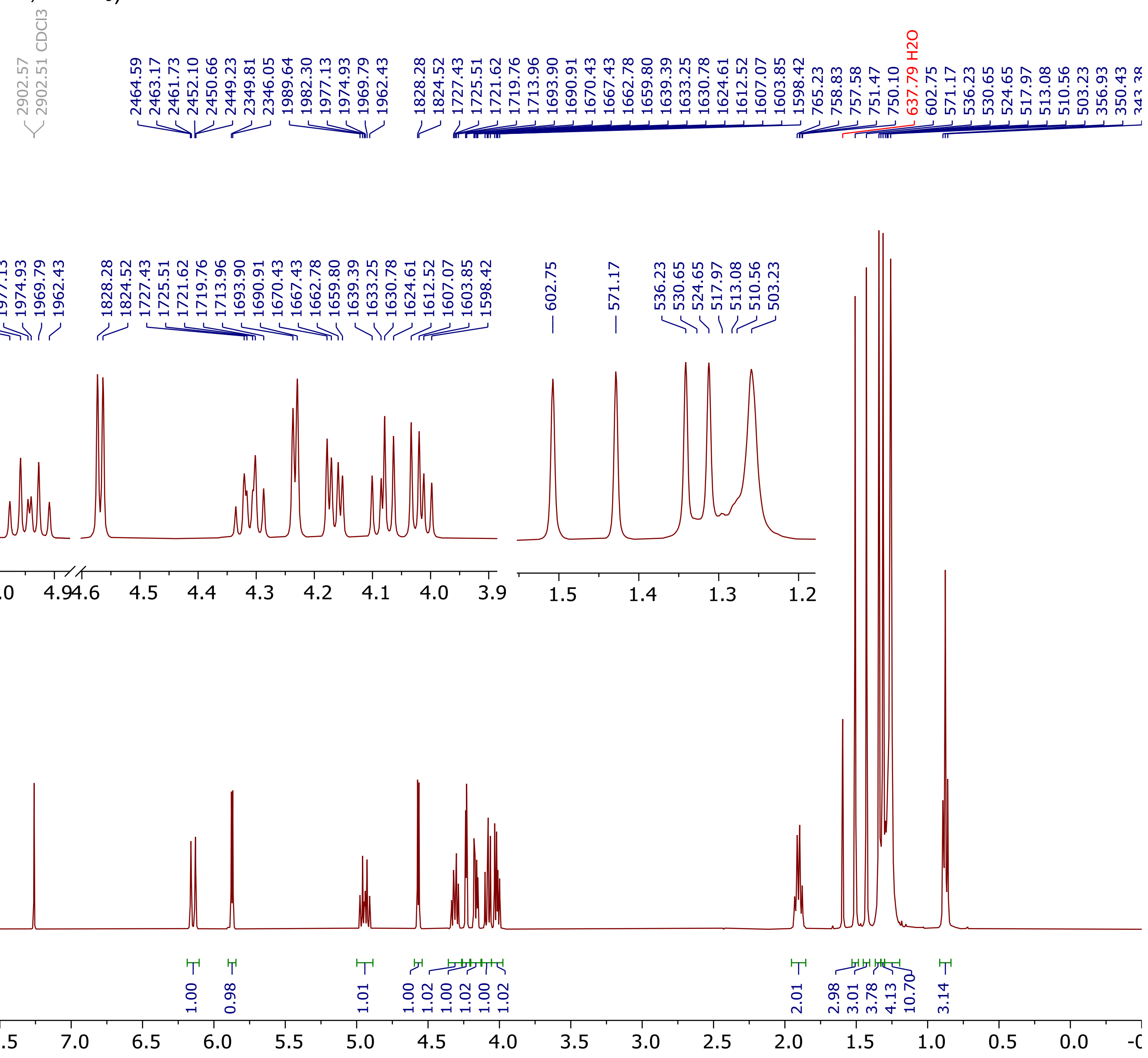

^{13}C NMR spectrum of **33** (101 MHz, $CDCl_3$)

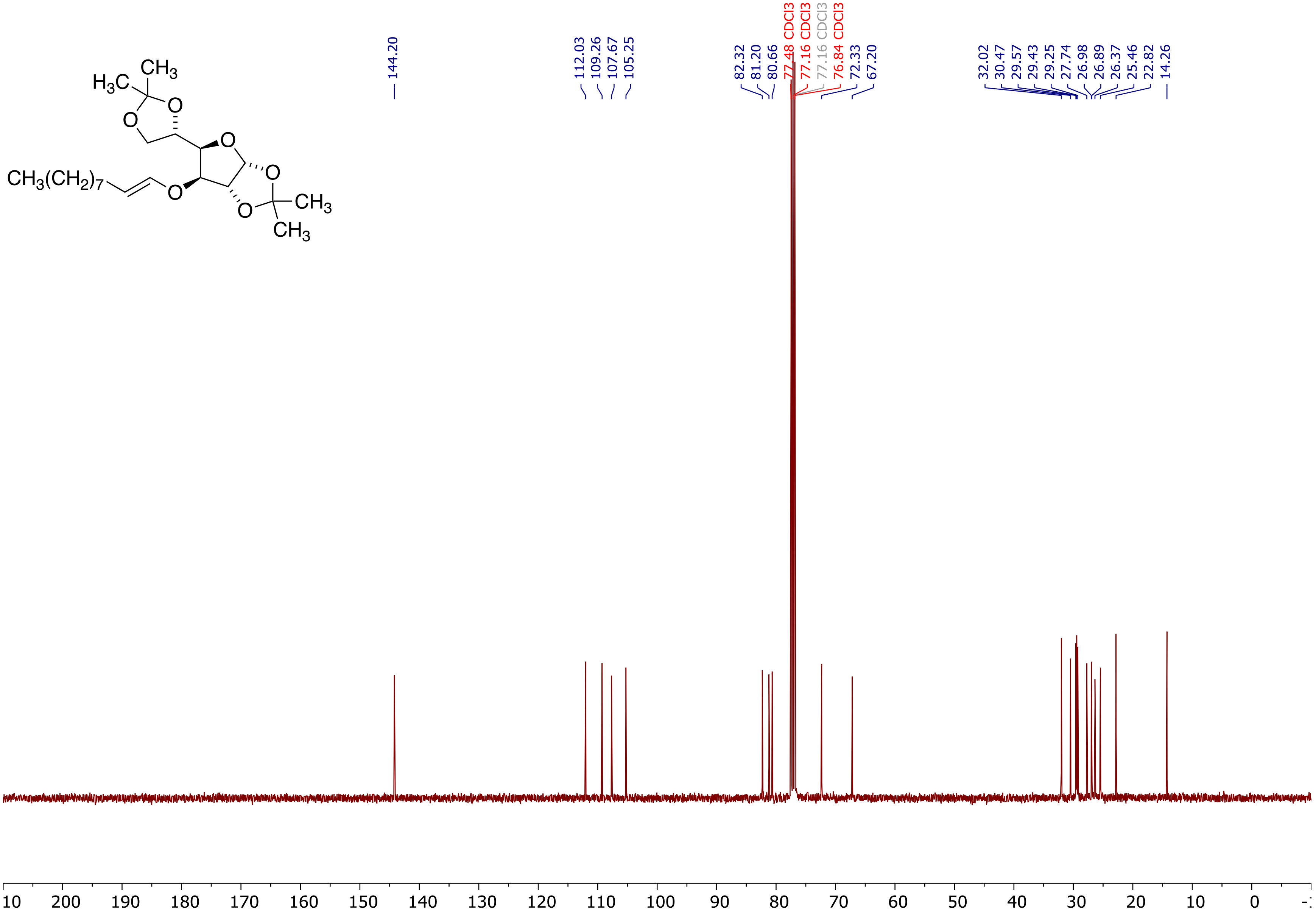

COSY spectrum of **33** (600 MHz, $CDCl_3$)

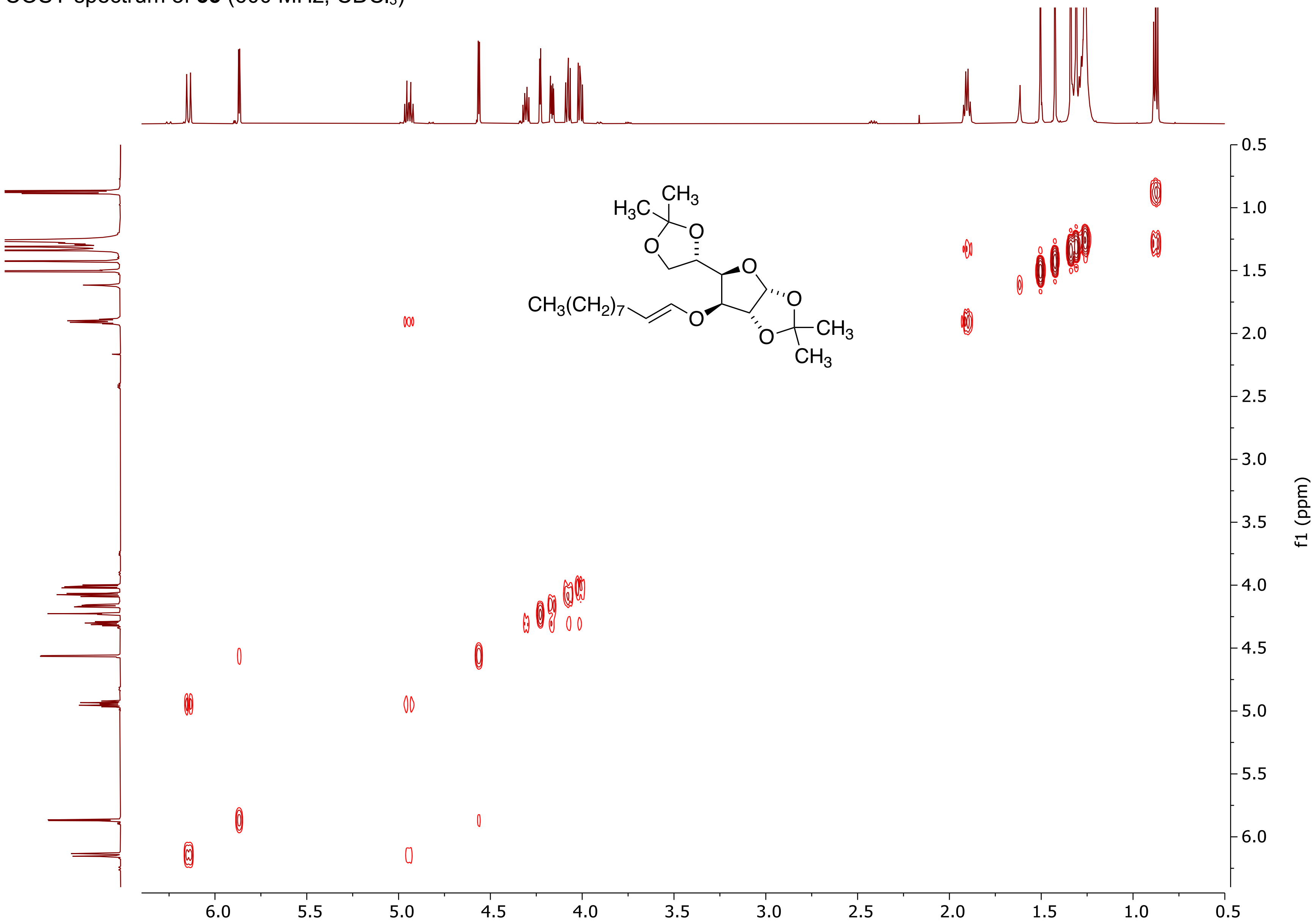

COSY spectrum of **33** (600 MHz, $CDCl_3$) expansion

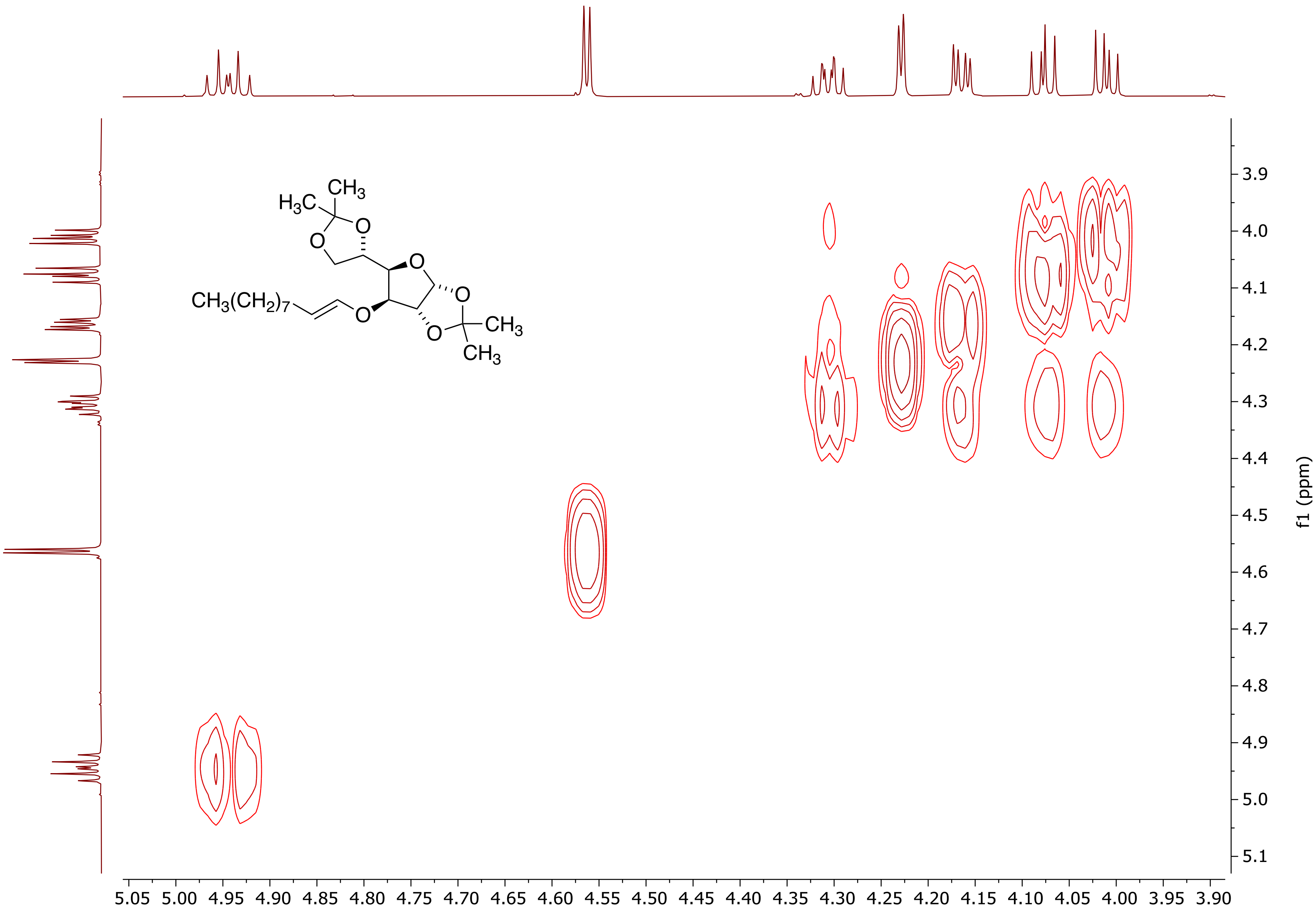

HSQC spectrum of **33** (600 MHz, $CDCl_3$)

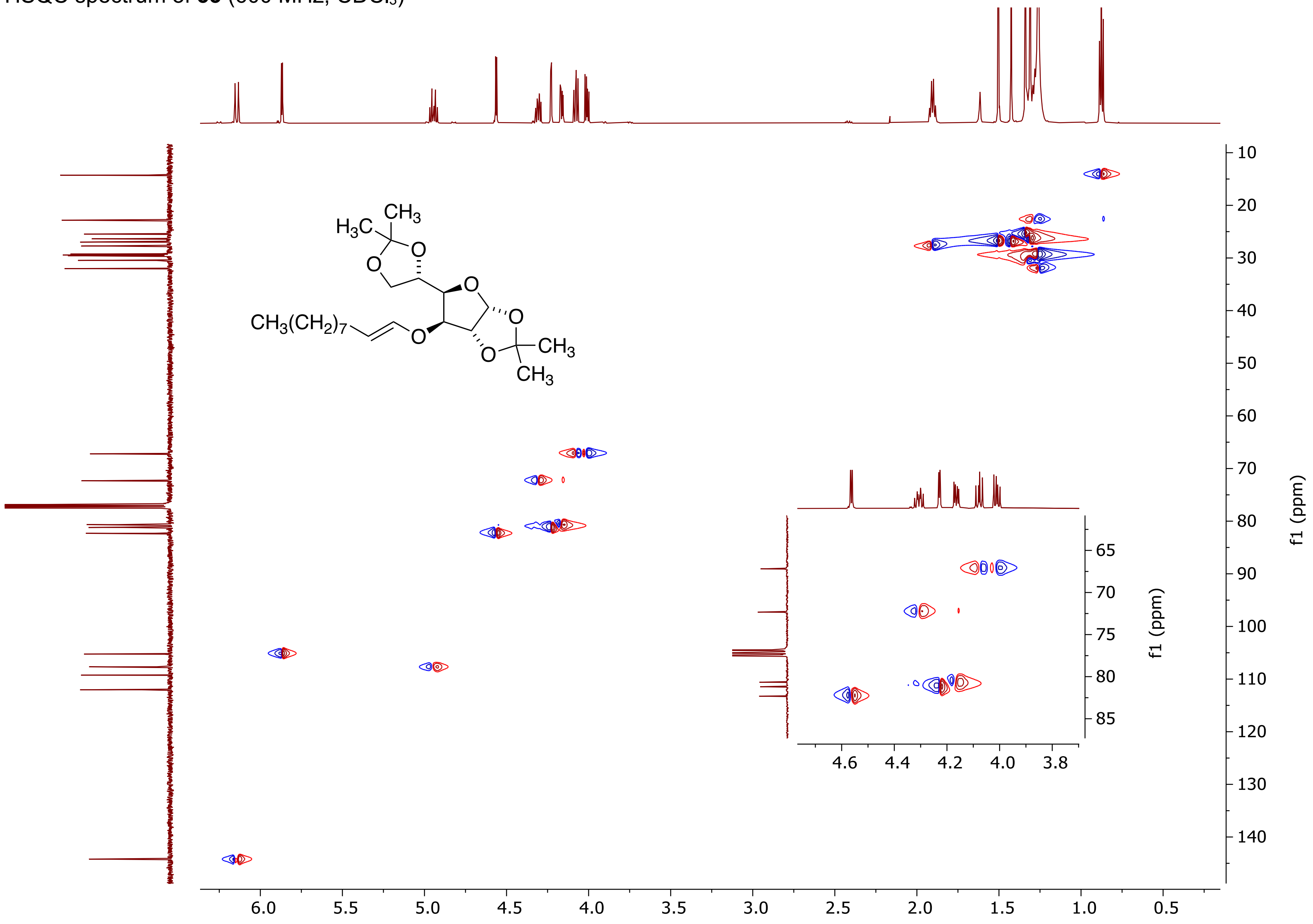

^{1}H NMR spectrum of **34** (17:1 Z/E, 600 MHz, $CDCl_3$)

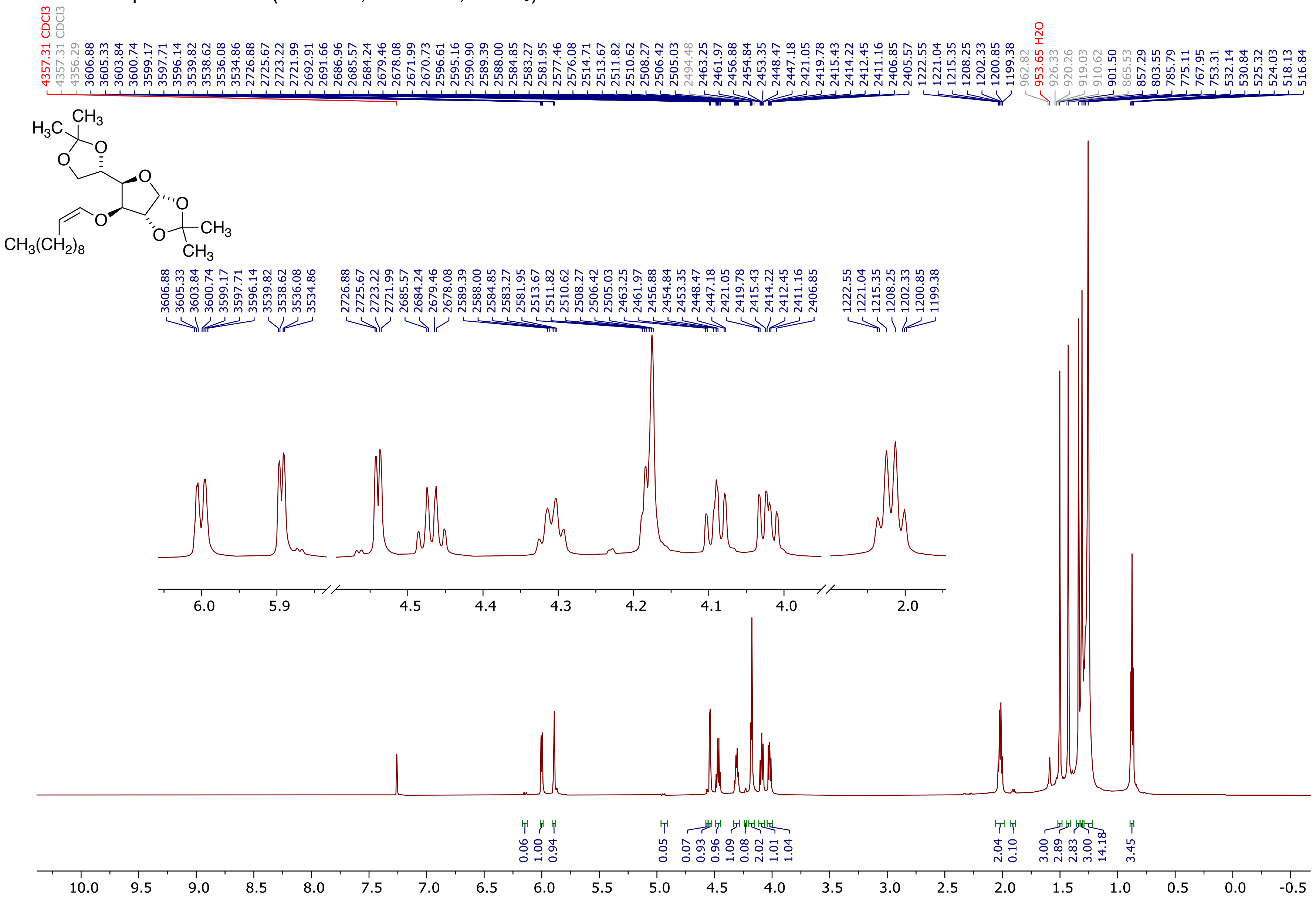

^{13}C NMR spectrum of **34** (17:1 Z/E, 151 MHz, $CDCl_3$)

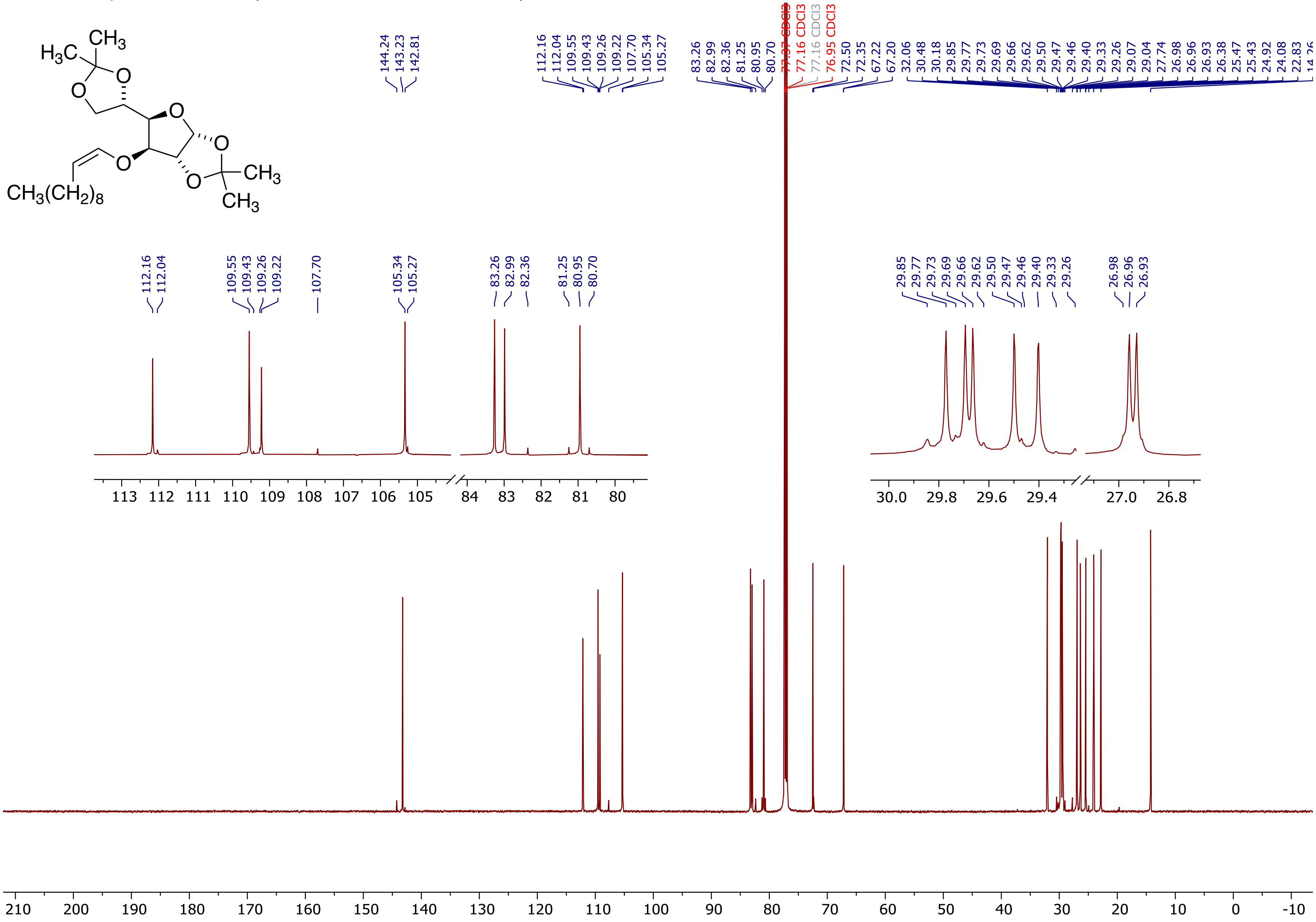

HSQC spectrum of **34** (17:1 Z/E, 600 MHz, $CDCl_3$)

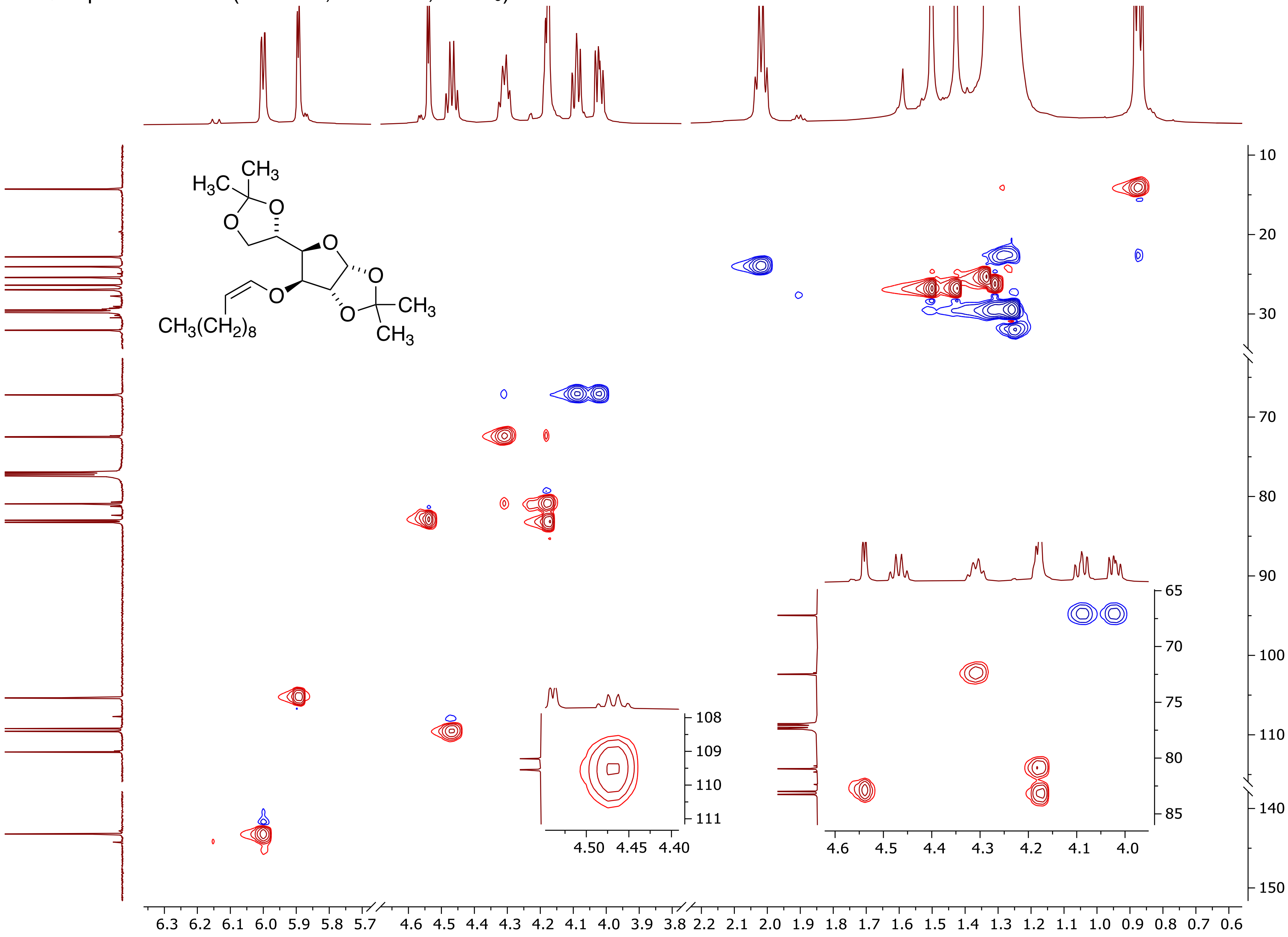

COSY spectrum of **34** (17:1 Z/E, 600 MHz, $CDCl_3$)

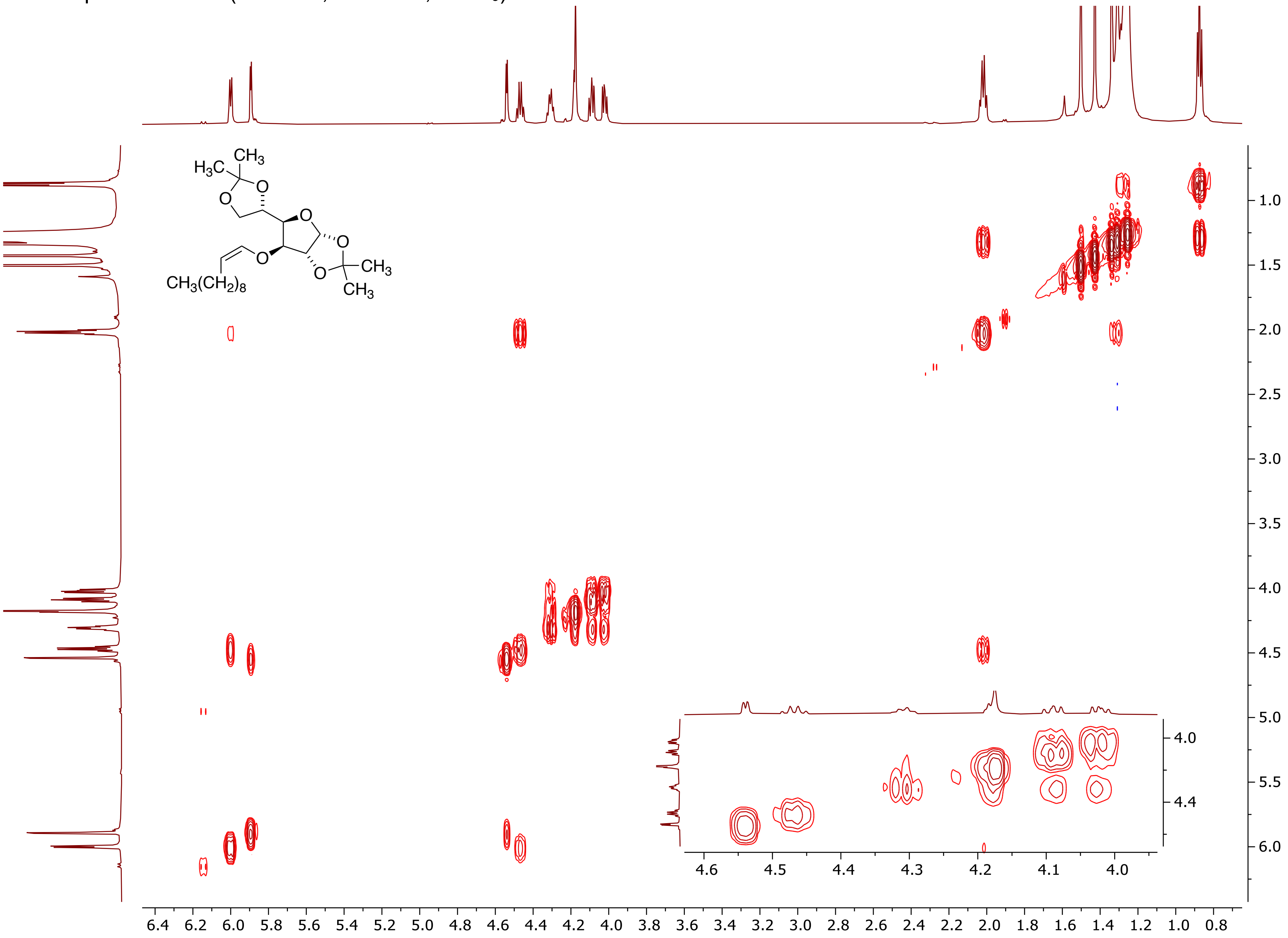

^{1}H NMR spectrum of **35** (400 MHz, $CDCl_3$)

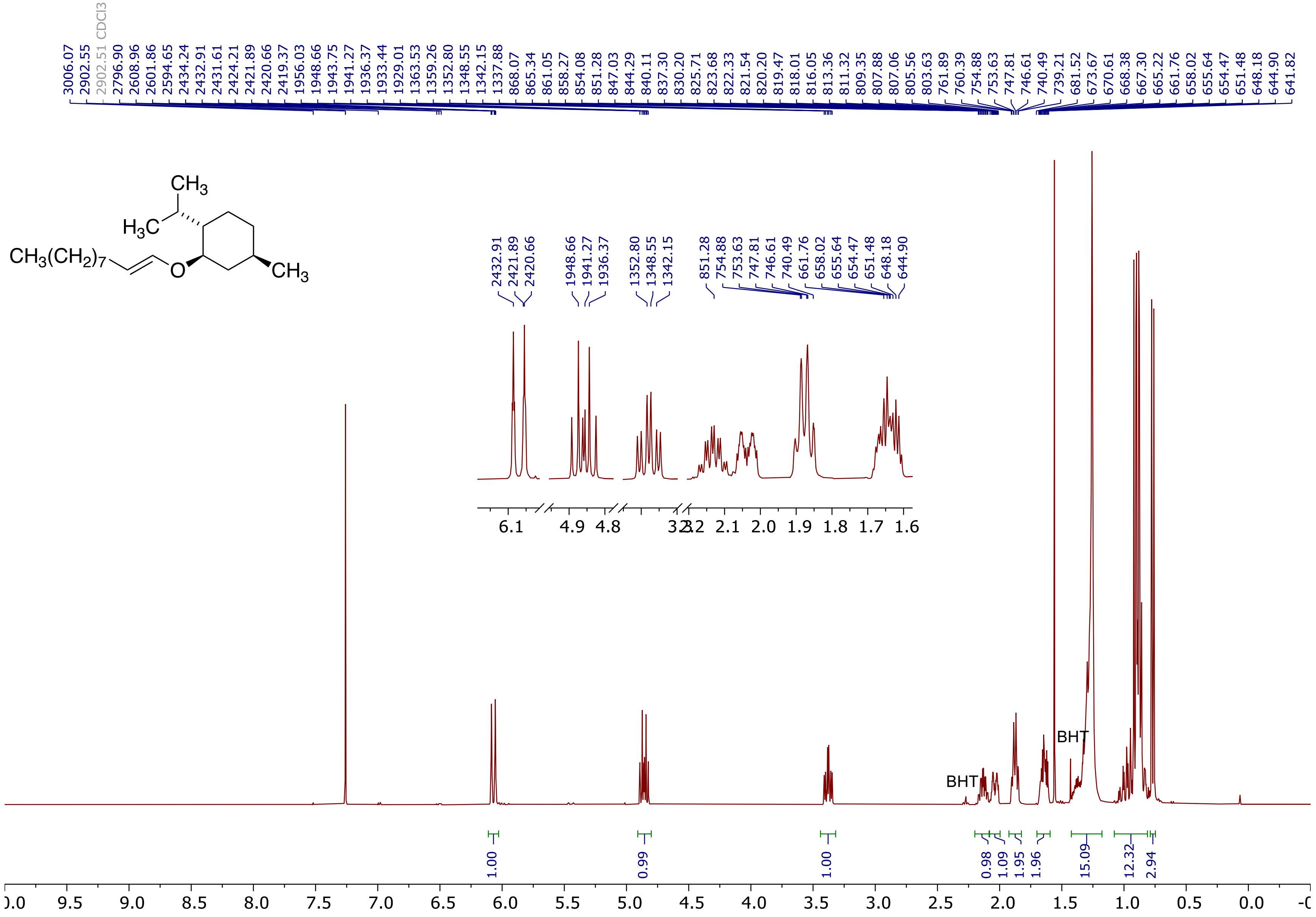

^{13}C NMR spectrum of **35** (101 MHz, $CDCl_3$)

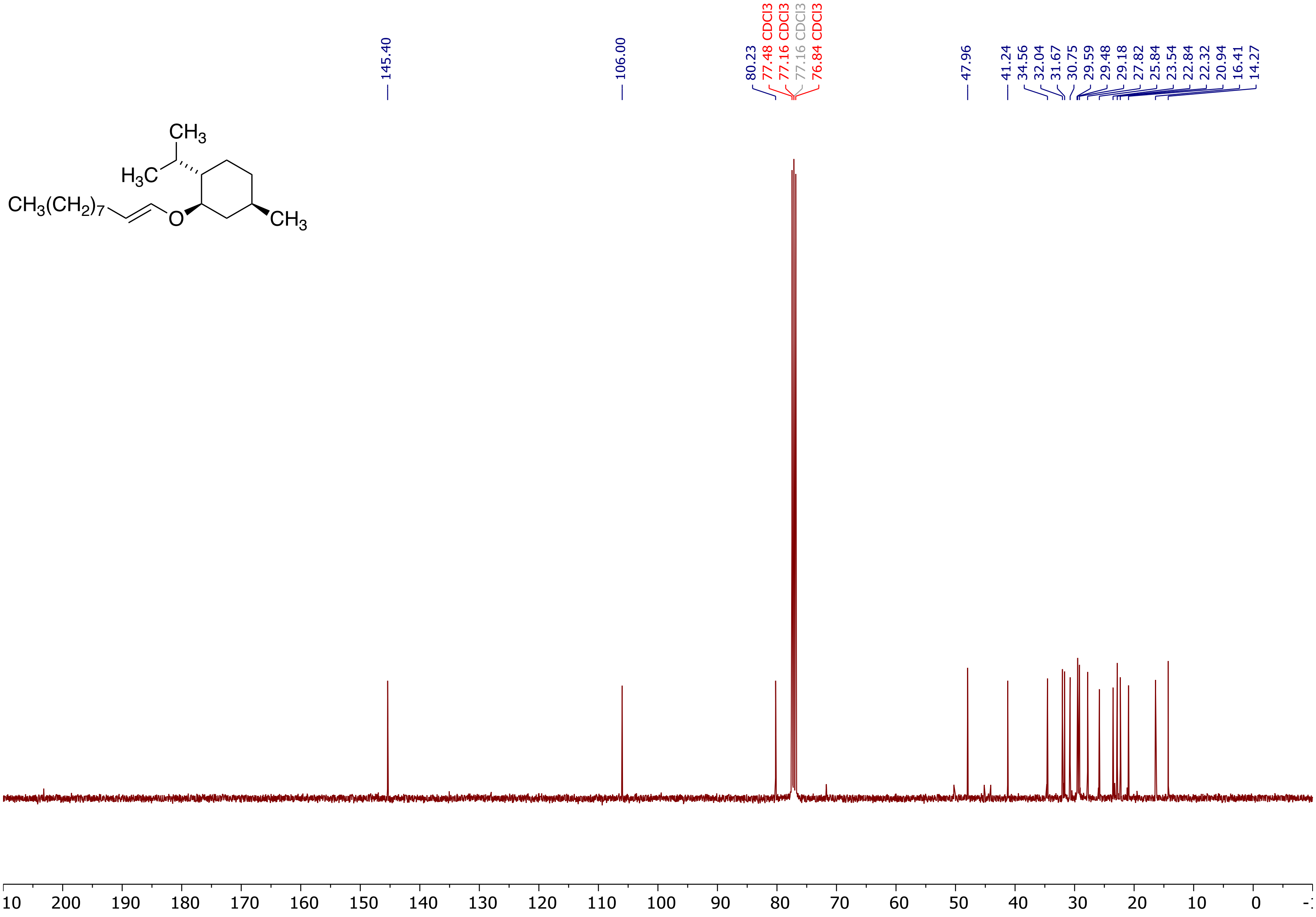

^{1}H NMR spectrum of **36** (>20:1 Z/E, 5.6:1 vinyl ether to enyne, 400 MHz, $CDCl_3$)

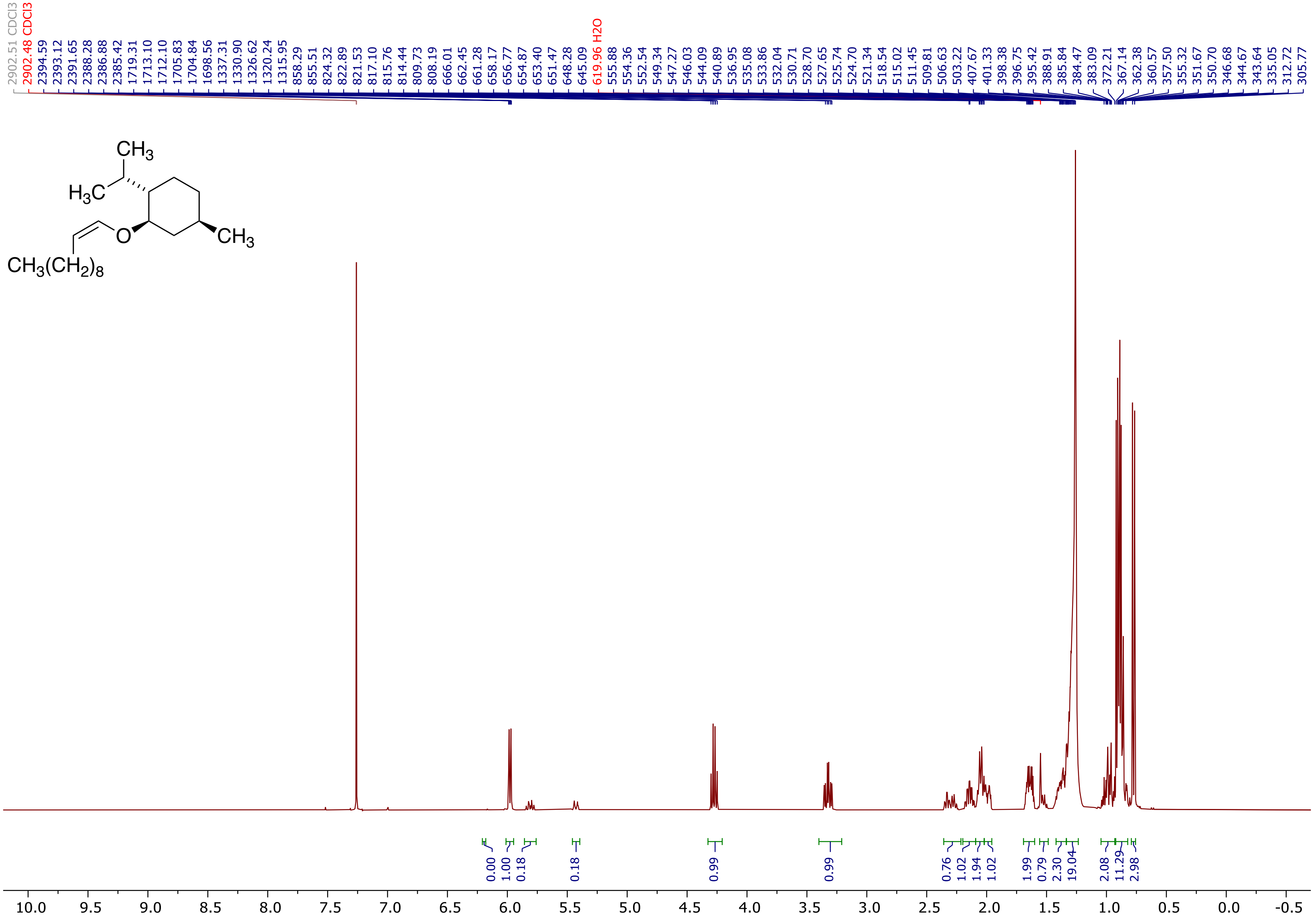

^{13}C NMR spectrum of **36** (>20:1 Z/E, 5.6:1 vinyl ether to enyne, 101 MHz, $CDCl_3$)

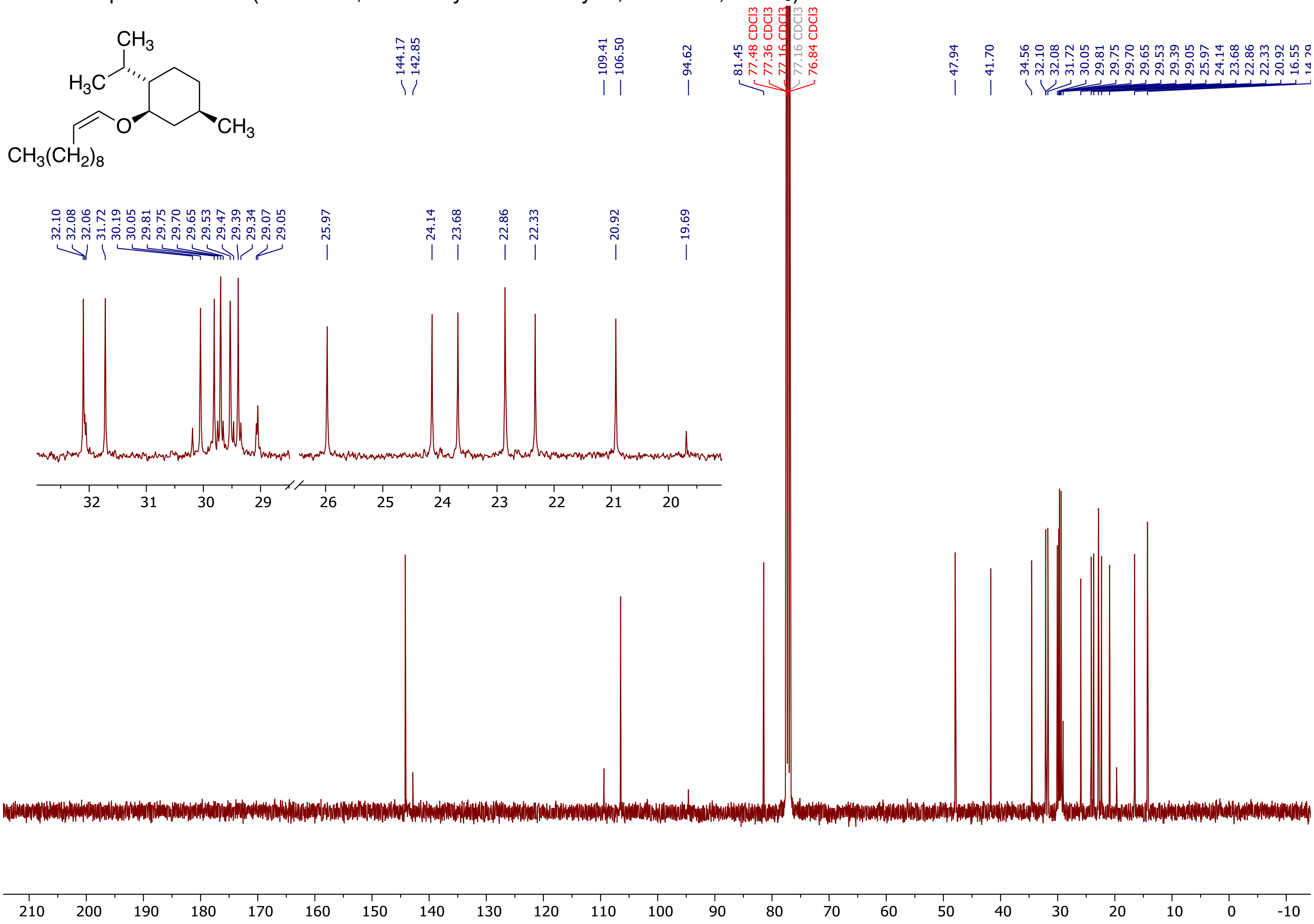

^{1}H NMR spectrum of **37** (400 MHz, $CDCl_3$)

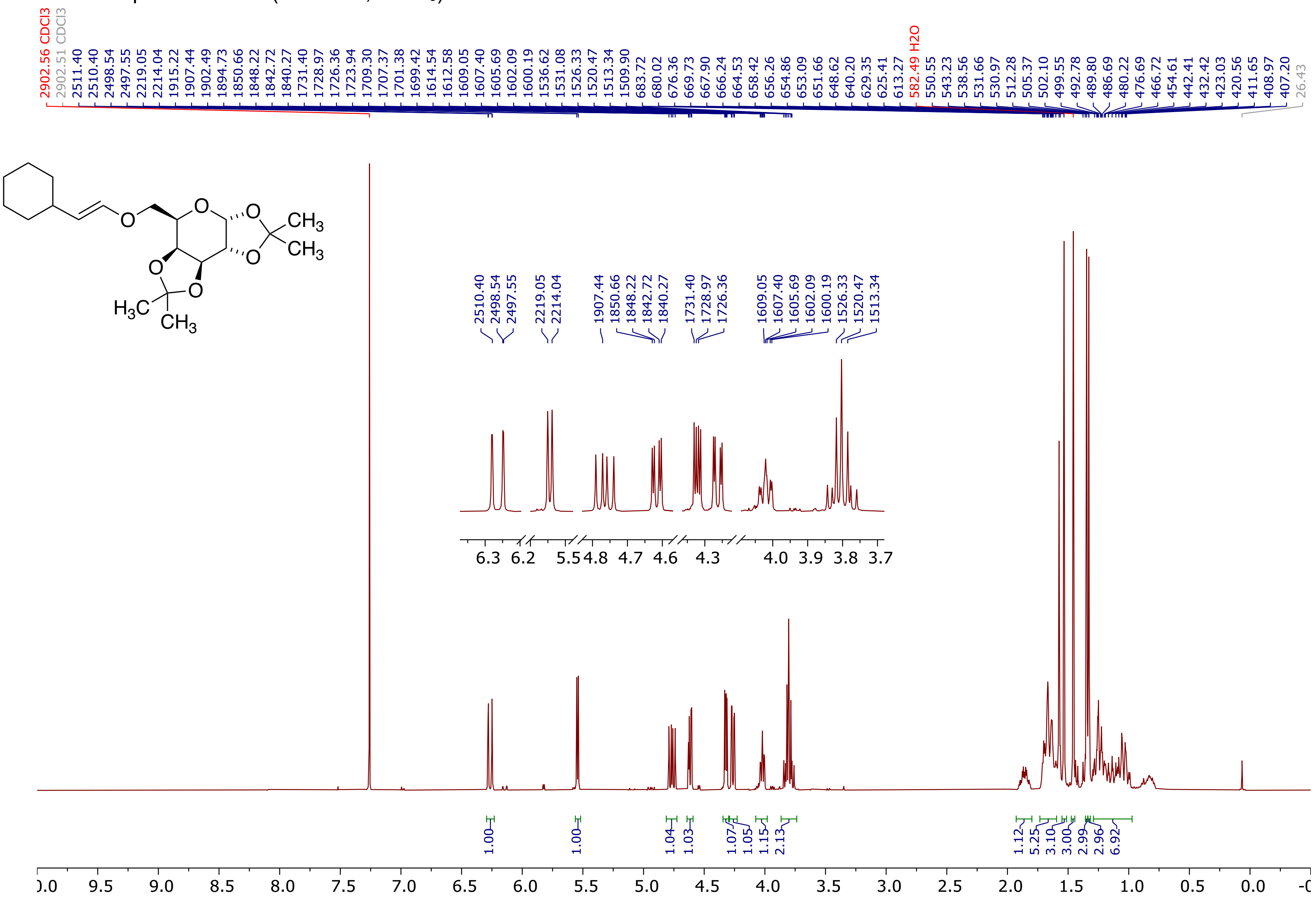

^{13}C NMR spectrum of **37** (101 MHz, $CDCl_3$)

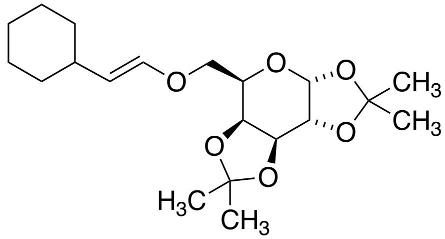

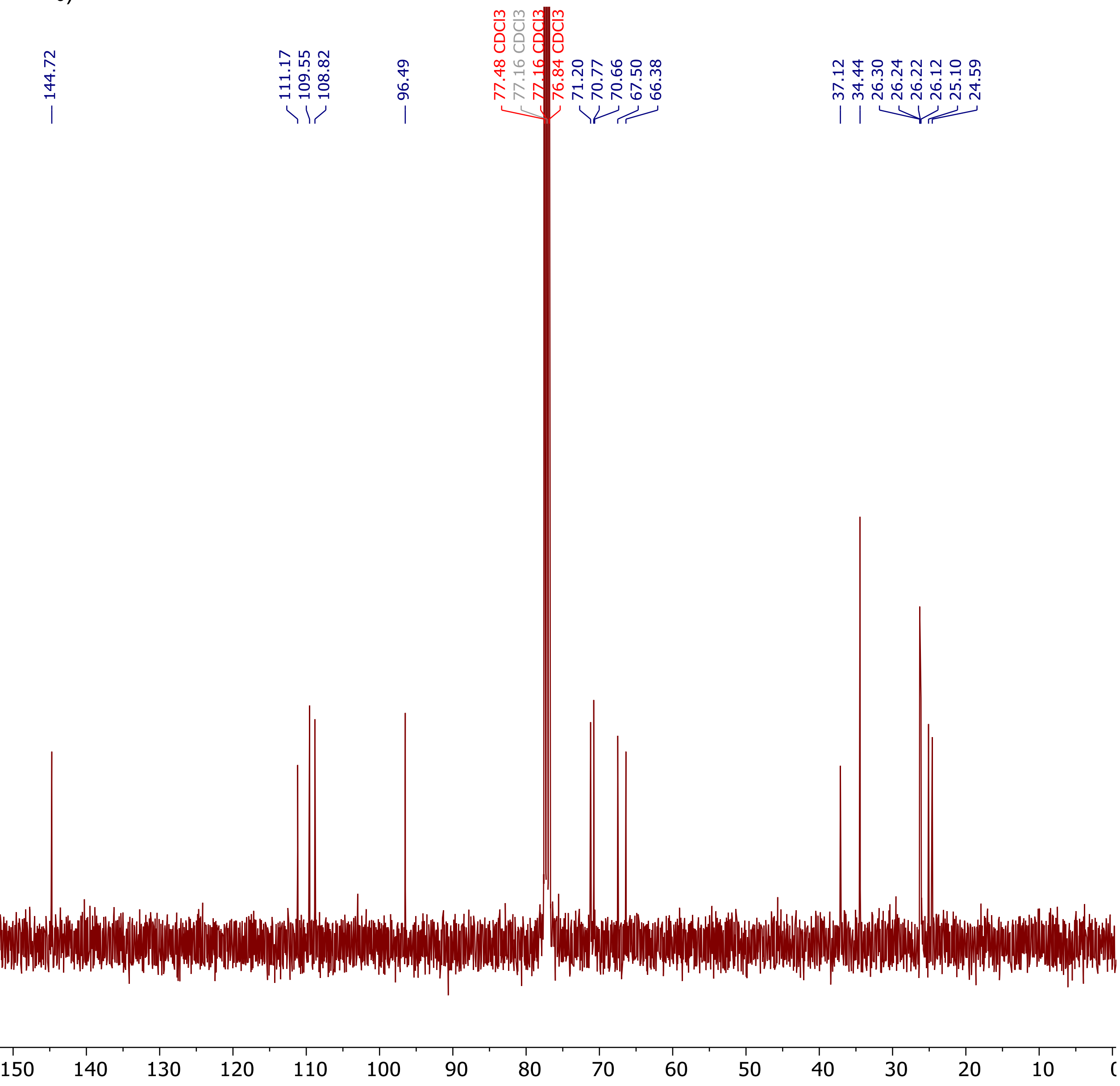

COSY spectrum of **37** (600 MHz, $CDCl_3$)

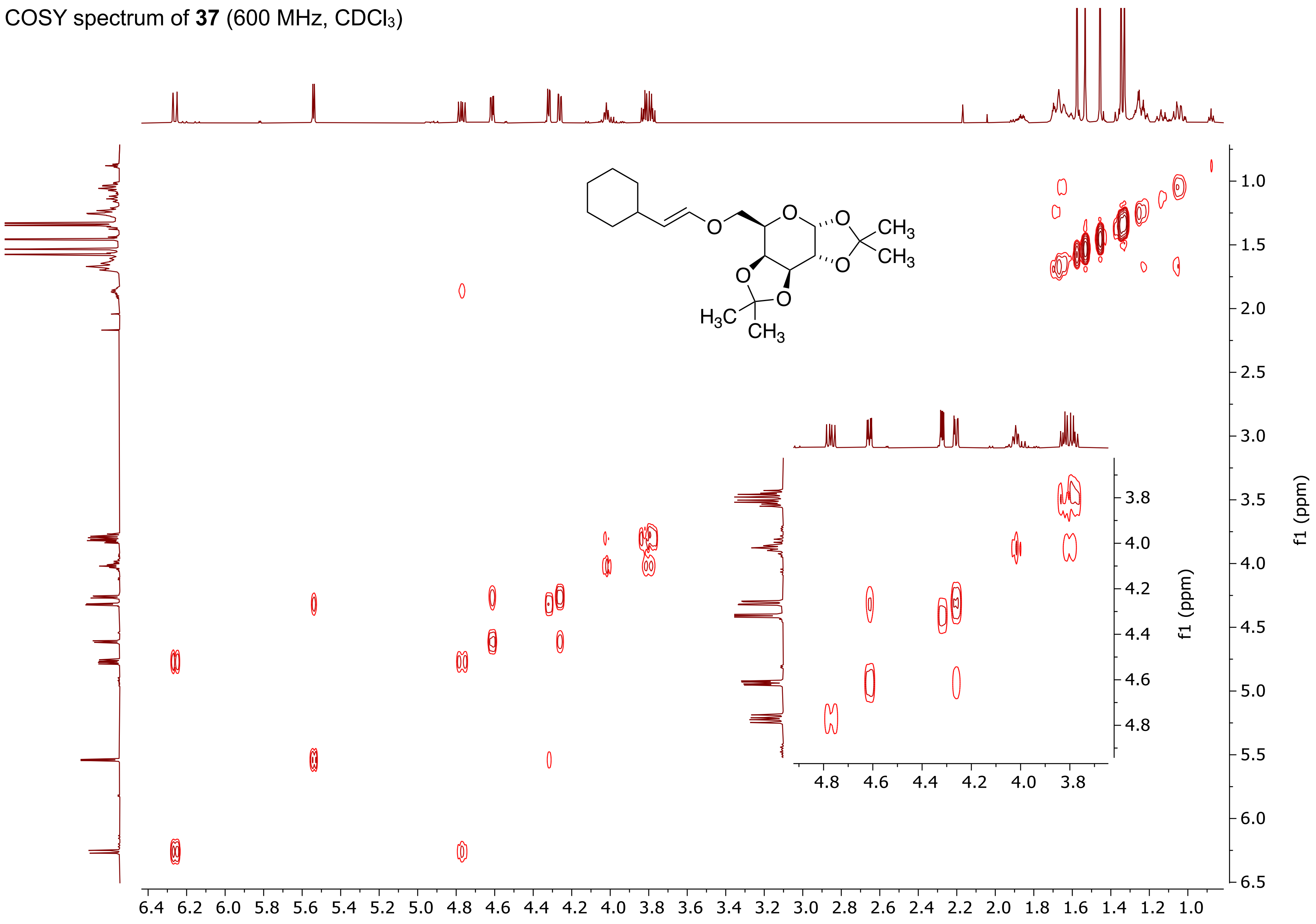

HSQC spectrum of **37** (600 MHz, $CDCl_3$)

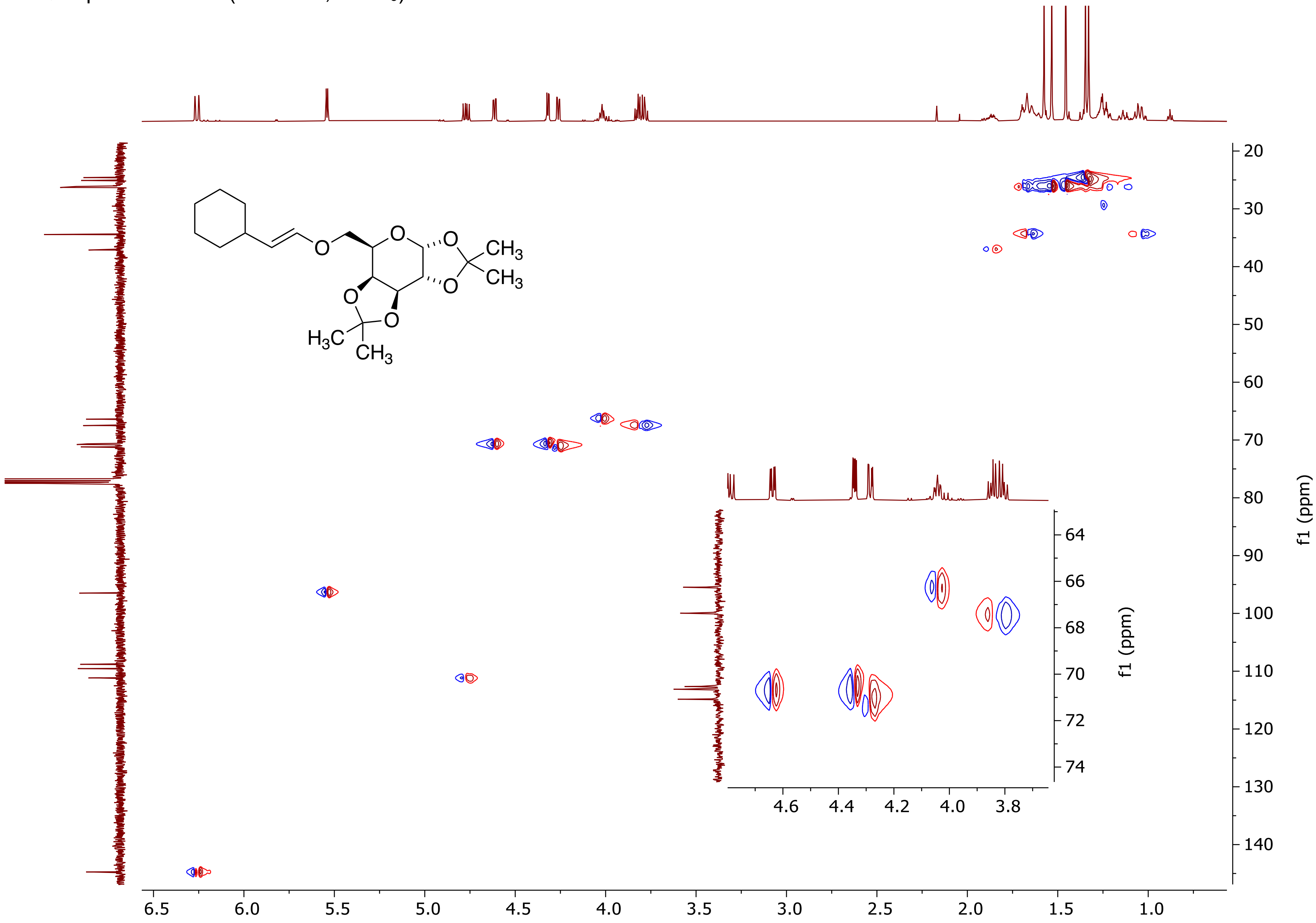

^{1}H NMR spectrum of **38** (14:1 Z/E, 600 MHz, $CDCl_3$)

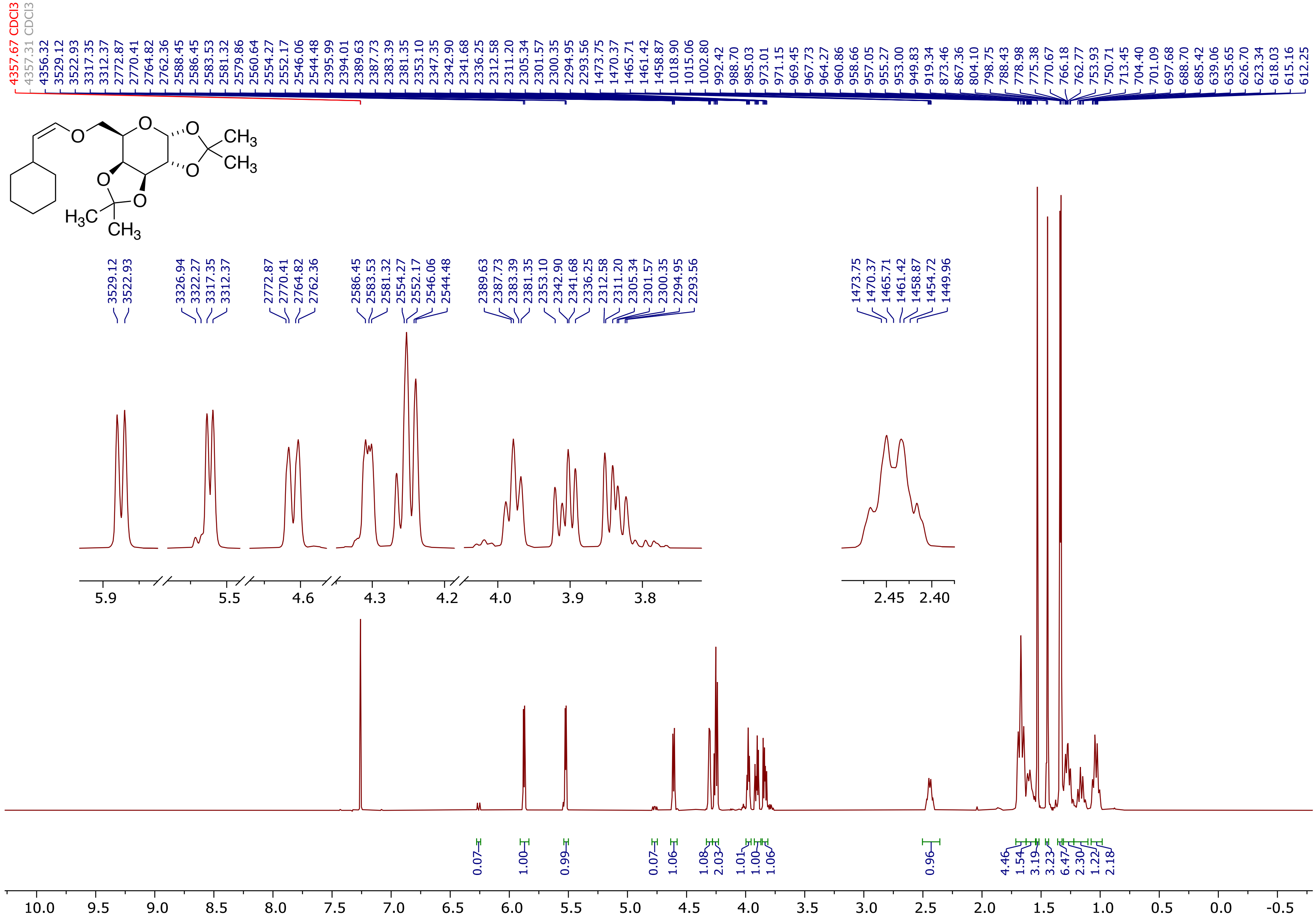

^{13}C NMR spectrum of **38** (14:1 Z/E, 151 MHz, $CDCl_3$)

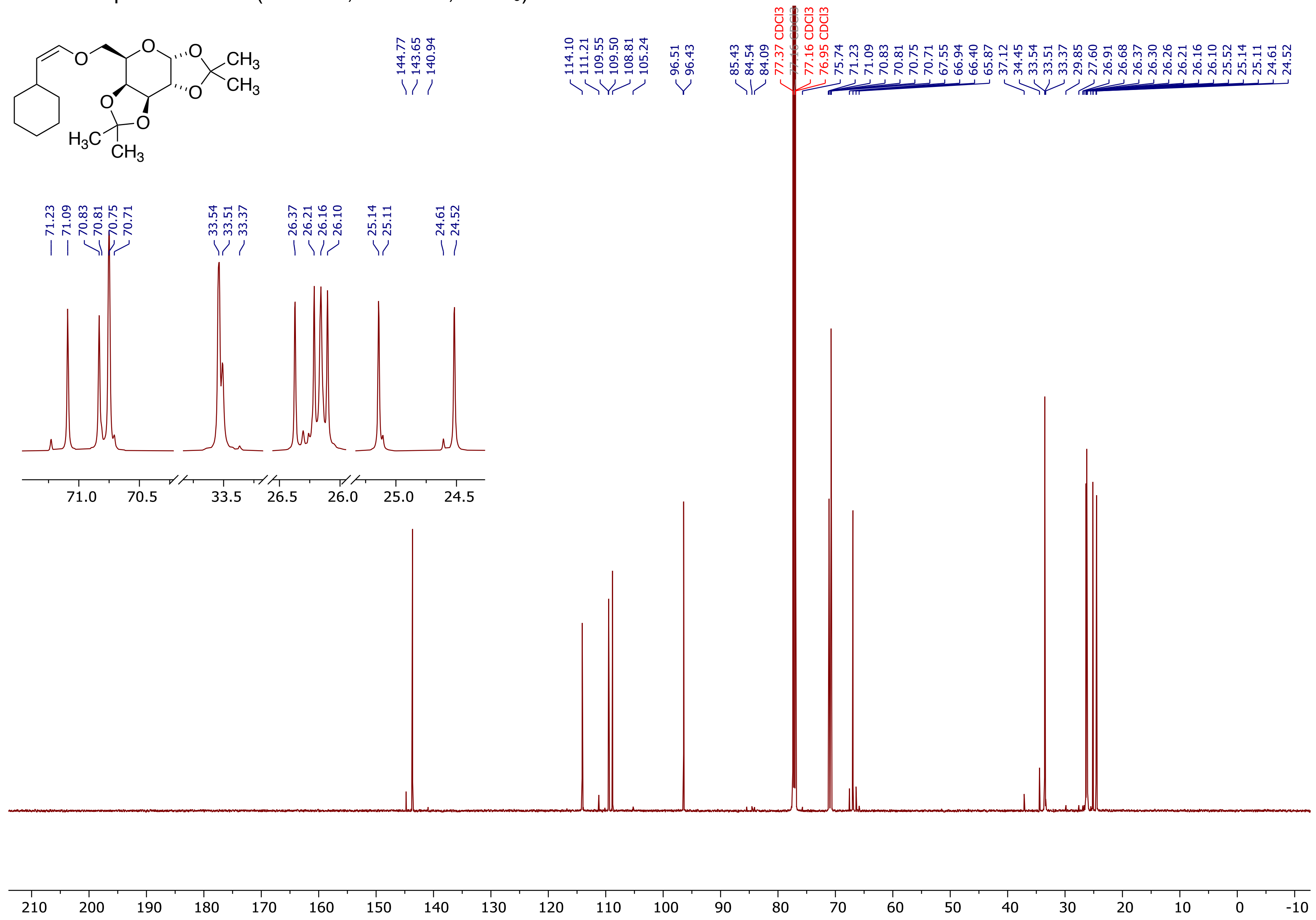

COSY spectrum of **38** (14:1 Z/E, 800 MHz, $CDCl_3$)

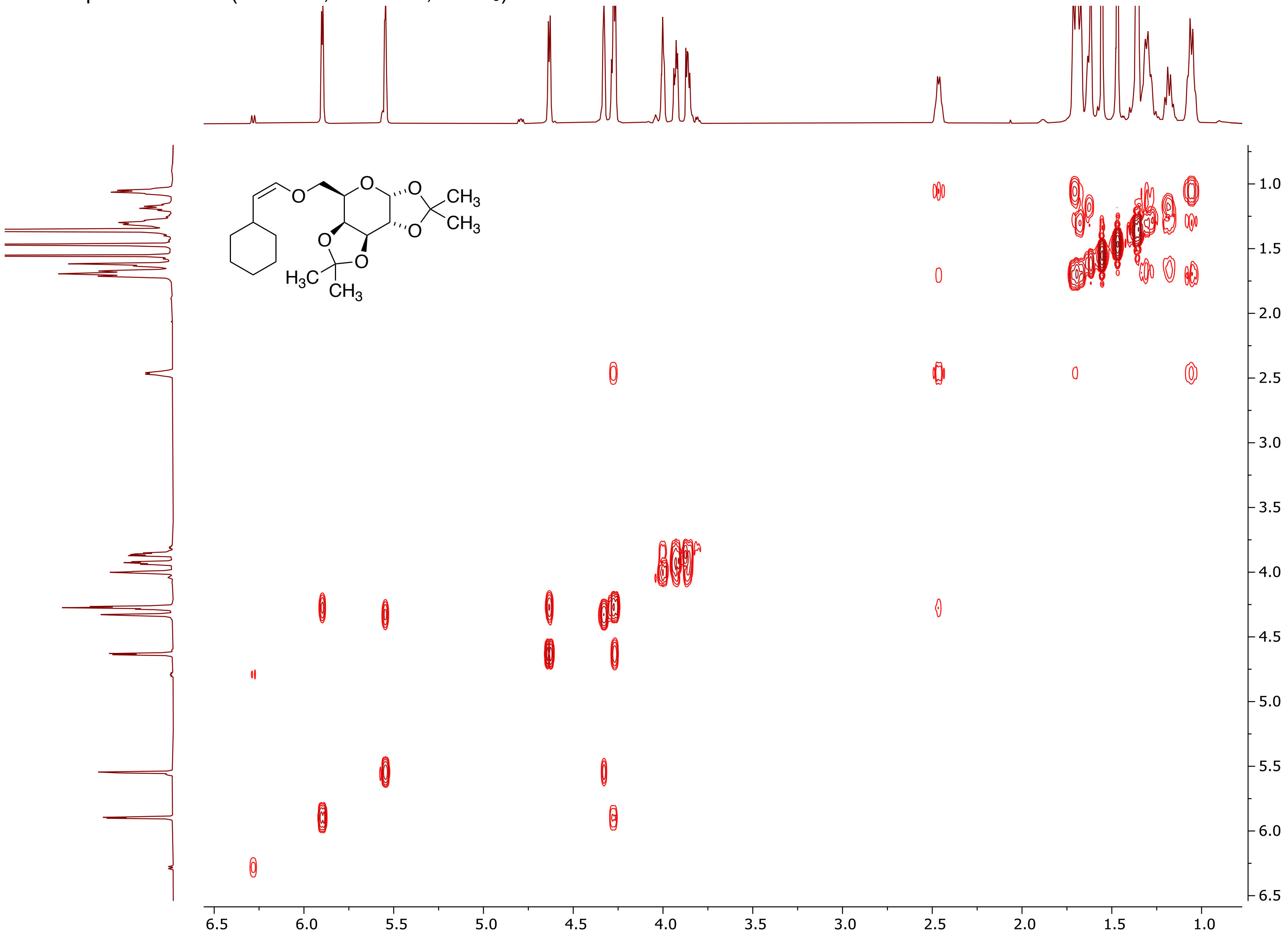

HSQC spectrum of **38** (14:1 Z/E, 600 MHz, $CDCl_3$)

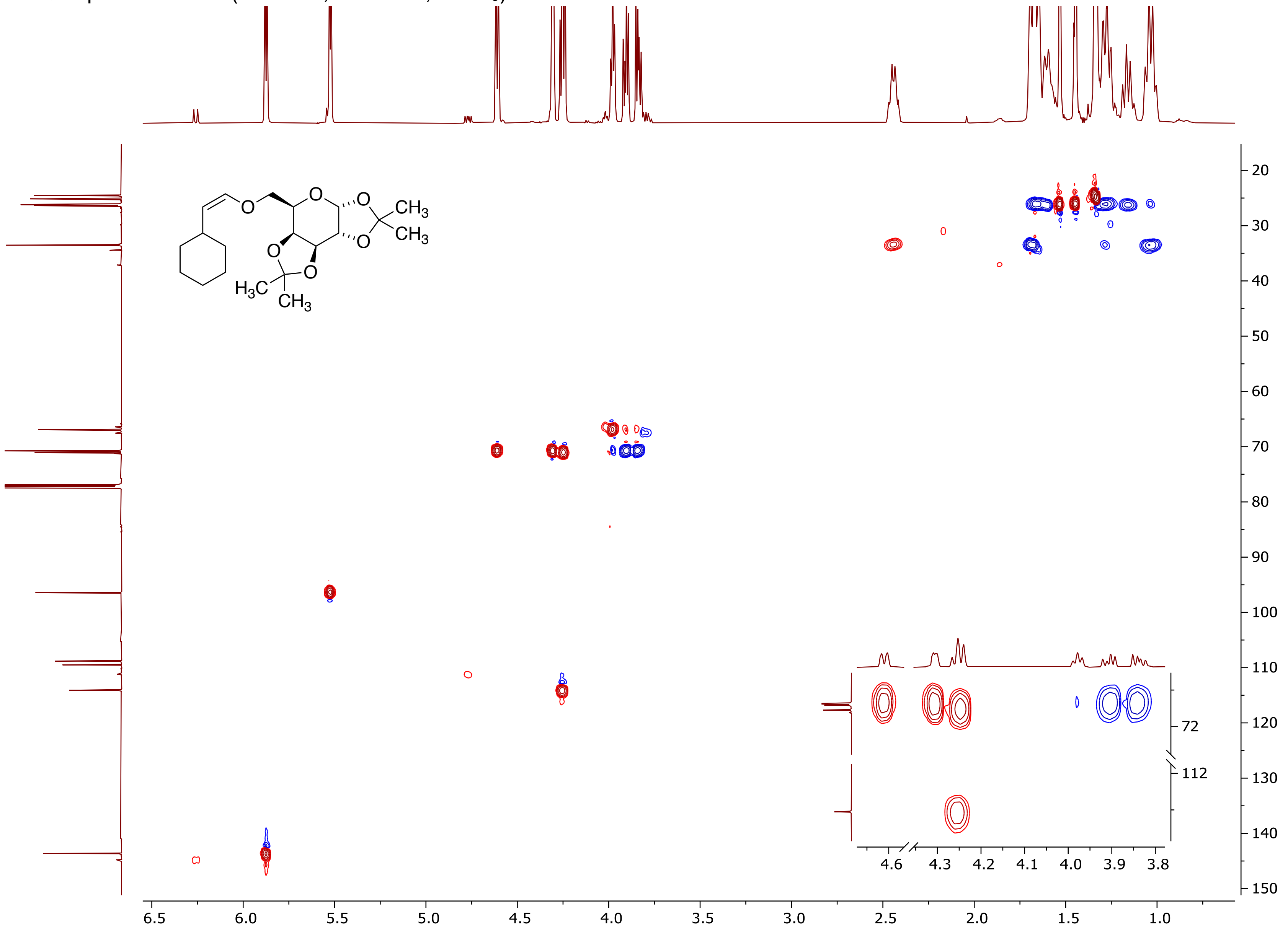

HSQC spectrum of **38** (14:1 Z/E, 600 MHz, $CDCl_3$) expansion

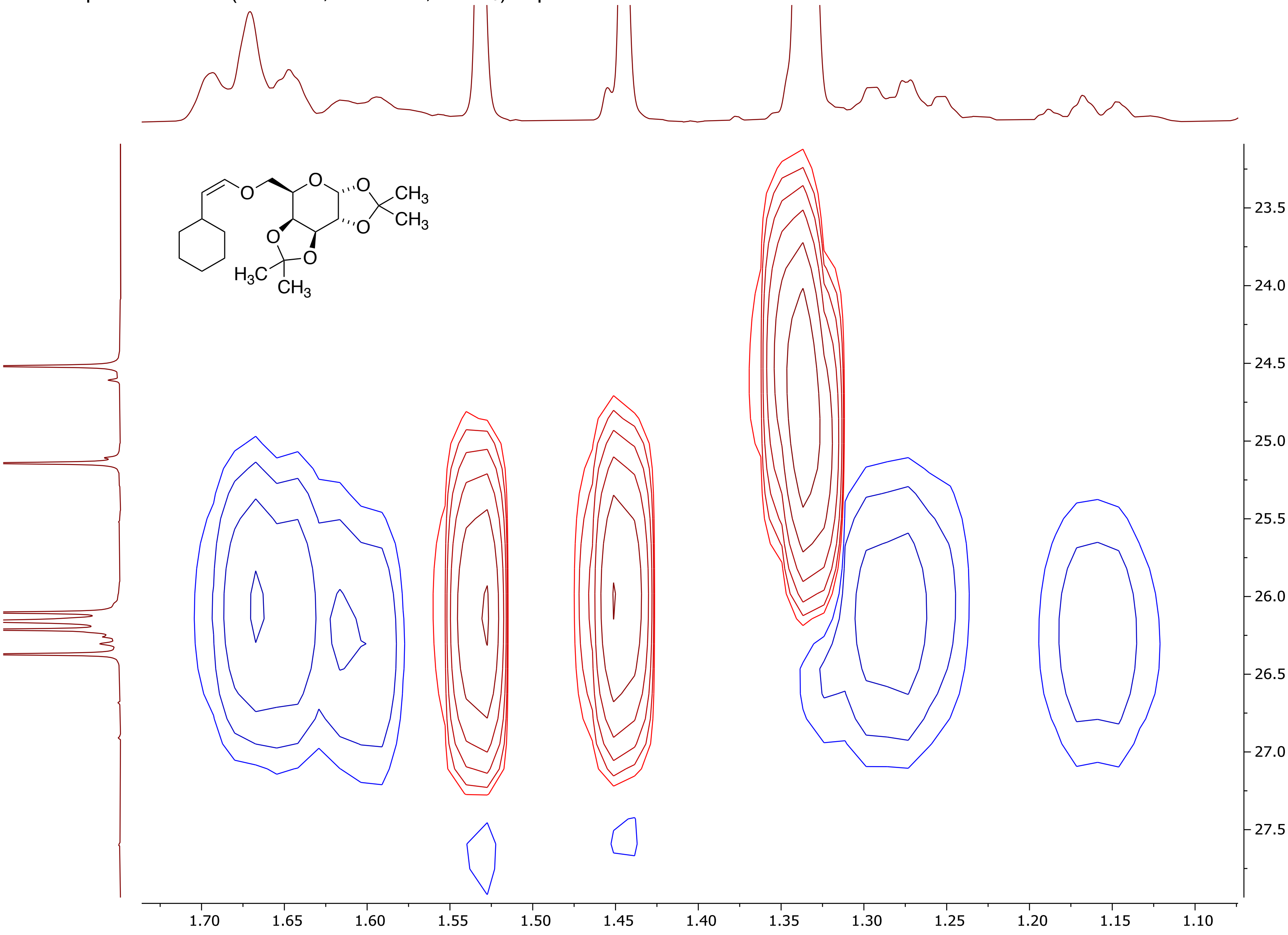

^{1}H NMR spectrum of enyne **S17** (400 MHz, $CDCl_3$)

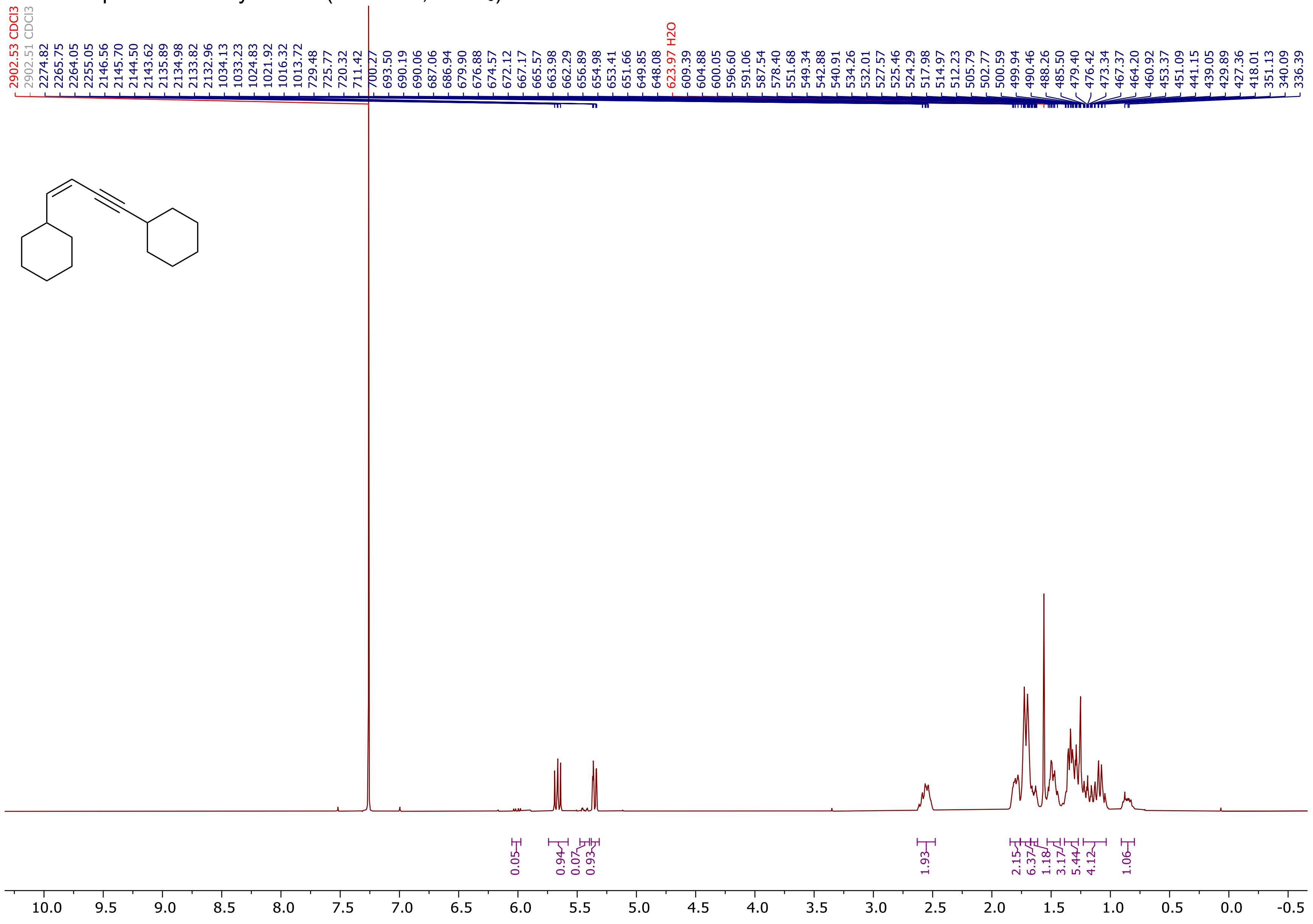

^{13}C NMR spectrum of enyne **S17** (101 MHz, $CDCl_3$)

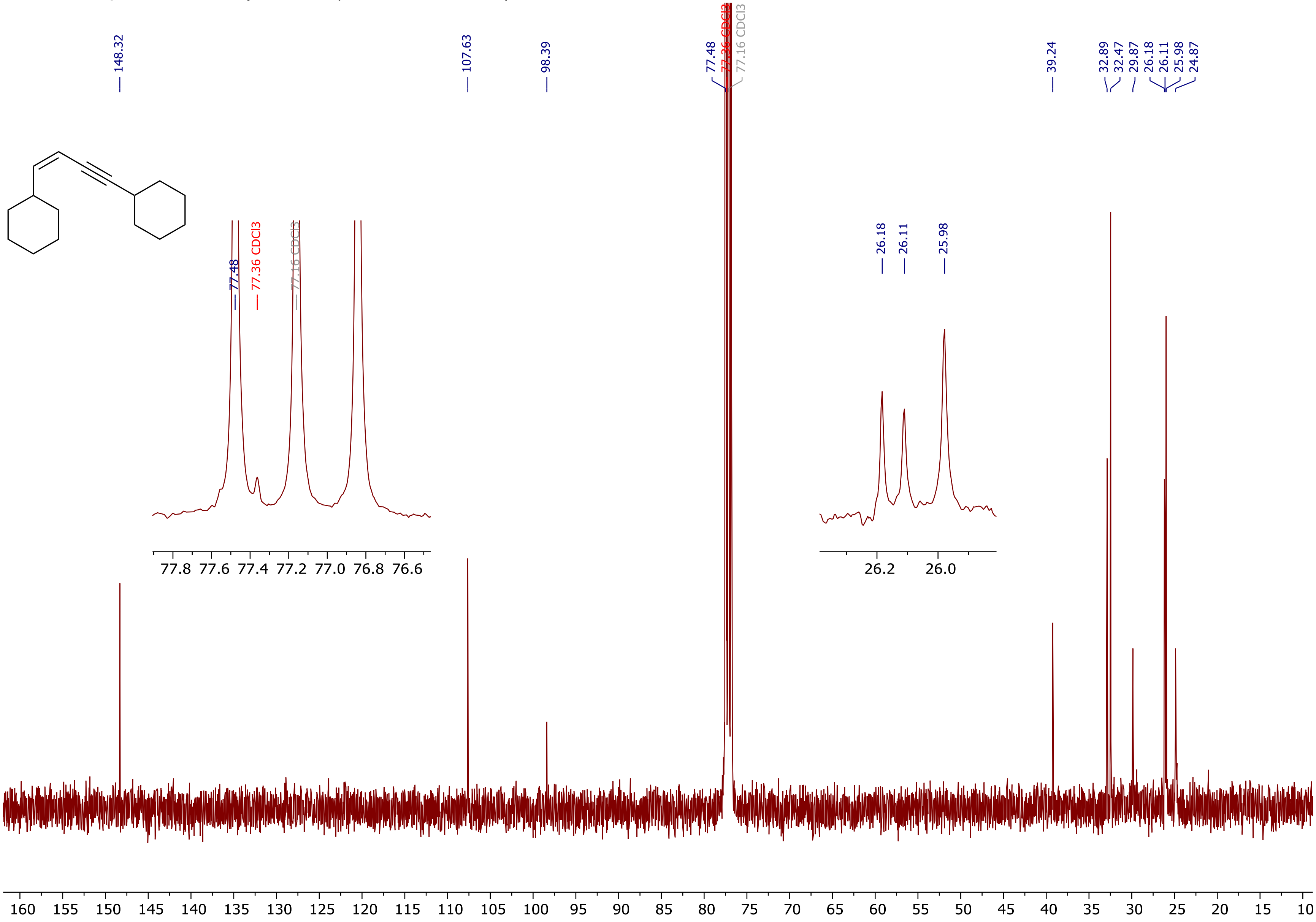

^{1}H NMR spectrum of **39** (400 MHz, $CDCl_3$)

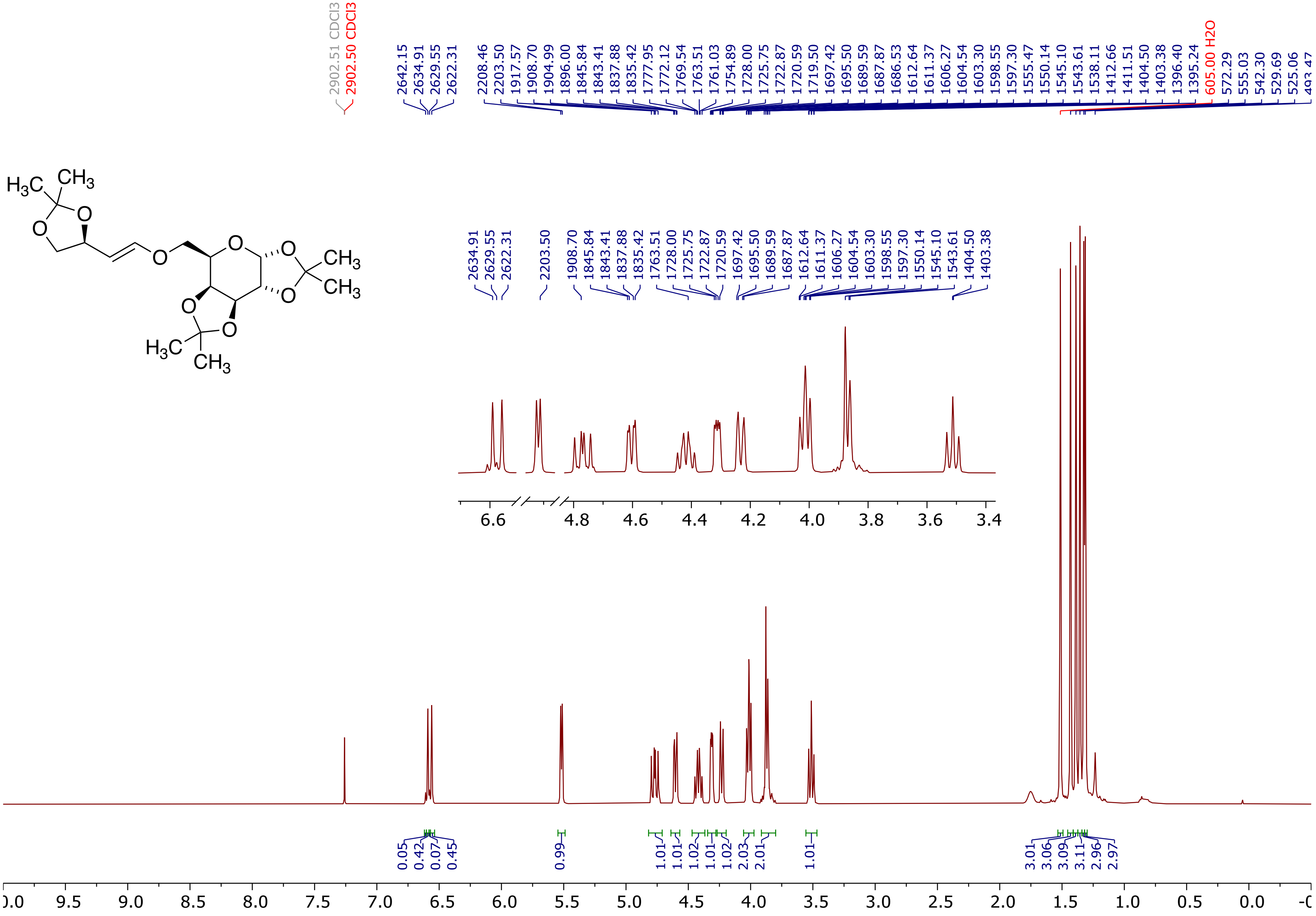

^{13}C NMR spectrum of **39** (101 MHz, $CDCl_3$)

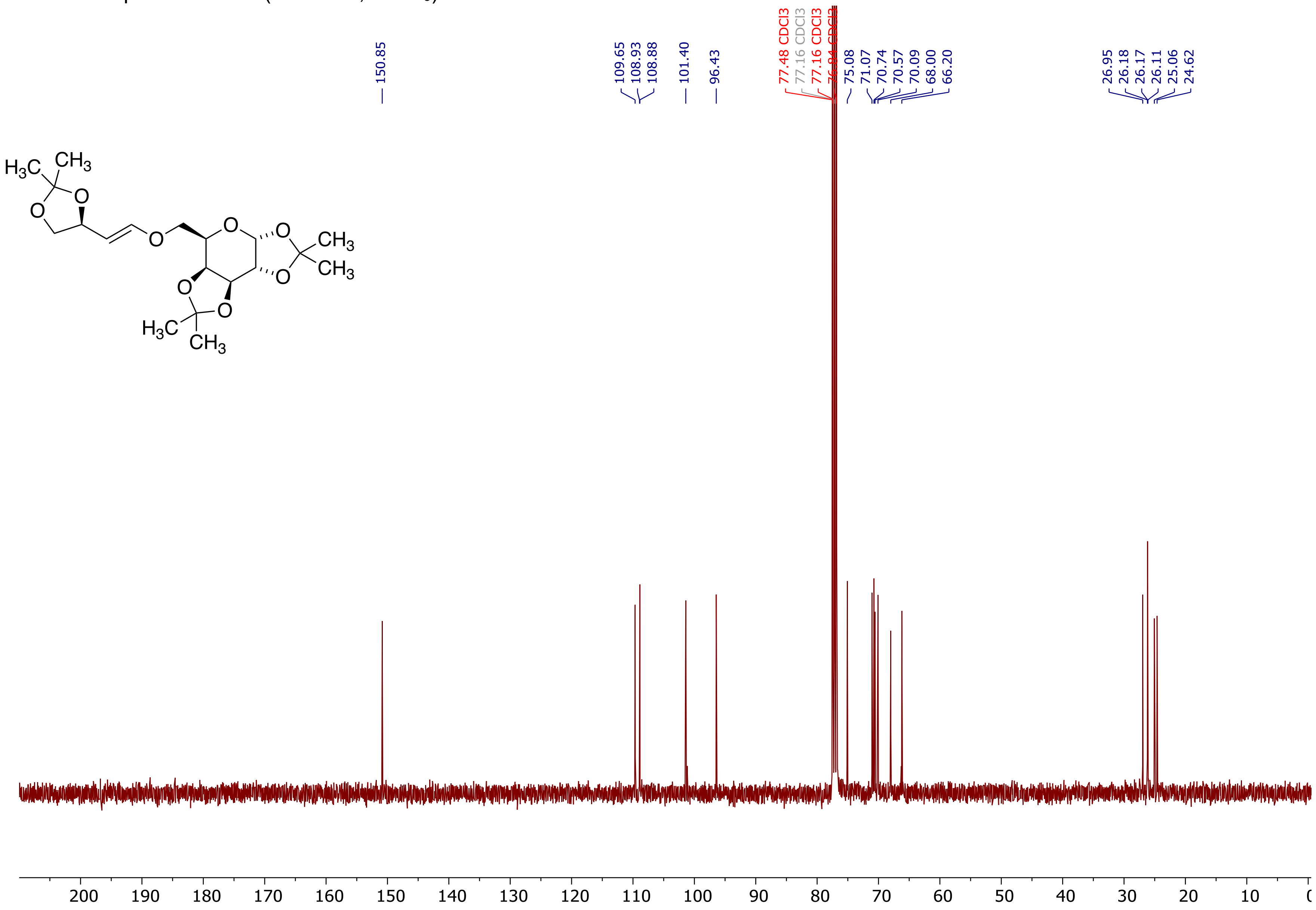

COSY spectrum of **39** (600 MHz, $CDCl_3$)

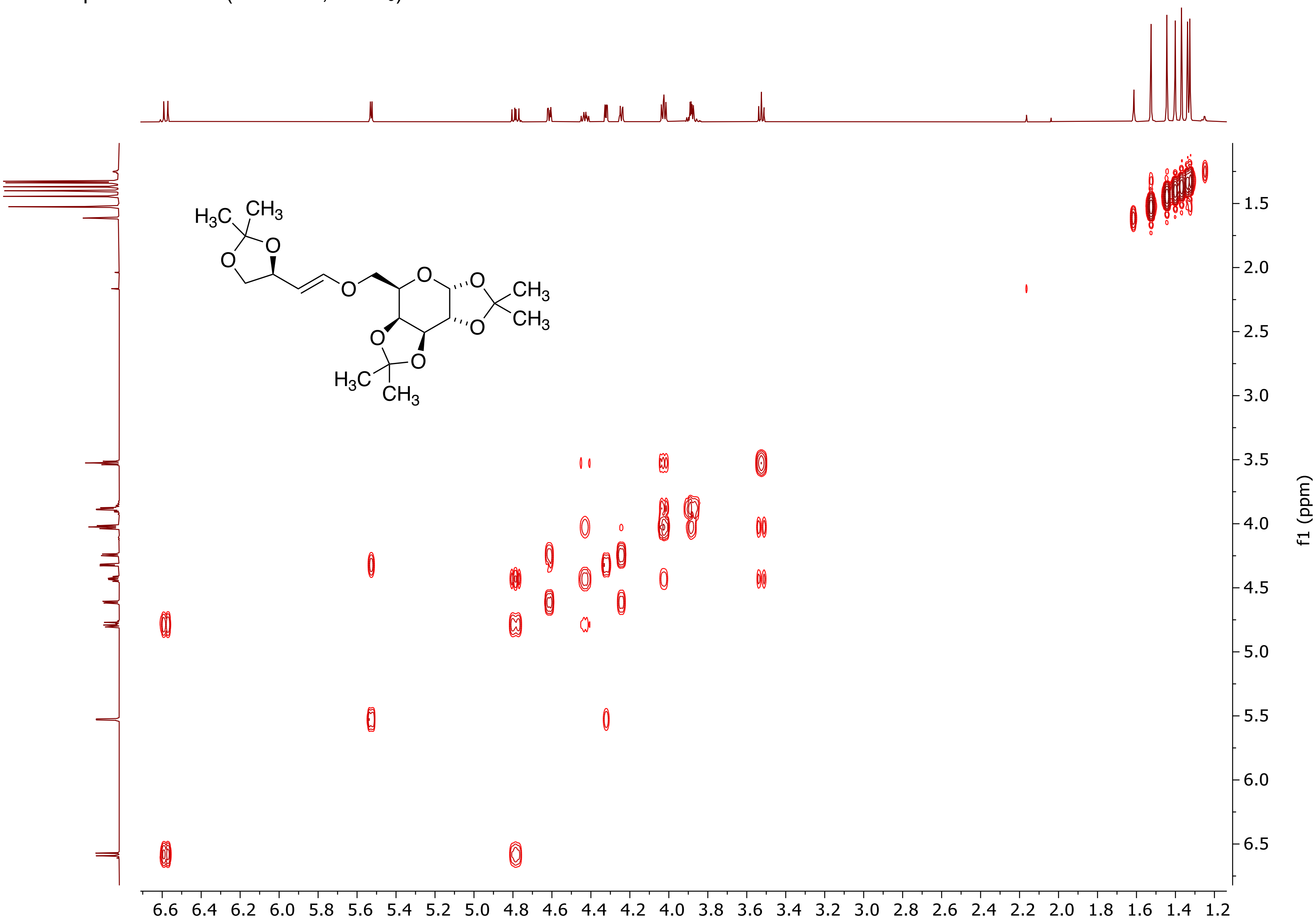

COSY spectrum of **39** (600 MHz, $CDCl_3$) expansion

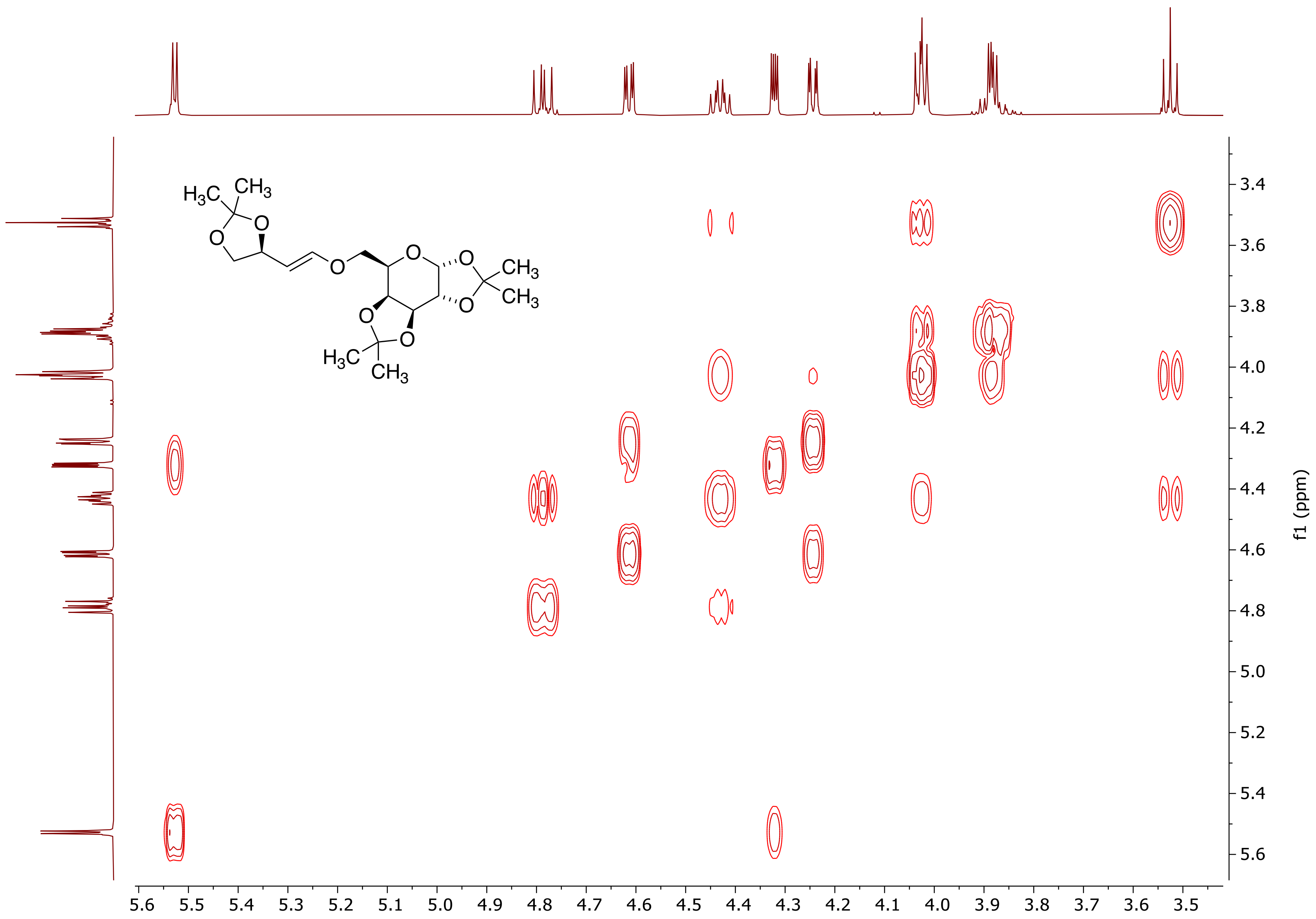

HSQC spectrum of **39** (600 MHz, $CDCl_3$)

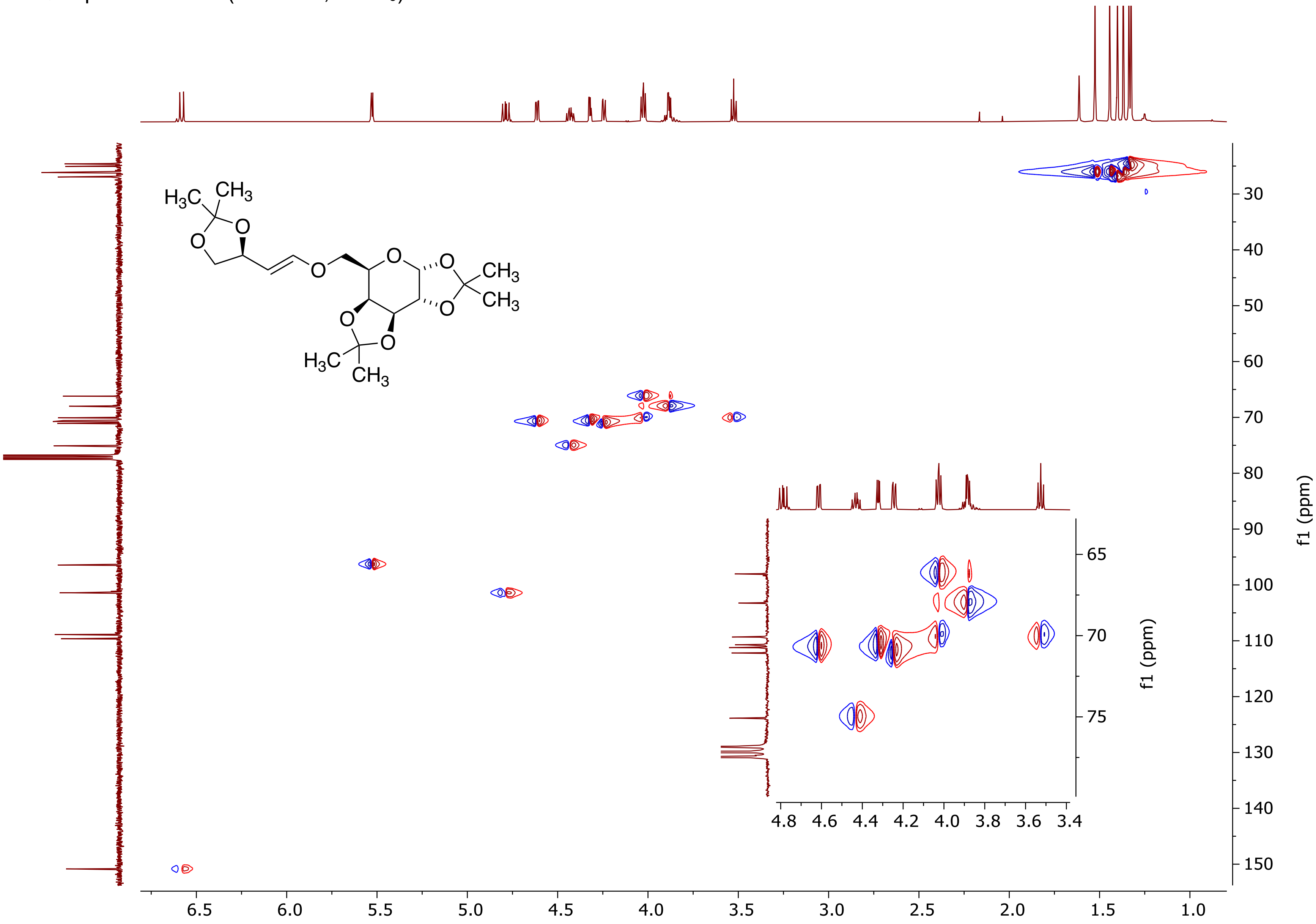

^{1}H NMR spectrum of **40** (4:1 Z/E, 10:1 d.r., >95% pure, 600 MHz, $CDCl_3$)

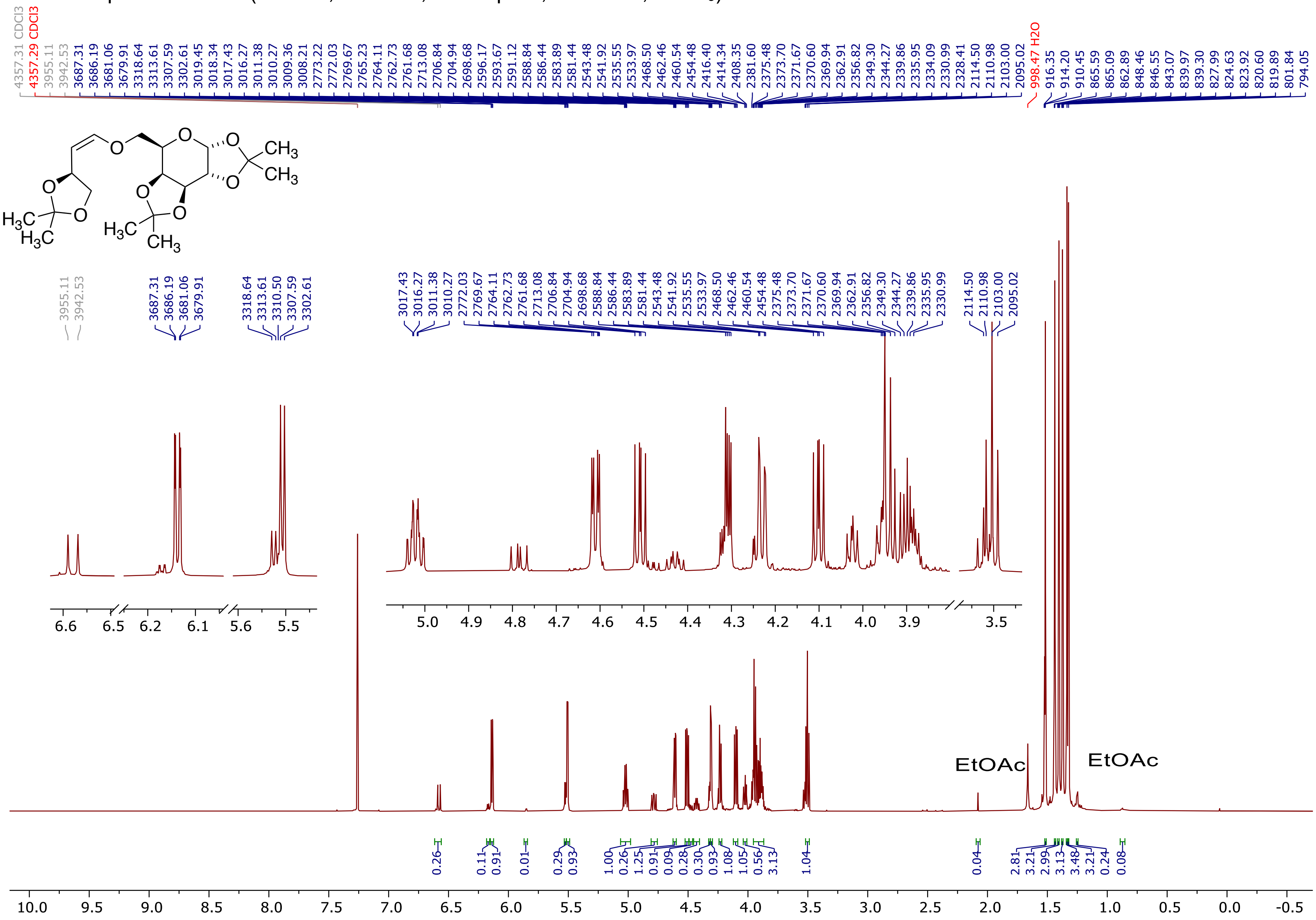

^{13}C NMR spectrum of **40** (4:1 Z/E, 10:1 d.r., >95% pure, 151 MHz, $CDCl_3$)

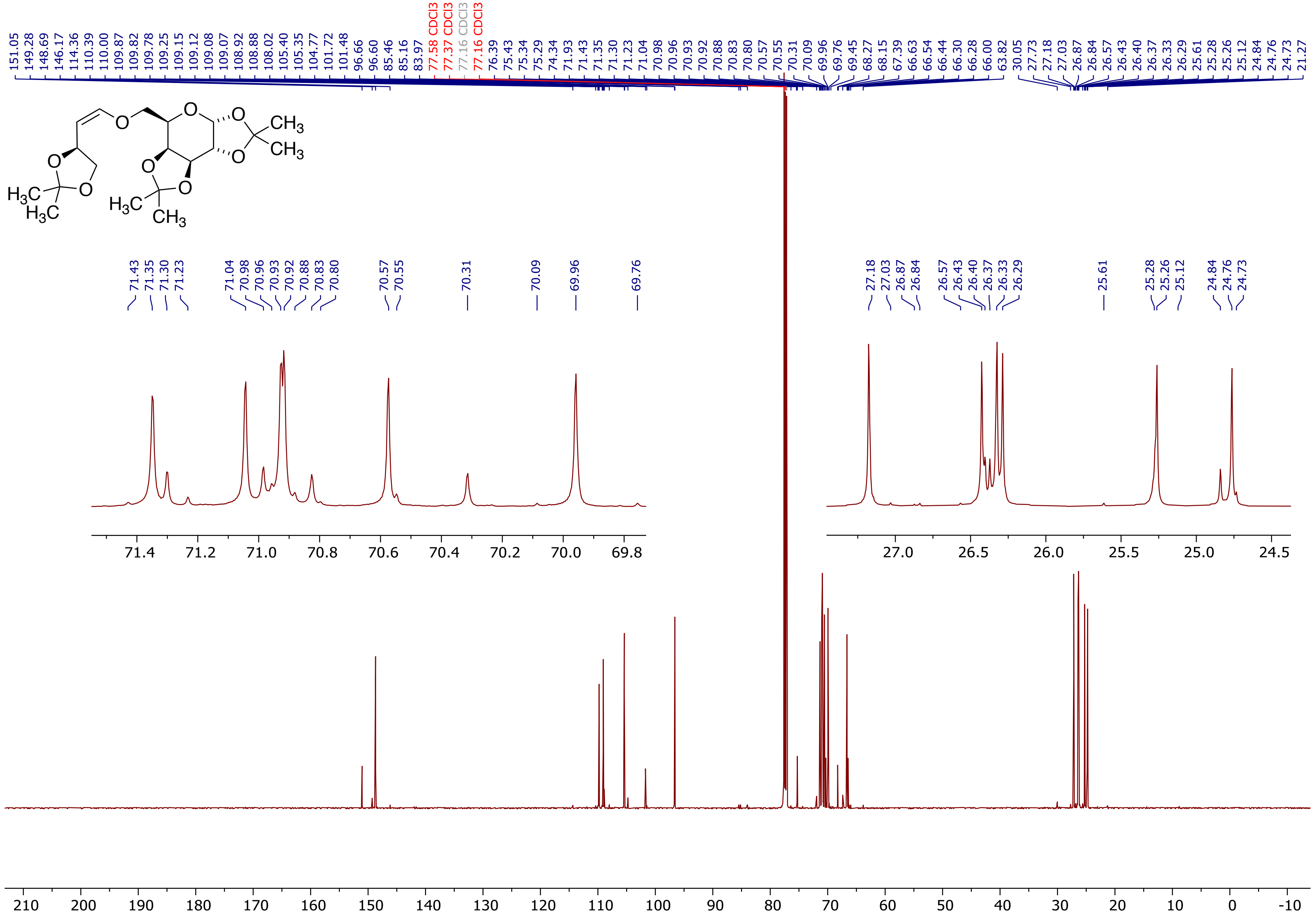

COSY spectrum of **40** (4:1 Z/E, 10:1 d.r., >95% pure, 600 MHz, $CDCl_3$)

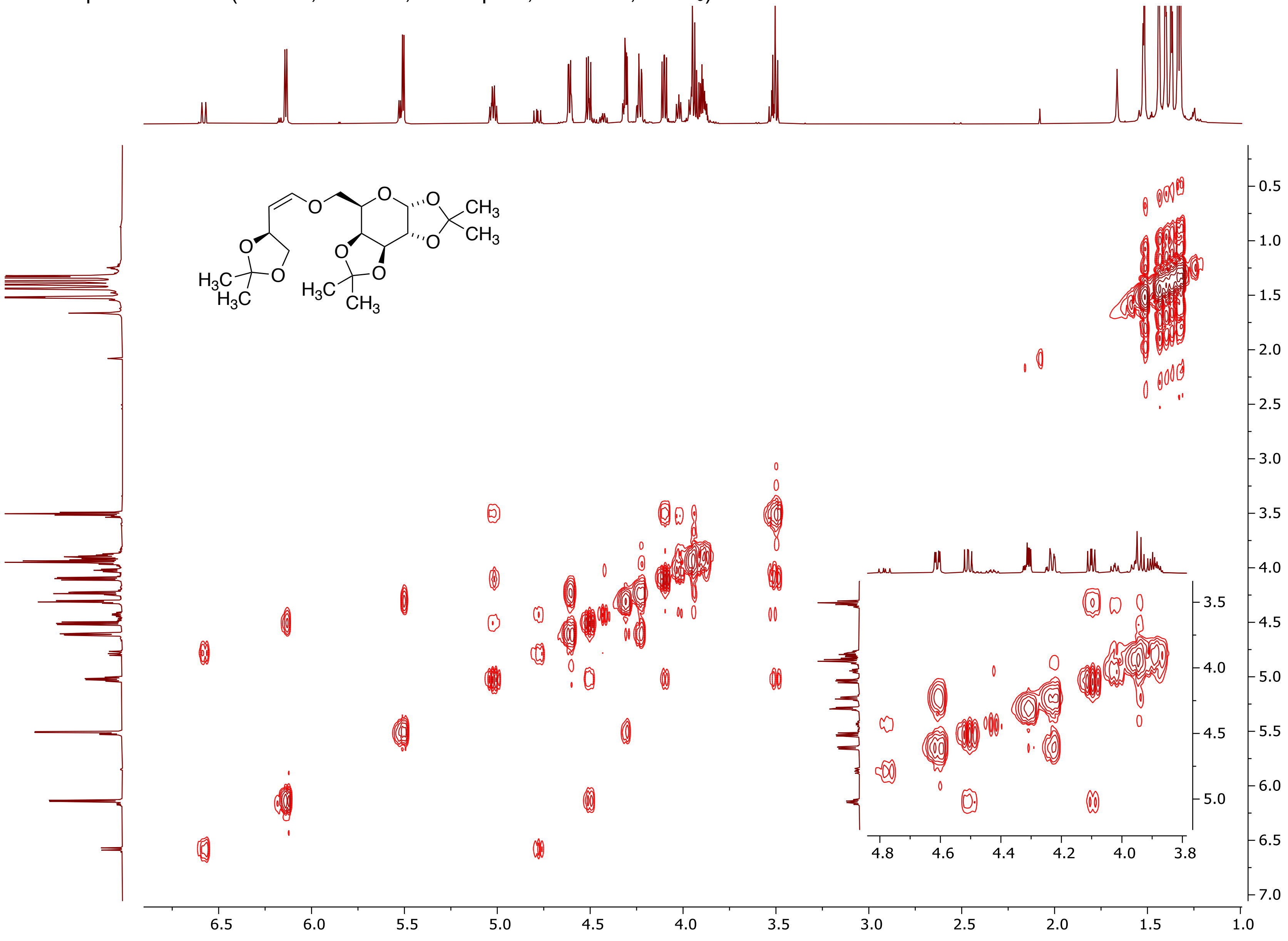

HSQC spectrum of **40** (4:1 Z/E, 10:1 d.r., >95% pure, 600 MHz, $CDCl_3$)

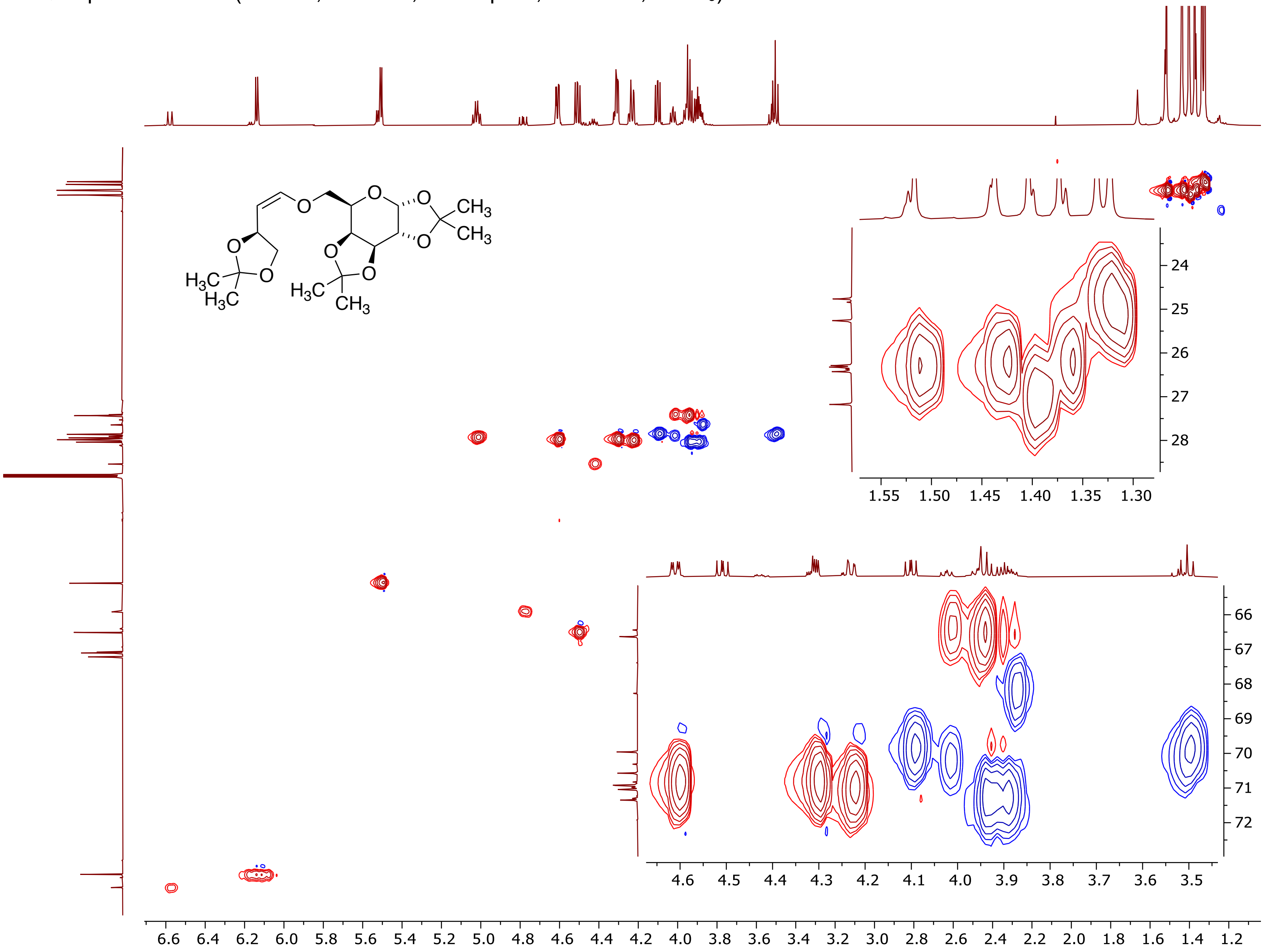

^{1}H NMR spectrum of **41** (95:5 r.r., 400 MHz, $CDCl_3$)

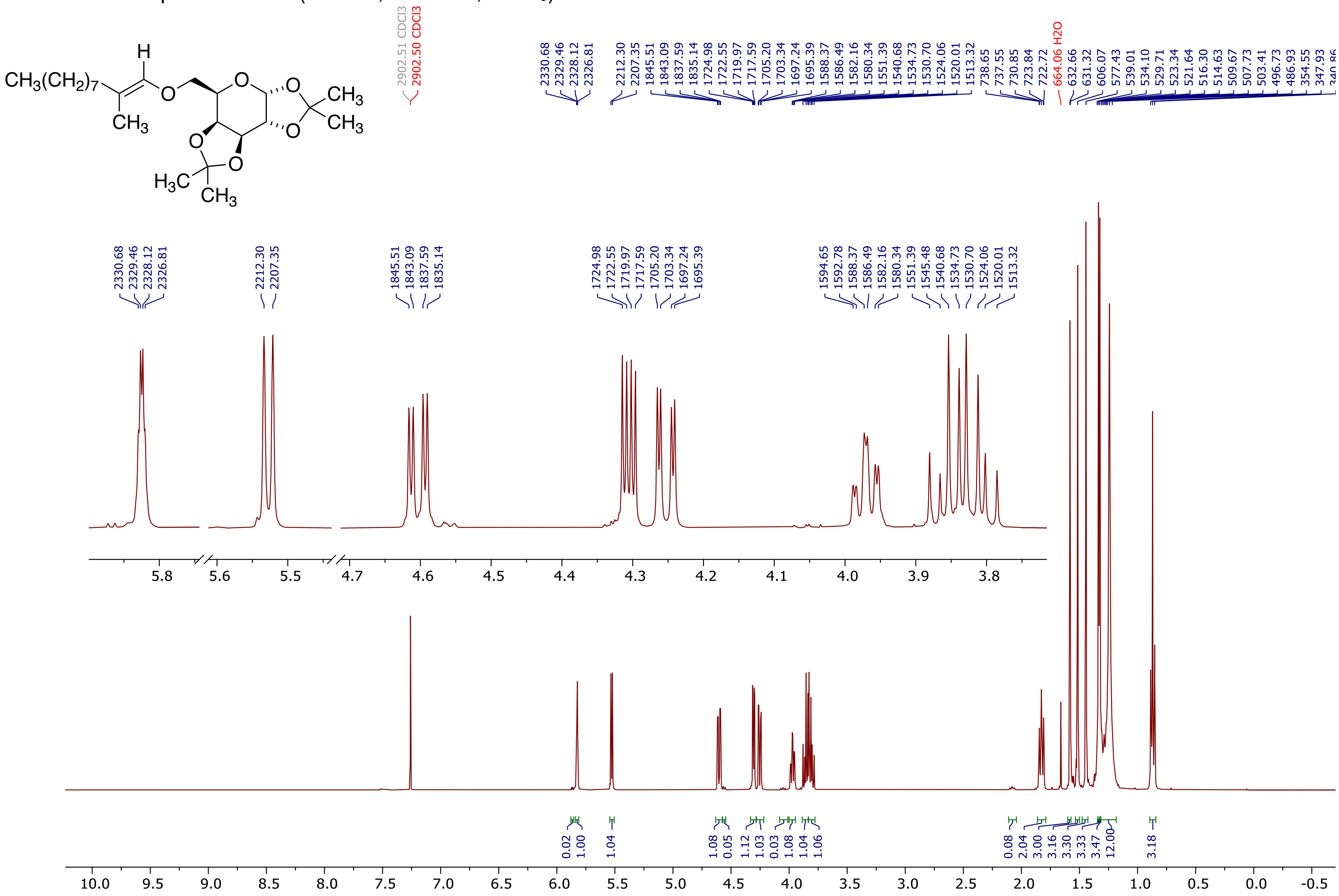

^{13}C NMR spectrum of **41** (95:5 r.r., 151 MHz, $CDCl_3$)

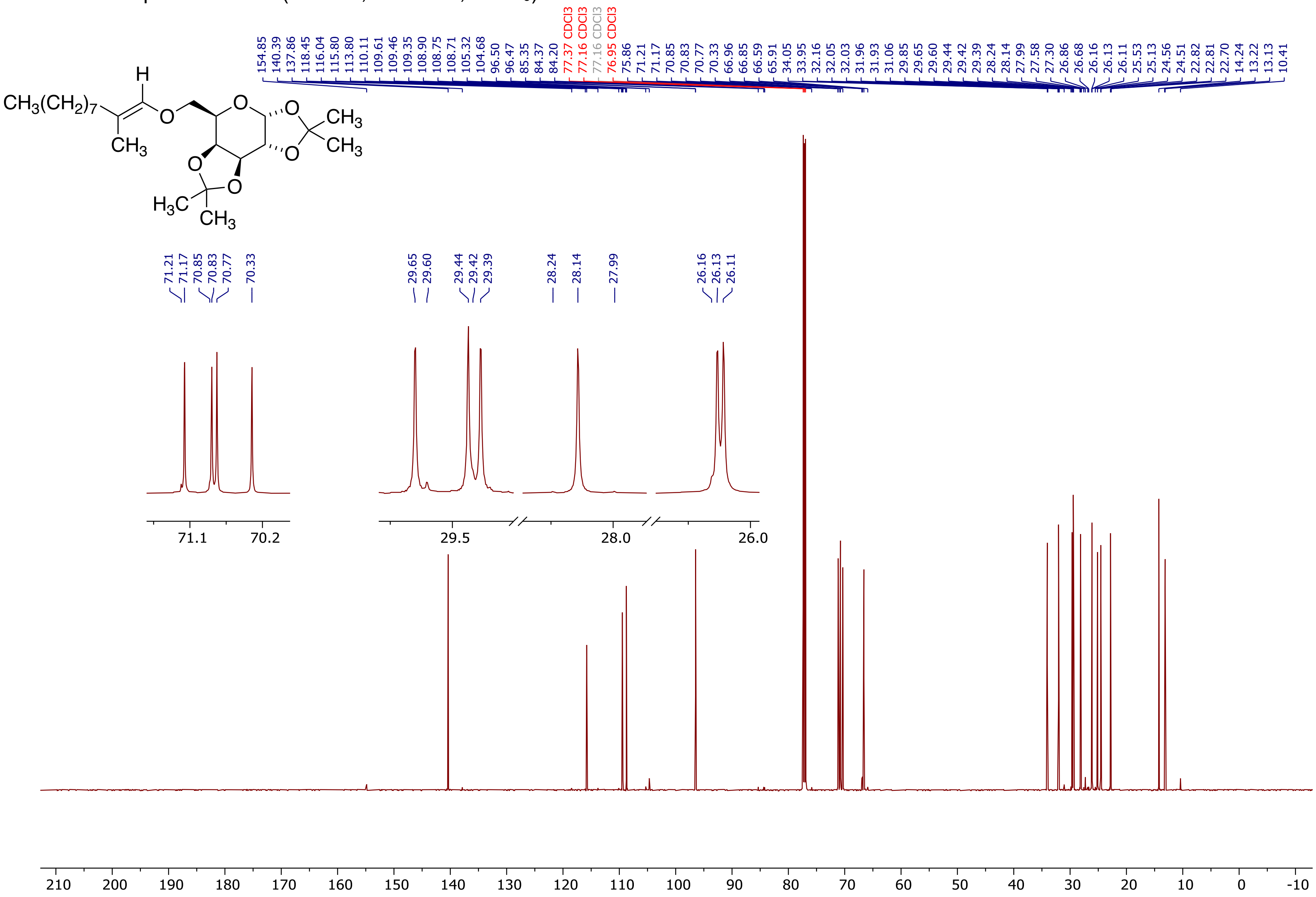

^{1}H NMR spectrum of **42** (400 MHz, $CDCl_3$)

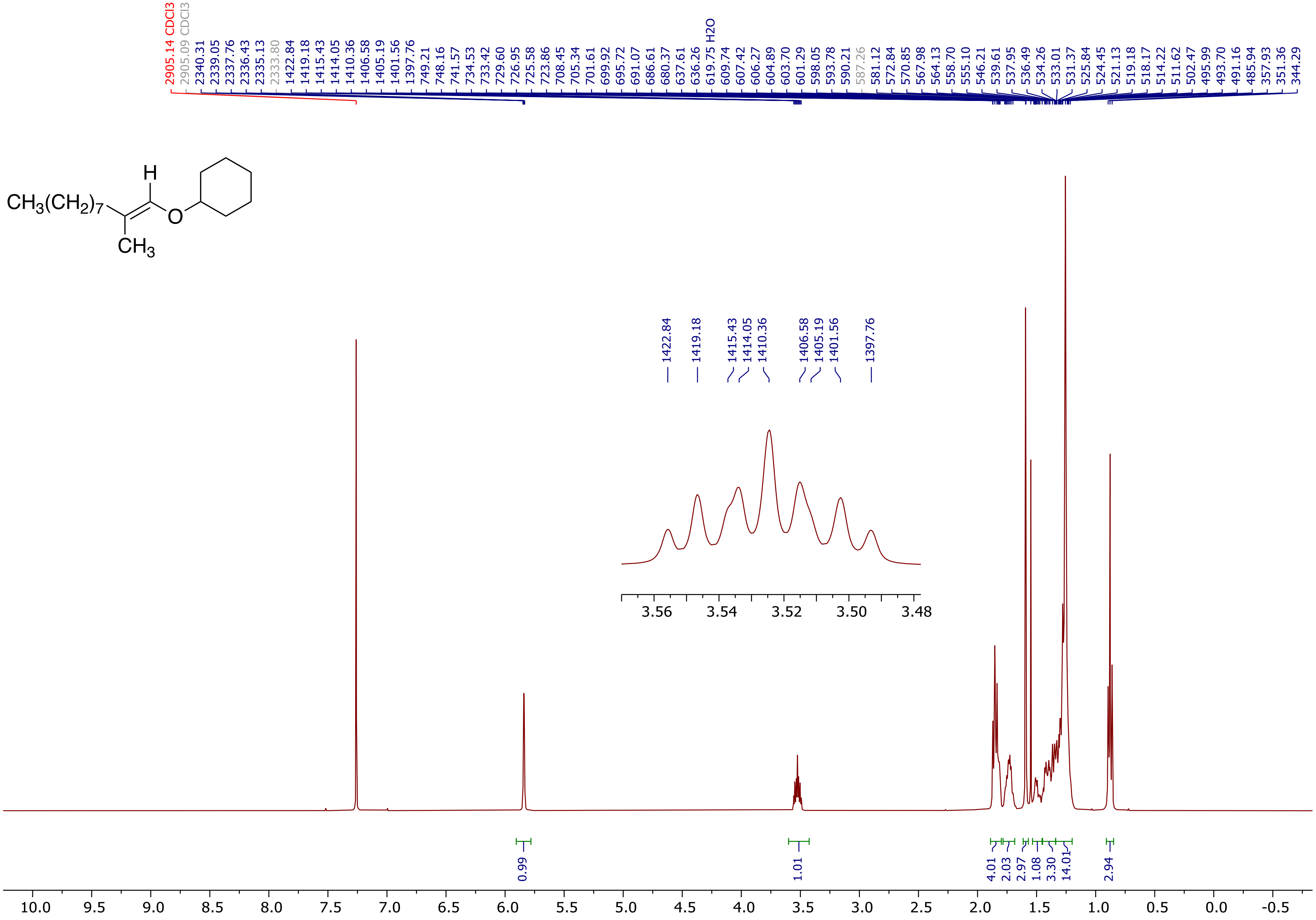

^{13}C NMR spectrum of **42** (101 MHz, $CDCl_3$)

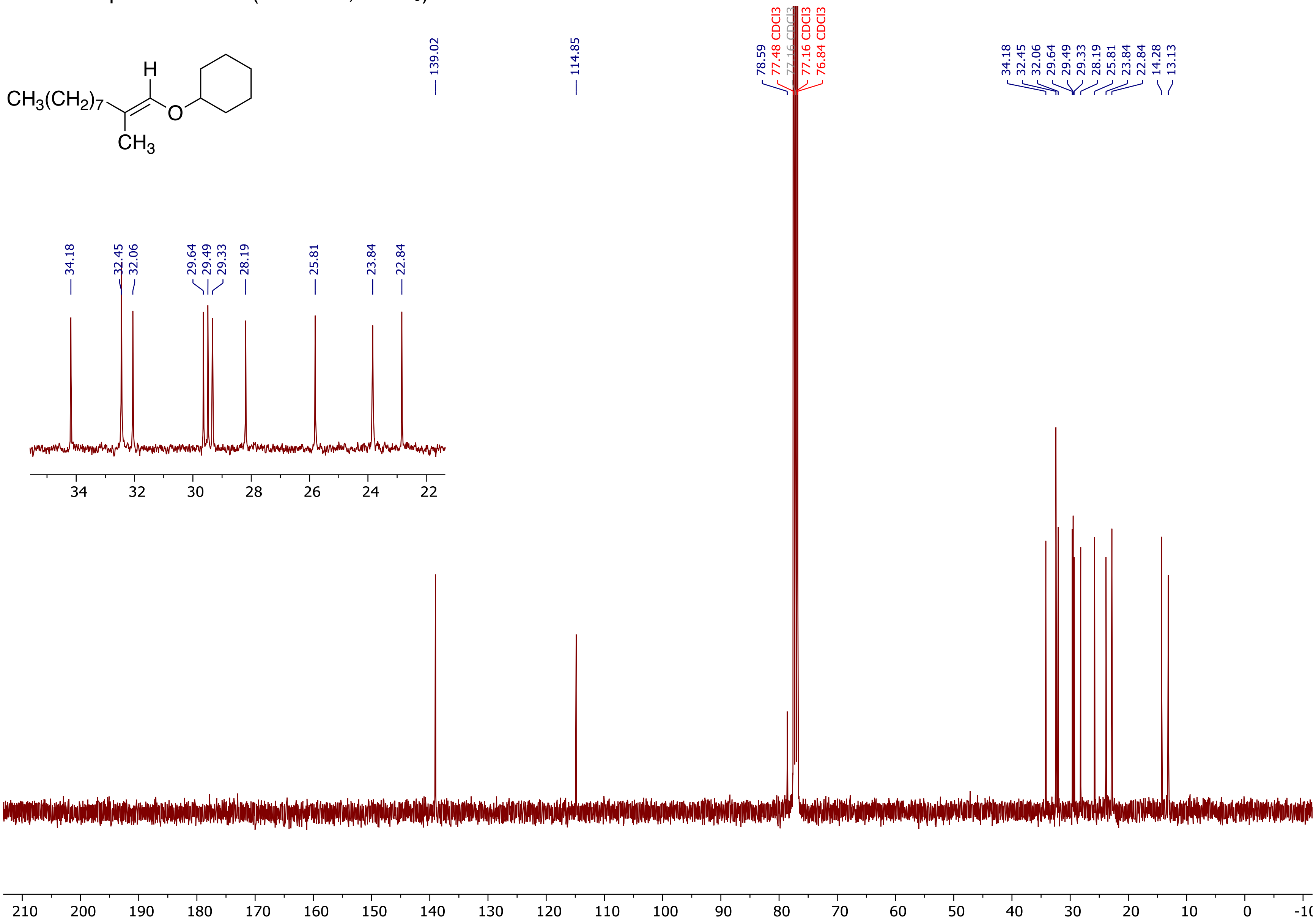

^{1}H NMR spectrum of **43** (400 MHz, $CDCl_3$)

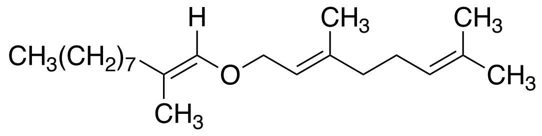

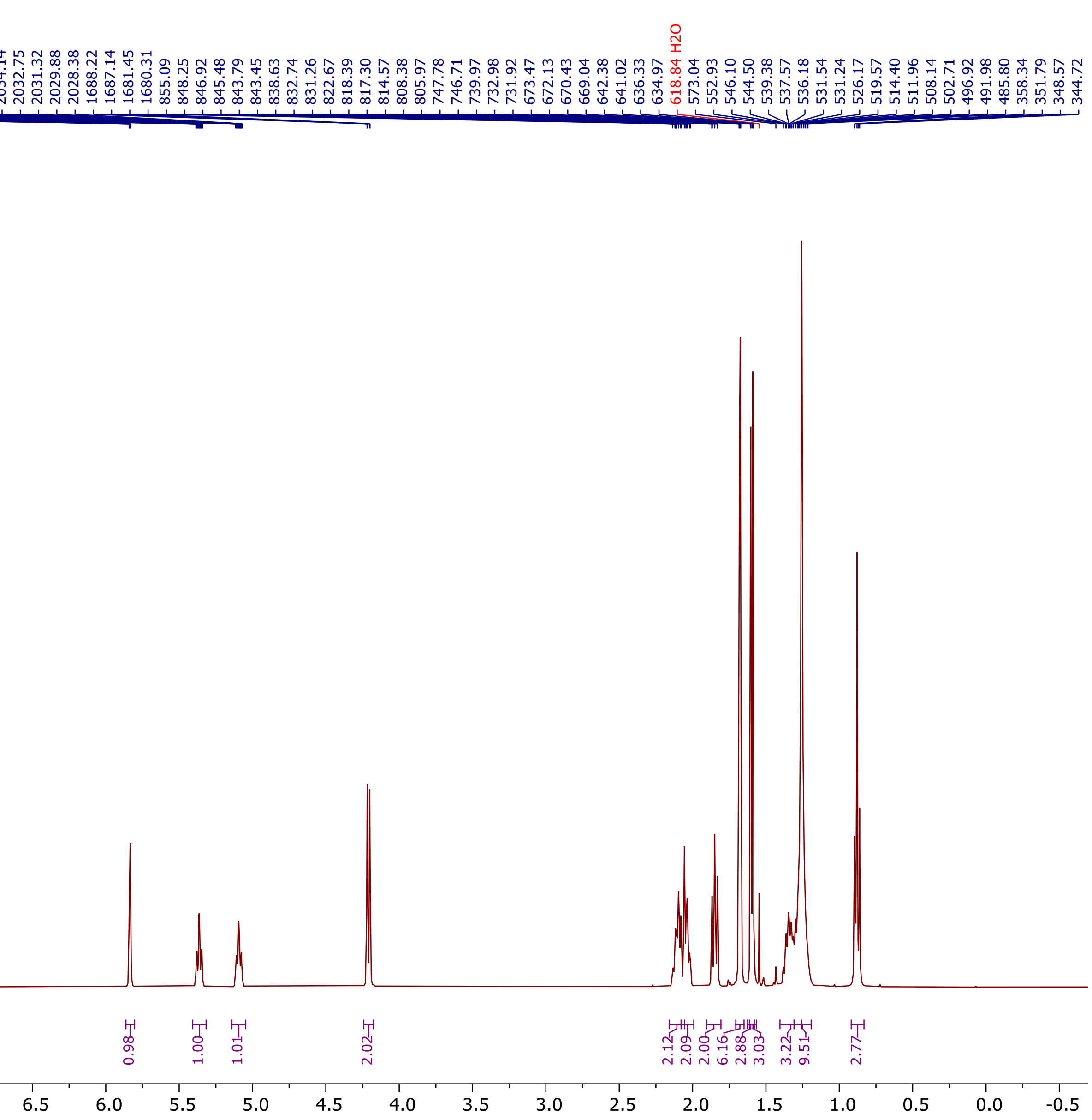

^{13}C NMR spectrum of **43** (101 MHz, $CDCl_3$)

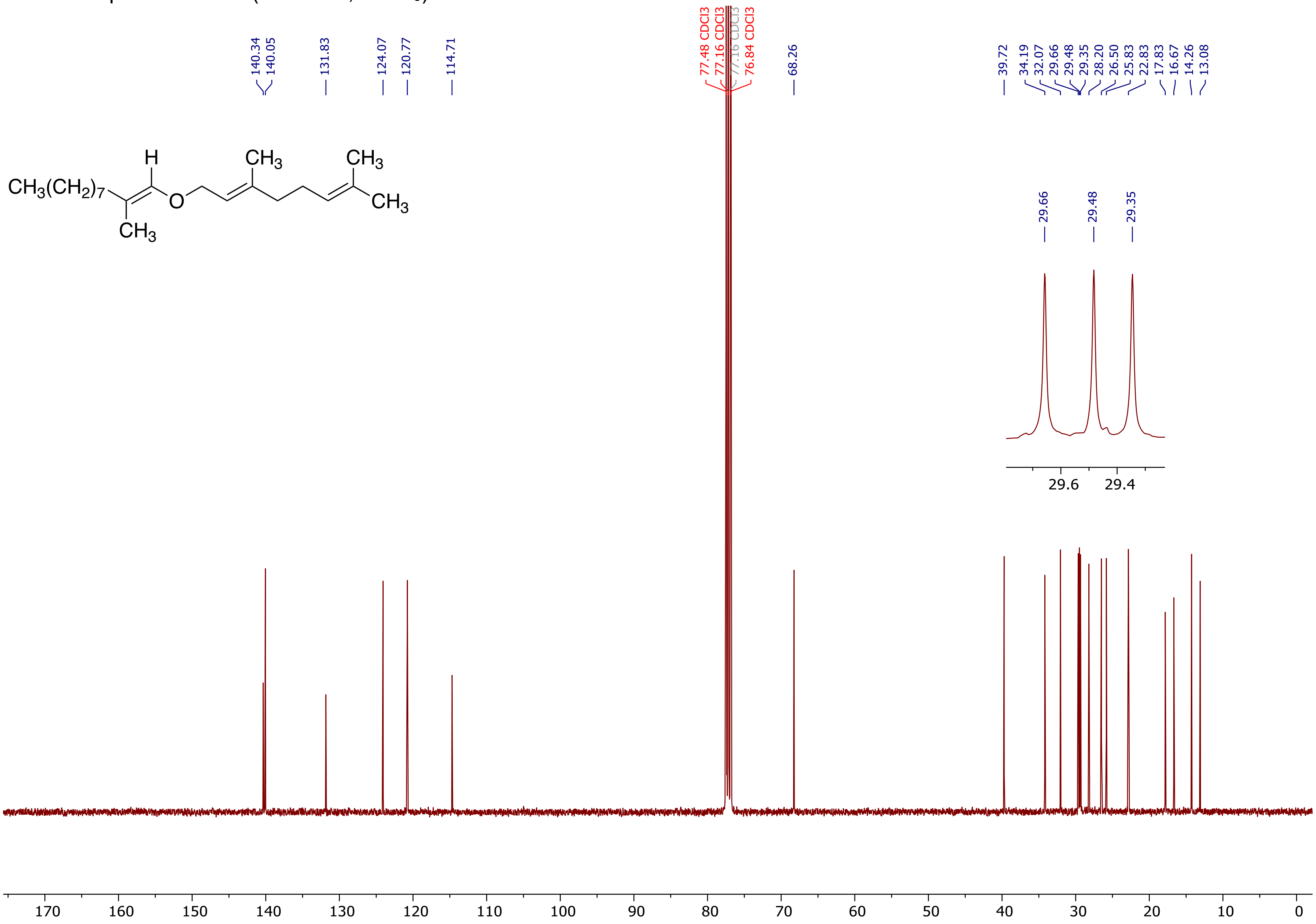

^{1}H NMR spectrum of **44** (400 MHz, $CDCl_3$)

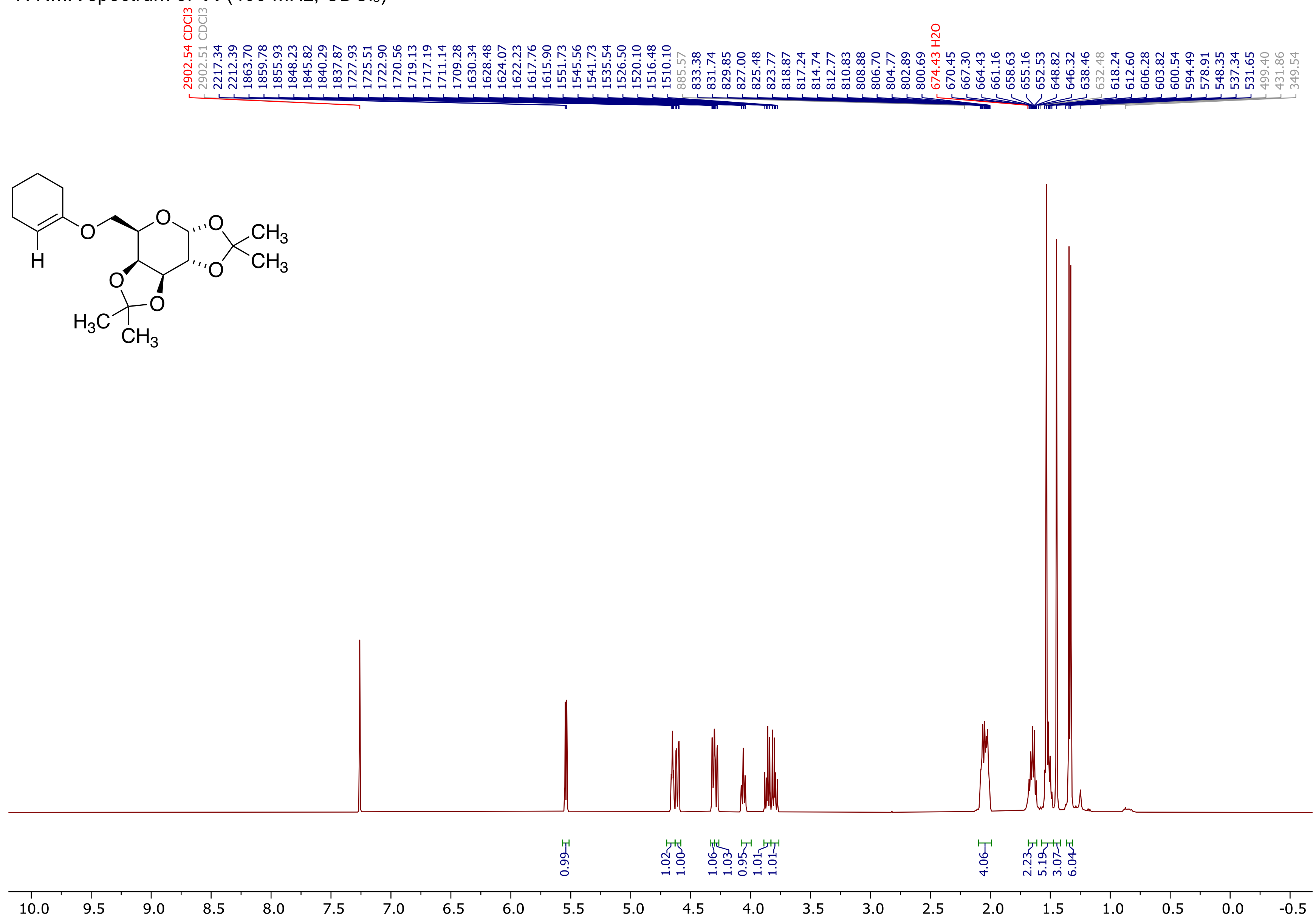

^{13}C NMR spectrum of **44** (151 MHz, $CDCl_3$)

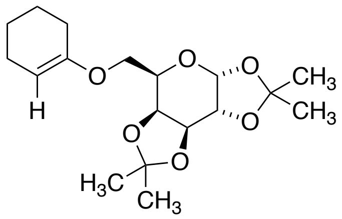

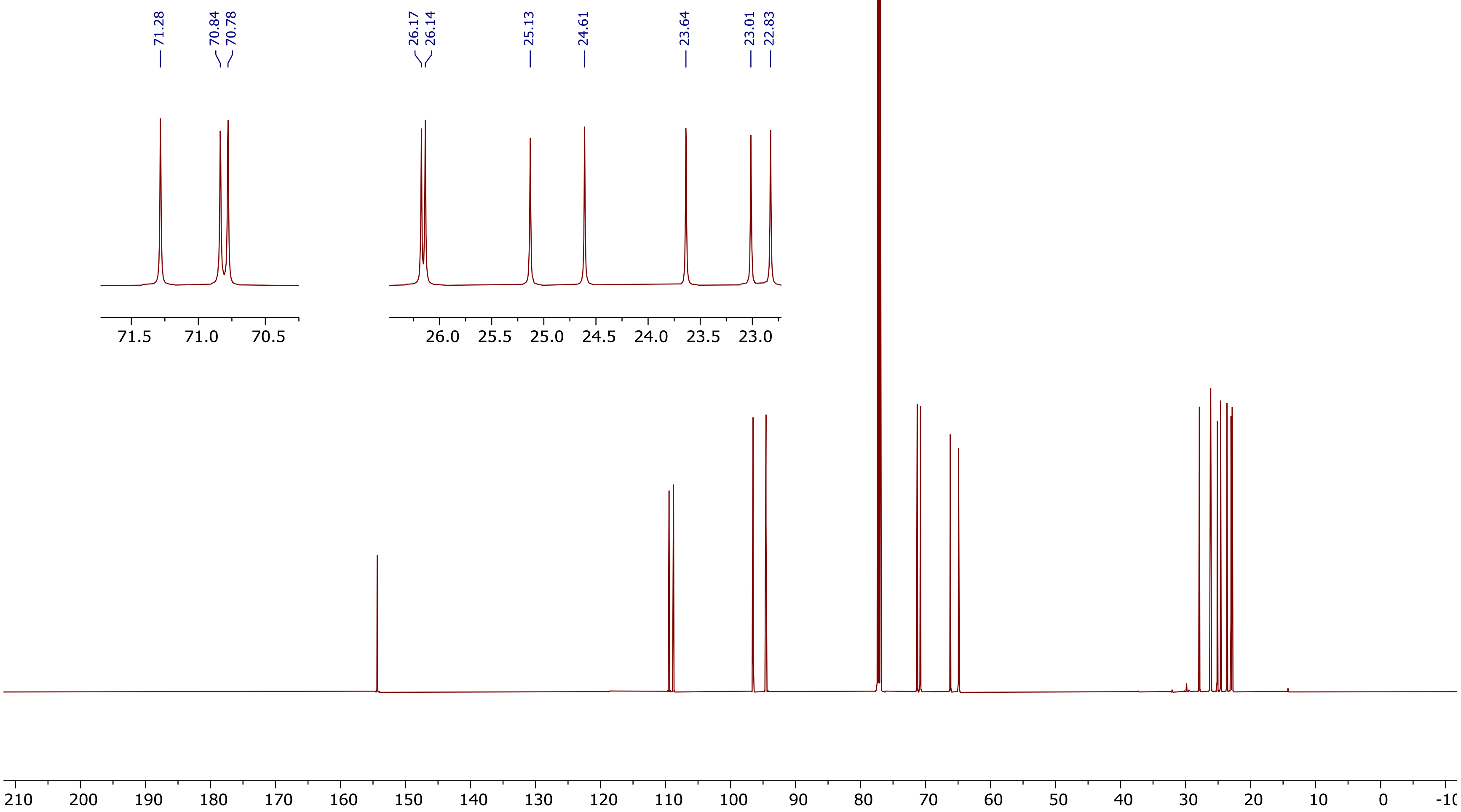

^{1}H NMR spectrum of **45** (400 MHz, $CDCl_3$)

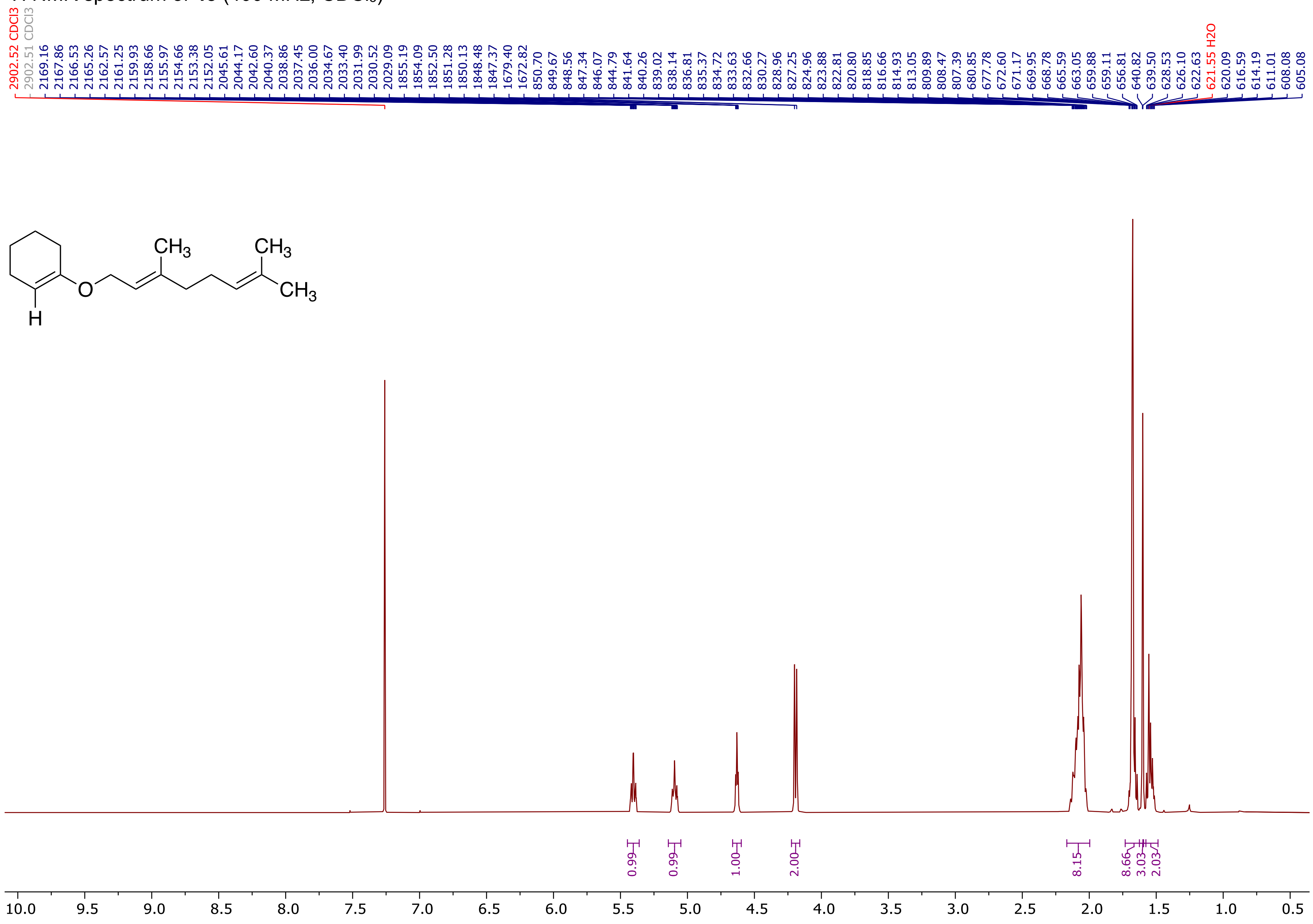

^{13}C NMR spectrum of **45** (101 MHz, $CDCl_3$)

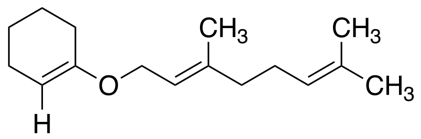

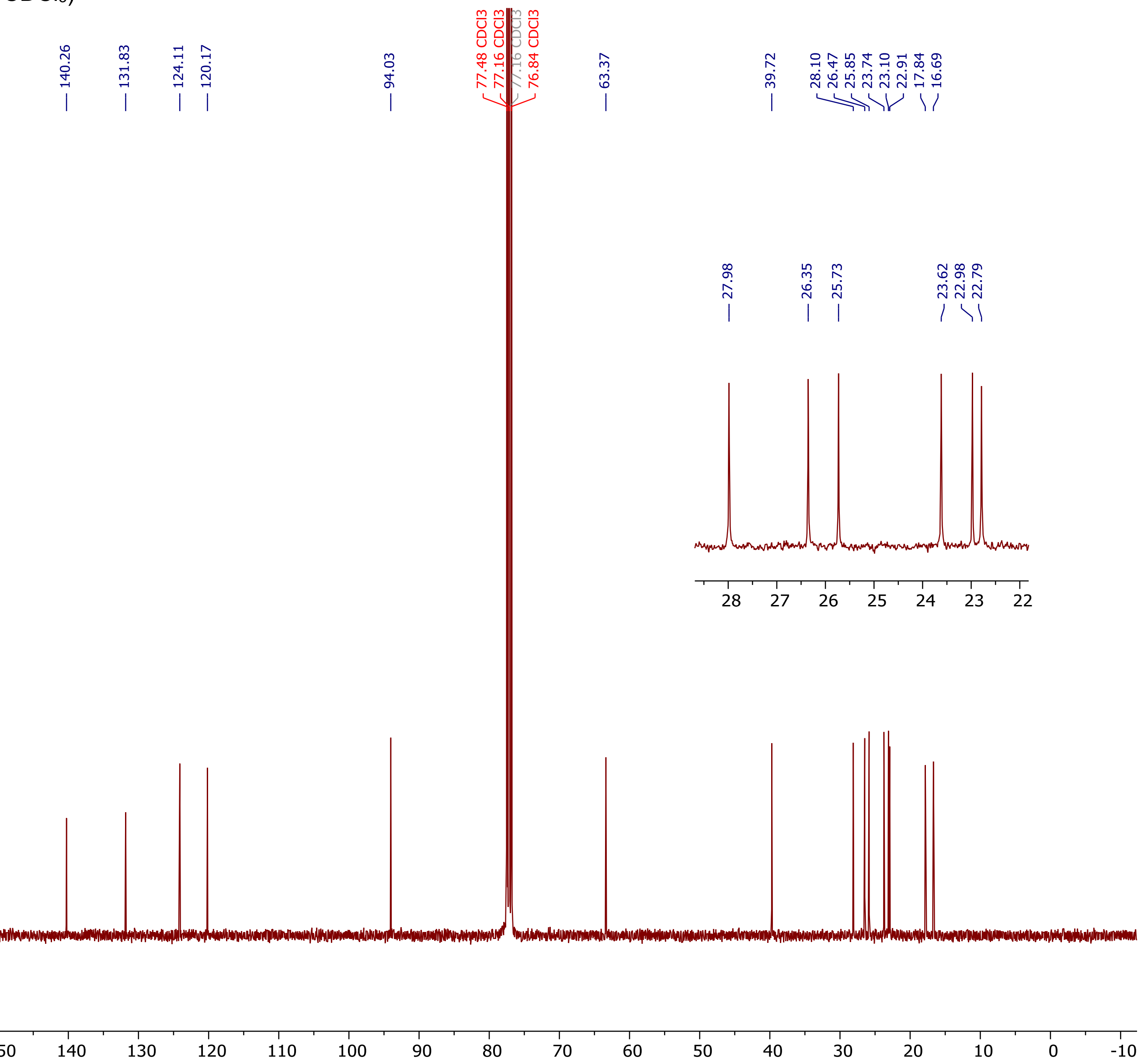

^{1}H NMR spectrum of **46** (400 MHz, $CDCl_3$)

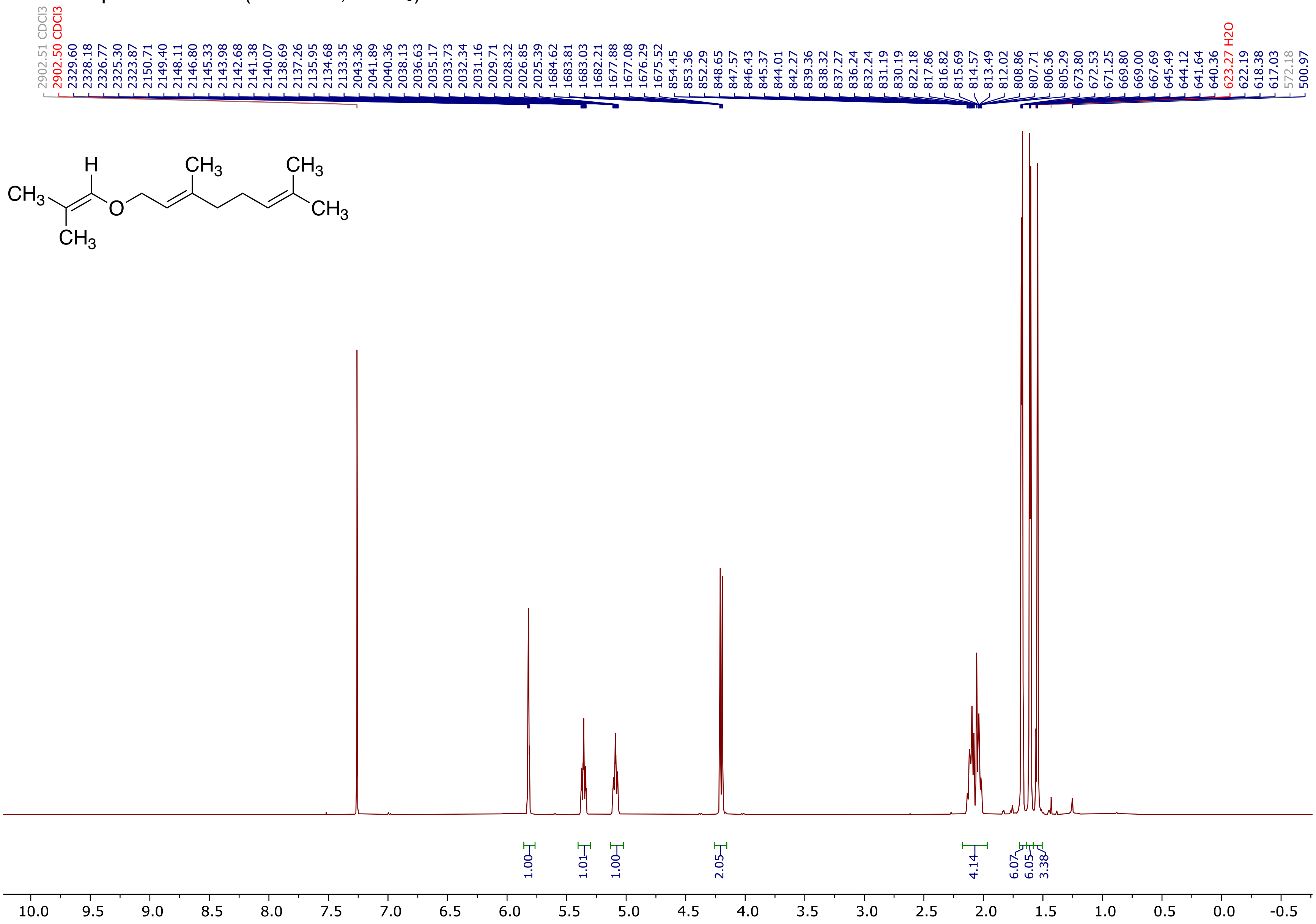

^{13}C NMR spectrum of **46** (101 MHz, $CDCl_3$)

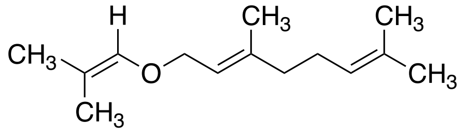

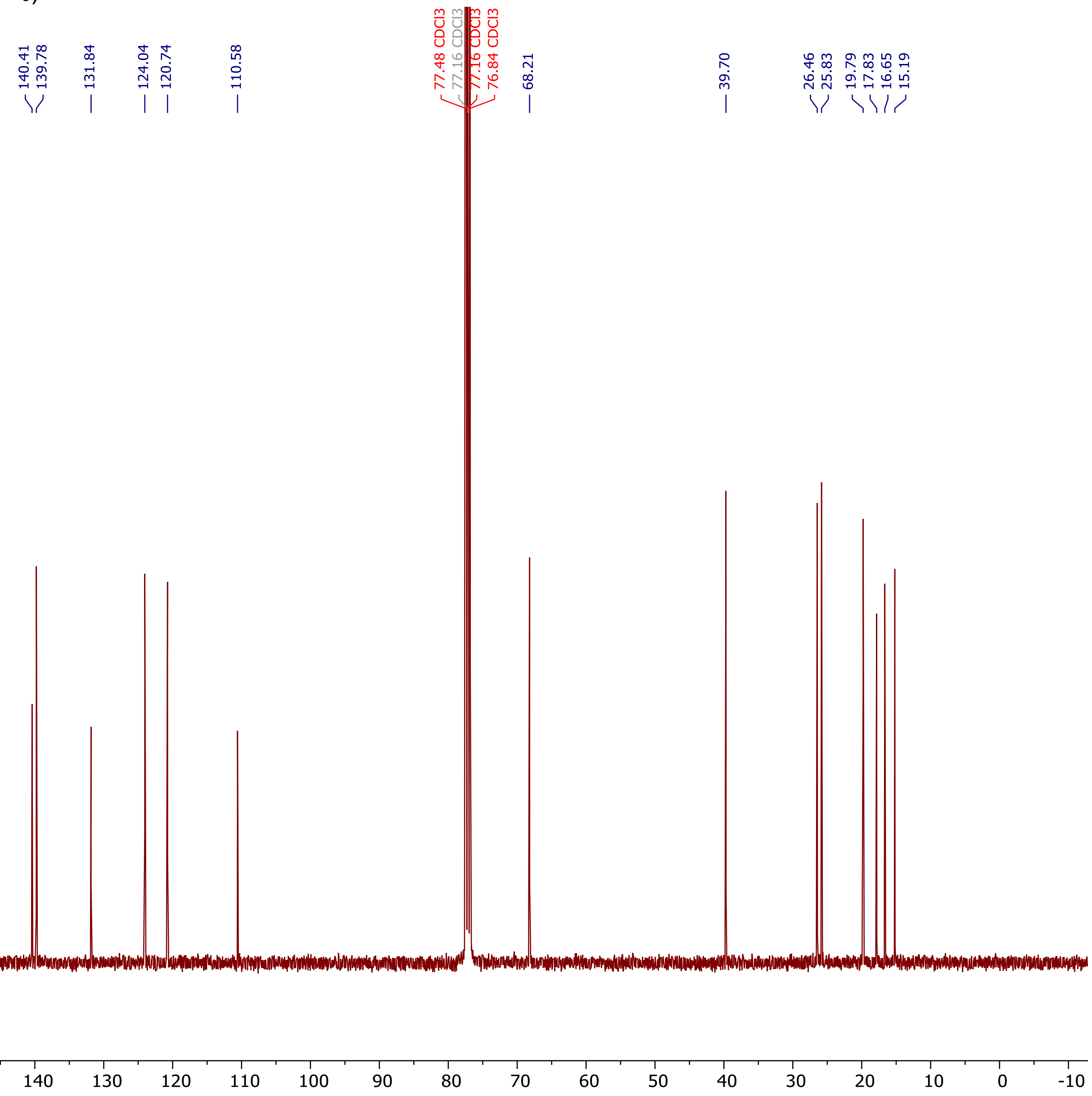

Supplement: Supplementary file 2 — ol3c01849_si_002.pdf [file ol3c01849_si_002.pdf]
