# Supplementary material for: Stereospecific Cu(I)-Catalyzed C–O Cross-Coupling Synthesis of Acyclic 1,2-Di- and Trisubstituted Vinylic Ethers from Alcohols and Vinylic Halides
Source: Org Lett. 2023 Jul 12;25(28):5297–301. doi: 10.1021/acs.orglett.3c01849 (PMC10367064; doi:10.1021/acs.orglett.3c01849)
Supplement: Supplementary file 3 — ol3c01849_si_003.zip [file ol3c01849_si_003.zip › FID for Publication/NMR general information.docx]

^1^H, ^13^C, and ^19^F NMR spectra were recorded with the following NMR spectrometers:

for Varian spectrometers, acquisition software is VnmrJ

Varian AVIII 400 (400 MHz for ^1^H NMR)

INOVA 500 (500 MHz for ^1^H NMR)

for Bruker spectrometers, acquisition software is TopSpin

Bruker NEO 400 (400 MHz for ^1^H NMR)

AVANCE 600 equipped with a cryogen probe (600 MHz for ^1^H NMR)

ASCEND 800 (800 MHz for ^1^H NMR)

NMR spectra were processed using MestReNova v14.2.1.
